# GIT2 Acts as a Systems-Level Coordinator of Neurometabolic Activity and Pathophysiological Aging

**DOI:** 10.3389/fendo.2015.00191

**Published:** 2016-01-18

**Authors:** Bronwen Martin, Wayne Chadwick, Jonathan Janssens, Richard T. Premont, Robert Schmalzigaug, Kevin G. Becker, Elin Lehrmann, William H. Wood, Yongqing Zhang, Sana Siddiqui, Sung-Soo Park, Wei-na Cong, Caitlin M. Daimon, Stuart Maudsley

**Affiliations:** ^1^Metabolism Unit, National Institute on Aging, National Institutes of Health, Baltimore, MD, USA; ^2^Receptor Pharmacology Unit, National Institute on Aging, National Institutes of Health, Baltimore, MD, USA; ^3^Translational Neurobiology Group, VIB Department of Molecular Genetics, University of Antwerp, Antwerp, Belgium; ^4^Laboratory of Neurogenetics, Institute Born-Bunge, University of Antwerp, Antwerp, Belgium; ^5^Department of Medicine, Gastroenterology Division, Duke University, Durham, NC, USA; ^6^Gene Expression and Genomics Unit, National Institutes of Health, Baltimore, MD, USA

**Keywords:** GIT2, metabolism, keystone, aging, dysglycemia, trajectory

## Abstract

Aging represents one of the most complicated and highly integrated somatic processes. Healthy aging is suggested to rely upon the coherent regulation of hormonal and neuronal communication between the central nervous system and peripheral tissues. The hypothalamus is one of the main structures in the body responsible for sustaining an efficient interaction between energy balance and neurological activity and therefore likely coordinates multiple systems in the aging process. We previously identified, in hypothalamic and peripheral tissues, the G protein-coupled receptor kinase interacting protein 2 (GIT2) as a stress response and aging regulator. As metabolic status profoundly affects aging trajectories, we investigated the role of GIT2 in regulating metabolic activity. We found that genomic deletion of GIT2 alters hypothalamic transcriptomic signatures related to diabetes and metabolic pathways. Deletion of GIT2 reduced whole animal respiratory exchange ratios away from those related to primary glucose usage for energy homeostasis. GIT2 knockout (GIT2KO) mice demonstrated lower insulin secretion levels, disruption of pancreatic islet beta cell mass, elevated plasma glucose, and insulin resistance. High-dimensionality transcriptomic signatures from islets isolated from GIT2KO mice indicated a disruption of beta cell development. Additionally, GIT2 expression was prematurely elevated in pancreatic and hypothalamic tissues from diabetic-state mice (*db/db*), compared to age-matched wild type (WT) controls, further supporting the role of GIT2 in metabolic regulation and aging. We also found that the physical interaction of pancreatic GIT2 with the insulin receptor and insulin receptor substrate 2 was diminished in *db/db* mice compared to WT mice. Therefore, GIT2 appears to exert a multidimensional “keystone” role in regulating the aging process by coordinating somatic responses to energy deficits.

## Introduction

The natural aging process is associated with an accumulation of molecular perturbations affecting almost all cells, tissues and organs of the body. These alterations have long been known to affect multiple processes related to cell survival, genomic instability, altered gene expression patterns, aberrant cellular replication, oxidative damage by reactive oxygen species (ROS), and fluctuations in protein expression and coherent protein post-translational modification (1). Life expectancy in most westernized and developing countries is demonstrating an inexorable increase (2, 3), therefore either natural or pathological aging could be considered the primary threat to global health in the future. With increasing age, there is a system-wide reduction in the ability of the body to cope with stress in-part due to a degradation of the efficiency of energy-generating [e.g., ATP (adenosine triphosphate)] metabolic systems (4–6). Disruption of the primary energy-synthesizing system, i.e., glucose catabolism by oxidative phosphorylation, leads to both ATP depletion (thus affecting electrical cellular excitability, proteolytic activities, transmembrane transport processes, and kinase activity) as well as an increase in the deleterious effects of unregulated hyperglycemia, e.g., systemic inflammation, arterial stenosis, impaired tissue healing, neuronal damage, and renal failure. Hence, one of the most characteristic factors in the aging process are the generation of insulin resistance, disruptions to oxidative phosphorylation of glucose, and changes in body fat composition (6). Therapeutic interventions, such as caloric restriction or visceral fat depletion, designed to address features of metabolic/glycemic aging, have succeeded in improving insulinotropic activity and life span in rodents (7, 8). The importance of the efficiency in glucose metabolism in the aging process is underscored by the demonstration that multiple glucose-regulatory factors in nematode worms were also major age-regulating genes, e.g., *daf-2* [insulin receptor analog (9)] and *age-1* [phosphoinositide 3-kinase analog – a primary downstream factor of insulin receptor functions (10)]. Translation of these fundamental insulin/glucose-mediated life span alterations from nematodes to higher organisms has proven problematical: a situation likely due to the increased functional diversity of insulin/insulin-related ligands in more complex, higher organisms. Despite this however, adipose tissue-specific insulin receptor deletion is associated with life span alterations in mice (11). Linked to this observation, experimental paradigms in rhesus macaques that attenuate age-related glycemic disruption and increase insulin sensitivity (i.e., caloric restriction) can significantly reduce the incidence of age-dependent pathologies (12).

Due to the gradual loss of metabolic integrity with increasing age, the body becomes more prone to a variety of pathophysiologies linked to energy insufficiency such as neurodegeneration, metabolic syndrome (MetS), and chronic inflammation. These accumulated and progressive changes in complex physiological systems such as the endocrine or central nervous system (CNS) are highly likely to be mediated by gene and protein groups that are trophically controlled by “keystone” or “hub” factors that orchestrate the communication between lower-complexity functional signaling networks (13). Considerable research demonstrates that both neurodegenerative diseases and pathophysiological aging involve a functional interplay between a series of diverse biological systems including neurological, endocrinological, sensory, and metabolic activities (13–23). Many of these systems are functionally integrated together in one crucial CNS organ, i.e., the hypothalamus (24). The hypothalamus is responsible for the regulation of many metabolic pathways by synthesizing and secreting numerous neuronal hormones that stimulate or inhibit the secretion of trophic hormones from the anterior pituitary. The hypothalamus therefore can control global metabolism, body temperature, thirst, hunger, fatigue, and circadian rhythms (25). Not only does the hypothalamus act as a trophic master-controller of the endocrine system but it also possesses neuronal projections to many autonomous and higher centers of the brain (26), thereby providing a crucial link between aging and age-related disorders such as dementia (27). We have previously shown, through the development of *in cellula* aging models (19), that regulation of the G protein-coupled receptor kinase interacting protein 2 (GIT2) is highly sensitive to the chronological aging process and also to the presence of age-linked stressors such as ROS. Using a combinatorial informatics and proteomics approach, we demonstrated that GIT2 acts as a hypothalamic aging “keystone” factor (13). Subsequently, GIT2 has also been implicated in regulating DNA integrity via its interaction with ataxia telangiectasia-mutated (ATM), a DNA damage repair (DDR) kinase (18). In response to multiple forms of DNA damaging insults, GIT2 is rapidly phosphorylated by ATM and then forms a complex with multiple DDR proteins including MDC1, MRE11, and phosphorylated H2AX (18). Genomic deletion of GIT2 in mice (GIT2KO) resulted in a significant increase in the rate of DNA damage accumulation compared to wild type (WT) age-matched control mice (18). DNA instability has been associated with an advanced rate of aging for several decades (28–30) and this has been reinforced with the appreciation that accelerated molecular aging occurs in patients possessing mutations in multiple DDR proteins including ATM (31), WRN [Werner syndrome, RecQ helicase-like (32, 33)], and BLM [Bloom syndrome, RecQ helicase-like (34)]. Glucose-related diabetic pathologies often result in elevated cellular levels of ROS due to an attenuated glucose uptake capacity and support of oxidative phosphorylation causing cells to generate ATP via other less-efficient synthetic mechanisms. Prematurely elevated ROS levels, as with mutations in DDR proteins, are considered to be one of the major causes of advanced aging (35–37). Reinforcing the link between dysfunctional aging, diabetic pathologies, and DNA instability, DDR proteins such as ATM are repeatedly linked to impaired insulin secretion capacity and diabetic pathophysiologies (38–41). Therefore, given the demonstration of a crucial hypothalamic function of GIT2 both in the aging process and DNA integrity management, we investigated the potential role of this G protein-coupled receptor (GPCR)-interacting protein in controlling/coordinating glycemic or metabolic functions in the context of aging. We have analyzed the molecular metabolic signatures of pathological aging in mice at a relatively early phase of life, i.e., 2–8 months of life (approximately 10–30% of life span), in a similar vein to the human aging trajectory study of Belsky et al. (42). In the study of Belsky et al., it was suggested that research into the molecular mechanisms of aging are significantly confounded, both with animal models and human patients, by the accumulation of extraneous co-pathologies during life span (42). However, it is also likely that dysfunctional molecular aging paradigms are set in place during early life that effectively define either a pathological or “less-pathological (healthy)” aging trajectory (43–45). Here, we have found that high-dimensionality molecular transcriptomic signatures of pathological aging and pro-diabetic pathologies occur early in life and that GIT2 controls global somatic metabolism, circulating levels of multiple plasma energy-regulating hormones and pancreatic beta cell functionality.

## Materials and Methods

### Cell Culture

Murine Beta-TC-6 cells (ATCC^1^) were maintained in a humidified 5% CO_2_ environment at 37°C in Dulbecco’s modified Eagle’s medium (DMEM) media (Life Technologies, Carlsbad, CA, USA) supplemented with 15% heat-inactivated fetal bovine serum and 1% penicillin–streptomycin (Life Technologies, Carslbad, CA, USA). Murine GIT2 siRNA (Santa Cruz) was generated as a pool of three target-specific 19–25 nt siRNAs. Control siRNA-A consists of a scrambled sequence that does not induce specific degradation of any cellular mRNA. Cells were transfected with siRNA oligonucleotides using Lipofectamine RNAi MAX (Life Technologies, Carlsbad, CA, USA) according to the manufacturer’s protocol. To assess cell viability after any specific long-term treatment, cells were first washed in phosphate buffered saline (PBS: Sigma-Aldrich, St. Louis, MO, USA) and then collected from the growth plates using calcium-free, non-enzymatic cell dissociation solution (Sigma-Aldrich, St. Louis, MO, USA). Cells were gently pelleted by centrifugation at 1000 × *g* at room temperature before being resuspended in a Hanks’ Balanced Salt solution (cat.#H9269: Sigma Aldrich) and mixed with a 0.4% Trypan Blue solution and allowed to incubate for 15 min at room temperature. Cells were then transferred to a Bright-Line™ hemacytometer (Sigma-Aldrich) and a percentage of positive blue cells (representing cells possessing a severely compromised plasma membrane and thus at high risk of imminent death) were counted by an experienced researcher. As a positive control for loss of cell viability, cell cultures were pre-incubated with a 2 μg/ml concentration of actinomycin D (Sigma-Aldrich) for 2 h before collection and Trypan Blue processing as described here previously.

### Experimental Animal Models

G protein-coupled receptor kinase interacting protein 2 gene-trap knockout (KO) mice (GIT2KO), were originally obtained from Dr. Richard T. Premont (Duke University Medical Center, Durham, NC, USA) and genotyped using genomic PCRs as previously described (46). PCR amplification was performed by standard protocols. Primers used to screen the GIT2 knockout mice were as follows: forward primer 5′-TCTCCTGGAACTCAGGGATT, reverse primers (WT) 5′-CATTTCAGAGTCTGCTGCCTTA and (KO) 5′-GGCTACCGGCTAAAACTTGA. Male WT (WT: C57BL/6) and GIT2KO mice were group-housed (genotypes were housed separately) in temperature-controlled (22°C) and humidity-controlled (45%) rooms with a 12 h light–dark cycle with food and water available *ad libitum*. Male leptin receptor-mutant (*db/db*) mice, bred on a C57BL/6 background, were purchased from The Jackson Laboratories.^2^ All experimental mouse models were fed using a standard control animal chow containing 19% protein, 64% carbohydrates, and 17% fat (diet #101845 from Dyets Inc., Bethlehem, PA, USA). Experiments were conducted during the light phase of the light–dark cycle in accordance with NIH guidelines. Animal care and experimental procedures followed NIH guidelines and were approved by the National Institute on Aging Animal Care and Use Committee (433-LCI-2015).

### Whole Animal Glycemic Function Testing

Standard insulin tolerance tests (ITT), applied according to Jackson Laboratory handling protocols,^3^ involved blood glucose sampling from a distal tail snip after the specified time (0–90 min) of a subcutaneous Lantus^®^ (0.5 U/kg) bolus. Standard oral glucose tolerance tests (OGTT) were made using standardized protocols (47–49). Briefly, mice were fasted for 4 h, and blood was drawn from the tail and gathered using a heparinized micro-hematocrit capillary tube (Fisher Scientific, Pittsburgh, PA, USA), and clotisol (Agri-Med, St. Louis, MO, USA) was used to stop the bleeding. Mice were then administered with glucose (2 g/kg) via gavage and blood samples drawn at the specified time in minutes after glucose administration. For all time points, whole-blood glucose concentration was quantified at the time of blood draw using a Freestyle Glucose meter (Abbott Laboratories, Abbott Park, IL, USA). Blood samples were then centrifuged at 12,500 rpm for 12 min at 4°C, and plasma supernatant was removed and stored at −80°C. Insulin was quantified using a commercially available ELISA kit (Crystal Chem Inc., Downers Grove, IL, USA).

### Whole-Body Murine Metabolic and Behavioral Analyses

Murine behavior and metabolic parameters were measured, over a 48-h period, using a Columbus Instruments Comprehensive Lab Animal Monitoring System (CLAMS). The CLAMS system was used to evaluate food intake (gram), water intake (milliliter), *X*, *Z* total activity (counts), O_2_ consumption (milliliter/kilogram/hour), CO_2_ production (milliliter/kilogram/hour), and respiratory exchange ratio (RER = VCO_2_/VO_2_), as described previously with modifications (50–52). Eight mice per group were single housed for 24 h in the system. The mean weight of WT-control mice (27.25 ± 0.69 g) was slightly less but not significantly different from the GIT2KO counterparts (29.06 ± 1.17 g). Statistical analysis was performed using a two-tailed Student’s *t*-test, and *p* ≤ 0.05 was considered statistically significant.

### Measurement of Glucose, Lipids, and Hormone Levels

Blood was collected after the mice were euthanized with isoflurane. Eight to ten animals were included in each group. Glucose levels were measured using an EasyGluco blood glucose monitoring system (US Diagnostics, Inc.). For fasting glucose measurements, mice were food-deprived 12 h prior to blood collection. Plasma insulin, leptin, peptide YY (PYY), gastric inhibitory peptide (GIP), amylin, and pancreatic peptide (PP) levels were measured using Linco-Millipore multiplex kit (53, 54). Briefly, plasma samples were incubated together with antibody conjugated beads for 16 h at 4°C. After three times washing, biotinylated detection antibody was added: these were further incubated for 1 h at room temperature, and then streptavidin–horseradish peroxidase was added. Fluorescent signals were detected by Bio-Plex 200 systems. Each sample was assayed in duplicate on a 96-well plate. Hormone levels were derived by interpolation from a reference curve generated in the same assay with reference standards of known concentrations of the detected hormones.

### Pancreatic Tissue and Islet Extraction

As described previously (53), mice were euthanized with isoflurane followed by decapitation. Whole pancreata were removed from each animal. The pancreata were then processed differentially concerning their eventual usage. For immunohistochemical analyses, the pancreata were fixed in 4% formalin for 48 h and stored in PBS until processing. The pancreatic tissue was processed and embedded in paraffin wax. Pancreatic sections were cut at 5 μm thickness using a microtome, and the sections were adhered to poly-l-lysine-coated microscope slides (Fisher, Springfield, NJ, USA). For analysis of insulin- (beta cells) and glucagon- (alpha cells) secreting islet cells, pancreatic sections were immunostained according to a previously described protocol (53). Briefly, the tissue was incubated with the primary insulin antibody (guinea pig anti-swine insulin; 1:300; DakoCytomation, Carpinteria, CA, USA) or glucagon antibody (1:500; guinea pig anti-glucagon; Millipore, Billerica, MA, USA) for 2 h at room temperature and then incubated with secondary antibody [Alexa Fluor 488 (AF488) goat anti-–guinea pig, 1:200; or Alexa Fluor 568 (AF568) goat anti–guinea pig, 1:1,000; Life Technologies, Carlsbad, CA, USA] for 1 h at room temperature. Sections were imaged with an Olympus Fluoview IX70 microscope (Olympus America, Center Valley, PA, USA). Quantification of immunohistochemistry images was performed in MATLAB (Mathworks) using novel software in conjunction with the image processing toolbox. Intensity readings of each image ranged from 0 to 256, with 256 being the greatest pixel density and hence the highest staining intensity. The region of interest (ROI) was drawn around each islet after background subtraction. The pixels within the bounds of the ROI and above the set threshold of eight were selected, from which the actual islet area was calculated. The normalized variance of the ROI was used to calculate an artificial ellipse from which the major and minor axes were determined. The major axis is the longest diameter that can be drawn in the ellipse, and the minor axis is the shortest diameter, both giving an accurate approximation for the range of the actual islet diameter. Islet morphometry and sizing analyses were performed in an unbiased, random fashion (55). Specific murine islets were isolated using collagenase digestion, using the same protocol as described previously (56). Murine islets suspended in DMEM-containing 5 mM glucose and 1% bovine serum albumin (BSA) were pelleted and stored before extraction of total RNA for microarray analysis.

### Bioinformatic Analyses

Microarray-derived gene transcript lists were initially separated into specific expression polarity regulation groups using VennPlex venn diagram analysis (57) before pathway analysis using multiple forms [KEGG pathway using WebGestalt (58), Ingenuity Pathway Analysis (IPA) Canonical Signaling, Textrous!-based natural language processing (59)] of functional annotational clustering. For KEGG and IPA Canonical Pathway signaling analysis, we employed a cutoff of at least two transcripts (from the original filtered/analysis of variance datasets) needing to be present to minimally populate a particular canonical IPA or KEGG pathway with a probability (*p*) of enrichment value of ≤0.05. Where stated, a hybrid score process was employed to generate a single index for a specifically enriched KEGG pathway. Hybrid scores were generated by the multiplication of the negative log_10_ of enrichment probability (*p*) with the enrichment factor (*R*). Latent Semantic Indexing (LSI)-based analysis (60) was performed as described previously using Textrous! (59). Textrous! as well as other LSI-based informatics platforms (e.g., GeneIndexer), correlate the strength of association, using LSI (13, 19), between specific transcripts in a dataset with user-defined interrogation terms. Textrous! is able to investigate connections between input transcripts/proteins (using input Gene Symbols) and biomedical words and noun-word phrases using complete extracted data (in the form of curated gene-word documents) from PubMed Central,^4^ OMIM (Online Mendelian Inheritance in Man^5^), and the Mammalian Phenotypes Database at the Jackson Laboratories Mouse Genomic Informatics portal.^6^ For Textrous!-based natural language processing, the possible LSI correlation scores (termed Cosine Similarity Scores) for a gene/protein to be associated with an input interrogation term range from 0 to 1, with the stronger correlation scores approaching 1. A correlation score of ≥0.1 indicates at least an implicit correlation, between the specific gene and the user-defined input interrogation term (61). To generate a holistic and more specific high-dimensionality data annotation for Textrous!-based text outputs, significant data word clouds were created from the Textrous!-based semantic noun and noun-phrase outputs using the web-based application Wordle^7^ (62). The text size within the word clouds is directly proportional to the input word frequency. Analyses of word-phrase frequencies from Textrous! noun-phrase outputs were made using WriteWords.^8^ The use of freeform quantitative wordclouds to convey complex non-canonical signaling-activity relationships is becoming more and more recognized as a novel technique to investigate high-dimensionality data (63–65).

### Immunoprecipitation Assays

Whole murine pancreata, WT or *db/db*, were extracted through micro-dissection. For protein immunoprecipitation, cytoplasmic cell lysates were prepared using the Qproteome^®^ cell compartment kit according to the manufacturer’s instructions (Qiagen, Valencia, CA, USA) and as described here. All protein extracts were quantified using BCA reagent (ThermoScientific, Waltham, MA, USA) before normalization to a standard 1 mg/ml protein concentration before immunoprecipitation. Normalized supernatant protein lysates were pre-cleared by a 1 h incubation, tumbling at 4°C, with 30 μl of protein A/G pre-conjugated agarose beads (EMD Chemicals, Gibbstown, NJ, USA). For immunoprecipitation, the pre-cleared cell lysates were then incubated overnight for 16 h at 4°C with tumbling with 10 μg of either pre-immune IgG sera (Sigma-Aldrich, St. Louis, MO, USA) or 10 μg of anti-GIT2 antibody (Bethyl Laboratories, Montgomery, TX, USA). To collect the immunecomplexes, 30 μl of protein A/G pre-conjugated agarose beads (EMD Chemicals, Gibbstown, NJ, USA) were added to the lysates and then tumbled at 4°C for an additional hour. The agarose-associated immunecomplexes were then collected by centrifugation at 2000 × *g* for 5 min. Immunoprecipitated protein complexes were removed from the agarose-associated antibodies by the addition of 50 μl of a room-temperature CHAPS-Urea buffer [4% CHAPS ((3-((3-cholamidopropyl) dimethylammonio)-1-propanesulfonate) (Sigma-Aldrich, St. Louis, MO, USA), 8M Urea)] through gentle agitation before being stored at −80°C for further mass spectrometry (MS)-based processing.

### Mass Spectrometry

Proteins were extracted from GIT2 immunoprecipitates using 4% CHAPS-8M Urea and were analyzed using reverse-phase nano LC-MS/MS on a ThermoFinnigan LXQ linear ion MS (ThermoFinnigan, West Palm Beach, FL, USA) as described previously (66). Analytical samples were separated on an *in-house* fabricated 8-cm reverse-phase capillary emitter column or a c18 PicoFrit column (New Objectives, Boston, MA, USA), using 90 min gradients. The collision energy for the LXQ MS was set at 30%. Spectra were acquired in a data-dependent manner with the dynamic exclusion option enabled. The four most intense ions in each full MS scan were fragmented and analyzed. MS/MS spectral data were processed using MASCOT Daemon v. 2.2.2 (Matrix Science) for protein identification. For the MS/MS ion search using MASCOT, proteins with >1 peptides and a score of each peptide higher than 45 with an unambiguous identification were considered for further analysis without manual spectra inspection. Proteins with one peptide and a score lower than 45 were considered ambiguous and discarded from analysis. We used the pre-defined enzyme specificity in the Mascot Daemon v. 2.2.2. Search parameters included static mass modification to cysteine (iodoacetamide alkylation) and differential mass modification to methionine (oxidation). The decoy database option provided by MASCOT was selected to determine false discovery rates (FDR), which was kept below 1% by applying various significance thresholds to each search result.

### Western Blotting

Murine hypothalami or pancreata were extracted through micro-dissection, and cell lysates were also prepared from murine TC6 cells. For protein extraction, differential cellular compartment (cytoplasm, plasma membrane, nuclear/large organelle, and cytoskeleton) lysates were prepared using the Qproteome^®^ cell compartment kit according to the manufacturer’s instructions (Qiagen, Valencia, CA, USA). All protein extracts were quantified using BCA reagent (ThermoScientific, Rockford, IL, USA) and then normalized for each specific experiment before resolution with SDS-PAGE and semi-dry electrotransfer (Bio-Rad, Hercules, CA, USA) to PVDF membranes (PerkinElmer Life Sciences; Waltham, MA, USA). Membranes were blocked using a 4% BSA for Western blot as described previously (67), and primary antibody immune-reactive complexes were identified using alkaline phosphatase-conjugated secondary antisera (Sigma-Aldrich, St. Louis, MO, USA) with enzyme-linked chemifluorescence (GE Healthcare) and visualized with a Typhoon 9410 phosphorimager (GE Healthcare). Proteins were identified using primary antisera at 1:1,000 to 1:10,000 dilutions, followed by species-specific alkaline phosphatase-conjugated secondary antibodies (Sigma-Aldrich, St. Louis, MO, USA) at 1:7,000 dilution. The primary antibodies employed in this study are described in the next section.

### Experimental Antibodies

Primary antibodies specific for diablo homolog (*Drosophila*) (Diablo: ab8114), glyoxalase 1 (Glo1: 6F10: ab81461), sestrin 1 (Sesn1: ab103121), insulin receptor (Insr: ab5500), insulin receptor substrate 2 (Irs2: ab134101), p53 and DNA damage regulated 1 (Pdrg1: ab74500), plasma membrane Ca^2+^ ATPase (Pmca: ab2825), and tubulin alpha-1A (Tuba1a: ab76449) were obtained from Abcam (Cambridge, MA, USA). Primary antibodies specific for phosphatidylethanolamine binding protein 1 (Pebp1: clone EPR2875Y: C105420) and high mobility group nucleosomal binding domain 2 (Hmgn2: C151267) were obtained from LSBio (Seattle, WA, USA). Primary antibodies specific for receptor accessory protein 5 (Reep5: H-76), extracellular signal-regulated kinase 2 (Erk2: C-8), glucose transporter 2/solute carrier family 2 (facilitated glucose transporter), member 2 (Glut2/Slc2a2: H-67), glyceraldehyde-3-phosphate dehydrogenase (Gapdh: I-19), mitochondrial ribosomal protein L12 (Mrpl12: 397.1), F-box protein 24 (Fbxo24: D-14) microtubule-associated protein 1 A (Map1a: N-18), CASK-interacting protein 2 (Caskin2: P-13), and lamin A (Lmna: C-20) were obtained from Santa Cruz Biotechnology (Santa Cruz, CA, USA). Primary antibodies specific for doublecortin (Dcx: #4604), ataxia telangiectasia mutated (Atm: #2873), beta-PIX (Arhgef7: #4515), and p21-activated kinase 1 (Pak1: #2602) were obtained from Cell Signaling Technology (Danvers, MA, USA). The primary antibody specific for regenerating islet-derived 3 beta (Reg3b: Clone 518630) was obtained from R&D Systems (Minneapolis, MN, USA). Primary antibodies specific for NADH dehydrogenase (ubiquinone) 1 beta subcomplex and 10 (Ndufb10: 15589-1-AP) and ribosomal protein L17 (Rpl17: 14121-1-AP) were obtained from ProteinTech (Chicago, IL, USA). The primary antibody specific for Finkel–Biskis–Reilly murine sarcoma virus (FBR-MuSV) ubiquitously expressed (fox derived) (Fau: NBP1-55090) was obtained from Novus Biologicals (Littleton, CO, USA). Primary antibodies specific for G protein-coupled receptor kinase-interactor 2 (Git2: A302-102A) and structural maintenance of chromosomes protein 5 (Smc5: A300-236A) were obtained from Bethyl Laboratories Inc. (Montgomery, TX, USA).

### Differential Detergent Cellular Fractionation

For cellular differential detergent fractionation, cell lysates were prepared using the Qproteome^®^ cell compartment extraction kit (cat# 37502) according to the manufacturer’s instructions (Qiagen, Valencia, CA, USA) with minor modifications. In brief, cultured cells were first washed in ice-cold phosphate buffered saline (PBS: Sigma-Aldrich, St. Louis, MO, USA) and then collected from the plate using ice-cold calcium-free cell dissociation solution (Sigma-Aldrich, St. Louis, MO, USA). Intact cell pellets were created by centrifugation at 1000 × *g* at 4°C. Cell pellets were then solubilized in the pre-cooled Qproteome proprietary cytoplasmic protein extraction buffer (CE1: 500 μl), which was supplemented with 1× of the kit Protease Inhibitor Solution. The lysates were then tumbled in the cytoplasmic CE1 buffer for 10 min at 4°C. The CE1 lysate was then centrifuged at 1000 × *g* for 10 min at 4°C. The supernatant was then removed to a fresh Eppendorf tube to generate the cytoplasmic tissue extract. The extant cell pellet was then solubilized in 500 μl of ice-cold CE2 plasma membrane extraction buffer supplemented with 1× of the kit Protease Inhibitor Solution and then tumbled at 4°C for 30 min. The lysate was then clarified by centrifugation at 6000 × *g* for 10 min at 4°C. The supernatant, representing the plasma membrane enriched compartment was removed to a fresh Eppendorf tube, and the extant pellet was then incubated with 7 μl of Qproteome-supplied Benzonase^®^ Nuclease and 13 μl of distilled water, mixed through gentle agitation and then incubated at standard room temperature (21–24°C) for 15 min. The pellet was then solubilized, with additional pipette trituration, in 250 μl of ice-cold Qproteome CE3 nuclear/large organelle compartment extraction buffer supplemented with 1× of the kit Protease Inhibitor Solution before being tumbled for 10 min at 4°C. The lysate was next clarified by centrifugation at 6800 × *g* for 10 min at 4°C. The supernatant, representing the nuclear/large organelle cellular fraction, was removed to a fresh Eppendorf tube. The extant cell pellet was then solubilized in 250 μl of room temperature Qproteome CE4 cytoskeletal fraction extraction buffer.

### Glucose Uptake Assays

For specific glucose uptake assessment, cultured cell monolayers were glucose- and serum-deprived in Krebs Ringer buffer (25 mM NaCl, 5 mM KCl, 1.25 mM NaH_2_PO_4_, 2 mM CaCl_2_, 1 mM MgCl_2_, 25 mM NaHCO_3_) at 37°C and 5% CO_2_ for 40 min on the day of experimentation. To determine glucose uptake rates, the specified glucose bolus dose was added to the Krebs Ringer buffer, and 30 μL aliquots were taken at 0, 1, 5, 10, 15 and 30 min. Glucose concentration from each aliquot was determined using a fluorescent glucose assay kit (# K606-100: BioVision, Milpitas, CA, USA) as per manufacturer’s instructions. Cells were lysed at the end of the experiment, and protein concentration was determined through a BCA assay (ThermoFisher Scientific, Waltham, MA, USA). Glucose uptake curves were then normalized for each experiment against the respective total protein concentration measured.

### RNA Extraction and Oligonucleotide Microarray Hybridization

RNA isolation from three animals in each experimental group was carried out using the Qiagen RNeasy Mini Kit (Qiagen, Inc., Valencia, CA, USA), as described previously (68). RNA conversion to cDNA and subsequent hybridization with Sentrix MouseRef-8 Expression BeadChips (Illumina, San Diego, CA, USA) was performed as described previously (19, 69). Microarray data were analyzed using DIANE 6.0^9^, a spreadsheet-based microarray analysis program based on the SAS JMP7.0 system^10^. Raw microarray data were subjected to filtering and *z*-normalization and tested for significant changes as described previously (19). Initial filtering identified genes with a *z*-ratio of ≥ ±1.50, with the *z*-ratio being derived from the difference between the averages of the observed gene *z*-scores divided by the SD of all of the differences for that particular comparison. Genes were then refined by calculating the FDR, which controls for the expected proportion of falsely rejected hypotheses, and including only those genes with FDR <0.05. These data were further analyzed using analysis of variance with significance set at *p* < 0.05. This allowed us to identify transcripts that differed in their intensity across all of the animal replicates and the various experimental conditions of the mice employed in this study.

### Statistical Analyses

In each histogram or figure, data represent the means ± SEM. Statistical analyses (Student’s *t-*test) were performed using GraphPad Prism (GraphPad Software, San Diego, CA, USA). *p* ≤ 0.05 was considered statistically significant. Significance is indicated in each figure as *p ≤ 0.05; **p ≤ 0.01; ***p ≤ 0.001.

## Results

### Loss of GIT2 Protein Affects Whole-Body Metabolic Activity Independent of Physical Motor Activity

**Figure 1:**
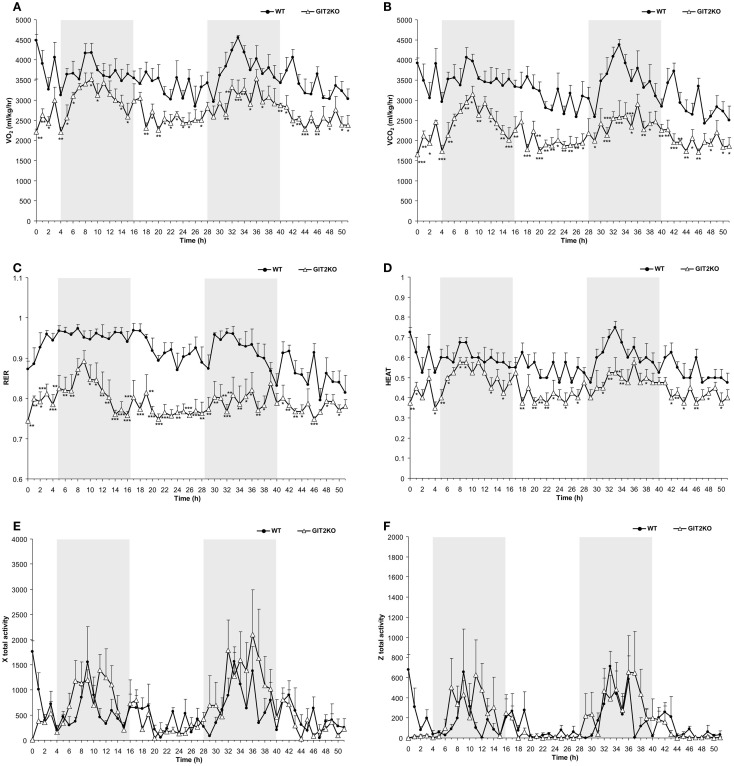

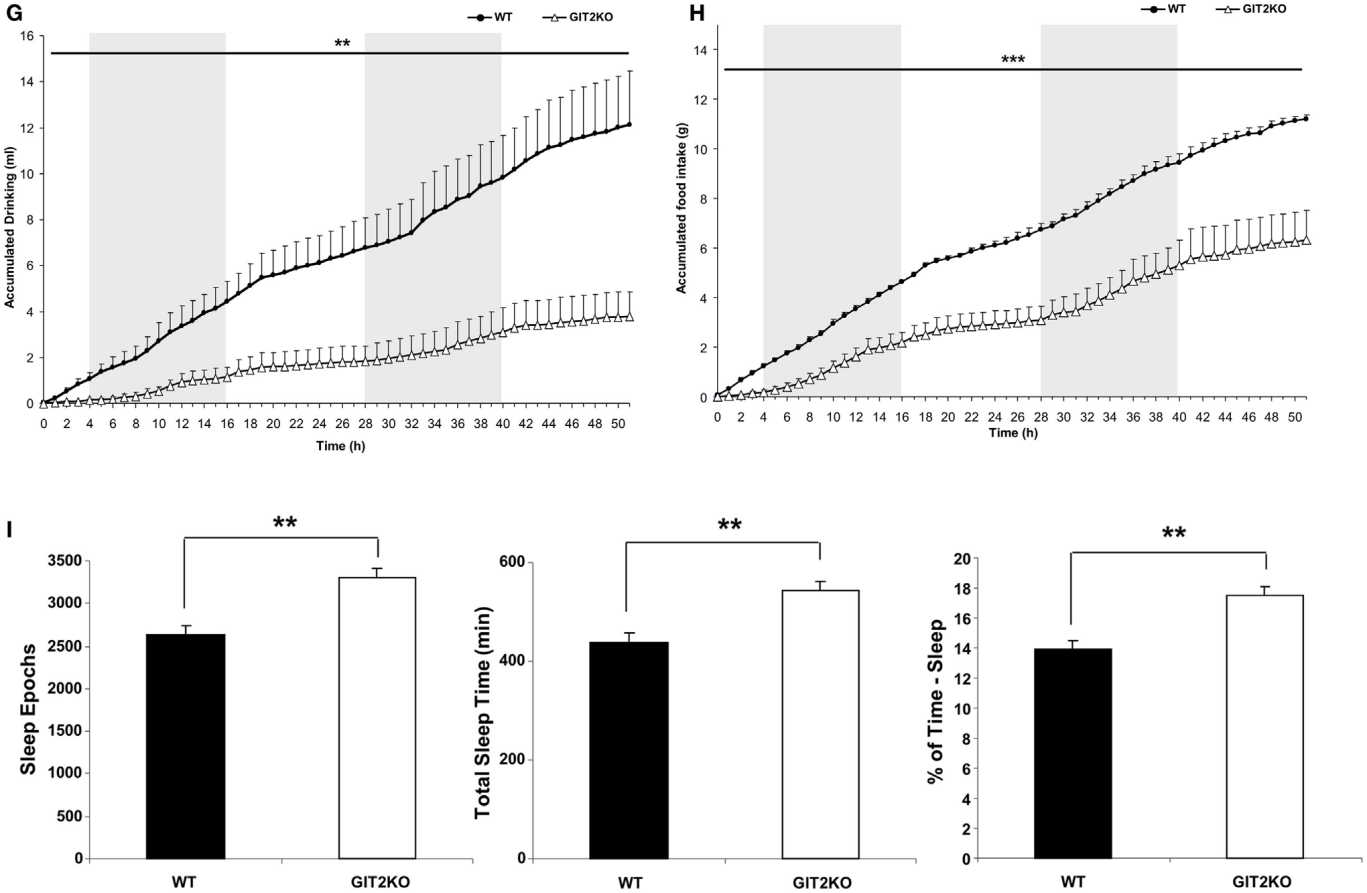
**Whole-body metabolic analysis of WT and GIT2KO mice**. Male WT or GIT2KO mice were housed for 48 h in a Columbus Instruments International Comprehensive Lab Animal Monitoring System (CLAMS). VO_2_ **(A)**, VCO_2_ **(B)**, RER (respiratory exchange ratio) **(C)**, HEAT (ambient heat generation) measurements for WT and GIT2KO mice **(D)**. Locomotor physical movement × total activity **(E)** and *z* total activity **(F)** measurements were made for WT and GIT2KO mice. Accumulated drinking **(G)** and accumulated food intake **(H)** and sleep epochs, total time sleeping, and % of time asleep **(I)** measurements were also recorded. For **(A–H)**, WT datapoints are represented by black circles; GIT2KO datapoints are represented by open triangles. Gray areas in each panel **(A–H)** indicate the dark period of animal incubation. For **(I)**, WT data is indicated by black bars and GIT2KO data by white bars. Statistical significance is indicated in each figure as *p ≤ 0.05; **p ≤ 0.01; ***p ≤ 0.001.

As metabolic activity has been strongly linked with aging and multiple disease processes, we first investigated how genetic deletion of the aging-associated factor, GIT2, affected somatic metabolism and activity in post-pubertal 4-month-old (m.o.) male GIT2KO mice compared to age- and gender-matched control WT mice (Figure 1). Compared to WT mice, over the 48-h CLAMS-assessment period, we found that male GIT2KO mice demonstrated significantly lower levels of VO_2_, VCO_2_, and consequently RER (Respiratory Exchange Ratio) (Figures 1A–C). In addition, we also found that GIT2KO mice demonstrated a significantly lower thermal output temperature, compared to WT controls (Figure 1D). In contrast to the significant alterations in the aforementioned metabolic parameters, no significant differences in physical motor activity (in *x* total or *z* axis) across the test period between WT and GIT2KO mice (Figures 1E,F) were observed, indicating that the metabolic distinctions were not due to alterations in physical motor activity. In addition to the altered metabolic parameters present in the GIT2KO mice, a significant reduction in both food and water intake was observed compared to WT controls (Figures 1G,H). GIT2KO mice also demonstrated an increased propensity for longer sleep/inactivity epochs compared to WT mice (Figure 1I).

### Transcriptomic Signatures of Metabolic Disruption in GIT2KO Hypothalami

**Figure 2:**
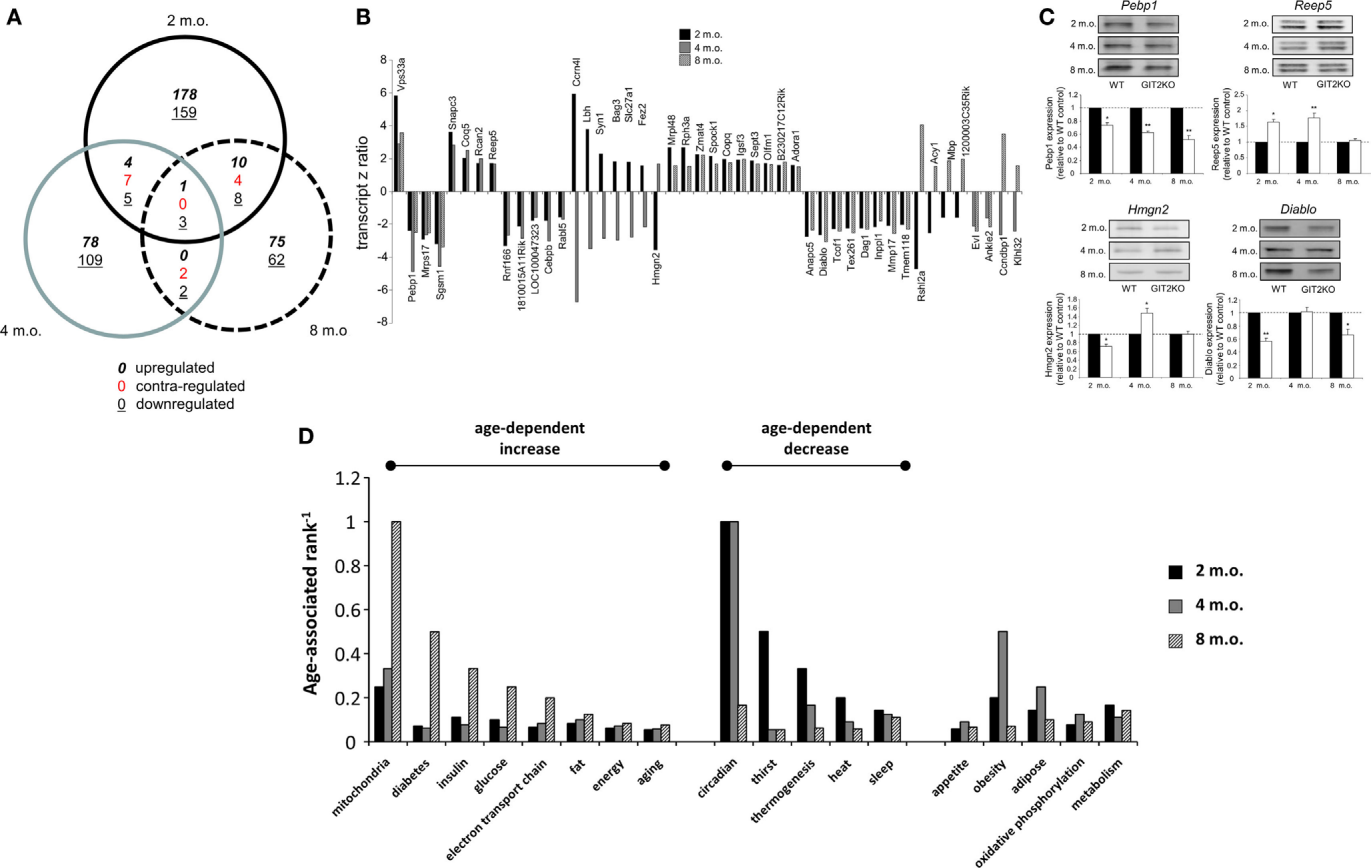
**Hypothalamic transcriptomic signatures in WT and GIT2KO mice across early life span timepoints**. **(A)** Differential VennPlex analysis of hypothalamic transcriptomic data from 2-, 4-, and 8-month-old (m.o.) GIT2KO mice compared to age-matched WT controls. Contra-regulation denotes transcripts common to at least two Venn sectors that possess a different expression polarity. **(B)** Transcript *z*-ratios of significantly regulated transcripts in GIT2KO mice (compared to WT controls) common to at least two age timepoints. **(C)** Western blot validation of selected differentially regulated gene transcripts. WT data is indicated by black histogram bars: white histogram bars denote GIT2KO data. **(D)** Latent semantic indexing-based age-dependent ranking of biological functions related to GIT2KO physical and molecular phenotypes. Statistical significance is indicated in each figure as *p ≤ 0.05; **p ≤ 0.01.

Our whole-body metabolic analyses of the GIT2KO mice demonstrated a strong alteration in metabolic activity. We have previously shown that aberrant hypothalamic signaling activity (13) as well as transcriptomic profiles (69) are strongly linked to both pathological aging as well as the presentation of neurodegenerative disease. We therefore assessed, using microarray, the effects of GIT2 genomic deletion on hypothalamic transcriptomic profiles in male mice at 2, 4, and 8 months of age as these timepoints encompass our initial CLAMS 4-m.o. timepoint data. This timeframe was chosen to represent post-pubertal early life periods in which overt age-related damage may not be present but signatures of pro-aging behavior may be observed (42). Gene transcripts differentially and significantly (*p* < 0.05) regulated in GIT2KO hypothalami compared to age-matched WT mice across the three time points were investigated using VennPlex (57) (Figure 2A). Surprisingly, we found that transcriptomic profiles [Tables S1 in Supplementary Material (2 m.o.), S2 (4 m.o.) and S3 (8 m.o.)] were strongly age-specific, with a considerable percentage of transcripts being uniquely regulated at a single time-point (2 m.o. – 88.9% unique; 4 m.o. – 88.6% unique; 8 m.o. – 82.03% unique). Across the three experimental ages, we found 46 transcripts common to at least two timepoints (Figure 2B) – we randomly selected four of these transcripts (Pebp1, Reep5, Hmgn2, Diablo) to validate, using Western blotting (Figure 2C). Our Western blot analysis of hypothalamic tissues replicated our microarray-derived transcriptomic regulation patterns in the GIT2KO mice. The functional effects of the group of 46 GIT2KO-modulatated transcripts identified across all timepoints were then investigated using unbiased Textrous!-mediated transcript-word association (59, 62, 70). Using the collective processing module of Textrous! to generate a hierarchical word cloud, a strong focus upon neurotransmission and metabolism was evident (Figure S1 in Supplementary Material). With extraction of the primary word list associated with the 46 input transcripts (Table S4 in Supplementary Material), multiple words associated with glucose regulation (“*insulin*,” “*proinsulin*,” “*euglycemic,”* “*glucose*,” “*intolerance*,” “*incretin*,” “*insulin-responsive*,” “*c-peptide”*) were strongly evident. A typical healthy animal using glucose as its primary source of energy demonstrates an RER of approximately 1 (observed in the WT mice: Figure 1C), the GIT2KO mice however possessed an RER of approximately 0.8 across the testing period (Figure 1C), which is suggestive of loss of glucose catabolism in favor of lipid metabolism. In line with this a strong representation of terms linked with lipid metabolism were also found in the Textrous! output form the 46 core GIT2KO transcripts (Figure S1 in Supplementary Material; Table S4 in Supplementary Material), e.g., “*adipocytes*,” “*lipolysis*,” “*fatty*,” and “*triacylglycerol*.”

### Early Aging Pathological Trajectories in GIT2KO Mice

With the combination of our whole-animal metabolic/behavioral analyses and our transcriptomic profiling of the GIT2KO hypothalamic, a strong metabolism-related pathological signature was evident. Using an analogous informatics process to Textrous!, i.e., GeneIndexer (Computable Genomix Inc.), that applies LSI to extract transcript-word associations in the reverse direction (19, 62), i.e., from input text term to correlated transcript, we investigated how pathological metabolic trajectories may be evident in the GIT2KO mice even at these early life 2, 4, and 8 month timepoints. Therefore, we created lists of implicitly associated transcripts, across the three timepoints, linked to functional and behavioral aspects we observed in the GIT2KO mice, denoted by our input GeneIndexer interrogator terms (Table S5 in Supplementary Material; Table S6 in Supplementary Material – 2 m.o.; Table S7 in Supplementary Material – 4 m.o.; Table S8 in Supplementary Material – 8 m.o.). To assess the relative importance, using the hypothalamic transcriptomic datasets (2, 4, and 8 m.o.), of these terms at the specific experimental time points, the mean cosine similarity scores for the implicitly associated transcripts for each interrogator term were calculated. Using these mean cosine similarity score magnitudes, we ranked (from term 1 to term 18) the terms at each age timepoint – for presentation purposes we reciprocally transformed these ranks (Table S9 in Supplementary Material). Using this novel informatics approach, it is clear that multiple metabolic aspects (“*mitochondria,” “diabetes,” “insulin,” “glucose,” “electron transfer chain,” “fat,” “energy,” “aging”*) demonstrate, at these early life timepoints, a coherent age-dependent increase (Figure 2D). Interestingly, many of the behavioral factors we observed (Figure 1) that were disrupted (“*thirst,” “thermogenesis,” “heat,” “sleep”*) demonstrated an opposite age-dependent ranking mechanism (Figure 2D).

### Multidimensional Signaling Network Alteration of Metabolic and Glycemic Activity in GIT2KO Mice

**Figure 3:**
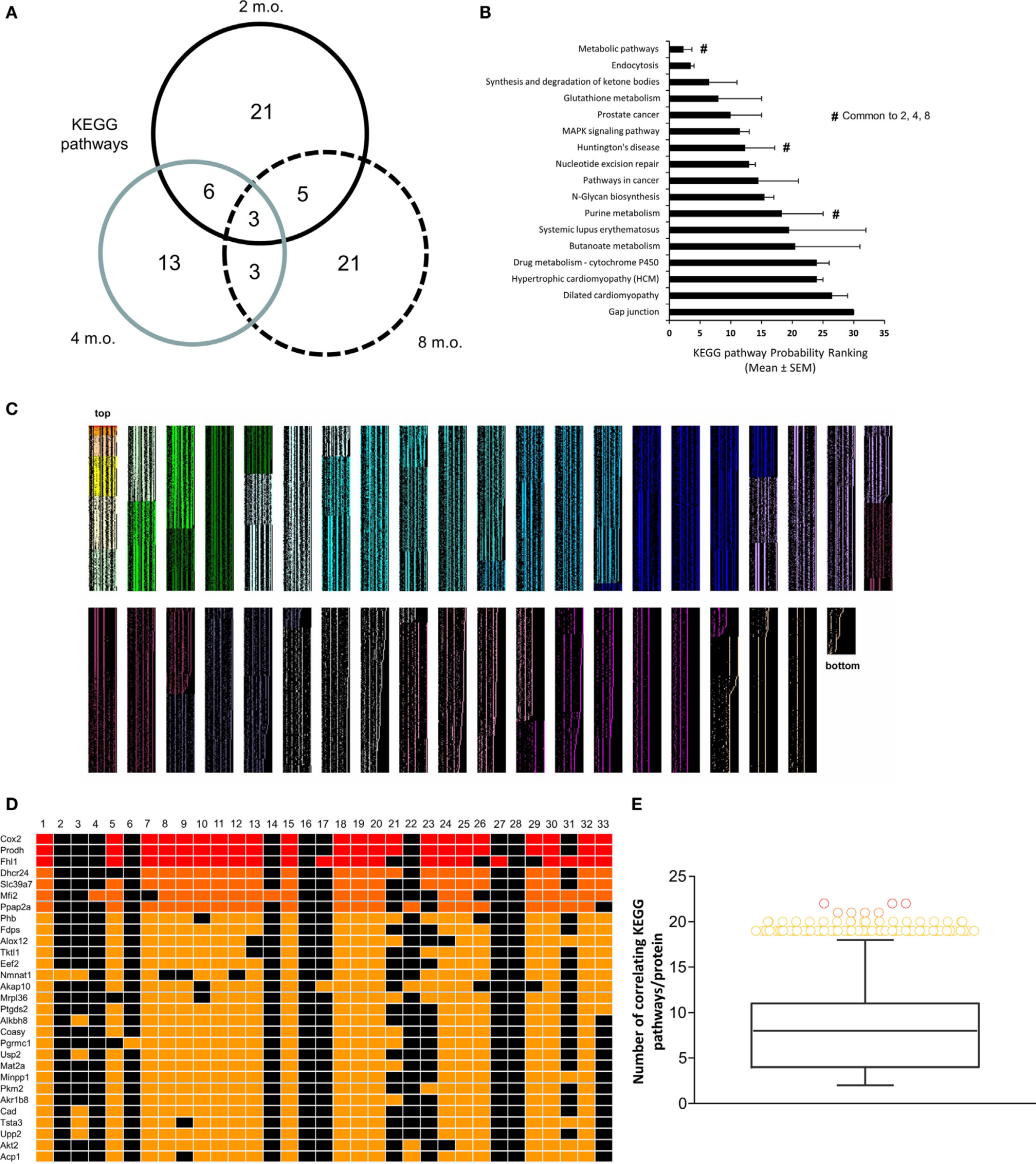
**KEGG signaling pathway-based keystone analysis of GIT2KO hypothalamic signatures**. **(A)** Differential VennPlex analysis of hypothalamic KEGG signaling pathway data generated with the primary microarray transcriptomic data from 2-, 4-, and 8-month-old (m.o.) GIT2KO mice compared to age-matched WT controls. **(B)** Probability ranking scores for KEGG signaling pathways common to at least two different experimental age timepoints. ^#^denotes KEGG pathways significantly populated by transcriptomic data from all three experimental timepoints. **(C)** KEGG pathway-generated cosine similarity score matrix generated using latent semantic output from GeneIndexer. Thirty two separate columns representing each significantly regulated KEGG pathway from the 8-m.o. hypothalamic microarray data were used to create the matrix of implicitly linked transcripts. Only transcripts demonstrating a correlation to at least two KEGG pathways were considered for analysis. Each level of transcript association (from 2 to 32 KEGG pathways) is indicated by a selective heatmap color (matrix size = 0.67 × 10^6^ cells: top and bottom of matrix are labeled). **(D)** Expanded inset of top 30 most highly correlating matrix transcripts across the 32 KEGG pathways. **(E)** Class-based identification of the 92 transcripts within the 99% percentile of cross-pathway correlation probability.

**Figure 4:**
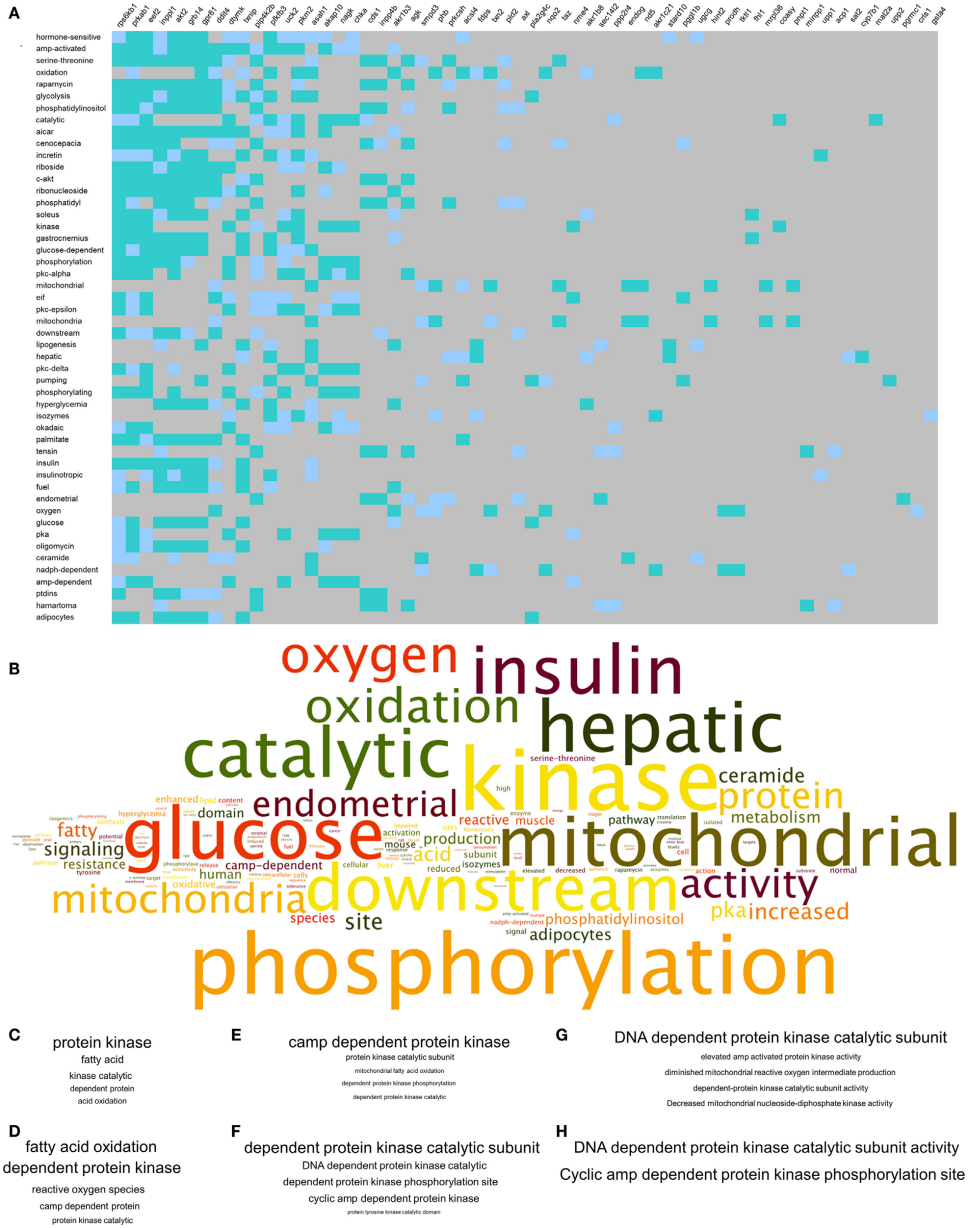
**Natural language processing-based analysis of multidimensional GIT2KO molecular pathway signatures**. **(A)** Textrous!-based individual processing heatmap processing of the core 92 transcripts represented in Figure 3 comprising the multidimensional GIT2KO pathway signature. The strength of noun-transcript correlation is indicated by the intensity of teal color of the block. **(B)** Higher-order word cloud representation of expanded noun and noun-phrase output from the individual processing analysis in **(A)**. The size of the resultant noun is indicative of the frequency in the output. **(C–H)** Textual phrase analysis (WriteWords*) of the higher-order word cloud data using length-dependent [**(C)** – two words, **(D)** – three words, **(E)** – four words, **(F)** – five words, **(G)** – six words, **(H)** – seven words] sentence analysis. Within each panel **(C–H)** the relative phrase frequencies is indicated relatively by the phrase text size. *http://www.writewords.org.uk/

To complement our novel transcriptomic data investigation using LSI-based techniques, we also performed unbiased KEGG signaling pathway enrichment analysis on the 2-, 4-, and 8-m.o. transcriptomic datasets (Table S10 in Supplementary Material – 2 m.o.; S11 – 4 m.o.; S12 – 8 m.o.). Similar to our basic transcriptomic analyses, we found that the predicted KEGG signaling pathway activity was age-specific, but to a much lesser extent than the primary transcripts, i.e., 2 m.o. 60% of pathways were unique; 4 m.o. 52% of pathways were unique and for 8 m.o. Sixty-five percent of pathways were unique (Figure 3A). In line with our CLAMS whole-body analysis and LSI-mediated transcriptomic investigation, the KEGG pathways demonstrating a link across the three timepoints (i.e., present in at least two of the age timepoints: 17 pathways) possessed a strong focus upon energy metabolism (“*Metabolic Pathways,” “Purine metabolism,” “Synthesis and degradation of ketone bodies,”* “*Glutathione metabolism”*) as well as upon signaling functions associated with GIT2, such as cellular trafficking (“*Endocytosis”*) and DNA damage/repair (“*Nucleotide excision repair”*) (Figure 3B). Three pathways were common to all three animal ages: “*Metabolic Pathways*,” “*Purine metabolism,”* and “*Huntington’s disease.”* We have previously demonstrated that Huntington’s disease is often associated, in patients and especially in transgenic murine models of the disease, with considerable glycemic and metabolic dysfunction linked to pancreatic morphology alteration and diabetic pathological phenotypes (53, 54, 71, 72). Therefore, this signaling pathway data reinforces the potential for GIT2 deletion to induce a systemic glycemic/metabolic disruption linked to central and peripheral degenerative actions. With respect to this pathological link, we chose to further investigate the high-dimensionality molecular metabolic phenotype of the GIT2KO mice using our KEGG data from the 8 m.o. timepoint as this age demonstrated the greatest linkages to glycemic/metabolic alterations (Figure 2D). Using our previously demonstrated KEGG signaling pathway-based keystone discovery workflow (13), we created an LSI-based pathway-transcript matrix (0.67 × 10^6^ total cell size: Figure 3C; Table S13 in Supplementary Material). From this matrix, we identified the 99% class percentile most cross-KEGG pathway conserved gene transcripts (92 in total: indicated in *italic* – Table S13 in Supplementary Material, Figure 3D-expanded view of top of heatmap in Figure 3C, Figure 3E-color-coded 99% percentile transcript identification). For illustration purposes, the top 30 cross-KEGG pathway correlated transcripts are indicated (Figures 3D,E). Many of the 92 transcripts identified in the 99% percentile of cross-KEGG pathway group are critically involved in metabolic activities linked to diabetes-related pathophysiologies, e.g., Cox2 [cytochrome c oxidase subunit II (73)], Coasy [CoA synthase (74)], and Akt2 (75). Textrous!-based interpretation of this 92 “keystone” transcript dataset, using the individual processing mode [Figure 4A (59)], revealed a strong link to glycemic regulation (“*glucose-dependent*,” “*insulin,” “glycolysis,” “amp-activated”*) and insulin functionality (“*incretin,” “hyperglycemia,” “insulinotropic”*). Using the ability of Textrous! to extract scientifically relevant noun-phrases linked to the input transcripts (62), we created a “higher-order” word cloud using Wordle^11^ from these semantically associated nouns and noun-phrases (3773 input words in total: Figure 4B). This word cloud again underscored the importance of insulin-related glycemic regulation in GIT2KO mice compared to WT controls. Using the web-based phrase frequency counter suite of WriteWords^12^, we were able to extract from the input nouns and noun-phrases, the most common phrases of various word lengths (Figures 4C–H). Using this unbiased dataset description technique, we found sentences describing “DNA damage repair,” “fatty acid oxidation,” “mitochondrial activity,” and “ROS” were prominent – thus again confirming a functional intersection between metabolic actions and GIT2-related functionality [e.g., DNA repair and ROS responsivity (18, 19)]. As we have consistently found a strong correlation between GIT2 genomic deletion and global metabolism disruption (Figure 1), predicted insulin/glucose system disruption (Figures 2–4) and potential pancreatic pathology (Figure 2), we next assessed whether GIT2 genomic deletion affected the functional morphology of the murine pancreas.

### Genomic Deletion of GIT2 Disrupts Pancreatic Morphology

**Figure 5:**
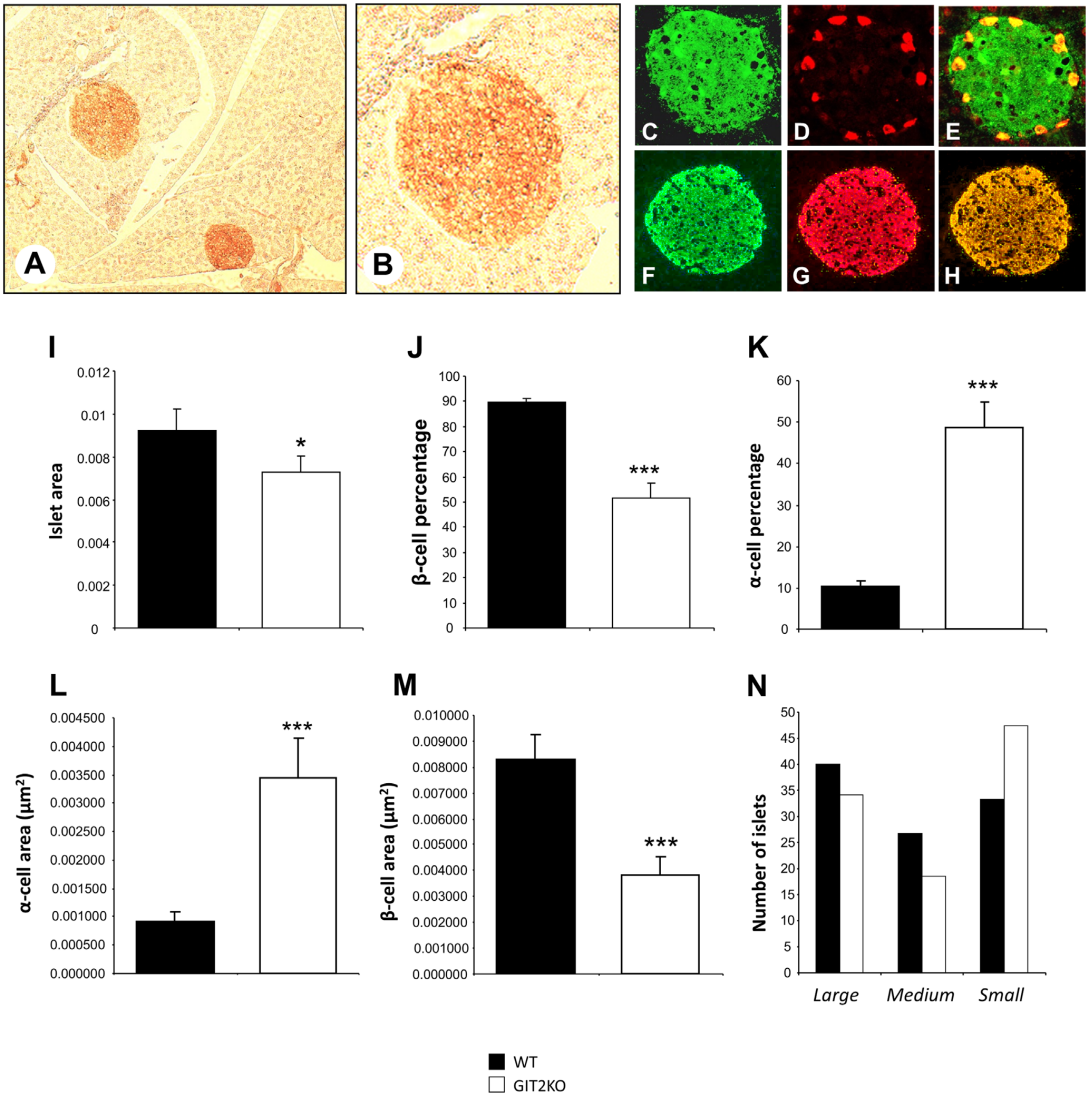

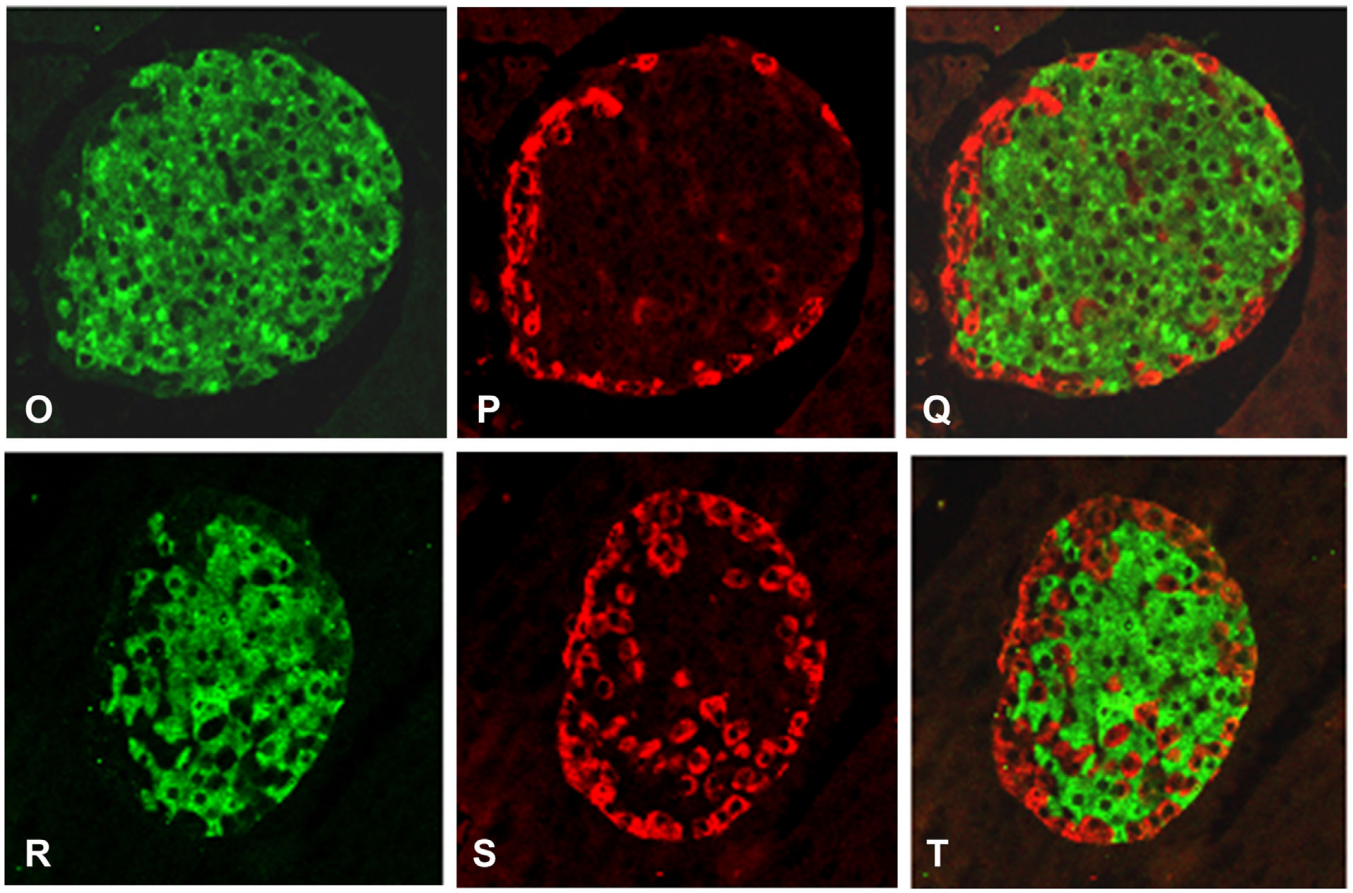
**Genomic GIT2 deletion affects pancreatic islet functional structure**. **(A)** DAB- (3,3′-diaminobenzidine) based immunohistochemical identification of GIT2-associated reactivity in discrete islet-like structures in WT murine pancreas. **(B)** High-magnification inset of DAB-stained, GIT2-immunoreactive pancreatic islet-like structure. **(C)** GIT2 AF488 immunostaining of WT pancreatic islet. **(D)** Glucagon AF568 immunostaining of WT pancreatic islet. **(E)** Merge of panels **(C,D)** indicating AF488–AF568 colocalization. **(F)** Insulin AF488 immunostaining of WT pancreatic islet. **(G)** GIT2 AF568 immunostaining of WT pancreatic islet. **(H)** Merge of panels **(F,G)** indicating AF488–AF568 colocalization. MATLAB-based pancreatic islet mathematical automated assessment of WT (black bars) or GIT2KO (white bars) pancreas islet area **(I)**, β-cell percentage **(J)**, α-cell percentage **(K)**, α-cell area **(L)**, β-cell area **(M)** and islet number/size distribution **(N)**. **(O)** Insulin AF488, glucagon **(P)** immunostaining of WT islet [8 month old (m.o.)]. **(Q)** Merge of panels **(O,P)**. **(R)** Insulin AF488, glucagon **(S)** immunostaining of GIT2KO islet (8 m.o.). **(T)** Merge of panels **(R,S)**. Statistical significance is indicated in a specific panel as *p ≤ 0.05; **p ≤ 0.01; ***p ≤ 0.001.

Histochemical analysis of isolated pancreatic islets from control WT mice demonstrated a strong reactivity with GIT2 antibodies in pancreatic islet-like structures (Figure 5A, inset B). With differential histochemical staining for GIT2, insulin (for beta cells), or glucagon (for alpha cells), it was evident that GIT2 was present in both alpha and beta cells of the pancreatic islets (GIT2: Figures 5C–E, glucagon: Figures 5F–H). Using our previously described islet analytical workflow (55), we found that 8-m.o. GIT2KO mice demonstrated a significantly reduced islet area (Figure 5I) and beta cell percentage (Figure 5J) with a concomitant significant increase in alpha cell percentage (Figure 5K). Linked to these data, we also found a significant increase in the total alpha cell area (Figure 5L), again with an expected reduction in total beta cell area (Figure 5M). Using our islet size differentiation parameters, we also found a general increase in the numbers of smaller islets in GIT2KO compared to WT controls (Figure 5N). Performing similar analysis on 2- and 4-m.o. GIT2KO mice, we found that while differences in pancreatic morphology compared to WT mice were evident, none however were significant (Figure S2 in Supplementary Material). Using visual morphological inspection of the 8-m.o. GIT2KO islets, it was clear that along with the decrease in beta cells and increase in alpha cells there was also an evident alpha cell involution into the beta cell mass – a facet indicative of diabetic pathologies in murine paradigms [WT mice: Figures 5O–Q, GIT2KO mice: Figures 5R–T (76, 77)]. This involution was generally absent in both 2-and 4-m.o. GIT2KO mice (Figure S3).

### Genomic Deletion of GIT2 Disrupts Plasma Membrane Metabolic Hormone Levels and Pancreatic Functionality

**Figure 6:**
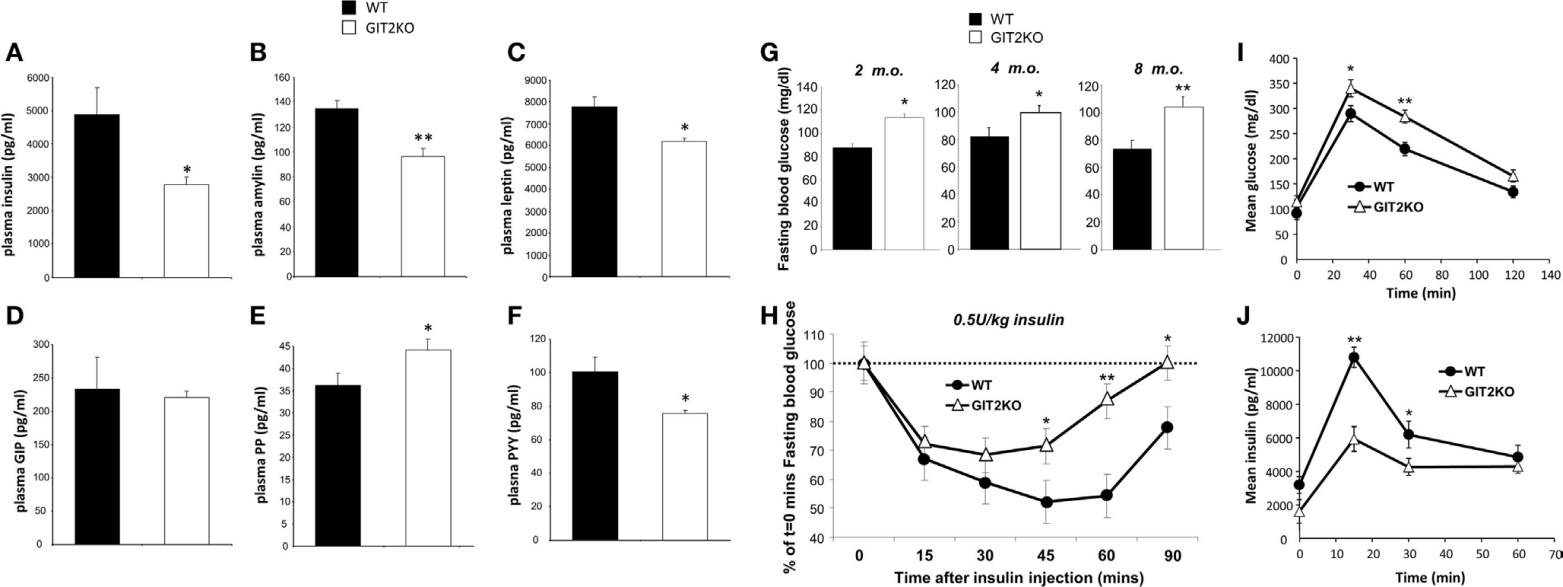
**GIT2 genomic deletion affects multiple circulating energy-regulatory factors and response to external glycemic challenges**. Differential circulating plasma levels of insulin **(A)**, amylin **(B)**, leptin **(C)**, GIP [gastric inhibitory peptide: **(D)**], PP [pancreatic polypeptide: **(E)**], and PYY [peptide YY: **(F)**] between 8-m.o. (month old) WT (black bars) and GIT2KO (white bars) mice. **(G)** Fasting blood glucose measurements for 2-, 4-, 8-m.o. WT (black bars) or GIT2KO (white bars) mice. For **(H–J)** WT or GIT2KO mouse data is represented by black circles or open triangles respectively. **(H)** Standard bolus insulin tolerance test (ITT: 0.5U/kg insulin) performed on 8-m.o. WT or GIT2KO mice. **(I)** Time-dependent elevation of plasma glucose in 8-m.o. WT or GIT2KO mice after intragastric introduction of a glucose bolus (2g/kg). **(J)** Time-dependent elevation of plasma insulin in 8-m.o. WT or GIT2KO mice after intragastric introduction of a glucose bolus (2 g/kg). For each experimental panel, statistical significance is indicated in each figure as *p ≤ 0.05; **p ≤ 0.01; ***p ≤ 0.001.

To further investigate the systemic metabolic effects of genomic GIT2 deletion, we measured the levels of multiple metabolic hormones [insulin, amylin, leptin, GIP (gastric inhibitory polypeptide), PP (pancreatic polypeptide), PYY (peptide YY)] in 8-m.o. GIT2KO mice compared to WT controls (Figures 6A–F). Deletion of GIT2 resulted, at the 8-m.o. timepoint, in significant reductions in plasma insulin, amylin, and PYY along with a significant increase in the PP levels. Significant age-dependent reductions of insulin were observed at the 2- and 4-m.o. timepoints (Figure S4): trends for non-significant reductions in both amylin and leptin where observed (Figure S4) as were non-significant increases in plasma GIP (Figure S4). Consistent with the multiple molecular glycemic pathologies observed in the GIT2KO mice, we found that as early as 4 months of age (and continuing onto 8 m.o.), a significantly higher fasting plasma glucose was evident compared to age-matched WT controls (Figure 6G). We investigated whether this elevated plasma glucose was associated with a reduction in the insulinotropic functionality of GIT2KO mice. Using a standard ITT approach, we found that the 8-m.o. GIT2KO demonstrated a significant degree of insulin resistance to a bolus dose of Lantus^®^ (Figure 6H). Therefore, along with the disruption of beta cell mass, alpha cell involution, and reduced plasma insulin levels the GIT2KO mice also demonstrate significant insulin resistance. Performing standardized OGTT analysis, we also found that GIT2KO mice (at 8 m.o.) demonstrated a significantly lower ability to uptake a glucose bolus (Figure 6I). Coordinating with this reduced glucose uptake ability, we found that GIT2KO mice possessed a significantly diminished capacity to functionally secrete insulin in response to the glucose bolus (Figure 6J).

### High-Dimensionality Transcriptomic Profiling of GIT2KO Pancreatic Islets

**Figure 7:**
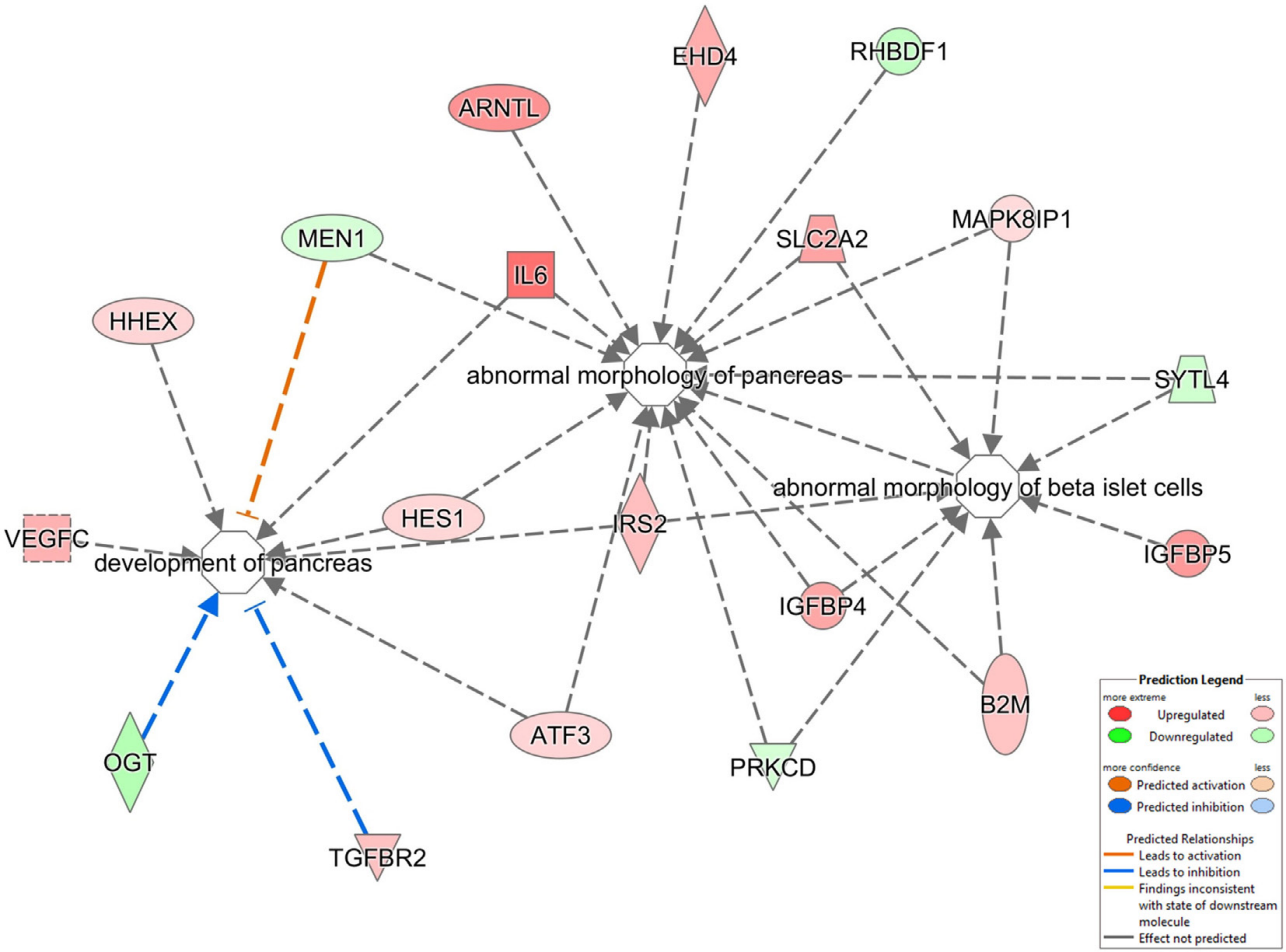
**Disease Bio-Function analysis of pancreatic islet transcriptomic data**. Significant transcriptomic data from isolated GIT2KO pancreatic islets was functionally annotated using the Disease/Bio-Function suite from IPA Pathways Analysis. The regulation of three processes vital for pancreatic function is indicated: “*development of pancreas, ‘abnormal morphology of pancreas,’ abnormal morphology of beta islet cells.”* The nature of the specific node components and their type of functional interaction (noted as arrows between factors) are described in the associated key. Red factors were upregulated in the input dataset while green factors were downregulated (GIT2KO versus WT).

**Figure 8:**
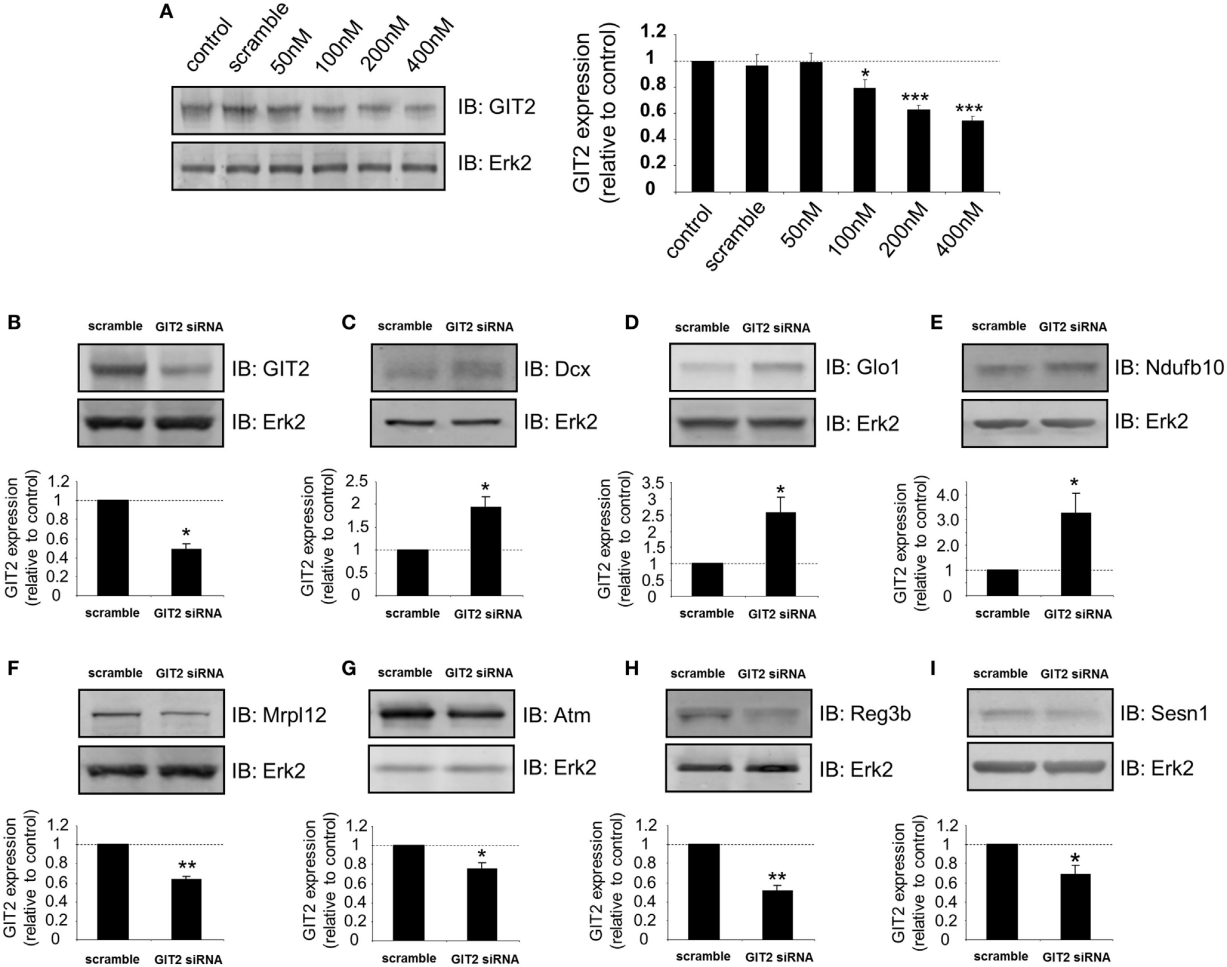
**Depletion of GIT2 in murine pancreatic TC-6 cells modulates the expression of multiple proteins involved in stress management and energy regulation**. **(A)** Dose-dependent GIT2 depletion curve for GIT2 siRNA. A random sequence “scramble” siRNA was employed as a negative control. Extracellular signal-regulated kinase 2 (Erk2) was employed as a loading control throughout this figure. GIT2 siRNA-mediated (using 400 nM siRNA concentration) modulation of TC-6 cells expression of GIT2 **(B)**, doublecortin [Dcx: **(C)**], glyoxylase 1 [Glo1: **(D)**], NADH dehydrogenase (ubiquinone) 1 beta subcomplex, 10 [Ndufb10: **(E)**], mitochondrial ribosomal protein L12 [Mrpl12: **(F)**], ataxia telangiectasia mutated [Atm: **(G)**], regenerating islet-derived 3 beta [Reg3b: **(H)**] and sestrin 1 [Sesn1: **(I)**]. Statistical significance is indicated in each figure as *p ≤ 0.05; **p ≤ 0.01; ***p ≤ 0.001.

From our metabolic, behavioral, informatic, and hormonal analyses, a clear disruption of pancreatic function in GIT2KO mice is evident. We chose to study this at a high-dimensionality level, using quantitative transcriptomics on mechanically isolated islets from 8-m.o. GIT2KO mice. Using purified islets from multiple GIT2KO or WT (*n* ≥ 3) mice, a differentially regulated transcriptomic profile was generated (Table S14 in Supplementary Material). At the basic transcriptomic level multiple transcripts closely linked to diabetic pathology and islet dysfunction were differentially regulated in GIT2KO compared to WT islets, e.g., Slc2a2 [glucose transporter 2 (78)], Reg3a/b [regenerating islet-derived protein 3 alpha/beta (79)], Ffa2 [free fatty acid receptor 2 (80)], Trib3 [tribbles homolog 3 (81)], PP [pancreatic polypeptide (82)], Sesn1 [sestrin 1 (83)], Glo1 [glyoxylase 1 (84)], and Lars2 [leucyl-tRNA synthetase 2, mitochondrial (85)]. Using “pancreas-specific” tissue database signaling investigation with IPA, we applied Disease/Bio-Function annotation to the GIT2KO islet transcriptome data (Table S15 in Supplementary Material). Corroborating our histochemical and functional pancreatic data, we found several prominent beta-cell focused functions (e.g., “*development of pancreas,” “abnormal morphology of pancreas,” “abnormal morphology of beta islet cells”*) within this unbiased analysis output (highlighted in Figure 7). To complement our Disease/Bio-Function annotation, we also assessed the most coherently associated functional networks within the GIT2KO transcriptomic data. The two highest scoring networks (based on numbers of enriched transcripts from the input data that form a coherent-curated network) focused on Scl2a2-Hnf4a-Hnf1a (Figure S5 in Supplementary Material) and Pdx1-Ins1-Glis3 (Figure S6 in Supplementary Material) were both officially (via IPA) denoted as involved in “*Cell Death and Survival* – *Endocrine System Disorders* – *Metabolic Disease*.” As several well-characterized murine pancreatic beta cell clonal lines exist, e.g., Beta-TC-6 (ATCC: abbreviated to “TC-6” hereafter), we decided to orthogonally investigate the link between GIT2 and several factors identified in our transcriptomic analysis using siRNA-mediated depletion of GIT2 followed by selective Western blot analysis (Figure 8), using the extracellular signal-regulated kinase 2 (Erk2) as a specific, well-characterized protein-gel loading control (86, 87). Using *in vitro* siRNA-mediated GIT2 depletion (50–400 nM siRNA: Figure 8A), we were able to significantly attenuate the expression of GIT2 in the TC-6 cells. Employing the 400 nM concentration of level of GIT2 siRNA to reduce cellular GIT2 levels, we were able to recapitulate selected expression data generated from our microarray analysis, with specific relevance to beta cell function, metabolism, diabetes, and aging (Table S15 in Supplementary Material: Figures 8B–I). Hence acute siRNA; mediated reduction of cellular GIT2 caused increased expression of Dcx [doublecortin, Figure 8C (88)], Glo1 [glyoxylase I, Figure 8D (89)], Ndufb10 [NADH dehydrogenase (ubiquinone) 1 beta subcomplex subunit 10, Figure 8E (90)) as well as a decreased expression of Mrpl12 [mitochondrial ribosomal protein L12, Figure 8F (91)], Atm [ataxia telangiectasia mutated, Figure 8G (18)], Reg3b [regenerating islet-derived 3 beta, Figure 8H (92)], and Sesn1 [sestrin 1, Figure 8I (93)].

### GIT2 Functionality in Murine Pancreatic Beta Cells

**Figure 9:**
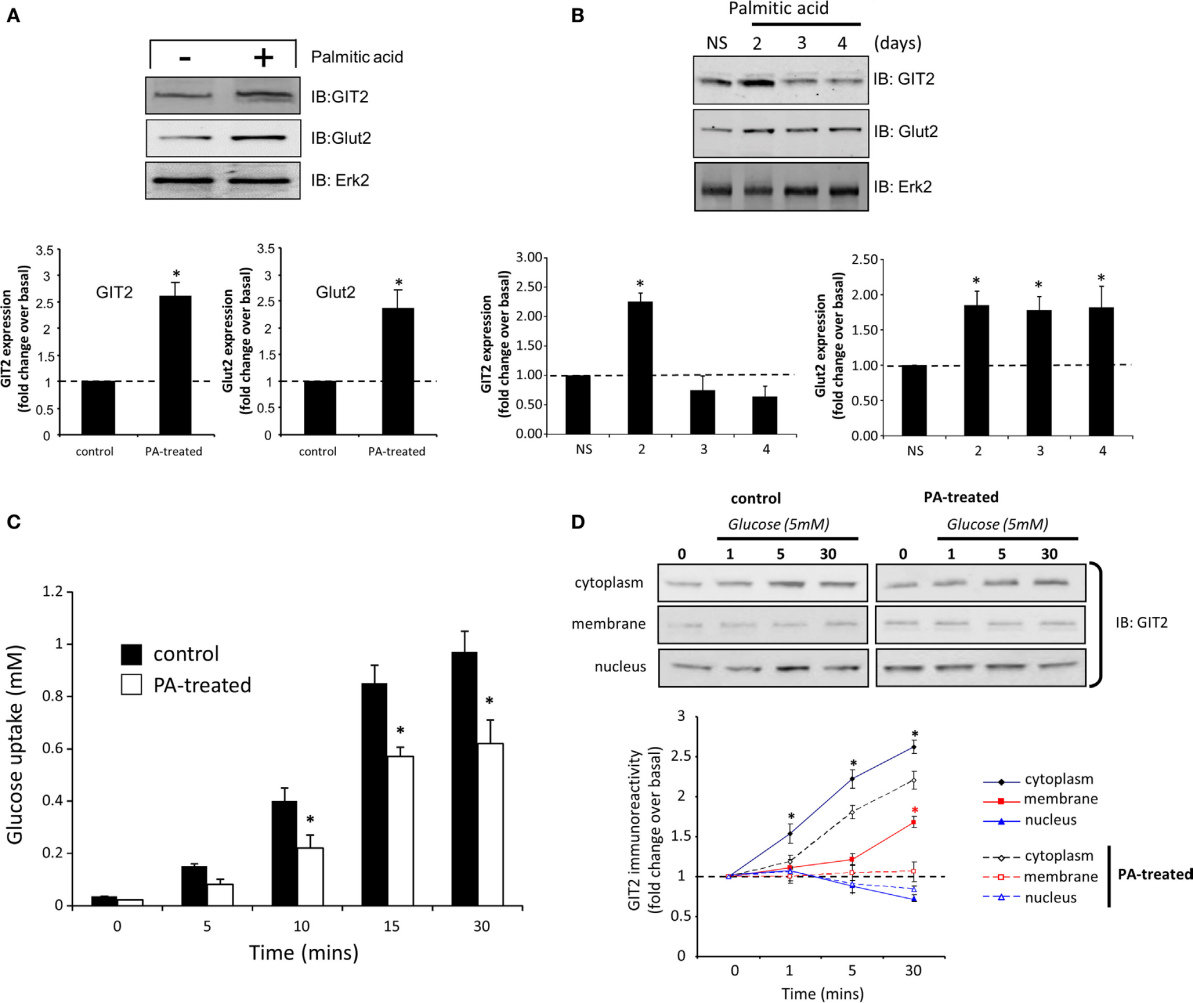
**Pathological manipulation mimicking insulin resistance in murine TC-6 cells affects GIT2 functionality and subcellular disposition**. Treatment (1 day) of TC-6 cells with palmitic acid (10 μM) and mimicking an insulin-resistant cellular status potentiates GIT2 and Glut2 expression **(A)**. Long-term palmitic acid treatment (2–4 days) differentially and biphasically regulates GIT2/Glut 2 expression **(B)**. Extracellular signal-regulated kinase 2 (Erk2) was employed as a loading control in **(A,B)**. Attenuation of bolus (5 mM) glucose uptake in TC-6 cells by long-term (4 days) palmitic acid treatment **(C)**. Palmitic acid treatment (4 days) of TC-6 cells affects glucose-driven subcellular redistribution of GIT2 across the cytoplasmic, plasma membrane, and nuclear microdomains **(D)**. Statistical significance is indicated in each figure as *p ≤ 0.05; **p ≤ 0.01; ***p ≤ 0.001.

From our transcriptomic and functional analyses, it is evident that GIT2 integrity is important in regulating insulin sensitivity and beta cell function. We next investigated the molecular nature of GIT2 activity, related to glycemic control, in beta cells using the TC-6 murine model cell line. As the GIT2KO mice demonstrate insulin resistance, we mimicked this pathophysiological state in the TC-6 cells by chronic (24 h) treatment with palmitic acid (94). In response to palmitic acid exposure, we found both increases in Glut2 as well as GIT2 (Figure 9A), here again we employed the extracellular signal-regulated kinase 2 (Erk2) as a specific, well-characterized protein gel loading control (86, 87). Extension of the chronic exposure to 4 days (10 μM palmitic acid – replenished every 6 h) demonstrated that the GIT2 expression was subsequently attenuated while the Glut2 potentiation persisted, mimicking our mouse model paradigm of GIT2KO status with an elevation of beta cell Glut2/Slc2a2 (Table S15 in Supplementary Material; Figure 9B). We assessed the viability of the TC-6 cells in response to extended palmitate treatments using Trypan Blue exclusion cell counting and failed to demonstrate any significant loss of viability compared to actinomycin D-treated (2 μg/ml, 2-h exposure) positive controls (Figure S7 in Supplementary Material). In this *in vitro* experimental system, we then assessed the ability of the palmitic acid-treated cells to uptake glucose. Performing an *in vitro* glucose uptake assay using 5 mM glucose (19), we found that cells possessing a diminished GIT2 in the presence of elevated Glut2 have an attenuation of glucose uptake capacity compared to non-treated cells (Figure 9C). Using Q-proteome^®^-based subcellular fractionation of these palmitic acid-treated cells (4 days treatment), we found that in response to the introduction of the glucose bolus, the pre-treatment of cell with palmitic acid significantly affected the glucose-induced subcellular redistribution of GIT2 (Figure 9D). Appropriate subcellular fractionation of the cellular samples with the Q-proteome^®^ system was assessed using specific western blots for marker proteins characterized by cytoplasmic (Gapdh), plasma membrane (Pmca), nuclear/large organelle (Lmna), or cytoskeletal (Tuba1a) expression (Figure S8 in Supplementary Material). In control cells, GIT2 immunoreactive signals increased in the cytoplasmic and plasma membrane fractions in response to the glucose dose. This increase, in both cytoplasmic and plasma membrane compartments, was significantly attenuated in the palmitate-treated cells. No significant dynamic changes in GIT2 immunoreactive signals were observed in the nuclear fractions, however an elevation of basal nuclear GIT2 was observed in the palmitate-treated cells (Figure 9D). This effect is likely caused by the metabolic stress of chronic palmitate treatment causing ROS damage as we have previously demonstrated that multiple cellular stressors promote GIT2 nuclear translocation to regulate the DNA damage response (18). These data therefore suggest that molecular attenuation of GIT2 affects glucose sensitivity of beta cells and cellular GIT2 redistribution is important for cellular functions such as glucose uptake. In line with these findings, we also noted that siRNA-mediated partial GIT2 depletion engendered a consistent but moderate (non-significant) attenuation of glucose uptake in TC-6 cells (Figure S9 in Supplementary Material). As GIT2 was first identified as a GPCR-associated scaffolding protein (95), its functions are typically mediated via assembling and regulating the composition of multiprotein complexes. Therefore, using selective immunoprecipitation in control, non-diabetic, and pathophysiological conditions from whole pancreas, we next investigated the nature of its physical molecular interactions in these paradigms.

### Pathological Metabolic Alteration of GIT2 Interaction Partners

**Figure 10:**
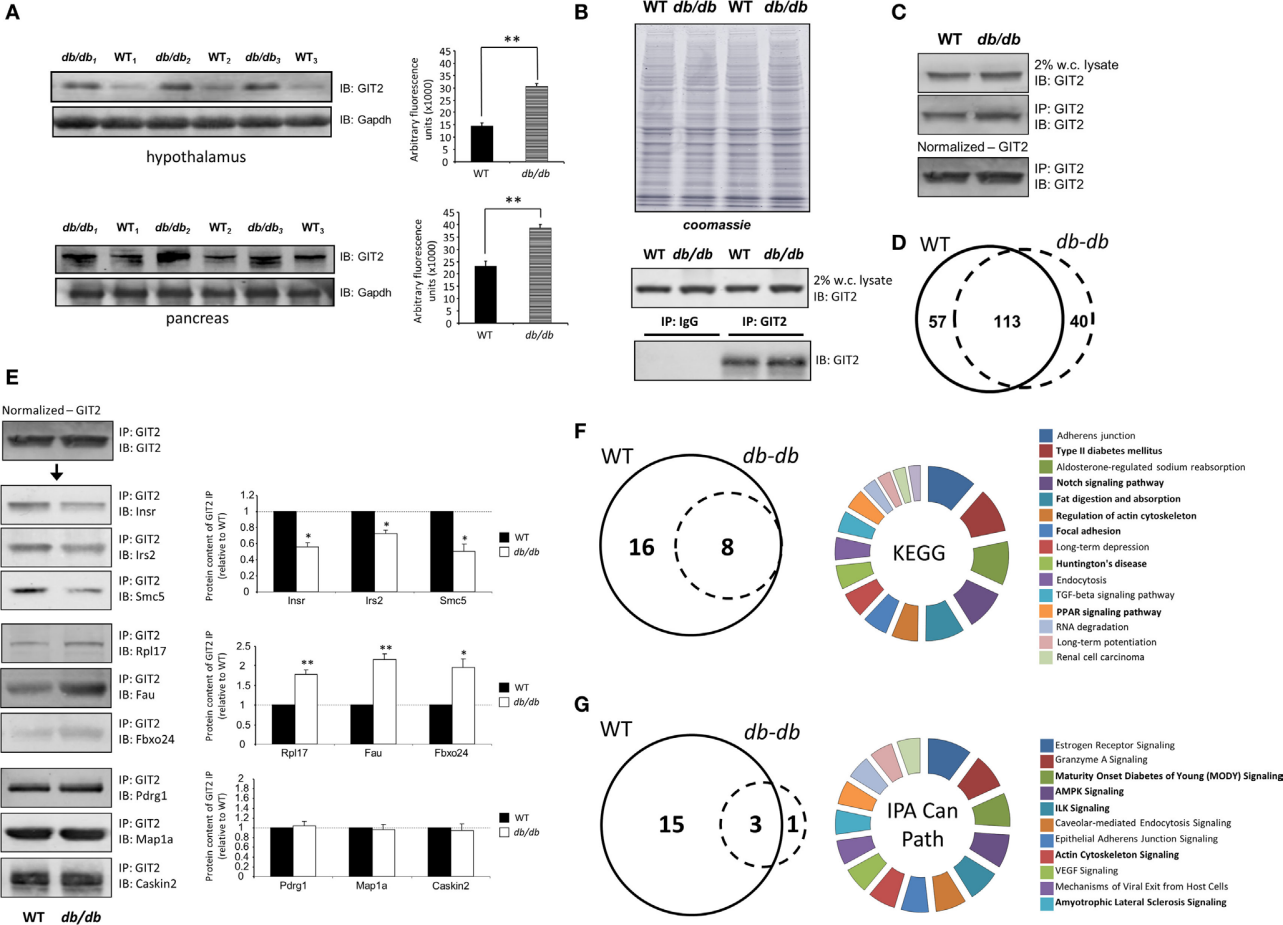
**Pathological metabolic status directly affects GIT2 expression**. **(A)** Advanced metabolic stress, independent of chronological age, potentiates GIT2 expression in hypothalamic and pancreatic tissues. **(B)** Selective immunoprecipitation of GIT2 (using pre-immune IgG as a negative control) from WT or *db/db* pancreatic whole-cell lysates. Coomassie staining of lysates is employed to indicate equal protein input for immunoprecipitation experiments. **(C)** Normalization of immunoprecipitated GIT2 from pancreatic lysates to adjust for *db/db*-induced elevation of GIT2. Normalized GIT2 co-immunoprecipitation extracts were then used for further semi-quantitative Western blot or mass spectrometry (MS) analysis. **(D)** Proportional Venn diagram analysis of the protein compositions of GIT2 co-immunoprecipitates from WT (solid line) of *db/db* (dashed line) pancreatic lysates. **(E)** Western blot validation of MS-identified GIT2-associating partners in WT and *db/db* pancreatic tissue lysates. WT data is indicated by black bars while *db/db* data is represented by white bars. **(F)** Proportional Venn diagram analysis of KEGG pathways significantly populated by GIT2 co-immunoprecipitation data from either WT (solid line) or *db/db* (dashed line) pancreatic lysates. The associated pie chart indicates the relative KEGG pathway hybrid scores [(−log_10_ (enrichment probability)) × enrichment ratio] for the 16 KEGG pathways unique to the WT dataset. Pathways related to metabolism, cytoskeletal signaling and disease are highlighted in bold. **(G)** Proportional Venn diagram analysis of IPA Canonical Signaling pathways significantly populated by GIT2 co-immunoprecipitation data from either WT (solid line) or *db/db* (dashed line) pancreatic lysates. The associated pie chart indicates the relative Canonical pathway score enrichment probabilities (−log_10_ transformed) for the 15 Canonical Signaling pathways unique to the WT dataset. Pathways related to metabolism, cytoskeletal signaling and disease are highlighted in bold. Statistical significance is indicated in each figure as *p ≤ 0.05; **p ≤ 0.01; ***p ≤ 0.001.

As GIT2 genomic deletion induces a strong metabolic phenotype to appreciate the effects of metabolic stress upon GIT2 functionality, we next investigated the potential functional interaction partners of GIT2 in pancreatic tissue from experimental animals experiencing significant metabolic stress, i.e., diabetic *db/db* mice (55, 96). *Db/db* mice possessing a mutation in the leptin receptor demonstrate an increased propensity for diet-induced obesity and diabetes via congenital hyperphagia (97). Upon inspection of the GIT2 expression levels in 8-m.o. WT control and *db/db* mice, in both the hypothalamus and pancreas GIT2 expression was significantly elevated on *db/db* mice compared to the controls (Figure 10A). Therefore, in a pro-diabetic state, GIT2 expression is elevated, suggesting that GIT2 expression is not only age- and stress-sensitive (13, 18, 19), but also sensitive to the alteration of “metabolic” age in the *db/db* model (42, 98–100). To identify the effects of diabetes-related pathology on GIT2 protein–protein complex interactions, we performed selective GIT2 co-immunoprecipitation (co-IP) (with pre-immune sera as a negative control) in WT and *db/db* pancreatic cell lysates (Figure 10B). Due to the elevation of GIT2 expression in *db/db* samples (Figures 10B,C), we normalized for GIT2 content of the co-IP samples used for subsequent Western blotting or MS analysis. Investigation of extracted and digested GIT2 co-IP samples with our linear ion trap LC-MS/MS identified 170 specific co-precipitating proteins from WT pancreatic lysates (Table S16 in Supplementary Material) and 153 specific co-precipitating proteins from *db/db* pancreatic lysates (Table S17 in Supplementary Material). The majority of these proteins were conserved (65%) between WT and *db/db* co-IP extractions (Figure 10D). However, it was interesting to note that within the proteins found only in the WT venn sector, i.e., lost with metabolic dysfunction, multiple glycemic-related factors were evident (Table S18 in Supplementary Material), e.g., the insulin receptor (Insr), insulin receptor substrate 2 (Irs2), fatty acid binding protein 2 [Fabp2 (101)], signal sequence receptor delta [Ssr4 (102)] and G protein-coupled receptor 101 [Gpr101 (103)]. In addition to these WT-specific metabolic factors, it was also evident that additional proteins within the WT-only venn sector were associated with other activities strongly implicated in pathological aging including molecular aging, DNA damage and stress, e.g., structural maintenance of chromosomes 5 [Smc5 (104)], eukaryotic translation initiation factor 2B [Eif2b2 (105)], negative regulator of ubiquitin-like proteins 1 [Nub1 (106)], and DEAD (Asp–Glu–Ala–Asp) box helicase 3, X-linked [Ddx3x (107)]. We validated our MS data using selective Western blotting for several of the GIT2 co-precipitating factors unique to WT or *db/db* tissues (Figure 10E). Hence, we found a reduced association between GIT2 and Insr, Irs2, and Smc5 in the *db/db* samples. In contrast, we found an increased association between GIT2 and Rpl17 (ribosomal protein L17), Fau/MNSFβ (Monoclonal non-specific suppressor factor β), and Fbxo24 (F-Box Only Protein 24/IKK1) in the pathological *db/db* state. Interestingly, both Fau/MNSFβ and Fbxo24 are associated with apoptotic activity linked to inflammation and DNA damage (108, 109). Proteins commonly co-precipitating with GIT2 in both WT and *db/db* conditions (Map1a, Pdrg1, Caskin2) were also validated and were found to equally associate with GIT2 in both conditions (Figure 10D). In addition to the investigation of individual GIT2 protein–protein complex interactions, we also investigated the potential cell signaling ramifications of these multiple associations. Using both KEGG (WT mice: Table S19 in Supplementary Material, Table S20 in Supplementary Material: *db/db* mice) and IPA Canonical Signaling Pathway analyses (WT mice: Table S21 in Supplementary Material, Table S22 in Supplementary Material: *db/db* mice) of the respective co-IP datasets we again demonstrated a strong distinction between the functional links of GIT2-associated proteins in control WT conditions compared to *db/db* (Figures 10F,G). Inspection of the selective WT-unique signaling pathways (Figures 10E,F) revealed a strong link of GIT2-associated proteins with energy regulation (“*Type II diabetes mellitus signaling,” “Fat digestion and absorption,” “PPAR signaling pathway,” “Maturity Onset Diabetes of Young (MODY) Signaling,” “AMPK Signaling”*), cellular development (“*Notch signaling”*) and degenerative diseases linked to metabolic dysfunctions [“*Huntington’s disease,” “Amyotrophic lateral sclerosis signaling”* (53, 110)]. As expected from the scaffolding nature of GIT2 and its well-characterized role in cytoskeletal regulation (111), a strong representation of such factors was evident (Figures 10E,F: “*Regulation of actin cytoskeleton,” “Focal adhesion,” “Actin Cytoskeleton Signaling”*).

## Discussion

Given the inevitable worldwide rise in the incidence of age-related disorders, it is imperative that a more nuanced understanding of the systemic control of the multiple processes that underpin aging is developed. Intensively complex, multidimensional events such as whole somatic aging are likely to be coordinated by a series of differential protein hierarchies, in which lower-dimensionality protein functions, e.g., simple mitogen-activated protein kinase (MAPK) cascades are orchestrated and connected to other linear pathways by master-controlling “keystone” or “hub” proteins (13). Experimental research into the potential molecular interactions that regulate aging across an organisms life span quickly engenders signaling complexity issues comparable to the mathematical difficulties of optimally solving NP-hard (Non-deterministic Polynomial-time hard) or NP-complete calculations (112, 113). Therefore, a more pragmatic approach to the investigation of hyper complex signaling architectures, both for mechanistic knowledge and for eventual therapeutic manipulation of highly complex systems, is to identify the central coordinators of these complex systems. We have previously illustrated a mechanism for uncovering such “keystone” regulatory factors [also known as “hub” proteins (114) using our combinatorial bioinformatics approach (13)]. Performing such an analysis with primary protein-based data, extracted from different aged samples of an endocrine organ thought to house the regulatory machinery of aging, i.e., the hypothalamus (24), we identified the GPCR-interacting protein GIT2 as a “keystone” of the aging process. Subsequently we demonstrated that this protein forms a link between age-related cellular damage (ROS, ionizing radiation) and the maintenance of DNA repair/stability – a process crucial to successful and healthy aging (18). Genomic deletion of GIT2 resulted, with respect to the presence of accumulated DNA damage, in accelerated aging of murine CNS tissue. As the age-dependent development of insulin resistance and the deterioration of glucose-focused oxidative phosphorylation efficiency contribute significantly to accelerated aging mechanisms, we sought to investigate the potential role of GIT2 in systemic metabolic regulation. Using whole-somatic metabolic chamber analysis, we found that GIT2KO mice demonstrate a significantly reduced RER at a relatively young (4 months) age indicating a switch away from glucose use toward adipose and then, potentially, protein to generate usable energy (Figure 1). Upon investigation of the hypothalamic transcriptomic profiles in GIT2KO mice at younger and older ages (2 and 8 months old respectively), we uncovered, using multiple bioinformatics techniques, a core transcriptomic “signature” linked to metabolic/glycemic regulatory activity (Figure 2D, Figure S1). We further reinforced this observation using classical pathway annotation pipelines (KEGG pathway analysis: Figure 3) as well as novel natural language processing-based investigations using the hypothalamic transcriptomic data (Figures 3 and 4). In these highly nuanced data outputs, a strong intersection between classical GIT2 functions (endocytosis and cytoskeletal organization; Figures 3 and 4) (111), novel functions (DDR; Figure 4) (18), and metabolic activity (glucose, mitochondrial, insulin, and fatty acid oxidation: Figure 4) was evident. Given the multiple indications of an important role of GIT2 in glycemic regulation, we investigated a potential direct impact of GIT2 in pancreatic tissues that secrete insulin and related metabolic factors. We found that GIT2 was expressed in pancreatic alpha and beta islet cells and that genomic deletion in GIT2KO mice resulted in a disruption of both beta and alpha cell distribution in the islets. Reinforcing the potential for GIT2 to act as a neurometabolic controller, we also found moderately diminished levels of the GIT2-associaciated signaling factors Pak1 (p21 activated kinase 1) and beta-PIX (Arhgef7, Rho guanine nucleotide exchange factor 7) in both GIT2KO hypothalamus and the pancreas compared to WT controls (Figure S10 in Supplementary Material). Arhgef7 has been demonstrated to play a role in insulin secretion via regulation of Cdc42 (cell division control protein 42 homolog) activity (115). In addition the downstream GIT effector Pak1, through physical binding and eventual phosphorylation, can control the activity of phosphoglucomutase 1, an important regulatory enzyme in cellular glucose utilization and energy homeostasis (116). In pancreatic β-cells, Pak1 is also involved in insulin granule localization and vesicle release (117), as well as playing a role in pancreatic cell development and incretin generation (118).

At the gross pancreatic histological level the GIT2KO mice effectively demonstrated (primarily at 8 m.o.) a classical alpha-cell involution of the insulin-secreting beta cell mass (Figure 5) (77). This was accompanied by reductions in circulating levels of insulin, amylin, leptin, and peptide YY in 8-m.o. GIT2KO mice compared to WT controls (Figure 6). These multiple pancreatic alterations were associated with elevated fasting blood glucose, insulin (Figure 6), and oral glucose tolerance and a diminished insulin secretion capacity in response to glucose stimulation (Figure 6). We further reinforced the validity of these findings via a high-dimensionality transcriptomic investigation of isolated GIT2KO pancreatic islets. Using IPA Disease/Bio-Function analysis of the islet data, a strong unbiased demonstration of beta cell developmental disruption was revealed (Figure 7). Using murine beta cell culture models, we also found that the functional activity of GIT2 in these cells was disrupted by cellular stress that mimics insulin resistant pathophysiology (Figure 9), i.e., palmitate treatment. While representing a commonly employed experimental process (119–122), it is highly likely that palmitate-induced insulin resistance is a complex multifactorial process that can involve multiple collateral signaling systems effects including altered ceramide levels (123), endoplasmic reticulum stress (124), mTOR/S6K activation (125), activation of protein phosphatase 2A (126), modulation of TNF-α signaling (127), and alterations in transmembrane free fatty acid receptor activity (128–130). It is interesting to note however, with specific respect to the functional intersection between insulin resistance and the aging process, Nakamura et al. (131) suggested that palmitate-induced insulin resistance in their model was strongly associated with mitochondrially derived ROS, a pathological process that is considered one of the hallmarks of aging (132). Linked to this it is therefore not surprising that in addition to its elevation in aging (13), GIT2 expression is enhanced by both direct oxygen radical exposure (19) and here in our current research with palmitate treatment. In line with the interconnection between aging and metabolic paradigms we subsequently demonstrated that in the *db/db* pathological paradigm GIT2 was prematurely upregulated compared to WT control mice, both in the hypothalamus and pancreas (Figure 10). In addition, we found in pancreatic tissues that the physical association of GIT2 with proteins involved in the glucose metabolic/insulin-regulatory system, e.g., insulin receptor and insulin receptor substrate 2, were disrupted in the *db/db* pathological state (Figure 10). With respect to the large number of potential GIT2-associating factors identified in our MS experiments, it is interesting to note that multiple interactome databases have already empirically identified well over 50 different partners, e.g., BioGRID reports 59 different GIT2 interactors^13^ and IntAct identifies at least 75 distinct GIT2 interactors^14^. The introduction of high-dimensionality proteomics-based protein complex analysis has greatly expanded the ability to discover both direct binary and co-complex binding partners (133–136). We believe that due to its widespread expression (46) and modular structure, GIT2 is likely to possess a broad spectrum of potential interacting partners and therefore our finding of a large number of potentially interacting proteins may not unexpected. GIT2 possesses multiple protein–protein interaction domains including a GAP (GTPase-activating protein) domain, two paxilin-binding subdomains (PBS1, PBS2), three ankyrin repeats, a Spa2-homology domain (SHD), a focal adhesion-targeting homology (FAH) domain, a coiled-coil region, a leucine zipper motif, and a synaptic localization domain (111). Hub or keystone proteins, which we contend GIT2 may be, are considered to play a potentially pivotal role in organizing complex molecular signaling networks via their ability to exert a more trophic level of signaling control (137–139). Using an unbiased discovery process of identifying proteins controlling multidimensional neurometabolic signaling networks in aging, we previously sought to discover target proteins that were associated with significantly more multiple signaling pathways compared to other proteins (13). Therefore, it not surprising that GIT2 may indeed function as a hub protein as aging affects nearly every physiological system in the body. The seemingly large array of protein interactions suggested for such hub proteins may reflect the ability of multiple proteoforms of the same protein to interact physically with several distinct signaling protein complexes simultaneously (140). Therefore, for any given protein, there are likely to be an ensemble of diverse interactomes that, unless differentially purified, will generate the appearance of a huge number of potential interacting proteins.

Highly connected hub proteins have been intensively studied in recent years as they potentially represent important therapeutic targets (141). Hub proteins have been themselves categorized into two general types, i.e., “Party hubs” and “Date hubs” (139, 142). The “Party” types are hubs whose expression is correlated with the expression levels of their interaction partners while “Date” types do not demonstrate this correlation. Linked to this distinction “Party” hubs are considered more likely to connect proteins within functional signaling modules, while “Date” hubs are more likely to connect different functional signaling modules. ”Party” hubs are generally considered to be multi-interface proteins whereas “Date” hubs are more frequently single-interaction interface proteins (143). However, such strong distinctions of the nature of hub proteins though are unlikely to be indicative of the reality of multifunctional proteins (144, 145). In the case of GIT2, and potentially many other “hub” proteins it is likely that it can, at various times depending on the specific cellular context, act as a “Party” hub, e.g., controlling skeletal dynamics, and act at times as a “Date” hub and cross-regulate multiple pathways involved in aging and metabolism, e.g., glycemic signaling and DNA repair. With regards to the pathophysiological functionality of GIT2, it is interesting to note that research over the last decade has generally concluded that human disease is rarely the consequence of an isolated abnormality in a particular gene or protein but is probably due to a series of complex perturbations in an underlying cellular network (141), in this case the neurometabolic “axis.” The structure of these networks/axes is likely governed, at some level by “hub” or keystone proteins, whose alterations can exert changes in their global properties thus linking their function to multiple disease processes (141, 146, 147). Further research into hub protein function is likely to uncover the biological significance of these network controllers in disease etiology (147) as well as help in the delineation of trophic biomarkers and novel drug targets (141).

Our multifactorial analyses concerning the physiological status of the GIT2KO mice reveals a potentially important factor for the generation of aging trajectories, i.e., the metabolic shift away from primary glucose use to glucose/adipose use and then finally to adipose/protein use. Each of these transitions results in the reduction of RER away from a value of 1.0, i.e., glucose as primary energy source, toward 0.85–0.8, i.e., glucose/adipose as primary energy source or toward <0.8, i.e., adipose/protein as a primary energy source. Our observed decline in RER of relatively young GIT2KO mice is in line with reports of age-dependent shifts in C57BL/6 mice (148). Maintaining metabolic efficiency, through preservation of the utilization of glucose as the primary energy source, in early and later life, will potentially reduce hyperglycemia-induced chronic inflammation (149, 150), attenuate generation of ROS (151, 152), and also support the activity of energetic processes such as membrane electrical excitability (153), activity of cation pumps (154), unfolded protein management (155, 156) and nucleic acid damage (157, 158). Therefore, in-part, the transition from “healthy” aging to “pathological” aging may coincide with a significant downshift in RER as nearly all of the processes entrained by energy insufficiency result in feed-forward pathological loops. An enhanced understanding of the molecular events that could predict this event would therefore provide potential remedial mechanisms to postpone this transition. As GIT2 possesses important roles in stress sensitivity (18, 19, 159), aging (13), and somatic energy management, this molecule potentially provides a novel target for therapies designed to interdict pathological aging and age-related disease. With respect to potential therapeutic exploitation of this keystone factor, our transcriptomic and proteomic analyses have demonstrated that GIT2 is strongly linked with multiple therapeutically tractable protein targets, e.g., glyoxylase 1 [Glo1: Figure 8 (160–163)] and sestrin 1 [Sesn1: Figure 8; (82, 92, 164, 165)]. Considerable further experimentation will be clearly required to uncover the optimal therapeutic mechanisms of exploiting GIT2 expression for the remediation of age-related pathologies, however, at the present time, it represents an interesting new master-regulator of the neurometabolic aging process.

With the advent of such a nuanced hierarchical approach, specifically of functional keystone factors, to the understanding of physiological and disease protein networks our concept of disease causation needs to take this into account. If potential keystone factors such as GIT2 are more important than other proteins with respect to their organization of other signaling modalities, it would be presumed that genomic deletion/modification may result in the generation of significant health issues. In our hands, aside from the metabolic alterations studied here, we have found that with *post-mortem* tissue extraction from aged GIT2KO males (12–18 months of age), we have identified consistent structural deformation of their upper gut (primarily ileal distention) as well as renal/hepatic adipose deposition. It is interesting to note however that potential “hub” or keystone proteins, as we propose GIT2 to be an example of, while being crucial for the organization of complex molecular events are not often the sole causes of disease (114, 166). Due to their trophic level of signaling pathway interactivity hub proteins are not often observed as genomic loci of disease (142) but if they are perturbed they will likely generate a wide spectrum of related pathologies. For example, genetic alteration of GIT2 has been identified in a GWAS risk allele screen for MetS (167). MetS represents a multifactorial disorder including an incredibly broad array of pathophysiologies including hypertriglyceridemia, insulin resistance, hypertension, vascular inflammation, atherosclerosis and renal, liver, and heart diseases (168). Therefore, it is perhaps not surprising that the GIT2KO mice do not present such a singular dramatic pathological phenotype as the effects of GIT2 deletion are likely to be highly multidimensional: GIT2 genomic deletion has already been shown to affect central nervous system aging and DNA repair (18), immune function (169), bone mineral density, bone marrow adiposity (170), and anxiety/stress phenotypic behavior (159). Also, associated with issues of congenital knockout models, it is clear that there is a strong reflexive survival response of the GIT2KO mice to metabolic disruption. For example, we have noted that while presenting a distinct metabolic phenotype, male GIT2KO mice on a regular chow diet (not high in fat or glucose) maintain a relatively similar bodyweight across their moderately reduced total life span. This interesting phenomenon is currently the subject of our ongoing research. In addition, the GIT2KO mice appear to be attempting to systemically ameliorate the deleterious effects of elevated glucose levels by increasing the expression of Glo1. Protracted exposure to excessive blood glucose can lead to the formation and accumulation of advanced glycation endproducts (AGEs) that are related to diabetes and other age-related diseases. Methylglyoxal (MGO), a highly reactive dicarbonyl compound, is considered to be one of the major precursor in the formation of AGEs and therefore is strongly implicated in accelerated pathophysiological aging mechanisms linked to metabolic instability (89). MGO is a cytotoxic, atherogenic (171), and neurotoxic (172) glycolytic intermediate that is elevated in diabetic patients (173, 174) as well as those with cognitive decline (175). Under physiological circumstances, MGO is detoxified by the glyoxalase system into D-lactate, with Glo1 as the key enzyme in the anti-glycation defense. Therefore, it is likely that congenital models of metabolic disruption, associated with a premature aging such as GIT2KO mice, may indeed help illuminate many of the endogenous protective mechanisms at the same time as demonstrating the pathophysiological effects of the genetic disruption.

**Figure 11:**
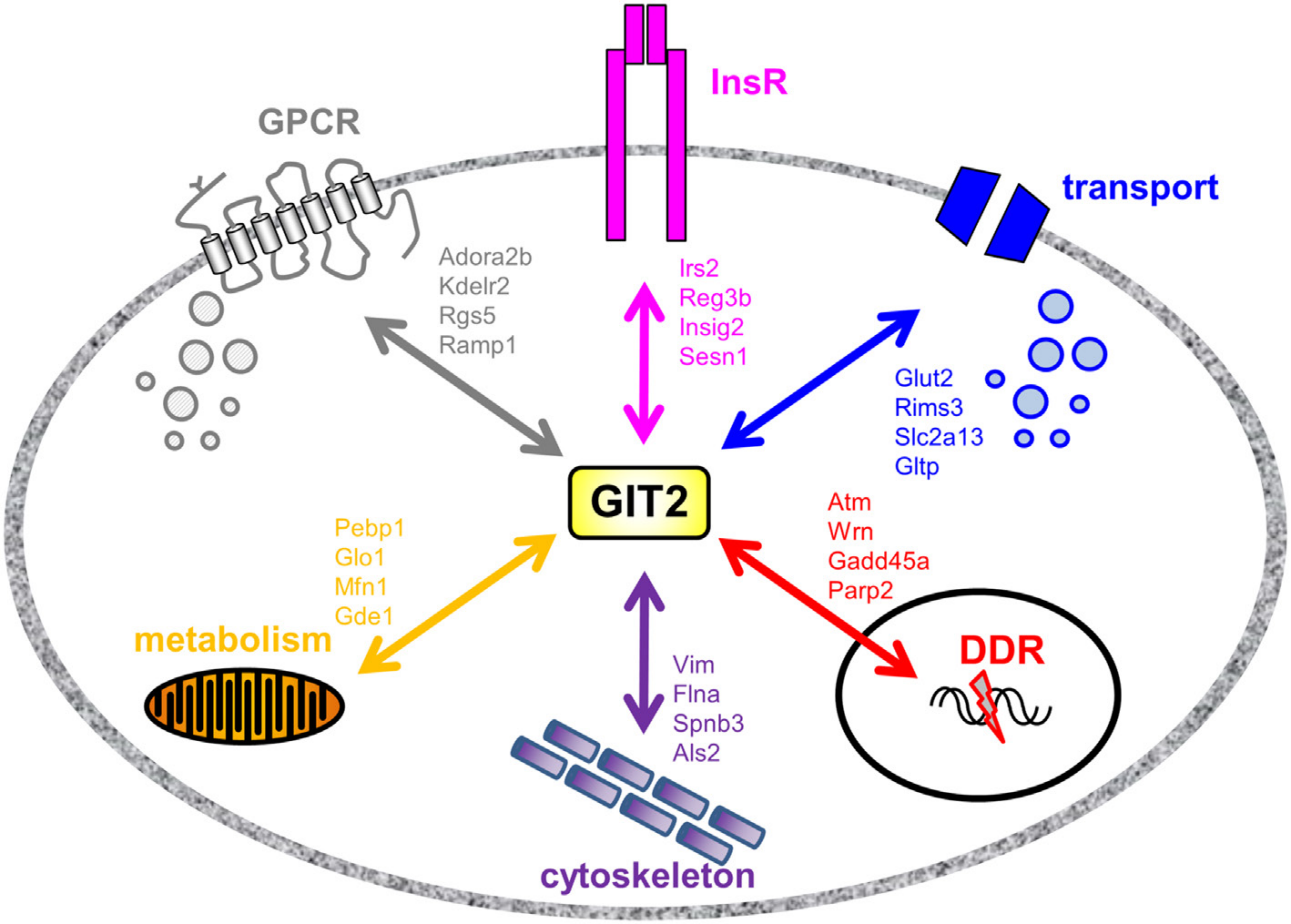
**Multifactorial functionality of GIT2 in the neurometabolic axis**. From our data presented here and from previous research it is becoming clear that GIT2 likely acts as a keystone or hub protein for many physiological or cellular signaling processes involved in the maintenance of homeostatic metabolic function across life span. To illustrate the multidimensional functionality of GIT2 in such a complex system we have selected proteins/transcripts identified in this manuscript to highlight the network-level functional signature of GIT2 activity. The ability of GIT2 to simultaneously control and integrate proteins involved in *GPCR signaling/trafficking* (Adora2b, adenosine A2b receptor; Kdelr2, Lys–Asp–Glu–Leu ER lumen protein retaining receptor 2; Rgs5, regulator of G-protein signaling 5; Ramp1, receptor activity-modulating protein 1), *insulin receptor (InsR) associated signaling* (Irs2, insulin receptor substrate 2; Reg3b, regenerating islet-derived 3 beta; Insig 2, Insulin induced gene 2; Sesn1, sestrin 1), *metabolite/vesicular transport* (Glut2, glucose transporter 2; Rims3, regulating synaptic membrane exocytosis 3; Slc2a13, solute carrier family 2 (facilitated glucose transporter), member 13; Gltp, glycolipid transport protein), *DNA damage repair (DDR)* (Atm, ataxia-telangiectasia mutated; Wrn, Werner syndrome ATP-dependent helicase; Gadd45a, growth arrest and DNA-damage-inducible protein GADD45 alpha; Parp2, Poly (ADP-ribose) polymerase 2), *cytoskeletal remodeling* (Vim, vimentin; Flna, filamin A; Spnb3, spectrin beta-3; Als2, Alsin) *and energy metabolism* (Pebp1, phosphatidylethanolamine binding protein 1; Glo1, glyoxylase 1; Mfn1, mitofusin 1; Gde1, glycerophosphodiester phosphodiesterase 1) clearly demonstrates keystone functionality of GIT2 in neurometabolic aging. Therefore the modulation of the expression of this protein, its post-translational and subcellular distribution are likely to strongly influence the orchestration of these vital functions in both health and pathophysiology.

Our observations that GIT2 expression is modulated by stress (18, 19), aging (13) and metabolic pathologies (*db/db*) underscores the contextuality of what we consider to be “aging.” Therefore, rather than the process of aging being dictated merely by simple chronology, the effective physiological rate of aging (linked to damage accumulation) is considerably plastic and is contingent on the interaction and coordination of, multiple pathophysiological signaling pathways simultaneously across multiple tissues of the body. Acting as a protein with an ability to integrate numerous physiological processes intrinsic to the control of healthy aging, e.g., GPCR signaling systems (176), insulin/IGF-1 signaling systems (177), neurometabolic transport processes (178, 179), DNA damage repair (39), cytoskeletal organization (180), and energy metabolism (181) GIT2 may represent an important target for both further diagnostic and therapeutic research for age-related disorders. An important component of this future research however should be dedicated to the appreciation of how these differential cellular requirements may interact with each other via their co-dependence on this interesting keystone protein (Figure 11). Hence a more nuanced and contextually sensitive understanding of how multiple complex signaling systems are integrated and regulated will profoundly enhance our appreciation, and hopefully successful treatment, of age-related disorders.

## Author Contributions

BM and SM designed the work. WC, RS, SS, SSP, W-NC, CMD, BM, and SM performed experiments. BM, SS, SSP, W-NC, BM, SM, RP, JJ analyzed and interpreted the data. BM, WC, SM, and JJ wrote the manuscript.

## Conflict of Interest Statement

The authors declare that the research was conducted in the absence of any commercial or financial relationships that could be construed as a potential conflict of interest.

## Footnotes

^1^www.atcc.org/

^2^http://jaxmice.jax.org/index.html

^3^http://jaxservices.jax.org/glucosetolerance_protocol.html

^4^http://www.ncbi.nlm.nih.gov/pmc/

^5^http://www.ncbi.nlm.nih.gov/omim

^6^http://www.informatics.jax.org/

^7^http://www.wordle.net/

^8^http://www.writewords.org.uk/word_count.asp

^9^http://www.grc.nia.nih.gov/branches/rrb/dna/dna.htm

^10^http://www.sas.com/en_us/home.html

^11^http://www.wordle.net/

^12^www.writewords.org.uk/phrase_count.asp

^13^http://thebiogrid.org/

^14^http://www.ebi.ac.uk/intact/
