# Supplementary material for: GIT2 Acts as a Systems-Level Coordinator of Neurometabolic Activity and Pathophysiological Aging
Source: Front Endocrinol (Lausanne). 2016 Jan 18;6:191. doi: 10.3389/fendo.2015.00191 (PMC4716144; doi:10.3389/fendo.2015.00191)
Supplement: Supplementary file 1 [file Data_Sheet_1.PDF]

Data Supplement

GIT2 acts as a systems-level coordinator of neurometabolic activity and pathophysiological aging

Bronwen Martin[1*], Wayne Chadwick[2*], Jonathan Janssens[3,4*], Richard T. Premont[5], Robert Schmalzigaug[5], Kevin G. Becker[6], Elin Lehrmann[6], William H. Wood III[6], Yongqing Zhang[6], Sana Siddiqui[2], Sung-Soo Park[2], Wei-na Cong[1], Caitlin M. Daimon[1], Stuart Maudsley[2,3,4†]

1. Metabolism Unit, National Institute on Aging, National Institutes of Health, Baltimore, USA.
2. Receptor Pharmacology Unit, National Institute on Aging, National Institutes of Health, Baltimore USA.
3. Translational Neurobiology Group, VIB Department of Molecular Genetics, University of Antwerp.
4. Laboratory of Neurogenetics, Institute Born-Bunge, University of Antwerp.
5. Duke University Department of Medicine, Gastroenterology Division, Durham USA.
6. Gene Expression and Genomics Unit, National Institutes of Health, 251 Bayview Blvd., Suite 100, Baltimore, MD 21224, USA

† Corresponding author: Dr. Stuart Maudsley, Translational Neurobiology Group, VIB-Department of Molecular Genetics, University of Antwerp, Universiteitsplein 1, Antwerp 2610. E-mail: stuart.maudsley@molgen.vib-ua.be

Index to Supplemental Files

I. Supplemental Figures

Figure S1. Collective Textrous! processing analysis of commonly-regulated hypothalamic transcripts from GIT2KO mice of 2, 4 and 8 months of age.
Figure S2. Genomic GIT2 deletion affects pancreatic islet functional structure in GIT2KO mice.
Figure S3. Functional beta and alpha structure of 4 or 2 month old GIT2KO mice islets.
Figure S4. GIT2 genomic deletion affects multiple circulating energy-regulatory factors in 2 or 4 month old mice.
Figure S5. IPA-mediated functional signaling network analysis of pancreatic islet transcriptomic data.
Figure S6. IPA-mediated functional signaling network analysis of pancreatic islet transcriptomic data.
Figure S7. Cell survival analysis of palmitate treatment of murine TC-6 cells.
Figure S8. Western blotting verification of selectivity of differential detergent fractionation process.
Figure S9. Cellular depletion of GIT2 partially attenuates responsive glucose uptake in murine TC-6 cells.
Figure S10. Genomic deletion of GIT2 results in reduced expression of GIT-associated signaling factors in hypothalamic and pancreatic tissues.

II. Supplemental Data Tables

Table S1. Significantly-regulated transcripts differentially regulated in 2 month old GIT2KO mice compared to age-matched WT controls.
Table S2. Significantly-regulated transcripts differentially regulated in 4 month old GIT2KO mice compared to age-matched WT controls.
Table S3. Significantly-regulated transcripts differentially regulated in 8 month old GIT2KO mice compared to age-matched WT controls.
Table S4. Textrous! Collective analysis of significantly-regulated hypothalamic transcripts common across GITKO mice of 2, 4 and 8 months of age compared top age-matched controls.
Table S5. User-defined input interrogator terms for hypothalamic transcriptome transcript-word association analysis across the three experimental timepoints.
Table S6. GeneIndexer age-ranking correlation matrix for 2 month old transcriptomic data (Table S6.xlsx).
Table S7. GeneIndexer age-ranking correlation matrix for 4 month old transcriptomic data (Table S7.xlsx).
Table S8. GeneIndexer age-ranking correlation matrix for 8 month old transcriptomic data (Table S8.xlsx).
Table S9. GeneIndexer LSI-based targeted analysis of hypothalamic transcriptomic data from GIT2KO mice.
Table S10. KEGG signaling pathway analysis of hypothalamic transcriptomic data from 2 m.o. GIT2KO mice.
Table S11. KEGG signaling pathway analysis of hypothalamic transcriptomic data from 4 m.o. GIT2KO mice.

Table S12. KEGG signaling pathway analysis of hypothalamic transcriptomic data from 8 m.o. GIT2KO mice.

Table S13. KEGG-based multidimensional keystone analysis matrix (Table S13.xlsx).

Table S14. Significantly-regulated transcripts differentially regulated in 8 month old GIT2KO mouse extracted pancreatic islets compared to age-matched WT control islets.\

Table S15. Ingenuity Pathway analysis (IPA)-Disease/Bio-Function analysis of the 8 month old GIT2KO pancreatic islet transcriptome.

Table S16. Proteins selectively co-immunoprecipitating with GIT2 in WT pancreatic whole-cell lysates.

Table S17. Proteins selectively co-immunoprecipitating with GIT2 in *db/db* pancreatic whole-cell lysates.

Table S18. Venn diagram analysis of co-IP proteins from WT or *db/db* pancreatic lysates.

Table S19. KEGG pathway analysis of proteins selectively co-immunoprecipitating with GIT2 in WT pancreatic whole-cell lysates.

Table S20. KEGG pathway analysis of proteins selectively co-immunoprecipitating with GIT2 in *db/db* pancreatic whole-cell lysates.

Table S21. IPA Canonical Signaling Pathway analysis of proteins selectively co-immunoprecipitating with GIT2 in WT pancreatic whole-cell lysates.

Table S22. IPA Canonical Signaling Pathway analysis of proteins selectively co-immunoprecipitating with GIT2 in *db/db* pancreatic whole-cell lysates.

I. Supplemental Figures

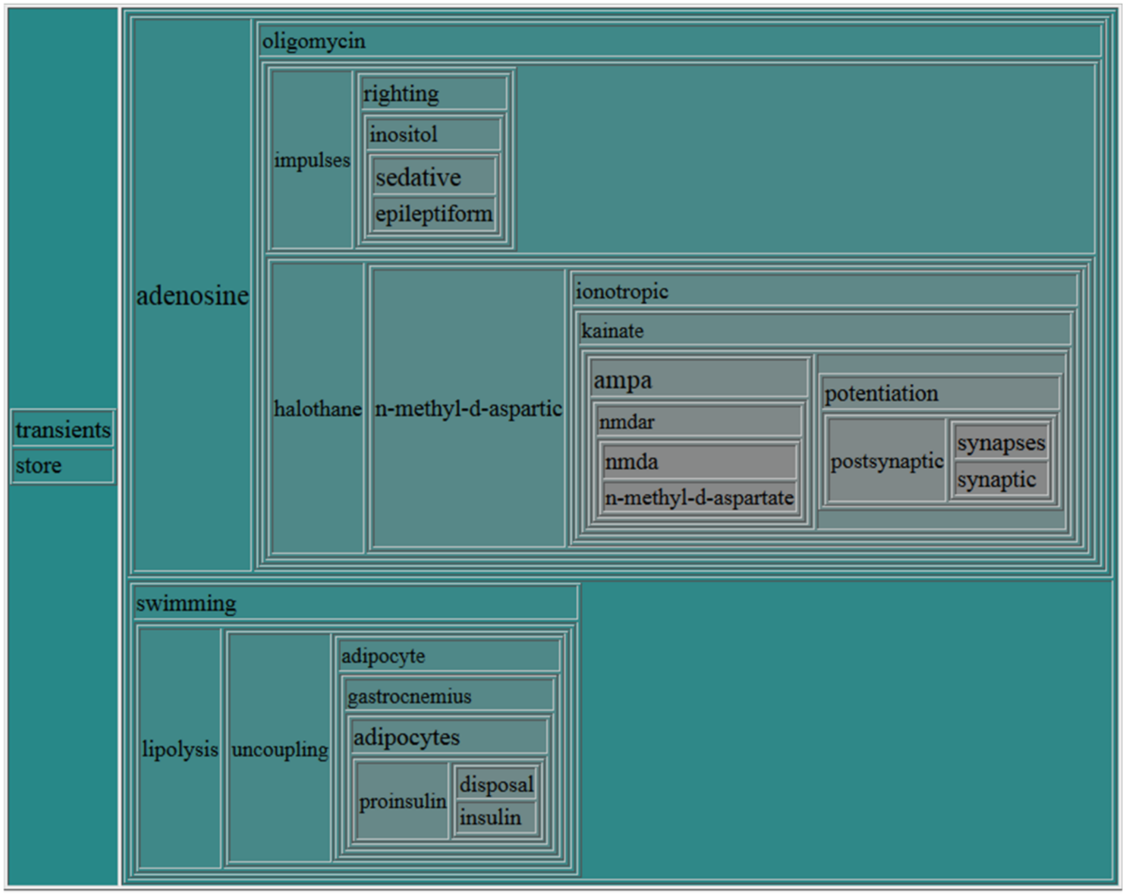

Figure S1. Collective Textrous! processing analysis of commonly-regulated hypothalamic transcripts from GIT2KO mice of 2, 4 and 8 months of age. The figure comprises a hierarchical word cloud generated using the collective processing module of the natural language informatics processor, Textrous!. Extracted scientifically-relevant nouns are arranged by their inter-relationships, demonstrating diverse functional domains of input dataset functionality. Words possessing higher degrees of correlation to the input transcriptomic data occupy the more red areas of the cloud while words in the more green areas possess a weaker semantic correlation to the input transcriptome data.

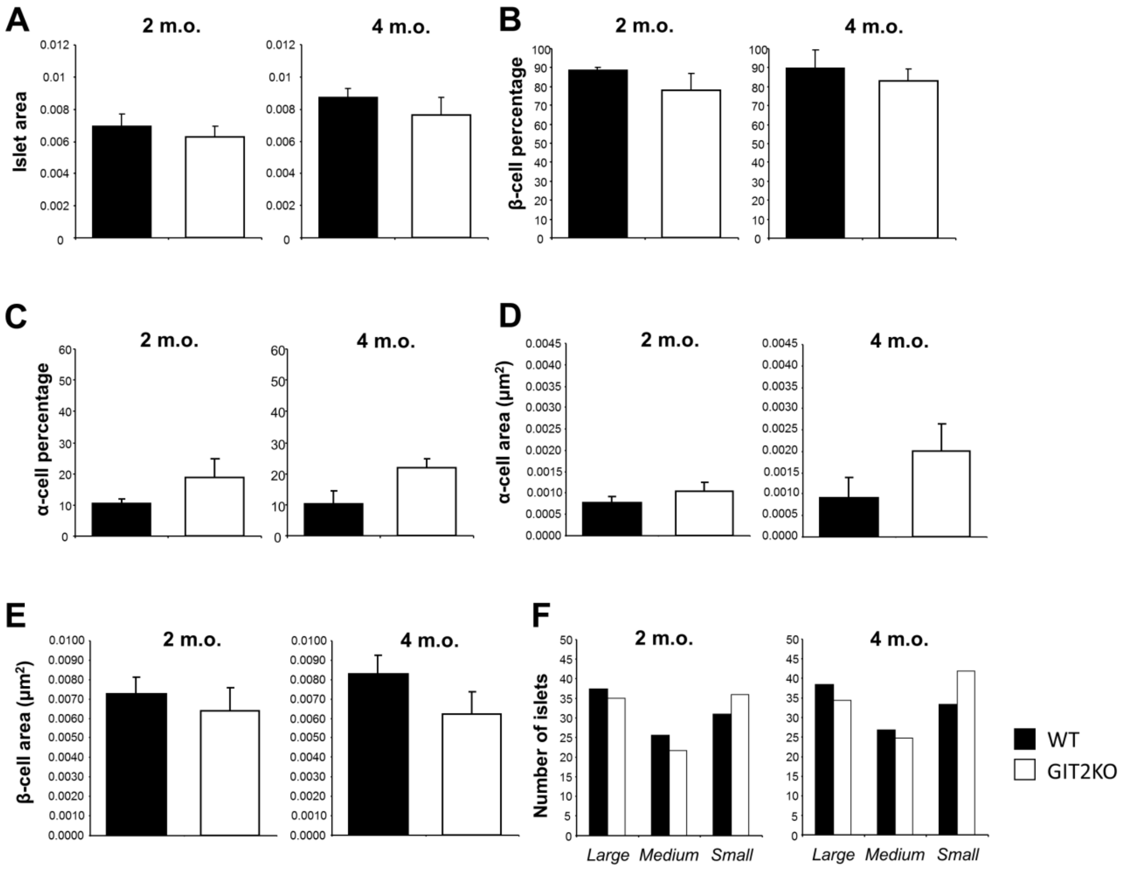

Figure S2. Genomic GIT2 deletion affects pancreatic islet functional structure in GIT2KO mice. A MATLAB-based pancreatic islet mathematical automated assessment of the substructural nature of both 2 m.o. (month old) and 4 m.o. GIT2KO mice (white bars) compared to WT control (black bars) was applied to investigate the effect of GIT2 deletion. Minimal differences in total islet area between WT and GIT2KO at the 2 or 4 m.o. timepoints were noted **(A)**. No significant differences at the 2 or 4 m.o. timepoints between WT or GIT2KO mice were noted for β-cell percentage of the islets **(B)**. At both the 2 and 4 m.o. timepoints a non-significant increase in pancreatic α-cell percentage was seen in GIT2KO compared to WT mice **(C)** – this effect was closely mirrored with respect to the total α-cell area **(D)**. With respect to the β-cell area of the 2 and 4 m.o. WT or GIT2KO islets there was a non-significant trend for a reduction in the GIT2KO mice **(E)**. Upon inspection of the pattern of islet size distribution at both 2 and 4 m.o. there seemed to be minimal differences between WT and GIT2KO mice **(F)**.

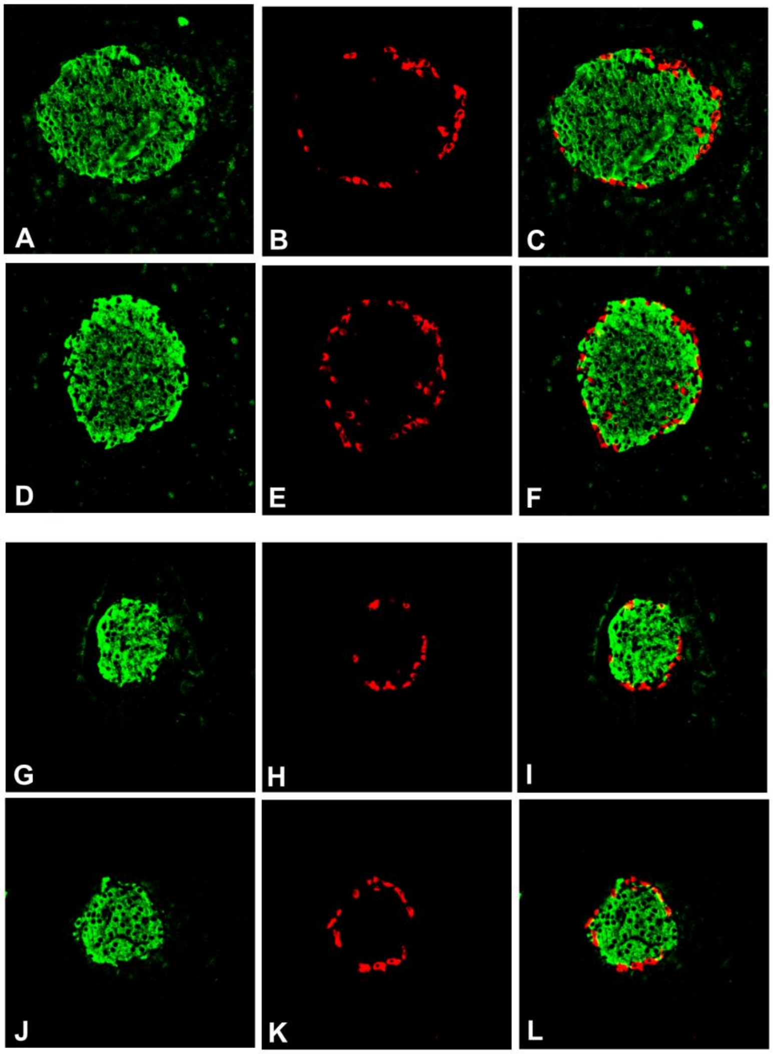

Figure S3. Functional beta and alpha cell structure of 2 or 8 month old GIT2KO mice islets. (A) Insulin AF488, glucagon AF568 **(B)** immunostaining of WT islet (4 m.o.). **(C)** Merge of panels **(A-B)**. **(D)** Insulin AF488, glucagon AF568 **(E)** immunostaining of GIT2KO islet (4 m.o.). **(F)** Merge of panels **(D-E)**. **(G)** Insulin AF488, glucagon AF568 **(H)** immunostaining of WT islet (2 m.o.). **(I)** Merge of panels **(G-H)**. **(J)** Insulin AF488, glucagon AF568 **(K)** immunostaining of GIT2KO islet (2 m.o.). **(L)** Merge of panels **(J-K)**.

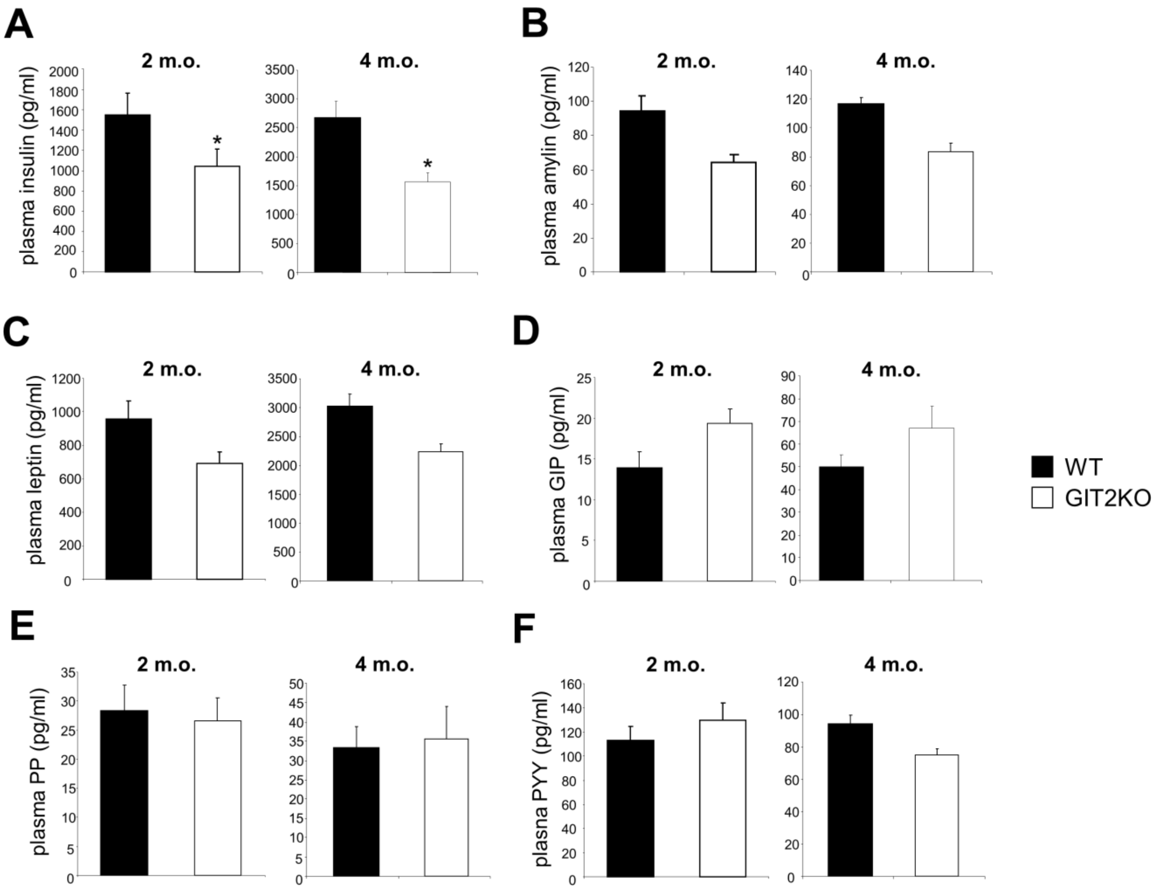

Figure S4. GIT2 genomic deletion affects multiple circulating energy-regulatory factors in 2 or 4 month old mice. Measurements of multiple circulating energy-regulatory factors were made in 2 or 4 month old (m.o.) WT (n=3, black bars) or GIT2KO (n=3, white bars) mice. **(A)** Significant reductions in circulating insulin levels were found in both 2 and 4 m.o. GIT2KO mice compared to WT controls. **(B)** Considerable but non-significant reductions in circulating amylin levels in GIT2KO mice compared to WT controls were noted at the 2 and 4 m.o. timepoints. **(C)** A trend for reduced levels of circulating leptin were noted at both 2 and 4 m.o. timepoints for GIT2KO mice compared to WT controls. **(D)** Both 2 and 4 m.o. GIT2KO mice demonstrate a trend for elevated circulating GIP levels compared to WT control mice. No significant differences at either the 2 or 4 m.o. timepoints between the GIT2KO and WT mice were noted for circulating levels of PP **(E)** or PYY **(F)**. Statistical significance is indicated in each figure panel as * $p \leq 0.05$; ** $p \leq 0.01$; *** $p \leq 0.001$.

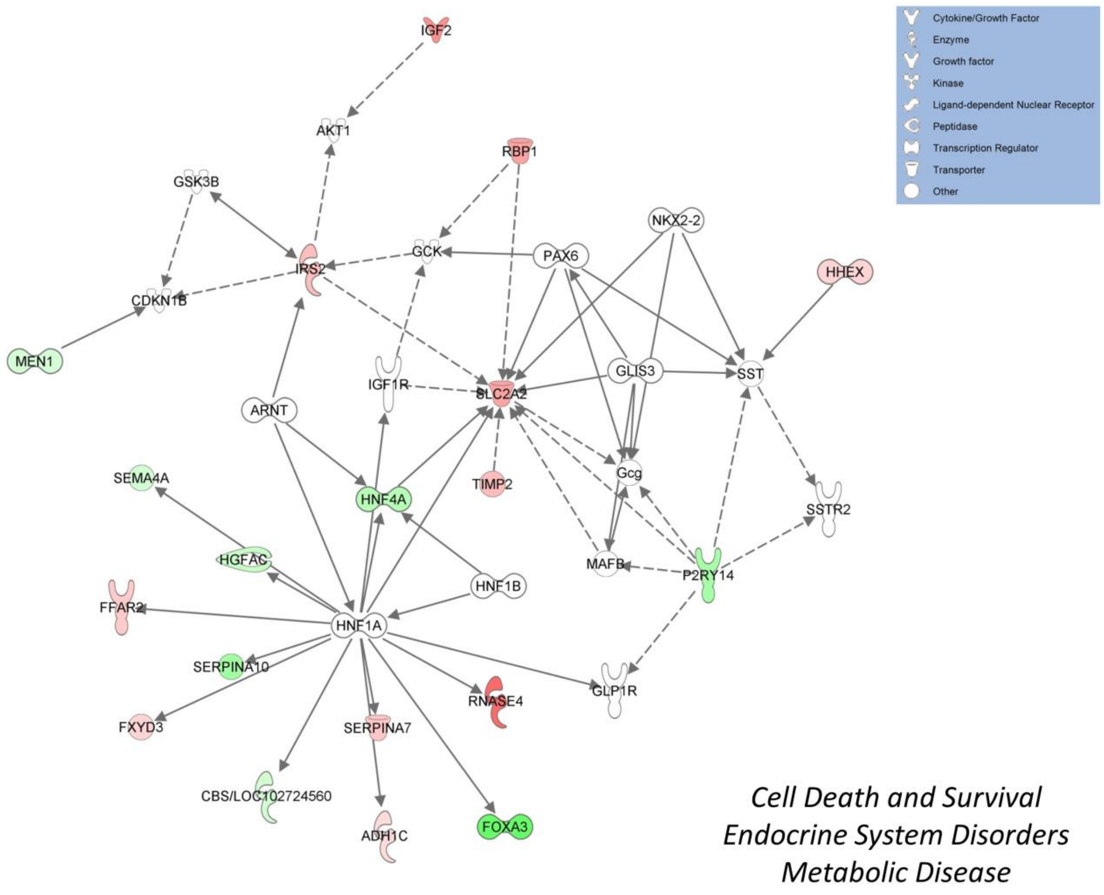

Figure S5. IPA-mediated functional signaling network analysis of pancreatic islet transcriptomic data. Significant transcriptomic data from isolated GIT2KO pancreatic islets was functionally annotated using the functional network analysis suite from IPA Pathways Analysis. The nature of the specific node components and their type of functional interaction (noted as arrows between factors) are described in the associated key. Red factors were upregulated in the input dataset while green factors were downregulated (GIT2KO versus WT). The IPA-generated textual description of the functional ramifications of the network are indicated in the lower right of the panel in italics. Networks in Figure S5 (Scl2a2-Hnf4a-Hnf1a focused) and Figure S6 (Pdx1-Ins1-Glis3 focused) were composed of diverse factors but possessed similar IPA textual definitions and priority enrichment scores.

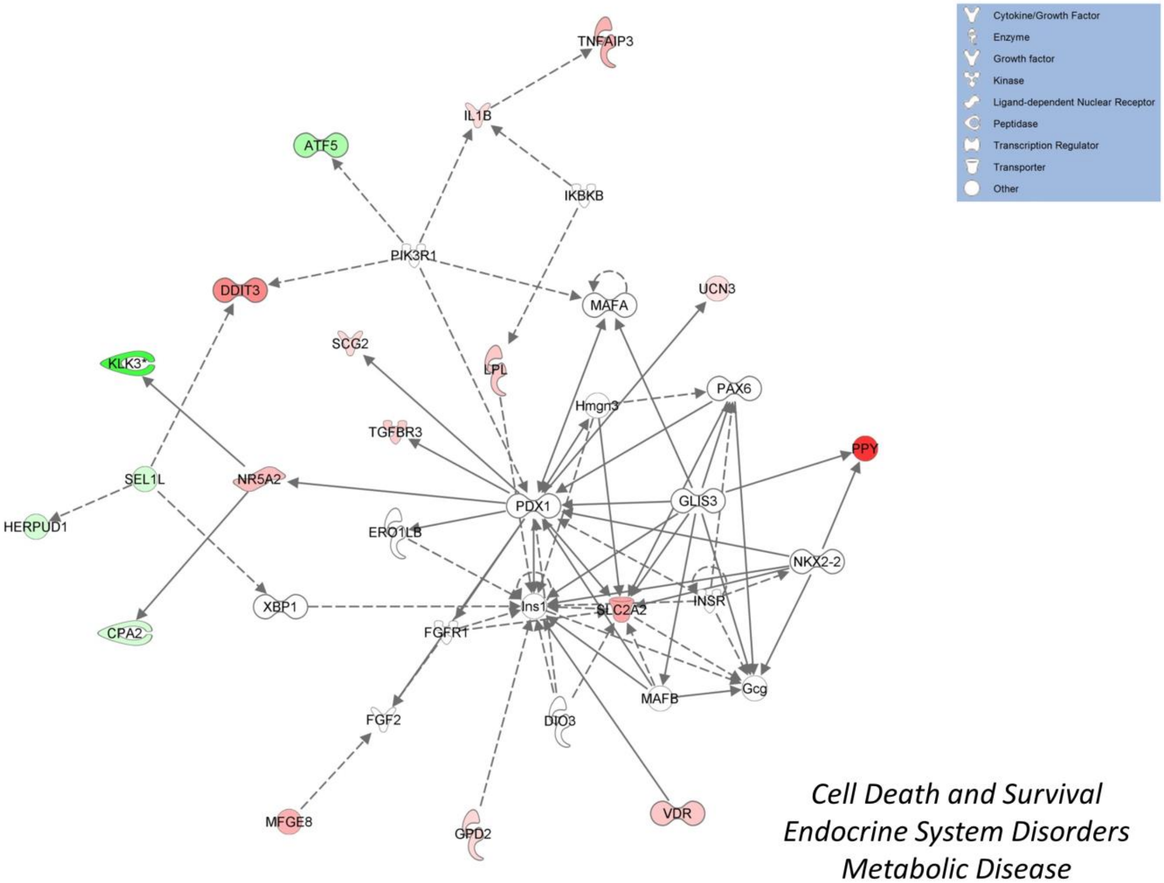

Figure S6. IPA-mediated functional signaling network analysis of pancreatic islet transcriptomic data. Significant transcriptomic data from isolated GIT2KO pancreatic islets was functionally annotated using the functional network analysis suite from IPA Pathways Analysis. The nature of the specific node components and their type of functional interaction (noted as arrows between factors) are described in the associated key. Red factors were upregulated in the input dataset while green factors were downregulated (GIT2KO versus WT). The IPA-generated textual description of the functional ramifications of the networks are indicated in the lower right of the panel in italics. Networks in Figure S5 (Scl2a2-Hnf4a-Hnf1a focused) and Figure S6 (Pdx1-Ins1-Glis3 focused) were composed of diverse factors but possessed similar IPA textual definitions and priority enrichment scores.

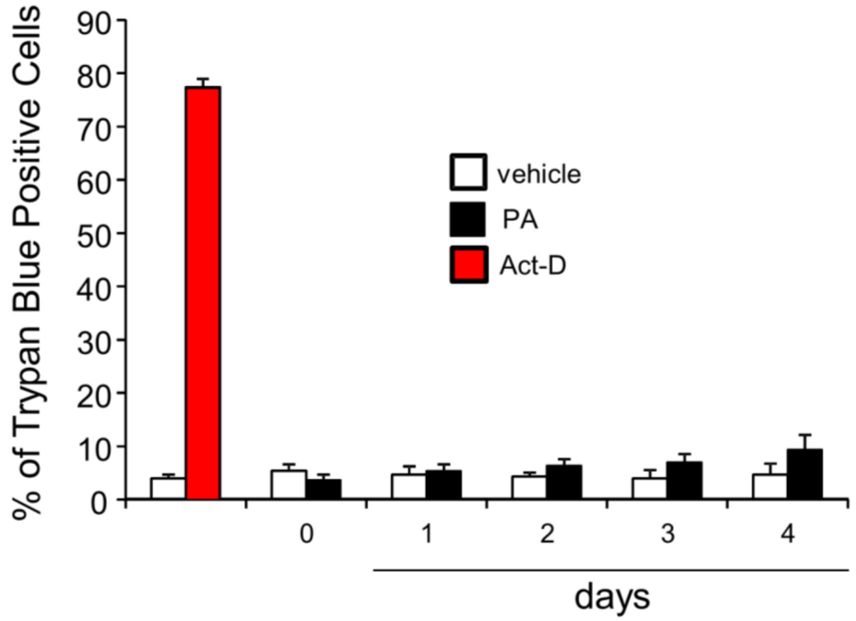

Figure S7. Cell survival analysis of palmitate treatment of murine TC-6 cells. The effects on TC-6 cell viability of extended palmitate (PA: 10µM), or vehicle, treatment times (1-4 days) were assessed using a standard Trypan Blue exclusion hemocytometer counting assay. The proportion of assayed cells exposed to palmitate for the specified time period demonstrating visually-measurable Trypan Blue cellular uptake is displayed. Exposure to the cytotoxic actinomycin-D (Act-D: 2µg/ml, 2hr) was used a positive control of visually-measurable Trypan Blue cellular uptake.

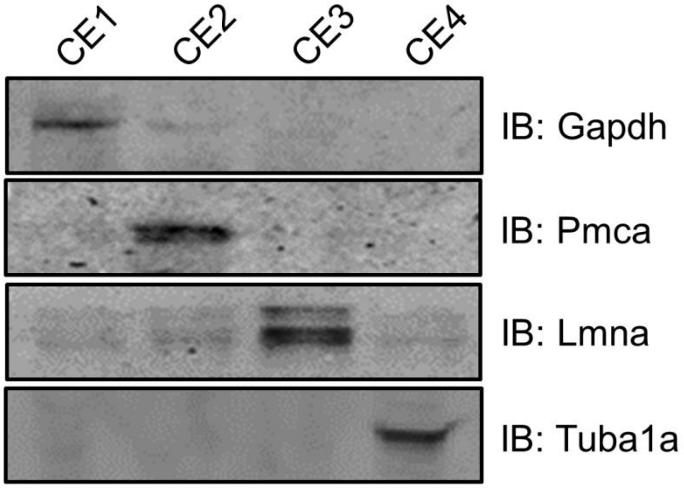

Figure S8. Western blotting verification of selectivity of differential detergent fractionation process. Murine TC-6 cells were differentially fractionated, using the Qiagen Qproteome® subcellular compartment extraction kit, into soluble fractions representing samples enriched for cytoplasmic proteins (referred to as the CE1 fraction), plasma membrane proteins (CE2 fraction), nuclear/large organelle proteins (CE3 fraction) and cytoskeletal proteins (CE4 fraction). Individual Western blots were performed for exemplar proteins from these four cellular compartments: Gapdh (glyceraldehyde 3-phosphate dehydrogenase) for CE1, Pmca (plasma membrane Ca^{2+} ATPase) for CE2, Lmna (lamin A) for CE3 and Tuba1a (tubulin alpha-1A chain) for CE4.

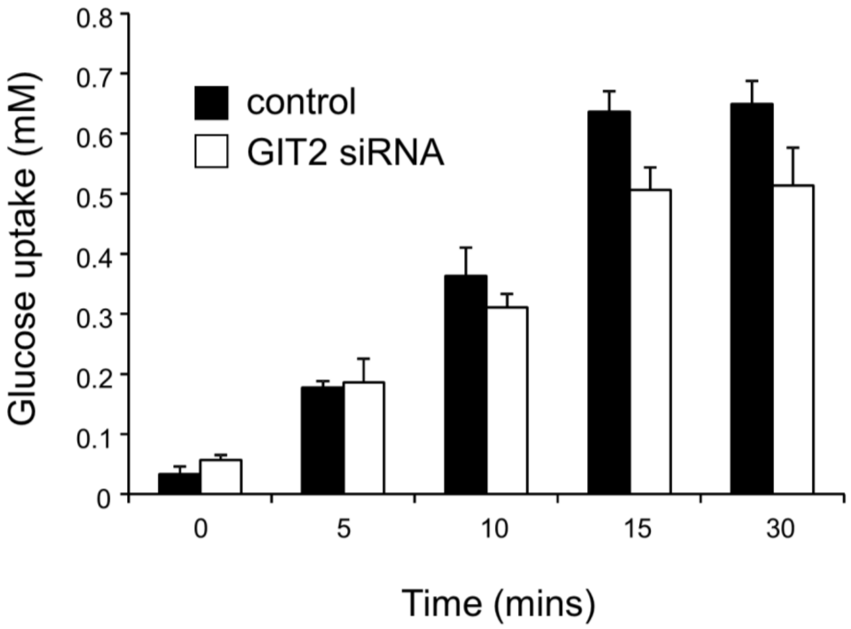

Figure S9. Cellular depletion of GIT2 partially attenuates responsive glucose uptake in murine TC-6 cells. siRNA-mediated (400nM siRNA exposure) depletion of GIT2 expression results in a non-significant, but consistent trend for the attenuation of bolus (5mM) glucose uptake over the experimental time period. Data from TC-6 cells exposed to a random scramble sequence siRNA (control) is represented by black bars while data for GIT2 siRNA-treated cells is indicated by white bars.

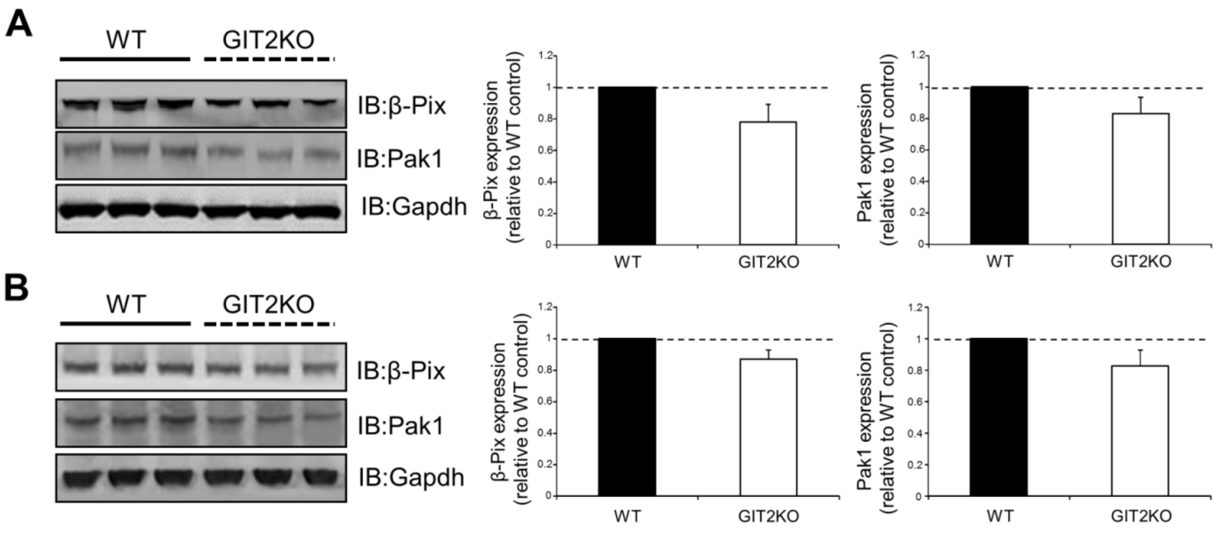

Figure S10. Genomic deletion of GIT2 results in reduced expression of GIT-associated signaling factors in hypothalamic and pancreatic tissues. Selective Western blotting for β-Pix (Rho guanine nucleotide exchange factor, Arhgef7) and Pak1 (p21-activated kinase 1) in hypothalamic **(A)** and pancreatic **(B)** tissue protein extracts for both WT (n=3, black bars) and GIT2KO (n=3, white bars) is depicted. In both tissues the GIT2KO mice (each western lane indicates protein from an individual mouse) demonstrate a moderate reduction in the expression of both β-Pix and Pak1.

II. Supplemental Tables

Table S1. Significantly-regulated transcripts differentially regulated in 2 month old GIT2KO mice compared to age-matched WT controls. For each significantly-regulated transcript the Official Gene Symbol, transcript description and expression Z ratio, GIT2KO versus WT are represented.

Gene Symbol	Description	Z ratio GIT2KO vs. WT
Cops8	COP9 (constitutive photomorphogenic) homolog, subunit 8 (Arabidopsis thaliana) (Cops8)	21.7533899
Slc7a14	solute carrier family 7 (cationic amino acid transporter, y+ system), member 14 (Slc7a14)	14.38411663
Golga2	golgi autoantigen, golgin subfamily a, 2 (Golga2), transcript variant 1	9.714595114
B3galt6	UDP-Gal:betaGal beta 1,3-galactosyltransferase, polypeptide 6 (B3galt6)	9.14228518
Dusp7	dual specificity phosphatase 7 (Dusp7)	8.506051606
Bsdc1	BSD domain containing 1 (Bsdc1)	6.629895458
Deadc1	deaminase domain containing 1 (Deadc1)	6.576669375
Insig2	insulin induced gene 2 (Insig2)	6.298551298
D14Ertd449e	DNA segment, Chr 14, ERATO Doi 449, expressed (D14Ertd449e)	6.226693483
Ccrn4l	CCR4 carbon catabolite repression 4-like (S. cerevisiae) (Ccrn4l)	5.92349167
Vps33a	vacuolar protein sorting 33A (yeast) (Vps33a)	5.831074837
Pkm2	pyruvate kinase, muscle (Pkm2)	5.818120798
Rapgefl1	Rap guanine nucleotide exchange factor (GEF)-like 1 (Rapgefl1)	5.67490535
Tomm22	translocase of outer mitochondrial membrane 22 homolog (yeast) (Tomm22), nuclear gene encoding mitochondrial protein	5.56971343
LOC545056	ubiquitin-conjugating enzyme E2, J2 homolog pseudogene (LOC545056) on chromosome 14.	5.066069279
Rgl1	ral guanine nucleotide dissociation stimulator,-like 1 (Rgl1)	5.033851983
Rpl29	ribosomal protein L29 (Rpl29)	4.588612189
Xkr8	X Kell blood group precursor related family member 8 homolog (Xkr8)	4.571088556
Atf4	activating transcription factor 4 (Atf4)	4.478640972
Klhl21	kelch-like 21 (Drosophila) (Klhl21)	4.334842582
Cryab	crystallin, alpha B (Cryab)	4.006436345
Mgat3	mannoside acetylglucosaminyltransferase 3 (Mgat3)	3.897647064
Agxt2l1	alanine-glyoxylate aminotransferase 2-like 1 (Agxt2l1)	3.868793187
Rbm28	RNA binding motif protein 28 (Rbm28), transcript variant 2	3.830841402
Lbh	limb-bud and heart (Lbh)	3.77225784
Agxt2l1	alanine-glyoxylate aminotransferase 2-like 1 (Agxt2l1)	3.734550716
LOC100046039	similar to histone deacetylase HD1 (LOC100046039)	3.659756212
Snapc3	small nuclear RNA activating complex, polypeptide 3 (Snapc3)	3.597438571
Ttyh3	tweety homolog 3 (Drosophila) (Ttyh3)	3.566407086
Zfp295	zinc finger protein 295 (Zfp295), transcript variant 3	3.559186375
Ppm1m	protein phosphatase 1M (Ppm1m), transcript variant 1	3.374742822
Efhd1	EF hand domain containing 1 (Efhd1)	3.267196454
Stk25	serine/threonine kinase 25 (yeast) (Stk25)	3.209869167
Map1lc3a	microtubule-associated protein 1 light chain 3 alpha (Map1lc3a)	3.117534242
Sin3a	transcriptional regulator, SIN3A (yeast) (Sin3a)	3.049142052
Tubb4	tubulin, beta 4 (Tubb4)	2.999884002
Fndc5	fibronectin type III domain containing 5 (Fndc5)	2.980719203
Sepw1	selenoprotein W, muscle 1 (Sepw1)	2.980180647
AI836003	expressed sequence AI836003 (AI836003)	2.689241699

Dpp10	dipeptidylpeptidase 10 (Dpp10)	2.687971674
Stk25	serine/threonine kinase 25 (yeast) (Stk25)	2.675274135
Mrpl48	mitochondrial ribosomal protein L48 (Mrpl48), transcript variant 1	2.675022782
Rph3a	rabphilin 3A (Rph3a)	2.667478344
Med8	mediator of RNA polymerase II transcription, subunit 8 homolog (yeast) (Med8), transcript variant 1	2.641335829
BC055107	cDNA sequence BC055107 (BC055107)	2.595996264
Sytl2	synaptotagmin-like 2 (Sytl2), transcript variant 5	2.573918966
Ugp2	UDP-glucose pyrophosphorylase 2 (Ugp2)	2.571811096
Scmh1	sex comb on midleg homolog 1 (Scmh1)	2.537104385
Pcsk2	proprotein convertase subtilisin/kexin type 2 (Pcsk2)	2.520289439
Syp	synaptophysin (Syp)	2.517408451
Elfn2	leucine rich repeat and fibronectin type III, extracellular 2 (Elfn2)	2.501509046
Cnp	2',3'-cyclic nucleotide 3' phosphodiesterase (Cnp)	2.480234536
Ddo	D-aspartate oxidase (Ddo)	2.474310551
Elovl4	elongation of very long chain fatty acids (FEN1/Elo2, SUR4/Elo3, yeast)-like 4 (Elovl4)	2.454457132
Pde1b	phosphodiesterase 1B, Ca2+-calmodulin dependent (Pde1b)	2.45427638
Lix1	limb expression 1 homolog (chicken) (Lix1)	2.454041883
Il33	interleukin 33 (Il33)	2.429250713
6430510M02Rik	RIKEN cDNA 6430510M02 gene (6430510M02Rik)	2.416698129
Gprc5b	G protein-coupled receptor, family C, group 5, member B (Gprc5b)	2.40309449
Gls2	glutaminase 2 (liver, mitochondrial) (Gls2)	2.38627134
Acat2	acetyl-Coenzyme A acetyltransferase 2 (Acat2)	2.383958264
Acpl2	acid phosphatase-like 2 (Acpl2)	2.381552993
Fbxo44	F-box protein 44 (Fbxo44)	2.37614798
Mkks	McKusick-Kaufman syndrome protein (Mkks)	2.331438099
Cidea	cell death-inducing DNA fragmentation factor, alpha subunit-like effector A (Cidea)	2.326795219
Tmem108	transmembrane protein 108 (Tmem108)	2.322053829
Drbp1	RNA binding motif protein 45 (Drbp1)	2.320355156
Spire1	spire homolog 1 (Drosophila) (Spire1), transcript variant 2	2.316088779
Ostm1	osteopetrosis associated transmembrane protein 1 (Ostm1)	2.313222796
6330442E10Rik	RIKEN cDNA 6330442E10 gene (6330442E10Rik)	2.312230403
Dlst	dihydrolipoamide S-succinyltransferase (E2 component of 2-oxo-glutarate complex) (Dlst)	2.303587553
Ppargc1a	peroxisome proliferative activated receptor, gamma, coactivator 1 alpha (Ppargc1a)	2.295452879
Zdhhc21	zinc finger, DHHC domain containing 21 (Zdhhc21)	2.294118078
Atg16l1	autophagy-related 16-like 1 (yeast) (Atg16l1), transcript variant b	2.29407346
Eif4ebp2	eukaryotic translation initiation factor 4E binding protein 2 (Eif4ebp2)	2.289396462
Syn1	synapsin I (Syn1)	2.271252059
Zmat4	zinc finger, matrin type 4 (Zmat4)	2.252075702
Cxcl14	chemokine (C-X-C motif) ligand 14 (Cxcl14)	2.240235396
Cmtm5	CKLF-like MARVEL transmembrane domain containing 5 (Cmtm5)	2.221043912
Darc	Duffy blood group, chemokine receptor (Darc)	2.220793441
Ppp1r8	protein phosphatase 1, regulatory (inhibitor) subunit 8 (Ppp1r8)	2.203599207
Hmga1	high mobility group AT-hook 1 (Hmga1), transcript variant 1	2.196843586
Celsr2	cadherin, EGF LAG seven-pass G-type receptor 2 (flamingo homolog, Drosophila) (Celsr2), transcript variant 1	2.180135609
Bcl2l1	Bcl2-like 1 (Bcl2l1), nuclear gene encoding mitochondrial protein	2.153697238
Rbbp5	retinoblastoma binding protein 5 (Rbbp5)	2.15162227
Spock1	sparc/osteonectin, cwcv and kazal-like domains proteoglycan 1 (Spock1)	2.145920654
Ccdc127	coiled-coil domain containing 127 (Ccdc127)	2.145219676

Mmab	methylmalonic aciduria (cobalamin deficiency) type B homolog (human) (Mmab), nuclear gene encoding mitochondrial protein	2.125299947
Psmb7	proteasome (prosome, macropain) subunit, beta type 7 (Psmb7)	2.124556577
Iqsec3	IQ motif and Sec7 domain 3 (Iqsec3)	2.121826458
Tmem127	transmembrane protein 127 (Tmem127)	2.119976487
Hapln4	hyaluronan and proteoglycan link protein 4 (Hapln4)	2.100286342
Asna1	arsA (bacterial) arsenite transporter, ATP-binding, homolog 1 (Asna1)	2.094259076
Fgf13	fibroblast growth factor 13 (Fgf13)	2.087181927
Akap6	A kinase (PRKA) anchor protein 6 (Akap6)	2.083578876
Bzrap1	benzodiazapine receptor associated protein 1 (Bzrap1)	2.080308045
Kifc2	kinesin family member C2 (Kifc2)	2.064173779
Slc6a11	solute carrier family 6 (neurotransmitter transporter, GABA), member 11 (Slc6a11)	2.047163141
2310005E10Rik	RIKEN cDNA 2310005E10 gene (2310005E10Rik)	2.0395108
Ppid	peptidylprolyl isomerase D (cyclophilin D) (Ppid)	2.036872407
LOC100044576	hypothetical protein LOC100044576 (LOC100044576)	2.036786202
Trappc2l	trafficking protein particle complex 2-like (Trappc2l)	2.030752949
Prickle4	prickle homolog 4 (Drosophila) (Prickle4)	2.024644024
Ensa	endosulfine alpha (Ensa), transcript variant 1	2.022929656
Cnot4	CCR4-NOT transcription complex, subunit 4 (Cnot4)	2.018973904
Lonrf2	LON peptidase N-terminal domain and ring finger 2 (Lonrf2)	2.016238428
Coq5	coenzyme Q5 homolog, methyltransferase (yeast) (Coq5)	2.008231116
Phf13	PHD finger protein 13 (Phf13)	2.007697012
BC003885	cDNA sequence BC003885 (BC003885)	2.00623144
Mecp2	methyl CpG binding protein 2 (Mecp2), transcript variant 1	1.998678585
Tipin	timeless interacting protein (Tipin)	1.984481955
Vti1a	vesicle transport through interaction with t-SNAREs homolog 1A (yeast) (Vti1a)	1.979949291
Itfg2	integrin alpha FG-GAP repeat containing 2 (Itfg2)	1.978705942
Myo9a	myosin IXa, transcript variant 2 (Myo9a)	1.969915194
Copg	coatomer protein complex, subunit gamma (Copg), transcript variant 2	1.963262095
Itgb1bp1	integrin beta 1 binding protein 1 (Itgb1bp1)	1.959838749
6-Sep	septin 6 (Sept6)	1.956469572
Pex19	peroxisome biogenesis factor 19 (Pex19)	1.952471323
Rassf2	Ras association (RalGDS/AF-6) domain family member 2 (Rassf2)	1.949103344
Nav1	neuron navigator 1 (Nav1)	1.939233954
Cds1	CDP-diacylglycerol synthase 1 (Cds1)	1.932101383
Fgf1	fibroblast growth factor 1 (Fgf1)	1.921521691
Nav1	neuron navigator 1 (Nav1)	1.917361042
Igsf3	immunoglobulin superfamily, member 3 (Igsf3)	1.913030409
4930572J05Rik	RIKEN cDNA 4930572J05 gene (4930572J05Rik)	1.907046883
Snca	synuclein, alpha (Snca), transcript variant 2	1.892823515
Slc15a4	solute carrier family 15, member 4 (Slc15a4)	1.887648812
Ttc14	tetratricopeptide repeat domain 14 (Ttc14)	1.877849253
Fn3k	fructosamine 3 kinase (Fn3k), transcript variant 1	1.875958691
Ncald	neurocalcin delta (Ncald)	1.875180337
Ndrg2	N-myc downstream regulated gene 2 (Ndrg2)	1.872272182
LOC100046457	similar to Cltb protein (LOC100046457), misc RNA.	1.862217307
Amigo1	adhesion molecule with Ig like domain 1 (Amigo1)	1.852547941
LOC100047659	hypothetical protein LOC100047659 (LOC100047659)	1.850400702
Ralyl	RALY RNA binding protein-like (Ralyl)	1.847025459

6-Sep	septin 6 (Sept6)	1.839151886
Chmp2a	chromatin modifying protein 2A (Chmp2a)	1.838281371
1300001I01Rik	RIKEN cDNA 1300001I01 gene (1300001I01Rik)	1.835052651
Ppm1b	protein phosphatase 1B, magnesium dependent, beta isoform (Ppm1b)	1.83083589
3-Sep	septin 3 (Sept3)	1.830222847
BC050811	cDNA sequence BC050811 (BC050811)	1.828176841
Mrpl48	mitochondrial ribosomal protein L48 (Mrpl48), nuclear gene encoding mitochondrial protein	1.824429541
Rassf5	Ras association (RalGDS/AF-6) domain family 5 (Rassf5)	1.817553729
Bag3	Bcl2-associated athanogene 3 (Bag3)	1.812467838
Rnasen	ribonuclease III, nuclear (Rnasen)	1.802345386
D0H4S114	DNA segment, human D4S114 (D0H4S114)	1.800412524
Copg	coatomer protein complex, subunit gamma 1 (Copg1)	1.796797164
Ppm1b	protein phosphatase 1B, magnesium dependent, beta isoform (Ppm1b)	1.7953273
Prpf19	PRP19/PSO4 pre-mRNA processing factor 19 homolog (S. cerevisiae) (Prpf19)	1.794038779
Pcdh20	protocadherin 20 (Pcdh20)	1.785319105
Cadps	Ca2+-dependent secretion activator (Cadps), transcript variant 2	1.784408994
Eif2ak1	eukaryotic translation initiation factor 2 alpha kinase 1 (Eif2ak1)	1.783581815
Pigt	phosphatidylinositol glycan anchor biosynthesis, class T (Pigt)	1.77320226
Mapk6	mitogen-activated protein kinase 6 (Mapk6), transcript variant 2	1.77067065
Slc27a1	solute carrier family 27 (fatty acid transporter), member 1 (Slc27a1)	1.768216282
Ank2	ankyrin 2, brain (Ank2), transcript variant 3	1.767155243
Kcnk1	potassium channel, subfamily K, member 1 (Kcnk1)	1.749865467
Nrsn2	neurensin 2 (Nrsn2)	1.748901642
Gls2	glutaminase 2 (liver, mitochondrial) (Gls2), nuclear gene encoding mitochondrial protein	1.742047881
Ppp1r3f	protein phosphatase 1, regulatory (inhibitor) subunit 3F (Ppp1r3f)	1.733537037
Ppp1r3c	protein phosphatase 1, regulatory (inhibitor) subunit 3C (Ppp1r3c)	1.732784875
Srxn1	sulfiredoxin 1 homolog (S. cerevisiae) (Srxn1)	1.73107976
Chrnb2	cholinergic receptor, nicotinic, beta polypeptide 2 (neuronal) (Chrnb2)	1.725656596
Mthfd1	methylenetetrahydrofolate dehydrogenase (NADP+ dependent), methenyltetrahydrofolate cyclohydrolase, formyltetrahydrofolate synthase (Mthfd1)	1.722358593
Hsd11b1	hydroxysteroid 11-beta dehydrogenase 1 (Hsd11b1)	1.718116646
Rcan2	regulator of calcineurin 2 (Rcan2), transcript variant 1	1.71381777
Spire2	spire homolog 2 (Drosophila) (Spire2)	1.710210932
LOC433886	hypothetical gene supported by AK049058; BC025881 (LOC433886)	1.709280298
Chd5	chromodomain helicase DNA binding protein 5 (Chd5)	1.703357279
Pctk1	PCTAIRE-motif protein kinase 1 (Pctk1)	1.700374822
Reep5	receptor accessory protein 5 (Reep5)	1.699126229
Armcx3	armadillo repeat containing, X-linked 3 (Armcx3)	1.696792791
Olfm1	olfactomedin 1 (Olfm1), transcript variant 1	1.688339666
Caskin1	CASK interacting protein 1 (Caskin1)	1.682215622
Gstz1	glutathione transferase zeta 1 (maleylacetoacetate isomerase) (Gstz1)	1.681037073
Arf3	ADP-ribosylation factor 3 (Arf3)	1.677546766
Fuk	fucokinase (Fuk), transcript variant 2	1.657726567
Dync1li2	dynein, cytoplasmic 1 light intermediate chain 2 (Dync1li2)	1.657564381
Cry2	cryptochrome 2 (photolyase-like) (Cry2)	1.656698961
Mrm1	mitochondrial rRNA methyltransferase 1 homolog (S. cerevisiae) (Mrm1), nuclear gene encoding mitochondrial protein	1.646885002
Tmem62	transmembrane protein 62 (Tmem62)	1.642389322
Slc4a3	solute carrier family 4 (anion exchanger), member 3 (Slc4a3)	1.6329134
Smap2	stromal membrane-associated GTPase-activating protein 2 (Smap2)	1.631377142

Copa	coatomer protein complex subunit alpha (Copa)	1.628975681
Pitpnm2	phosphatidylinositol transfer protein, membrane-associated 2 (Pitpnm2)	1.622606659
Zfyve20	zinc finger, FYVE domain containing 20 (Zfyve20)	1.596104705
Slc2a3	solute carrier family 2 (facilitated glucose transporter), member 3 (Slc2a3)	1.593847894
B230217C12Rik	RIKEN cDNA B230217C12 gene (B230217C12Rik)	1.584490158
5330431N19Rik	RIKEN cDNA 5330431N19 gene (5330431N19Rik)	1.583835551
Adora1	adenosine A1 receptor (Adora1), transcript variant 1	1.583359792
Sez6	seizure related gene 6 (Sez6)	1.580075433
Hspa9	heat shock protein 9 (Hspa9)	1.575986655
Ric3	resistance to inhibitors of cholinesterase 3 homolog (C. elegans) (Ric3), transcript variant 1	1.574828697
Fez2	fasciculation and elongation protein zeta 2 (zygin II) (Fez2)	1.573025153
1810007P19Rik	RIKEN cDNA 1810007P19 gene (1810007P19Rik)	1.571741657
Pdcl	phosducin-like (Pdcl)	1.570901777
Sumf2	sulfatase modifying factor 2 (Sumf2)	1.560594629
Cdv3	carnitine deficiency-associated gene expressed in ventricle 3 (Cdv3), transcript variant CDV3A	1.557295624
Lsm6	LSM6 homolog, U6 small nuclear RNA associated (S. cerevisiae) (Lsm6)	1.554144054
Slc12a6	solute carrier family 12, member 6 (Slc12a6), transcript variant 1	1.552043163
BC003266	cDNA sequence BC003266 (BC003266)	1.540966139
Cntn6	contactin 6 (Cntn6)	1.533851358
Ercc4	excision repair cross-complementing rodent repair deficiency, complementation group 4 (Ercc4)	1.524233629
1700084C01Rik	RIKEN cDNA 1700084C01 gene (1700084C01Rik)	1.522127889
Ldoc1l	leucine zipper, down-regulated in cancer 1-like (Ldoc1l)	1.517700169
1700084C01Rik	RIKEN cDNA 1700084C01 gene (1700084C01Rik)	1.511006498
D6Wsu176e	family with sequence similarity 3, member C (Fam3c)	1.50253436
Ap2a1	adaptor protein complex AP-2, alpha 1 subunit (Ap2a1), transcript variant 1	1.501405151
Hnrpdl	heterogeneous nuclear ribonucleoprotein D-like (Hnrpdl)	-1.50584685
Prdx4	peroxiredoxin 4 (Prdx4)	-1.508936301
Gtf2h4	general transcription factor II H, polypeptide 4 (Gtf2h4)	-1.515598797
EG545056	predicted gene, EG545056 (EG545056) on chromosome 14.	-1.516959796
Ncl	nucleolin (Ncl)	-1.519554441
Dhx57	DEAH (Asp-Glu-Ala-Asp/His) box polypeptide 57 (Dhx57)	-1.520202056
Dtx2	deltex 2 homolog (Drosophila) (Dtx2)	-1.525749661
Tmem192	transmembrane protein 192 (Tmem192)	-1.528991622
Dmap1	DNA methyltransferase 1-associated protein 1 (Dmap1)	-1.52997468
Tsfm	Ts translation elongation factor, mitochondrial (Tsfm), nuclear gene encoding mitochondrial protein	-1.541812723
Ccrn4l	CCR4 carbon catabolite repression 4-like (S. cerevisiae) (Ccrn4l)	-1.541943277
Brunol4	bruno-like 4, RNA binding protein (Drosophila) (Brunol4)	-1.542319506
Surf4	surfeit gene 4 (Surf4)	-1.548129449
Gnaz	guanine nucleotide binding protein, alpha z subunit (Gnaz)	-1.551970647
Rabl5	RAB, member RAS oncogene family-like 5 (Rabl5)	-1.567462781
Edg2	endothelial differentiation, lysophosphatidic acid G-protein-coupled receptor, 2 (Edg2)	-1.567895241
2700038C09Rik	RIKEN cDNA 2700038C09 gene (2700038C09Rik)	-1.567949579
1200003C05Rik	RIKEN cDNA 1200003C05 gene (1200003C05Rik)	-1.592406971
Idua	iduronidase, alpha-L- (Idua)	-1.596487407
Mbp	myelin basic protein (Mbp), transcript variant 7	-1.598666112
Notch4	Notch gene homolog 4 (Drosophila) (Notch4)	-1.604074638
Atf2	activating transcription factor 2 (Atf2), transcript variant 1	-1.60457857
LOC100046746	similar to SMAP1 (LOC100046746)	-1.605290185

Eif3k	eukaryotic translation initiation factor 3, subunit K (Eif3k)	-1.606850788
2700038C09Rik	RIKEN cDNA 2700038C09 gene (2700038C09Rik)	-1.606984414
Rnpep	arginyl aminopeptidase (aminopeptidase B) (Rnpep)	-1.610612041
Yipf3	Yip1 domain family, member 3 (Yipf3)	-1.611861952
Acta1	actin, alpha 1, skeletal muscle (Acta1)	-1.614098906
Arpc5	actin related protein 2/3 complex, subunit 5 (Arpc5)	-1.615341325
5730449L18Rik	RIKEN cDNA 5730449L18 gene (5730449L18Rik)	-1.617826649
BC055368	tubulin tyrosine ligase-like family, member 12 (Ttll12)	-1.621233877
Sfrs2	splicing factor, arginine/serine-rich 2 (SC-35) (Sfrs2)	-1.623721831
Pex14	peroxisomal biogenesis factor 14 (Pex14)	-1.628956026
Rnuxa	RNA U, small nuclear RNA export adaptor (Rnuxa)	-1.638284196
B4galt3	UDP-Gal:betaGlcNAc beta 1,4-galactosyltransferase, polypeptide 3 (B4galt3)	-1.653927077
5730410E15Rik	syntabulin (syntaxin-interacting) (Sybu)	-1.65438215
1110036O03Rik	RIKEN cDNA 1110036O03 gene (1110036O03Rik)	-1.654686617
Vkorc1	vitamin K epoxide reductase complex, subunit 1 (Vkorc1)	-1.656338762
Myst4	MYST histone acetyltransferase monocytic leukemia 4 (Myst4)	-1.657935459
Pex7	peroxisome biogenesis factor 7 (Pex7)	-1.66757101
Add3	adducin 3 (gamma) (Add3)	-1.673283043
Mtap7	microtubule-associated protein 7 (Mtap7)	-1.675878468
Lrp10	low-density lipoprotein receptor-related protein 10 (Lrp10)	-1.680631551
Abcb9	ATP-binding cassette, sub-family B (MDR/TAP), member 9 (Abcb9)	-1.690688356
Ergic2	ERGIC and golgi 2 (Ergic2), transcript variant 1	-1.703110751
Lrfn2	leucine rich repeat and fibronectin type III domain containing 2 (Lrfn2)	-1.706851524
2310036D22Rik	transmembrane protein 106B (Tmem106b)	-1.707522028
Crebl1	cAMP responsive element binding protein-like 1 (Crebl1)	-1.708521226
Nedd4l	neural precursor cell expressed, developmentally down-regulated gene 4-like (Nedd4l)	-1.712729372
Slc23a2	solute carrier family 23 (nucleobase transporters), member 2 (Slc23a2)	-1.71515612
Add3	adducin 3 (gamma) (Add3)	-1.718099066
2010007H12Rik	RIKEN cDNA 2010007H12 gene (2010007H12Rik)	-1.730281065
Mrps10	mitochondrial ribosomal protein S10 (Mrps10), nuclear gene encoding mitochondrial protein	-1.7382358
Clptm1	cleft lip and palate associated transmembrane protein 1 (Clptm1)	-1.742016893
Rap2a	RAS related protein 2a (Rap2a)	-1.746241794
Stard10	START domain containing 10 (Stard10)	-1.755140352
Twsg1	twisted gastrulation homolog 1 (Drosophila) (Twsg1)	-1.756246056
Rnaset2b	ribonuclease T2B (Rnaset2b)	-1.766972453
Cebpb	CCAAT/enhancer binding protein (C/EBP), beta (Cebpb)	-1.769034293
Grip1	glutamate receptor interacting protein 1 (Grip1), transcript variant 2	-1.770983288
Tnfaip1	tumor necrosis factor, alpha-induced protein 1 (endothelial) (Tnfaip1)	-1.775349739
Fbxl15	F-box and leucine-rich repeat protein 15 (Fbxl15)	-1.777014047
Triobp	TRIO and F-actin binding protein (Triobp), transcript variant 3	-1.784993984
Setd1b	SET domain containing 1B (Setd1b)	-1.785160296
LOC100047323	similar to ataxin 2 (LOC100047323), misc RNA.	-1.785629939
Serpinf1	serine (or cysteine) peptidase inhibitor, clade F, member 1 (Serpinf1)	-1.801198529
0710008K08Rik	RIKEN cDNA 0710008K08 gene (0710008K08Rik)	-1.804591501
Cog8	component of oligomeric golgi complex 8 (Cog8)	-1.813900625
Hist1h2bm	histone cluster 1, H2bm (Hist1h2bm)	-1.834158309
Ext2	exostoses (multiple) 2 (Ext2)	-1.837493621
Negr1	neuronal growth regulator 1 (Negr1), transcript variant 1	-1.853093645

Igsf9	immunoglobulin superfamily, member 9 (Igsf9)	-1.858888266
Pde6d	phosphodiesterase 6D, cGMP-specific, rod, delta (Pde6d)	-1.8989533
Hist1h2bk	histone cluster 1, H2bk (Hist1h2bk)	-1.90867353
Nudc	nuclear distribution gene C homolog (Aspergillus) (Nudc)	-1.914152616
Prlr	prolactin receptor (Prlr)	-1.920411604
Atf5	activating transcription factor 5 (Atf5)	-1.926706418
LOC546015	similar to ribosomal protein S9 (LOC546015), misc RNA.	-1.926725806
Hist1h2bc	histone cluster 1, H2bc (Hist1h2bc)	-1.956014556
Cno	cappuccino (Cno)	-1.966933455
Pdgfa	platelet derived growth factor, alpha (Pdgfa)	-1.968019571
Mgat4b	mannoside acetylglucosaminyltransferase 4, isoenzyme B (Mgat4b)	-1.977091268
Id4	inhibitor of DNA binding 4 (Id4)	-1.978780801
Hexb	hexosaminidase B (Hexb)	-1.999815627
Rims2	regulating synaptic membrane exocytosis 2 (Rims2)	-2.002836788
Zmpste24	zinc metallopeptidase, STE24 homolog (S. cerevisiae) (Zmpste24)	-2.008474061
Tekt1	tektin 1 (Tekt1)	-2.008918868
2310037I24Rik	RIKEN cDNA 2310037I24 gene (2310037I24Rik)	-2.017151435
Tmem118	transmembrane protein 118 (Tmem118)	-2.022357833
9530068E07Rik	RIKEN cDNA 9530068E07 gene (9530068E07Rik)	-2.02241505
Ccdc90b	coiled-coil domain containing 90B (Ccdc90b)	-2.031366099
Triobp	TRIO and F-actin binding protein (Triobp)	-2.041457031
Tnrc6a	trinucleotide repeat containing 6a (Tnrc6a)	-2.042433898
D3Bwg0562e	DNA segment, Chr 3, Brigham & Women's Genetics 0562 expressed (D3Bwg0562e)	-2.044449442
Dedd2	death effector domain-containing DNA binding protein 2 (Dedd2)	-2.066498049
Commd3	COMM domain containing 3 (Commd3)	-2.081580979
Mmp17	matrix metallopeptidase 17 (Mmp17)	-2.08246481
Gorasp1	golgi reassembly stacking protein 1 (Gorasp1)	-2.104749674
Pdpk1	3-phosphoinositide dependent protein kinase-1 (Pdpk1), transcript variant 2	-2.106195508
Sf3b5	splicing factor 3b, subunit 5 (Sf3b5)	-2.115484239
Alad	aminolevulinate, delta-, dehydratase (Alad)	-2.119400767
Rnaset2	ribonuclease T2 (Rnaset2), transcript variant 2	-2.124221942
1810015A11Rik	RIKEN cDNA 1810015A11 gene (1810015A11Rik)	-2.133675299
Nudt4	nudix (nucleoside diphosphate linked moiety X)-type motif 4 (Nudt4)	-2.136964303
Inppl1	inositol polyphosphate phosphatase-like 1 (Inppl1)	-2.145731109
Inppl1	inositol polyphosphate phosphatase-like 1 (Inppl1)	-2.172241653
Pdcd2	programmed cell death 2 (Pdcd2)	-2.19434634
Gpx1	glutathione peroxidase 1 (Gpx1)	-2.194608984
Prpf38b	PRP38 pre-mRNA processing factor 38 (yeast) domain containing B (Prpf38b)	-2.197868793
Pdyn	prodynorphin (Pdyn)	-2.209821951
Dag1	dystroglycan 1 (Dag1)	-2.219746302
Mrpl3	mitochondrial ribosomal protein L3 (Mrpl3), nuclear gene encoding mitochondrial protein	-2.221747711
Tex261	testis expressed gene 261 (Tex261)	-2.226856382
Imp3	IMP3, U3 small nucleolar ribonucleoprotein, homolog (yeast) (Imp3)	-2.232947726
Man2c1	mannosidase, alpha, class 2C, member 1 (Man2c1)	-2.239656651
Wtip	WT1-interacting protein (Wtip)	-2.240185994
Vdac2	voltage-dependent anion channel 2 (Vdac2)	-2.268795051
Kpna3	karyopherin (importin) alpha 3 (Kpna3)	-2.270734984
Pabpc1	poly A binding protein, cytoplasmic 1 (Pabpc1)	-2.273870447

Tcof1	Treacher Collins Franceschetti syndrome 1, homolog (Tcof1)	-2.284543557
Pdpk1	3-phosphoinositide dependent protein kinase 1 (Pdpk1)	-2.293003569
Phactr4	phosphatase and actin regulator 4 (Phactr4)	-2.307400385
Pcbp1	poly(rC) binding protein 1 (Pcbp1)	-2.328134803
Pdyn	prodynorphin (Pdyn)	-2.339451806
Ncl	nucleolin (Ncl)	-2.356972532
Ercc5	excision repair cross-complementing rodent repair deficiency, complementation group 5 (Ercc5)	-2.3661044
Prkg1	protein kinase, cGMP-dependent, type I (Prkg1), transcript variant 2	-2.367044223
Hmgcl	3-hydroxy-3-methylglutaryl-Coenzyme A lyase (Hmgcl)	-2.369685004
Pebp1	phosphatidylethanolamine binding protein 1 (Pebp1)	-2.382852804
Mdm2	transformed mouse 3T3 cell double minute 2 (Mdm2)	-2.383417272
Bcas2	breast carcinoma amplified sequence 2 (Bcas2)	-2.38578124
Gal3st1	galactose-3-O-sulfotransferase 1 (Gal3st1)	-2.386297341
Gp38	podoplanin (Pdpn)	-2.388576162
Slc2a1	solute carrier family 2 (facilitated glucose transporter), member 1 (Slc2a1)	-2.394387962
Phc2	polyhomeotic-like 2 (Drosophila) (Phc2)	-2.394793207
Arid2	AT rich interactive domain 2 (Arid-rfx like) (Arid2)	-2.401923212
Bcl11b	B-cell leukemia/lymphoma 11B (Bcl11b), transcript variant 2	-2.441326747
Pigp	phosphatidylinositol glycan anchor biosynthesis, class P (Pigp)	-2.453884822
1110008F13Rik	RIKEN cDNA 1110008F13 gene (1110008F13Rik)	-2.467992898
Bcl7a	B-cell CLL/lymphoma 7A (Bcl7a)	-2.486779313
Klf9	Kruppel-like factor 9 (Klf9)	-2.500354159
Gtf3a	general transcription factor III A (Gtf3a)	-2.501140471
Acy1	aminoacylase 1 (Acy1)	-2.529155161
Tagap	T-cell activation Rho GTPase-activating protein (Tagap)	-2.54973871
Dusp28	dual specificity phosphatase 28 (Dusp28)	-2.550088247
Cbr3	carbonyl reductase 3 (Cbr3)	-2.566691305
Ptprf	protein tyrosine phosphatase, receptor type, F (Ptprf)	-2.590007057
Glrx2	glutaredoxin 2 (thioltransferase) (Glrx2), transcript variant 1	-2.600419758
Rbbp9	retinoblastoma binding protein 9 (Rbbp9)	-2.617554032
Diablo	diablo homolog (Drosophila) (Diablo), nuclear gene encoding mitochondrial protein	-2.622859466
Mns1	meiosis-specific nuclear structural protein 1 (Mns1)	-2.644192562
BC008163	cDNA sequence BC008163 (BC008163)	-2.659523634
Pex6	peroxisomal biogenesis factor 6 (Pex6)	-2.661909246
Prr7	proline rich 7 (synaptic) (Prr7)	-2.693920385
Notch1	Notch gene homolog 1 (Drosophila) (Notch1)	-2.710424984
Anapc5	anaphase-promoting complex subunit 5 (Anapc5), transcript variant 1	-2.723921309
LOC100044779	similar to prothymosin alpha (LOC100044779), misc RNA.	-2.767787665
Med23	mediator complex subunit 23 (Med23)	-2.797018219
Dag1	dystroglycan 1 (Dag1)	-2.80388561
Pkig	protein kinase inhibitor, gamma (Pkig), transcript variant 3	-2.832632295
LOC677317	similar to Mod1 protein, transcript variant 4 (LOC677317)	-2.858763627
Mrps17	mitochondrial ribosomal protein S17 (Mrps17)	-2.908479398
Prune	prune homolog (Drosophila) (Prune)	-2.941360298
Itgb4	integrin beta 4 (Itgb4), transcript variant 2	-2.970915483
2410025L10Rik	RIKEN cDNA 2410025L10 gene (2410025L10Rik)	-2.988735113
Napepld	N-acyl phosphatidylethanolamine phospholipase D (Napepld)	-3.009384777
Nle1	notchless homolog 1 (Drosophila) (Nle1)	-3.084003963

Card10	caspase recruitment domain family, member 10 (Card10)	-3.089805501
2810432L12Rik	RIKEN cDNA 2810432L12 gene (2810432L12Rik)	-3.12311557
Scarf2	scavenger receptor class F, member 2 (Scarf2)	-3.146311285
Hyal2	hyaluronoglucosaminidase 2 (Hyal2)	-3.197256947
Sgsm1	small G protein signaling modulator 1 (Sgsm1)	-3.203684935
Rnf166	ring finger protein 166 (Rnf116)	-3.300315094
Ush2a	Usher syndrome 2A (autosomal recessive, mild) homolog (human) (Ush2a)	-3.436435713
Hist1h2bj	histone cluster 1, H2bj (Hist1h2bj)	-3.511402287
Pgls	6-phosphogluconolactonase (Pgls)	-3.522987814
Hmgn2	high mobility group nucleosomal binding domain 2 (Hmgn2)	-3.543889454
Tmem66	transmembrane protein 66 (Tmem66)	-3.696442025
Drctnnb1a	family with sequence similarity 126, member A (Fam126a)	-3.759056431
LOC100048105	similar to Ubc protein, transcript variant 1 (LOC100048105)	-3.972219584
Bok	BCL2-related ovarian killer (Bok)	-4.056219164
Igfbp7	insulin-like growth factor binding protein 7 (Igfbp7)	-4.291650838
Prkag2	protein kinase, AMP-activated, gamma 2 non-catalytic subunit (Prkag2)	-4.410234775
Rshl2a	radial spokehead-like 2A (Rshl2a)	-4.719902718
Cap1	CAP, adenylate cyclase-associated protein 1 (yeast) (Cap1)	-4.911150194
Med23	mediator complex subunit 23 (Med23)	-5.039952351
Oxt	oxytocin (Oxt)	-5.956808754
Pam	peptidylglycine alpha-amidating monooxygenase (Pam)	-6.099994195
1110012J17Rik	RIKEN cDNA 1110012J17 gene (1110012J17Rik)	-6.358775137
4930438O05Rik	armadillo repeat containing 9 (Armc9)	-11.82255149

Table S2. Significantly-regulated transcripts differentially regulated in 4 month old GIT2KO mice compared to age-matched WT controls. For each significantly-regulated transcript the Official Gene Symbol, transcript description and expression Z ratio, GIT2KO versus WT are represented.

Gene Symbol	Description	Z ratio GIT2KO vs. WT
Slc6a3	solute carrier family 6 (neurotransmitter transporter, dopamine), member 3 (Slc6a3)	8.884948429
Usp29	ubiquitin specific peptidase 29 (Usp29)	5.341907285
Tfrc	transferrin receptor (Tfrc)	3.87139835
Mtap1b	microtubule-associated protein 1 B (Mtap1b)	3.400549912
Dcun1d4	DCN1, defective in cullin neddylation 1, domain containing 4 (S. cerevisiae) (Dcun1d4)	3.054359536
Zfp292	zinc finger protein 292, transcript variant 4 (Zfp292)	3.042392196
Mark3	MAP/microtubule affinity regulating kinase 3 (Mark3)	3.011360482
Tfrc	transferrin receptor (Tfrc)	2.947233884
Vps33a	vacuolar protein sorting 33A (yeast) (Vps33a)	2.886795297
Sgip1	SH3-domain GRB2-like (endophilin) interacting protein 1 (Sgip1)	2.88396597
Grlf1	glucocorticoid receptor DNA binding factor 1 (Grlf1)	2.881309965
Atp6ap2	ATPase, H+ transporting, lysosomal accessory protein 2 (Atp6ap2)	2.874032162
Snapc3	small nuclear RNA activating complex, polypeptide 3 (Snapc3)	2.824227253
Slitrk4	SLIT and NTRK-like family, member 4 (Slitrk4)	2.635315326
Ddah1	dimethylarginine dimethylaminohydrolase 1 (Ddah1)	2.577621594
Cul3	cullin 3 (Cul3)	2.546525633
Coq5	coenzyme Q5 homolog, methyltransferase (yeast) (Coq5)	2.477456625
Serbp1	Serpine1 mRNA binding protein 1 (Serbp1)	2.441317036
Zfp655	zinc finger protein 655 (Zfp655), transcript variant 1	2.432411853
Eif4a2	eukaryotic translation initiation factor 4A2 (Eif4a2)	2.425771798
Skil	SKI-like (Skil), transcript variant 2	2.394716431
Cse1l	chromosome segregation 1-like (S. cerevisiae) (Cse1l)	2.373961692
Scoc	short coiled-coil protein (Scoc), transcript variant 2	2.370002634
Slc2a13	solute carrier family 2 (facilitated glucose transporter), member 13 (Slc2a13)	2.295277202
Nomo1	nodal modulator 1 (Nomo1)	2.257769263
Ntng1	netrin G1 (Ntng1)	2.25537914
Atad3a	ATPase family, AAA domain containing 3A (Atad3a)	2.247010786
EG245297	predicted gene, EG245297 (EG245297)	2.198133396
B230339M05Rik	RIKEN cDNA B230339M05 gene (B230339M05Rik)	2.180320111
Pigk	phosphatidylinositol glycan anchor biosynthesis, class K (Pigk), transcript variant 2	2.175906042
4930544G21Rik	RIKEN cDNA 4930544G21 gene (4930544G21Rik)	2.116304544
Usp22	ubiquitin specific peptidase 22 (Usp22)	2.094473657
Ccdc126	coiled-coil domain containing 126 (Ccdc126)	2.090085562
Syt1	synaptotagmin I (Syt1)	2.088783788
Nap1l1	nucleosome assembly protein 1-like 1 (Nap1l1)	2.083416711
Nrxn1	neurexin I (Nrxn1)	2.046213313
Rab3gap2	RAB3 GTPase activating protein subunit 2, transcript variant 4 (Rab3gap2)	2.039929163
Rcan2	regulator of calcineurin 2 (Rcan2), transcript variant 1	1.993866805
Cacnb4	calcium channel, voltage-dependent, beta 4 subunit (Cacnb4), transcript variant 2	1.985994761
Rundc3b	RUN domain containing 3B (Rundc3b)	1.968231597
Pitpna	phosphatidylinositol transfer protein, alpha (Pitpna)	1.954827835

Sidt2	SID1 transmembrane family, member 2 (Sidt2)	1.951592223
Pitx2	paired-like homeodomain transcription factor 2 (Pitx2), transcript variant 3	1.950255266
Myt1l	myelin transcription factor 1-like (Myt1l)	1.924190548
Tppp	tubulin polymerization promoting protein (Tppp)	1.913508437
Nrxn1	neurexin I (Nrxn1)	1.883656874
Hnrph1	heterogeneous nuclear ribonucleoprotein H1 (Hnrph1)	1.882820514
Lrrc49	leucine rich repeat containing 49 (Lrrc49)	1.872401181
Fusip1	serine/arginine-rich splicing factor 10 (Fusip1)	1.864602216
Btbd14a	BTB (POZ) domain containing 14A (Btbd14a), transcript variant 2	1.862464063
Sorl1	sortilin-related receptor, LDLR class A repeats-containing (Sorl1)	1.839214879
2610208M17Rik	RIKEN cDNA 2610208M17 gene (2610208M17Rik)	1.837486099
Stx5a	syntaxin 5A (Stx5a)	1.828867392
Necap1	NECAP endocytosis associated 1 (Necap1)	1.818046422
Fubp3	far upstream element (FUSE) binding protein 3 (Fubp3)	1.780836052
Rgs8	regulator of G-protein signaling 8 (Rgs8)	1.77782436
Ppm1e	protein phosphatase 1E (PP2C domain containing) (Ppm1e)	1.777336063
Fbxw17	F-box and WD-40 domain protein 17 (Fbxw17)	1.750565356
D10Ertd610e	DNA segment, Chr 10, ERATO Doi 610, expressed (D10Ertd610e)	1.737917625
Wipf2	WAS/WASL interacting protein family, member 2 (Wipf2)	1.72609076
Ppm1f	protein phosphatase 1F (PP2C domain containing) (Ppm1f)	1.725382256
Map3k12	mitogen-activated protein kinase kinase kinase 12 (Map3k12)	1.709767264
Sult4a1	sulfotransferase family 4A, member 1 (Sult4a1)	1.692137534
Zbtb33	zinc finger and BTB domain containing 33 (Zbtb33), transcript variant 2	1.68705513
Npm1	nucleophosmin 1 (Npm1)	1.678419705
Cops3	COP9 (constitutive photomorphogenic) homolog, subunit 3 (Arabidopsis thaliana) (Cops3)	1.67515031
Klhdc4	kelch domain containing 4 (Klhdc4)	1.67387089
Rrm2b	ribonucleotide reductase M2 B (TP53 inducible) (Rrm2b)	1.673553455
Ubac1	ubiquitin associated domain containing 1 (Ubac1)	1.671747178
Ulk1	Unc-51 like kinase 1 (C. elegans) (Ulk1)	1.667467498
Hmgn2	high mobility group nucleosomal binding domain 2 (Hmgn2)	1.65802932
Reep5	receptor accessory protein 5 (Reep5)	1.654227981
Wasf1	WASP family 1 (Wasf1)	1.630297497
Akap9	A kinase (PRKA) anchor protein (yotiao) 9 (Akap9)	1.619852457
Sfrs6	splicing factor, arginine/serine-rich 6 (Sfrs6)	1.586383111
Tax1bp1	Tax1 (human T-cell leukemia virus type I) binding protein 1 (Tax1bp1)	1.585412044
Syt11	synaptotagmin XI (Syt11)	1.575493959
Trp53bp1	transformation related protein 53 binding protein 1 (Trp53bp1)	1.559425487
AU040829	expressed sequence AU040829 (AU040829), transcript variant 2	1.558320351
Bcl2l2	Bcl2-like 2 (Bcl2l2)	1.553389727
Slc25a5	solute carrier family 25 (mitochondrial carrier, adenine nucleotide translocator), member 5 (Slc25a5), nuclear gene encoding mitochondrial protein	1.553378268
Zc3h13	zinc finger CCCH type containing 13 (Zc3h13)	1.542208518
Homer1	homer homolog 1 (Drosophila) (Homer1), transcript variant d	1.542111236
Dpm1	dolichol-phosphate (beta-D) mannosyltransferase 1 (Dpm1)	1.537375544
Ubp1	upstream binding protein 1 (Ubp1)	1.530770053
Snrk	SNF related kinase (Snrk)	1.513771426
H2-T23	histocompatibility 2, T region locus 23 (H2-T23)	-1.502825679
Znrf2	zinc and ring finger 2 (Znrf2)	-1.533957065
Bdh1	3-hydroxybutyrate dehydrogenase, type 1 (Bdh1)	-1.535705891

Igbp1	immunoglobulin (CD79A) binding protein 1 (Igbp1)	-1.550665009
Csnk1e	casein kinase 1, epsilon (Csnk1e)	-1.557275823
Trappc4	trafficking protein particle complex 4 (Trappc4)	-1.576080176
Scrib	scribbled homolog (Drosophila) (Scrib)	-1.576655514
LOC100047323	similar to ataxin 2 (LOC100047323), misc RNA.	-1.580072919
Hspb6	heat shock protein, alpha-crystallin-related, B6 (Hspb6)	-1.596615936
BC018242	cDNA sequence BC018242 (BC018242)	-1.601586462
Rabl3	RAB, member RAS oncogene family-like 3 (Rabl3)	-1.604501919
Tmem176b	transmembrane protein 176B (Tmem176b)	-1.604696251
Gpc3	glypican 3 (Gpc3)	-1.610770821
Ankle2	ankyrin repeat and LEM domain containing 2 (Ankle2)	-1.629850005
Ddost	dolichyl-di-phosphooligosaccharide-protein glycotransferase (Ddost)	-1.644332017
Tcp1	t-complex protein 1 (Tcp1)	-1.666154329
Btg1	B-cell translocation gene 1, anti-proliferative (Btg1)	-1.674771306
AA536749	myosin phosphatase Rho interacting protein (Mprip)	-1.680242592
Chgb	chromogranin B (Chgb)	-1.686987922
Rabl5	RAB, member RAS oncogene family-like 5 (Rabl5)	-1.699665869
Mapk1ip1	mitogen-activated protein kinase 1 interacting protein 1 (Mapk1ip1), transcript variant 1	-1.712989396
Cckbr	cholecystokinin B receptor (Cckbr)	-1.725123422
Eif4a1	eukaryotic translation initiation factor 4A1 (Eif4a1)	-1.732230946
Pih1d1	PIH1 domain containing 1 (Pih1d1)	-1.756576469
Tmem38b	transmembrane protein 38B (Tmem38b)	-1.769345585
Lrp4	low density lipoprotein receptor-related protein 4 (Lrp4)	-1.792618171
Cstf1	cleavage stimulation factor, 3' pre-RNA, subunit 1 (Cstf1)	-1.802097713
Ephx1	epoxide hydrolase 1, microsomal (Ephx1)	-1.808503977
LOC100048331	similar to DnaJ (Hsp40) homolog, subfamily A, member 4 (LOC100048331), misc RNA.	-1.821745793
Mdk	midkine (Mdk), transcript variant 1	-1.828259482
Ldb2	LIM domain binding 2 (Ldb2)	-1.833064952
Myadm	myeloid-associated differentiation marker (Myadm)	-1.834074247
Lass2	LAG1 homolog, ceramide synthase 2 (Lass2)	-1.838488083
4932409I22Rik	RIKEN cDNA 4932409I22 gene (4932409I22Rik)	-1.842261682
Hist1h2bn	histone cluster 1, H2bn (Hist1h2bn)	-1.844707225
Nipa1	non imprinted in Prader-Willi/Angelman syndrome 1 homolog (human) (Nipa1)	-1.881056998
Mgst1	microsomal glutathione S-transferase 1 (Mgst1)	-1.92196953
Manba	mannosidase, beta A, lysosomal (Manba)	-1.923304454
Dctn1	dynactin 1 (Dctn1)	-1.926846657
Trappc4	trafficking protein particle complex 4 (Trappc4)	-1.929808415
Rcl1	RNA terminal phosphate cyclase-like 1 (Rcl1)	-1.932664698
BC085271	cDNA sequence BC085271 (BC085271)	-1.935382845
Unc5c	unc-5 homolog C (C. elegans) (Unc5c)	-1.944927164
Wsb2	WD repeat and SOCS box-containing 2 (Wsb2)	-1.951142573
Vrk3	vaccinia related kinase 3 (Vrk3)	-1.959156703
Ncdn	neurochondrin (Ncdn)	-1.968156296
Pim3	proviral integration site 3 (Pim3)	-1.970144426
Mfsd11	major facilitator superfamily domain containing 11 (Mfsd11)	-1.97586597
Tgfb2	transforming growth factor, beta 2 (Tgfb2)	-1.978959018
Top1mt	DNA topoisomerase 1, mitochondrial (Top1mt)	-2.023211615
Bdh2	3-hydroxybutyrate dehydrogenase, type 2 (Bdh2)	-2.027682191

Vps24	vacuolar protein sorting 24 (yeast) (Vps24)	-2.028657002
Bxdc1	ribosome production factor 2 homolog (S. cerevisiae) (Bxdc1)	-2.04137933
Gstm2	glutathione S-transferase, mu 2 (Gstm2)	-2.043938439
Ormdl3	ORM1-like 3 (S. cerevisiae) (Ormdl3)	-2.04648611
Gstt3	glutathione S-transferase, theta 3 (Gstt3)	-2.046929881
Npc2	Niemann Pick type C2 (Npc2)	-2.047530558
B020018G12Rik	RIKEN cDNA B020018G12 gene (B020018G12Rik)	-2.054670362
Ssbp1	single-stranded DNA binding protein 1 (Ssbp1), transcript variant 2	-2.079013659
Lhfp	lipoma HMGIC fusion partner (Lhfp)	-2.089078251
EG277333	predicted gene, EG277333 (EG277333) on chromosome 1.	-2.120081045
Evl	Ena-vasodilator stimulated phosphoprotein (Evl)	-2.122496017
Prkcsh	protein kinase C substrate 80K-H (Prkcsh)	-2.138628586
Cetn4	centrin 4 (Cetn4)	-2.148373117
Zdhhc12	zinc finger, DHHC domain containing 12 (Zdhhc12)	-2.156110345
Eif1b	eukaryotic translation initiation factor 1B (Eif1b)	-2.16345075
2310007F21Rik	RIKEN cDNA 2310007F21 gene (2310007F21Rik)	-2.164311643
Fez2	fasciculation and elongation protein zeta 2 (zygin II) (Fez2)	-2.182298183
Dnaic1	dynein, axonemal, intermediate chain 1 (Dnaic1)	-2.199444934
Unc84b	unc-84 homolog B (C. elegans) (Unc84b)	-2.234440356
Nosip	nitric oxide synthase interacting protein (Nosip)	-2.274919256
Prr18	proline rich region 18 (Prr18)	-2.280509865
Coro2b	coronin, actin binding protein, 2B (Coro2b)	-2.302915707
2310079N02Rik	RIKEN cDNA 2310079N02 gene (2310079N02Rik)	-2.313900379
Lhfp	lipoma HMGIC fusion partner (Lhfp)	-2.375445866
LOC100047619	similar to solute carrier family 7 (cationic amino acid transporter, y+ system), member 5 (LOC100047619), misc RNA.	-2.37727313
Klhl32	kelch-like 32 (Drosophila) (Klhl32)	-2.399227866
D4Bwg0951e	DNA segment, Chr 4, Brigham & Women's Genetics 0951 expressed (D4Bwg0951e)	-2.440919063
Nbl1	neuroblastoma, suppression of tumorigenicity 1 (Nbl1)	-2.450946328
Centg2	centaurin, gamma 2 (Centg2), transcript variant 2	-2.452187792
Ppp1r10	protein phosphatase 1, regulatory subunit 10 (Ppp1r10)	-2.454523359
Hyi	hydroxypyruvate isomerase homolog (E. coli) (Hyi)	-2.47182066
1700047I17Rik1	RIKEN cDNA 1700047I17 gene 1 (1700047I17Rik1)	-2.512817347
Ifitm2	interferon induced transmembrane protein 2 (Ifitm2)	-2.521484816
Palm	paralemmin (Palm)	-2.536606226
Pomc	pro-opiomelanocortin-alpha (Pomc)	-2.585440863
Pard6a	par-6 (partitioning defective 6,) homolog alpha (C. elegans) (Pard6a), transcript variant 1	-2.60150514
Mrps17	mitochondrial ribosomal protein S17 (Mrps17)	-2.613771536
Ccndbp1	cyclin D-type binding-protein 1 (Ccndbp1)	-2.615423377
Atic	5-aminoimidazole-4-carboxamide ribonucleotide formyltransferase/IMP cyclohydrolase (Atic)	-2.615564572
Dusp19	dual specificity phosphatase 19 (Dusp19)	-2.620525774
Cldnd1	claudin domain containing 1 (Cldnd1)	-2.628755862
Cd63	CD63 antigen (Cd63)	-2.633818185
Snapc3	small nuclear RNA activating complex, polypeptide 3 (Snapc3)	-2.64199784
Rnf166	ring finger protein 166 (Rnf166)	-2.66446528
Kctd5	potassium channel tetramerisation domain containing 5 (Kctd5)	-2.734732908
Pop5	processing of precursor 5, ribonuclease P/MRP family (S. cerevisiae) (Pop5)	-2.744806589
Rhob	ras homolog gene family, member B (Rhob)	-2.744806862
Phlda3	pleckstrin homology-like domain, family A, member 3 (Phlda3)	-2.76892382

Slc27a1	solute carrier family 27 (fatty acid transporter), member 1 (Slc27a1)	-2.791266706
Anxa2	annexin A2 (Anxa2)	-2.791619525
H2-Bl	histocompatibility 2, blastocyst (H2-Bl)	-2.810180642
Syn1	synapsin I (Syn1)	-2.83291014
1810015A11Rik	RIKEN cDNA 1810015A11 gene (1810015A11Rik)	-2.843707964
Ptpre	protein tyrosine phosphatase, receptor type, E (Ptpre)	-2.896938051
Bag3	Bcl2-associated athanogene 3 (Bag3)	-2.948646299
LOC100048313	similar to ABC transporter, transcript variant 1 (LOC100048313)	-2.957586802
BC039093	zinc finger protein 362 (Zfp362)	-2.994761053
Cenpb	centromere protein B (Cenpb)	-3.025110723
Cebpb	CCAAT/enhancer binding protein (C/EBP), beta (Cebpb)	-3.03455863
Zfhx2	zinc finger homeobox 2 (Zfhx2)	-3.080982694
Tmem106c	transmembrane protein 106C (Tmem106c)	-3.084251825
1200015F23Rik	RIKEN cDNA 1200015F23 gene (1200015F23Rik)	-3.139588425
Emg1	EMG1 nucleolar protein homolog (S. cerevisiae) (Emg1)	-3.149242218
Serping1	serine (or cysteine) peptidase inhibitor, clade G, member 1 (Serping1)	-3.26071827
Dmwd	dystrophia myotonica-containing WD repeat motif (Dmwd)	-3.335593521
Zcchc3	zinc finger, CCHC domain containing 3 (Zcchc3)	-3.426357046
Selk	selenoprotein K (Selk)	-3.428833119
H2afx	H2A histone family, member X (H2afx)	-3.467780714
Lbh	limb-bud and heart (Lbh)	-3.484002114
Stmn1	stathmin 1 (Stmn1)	-3.646886342
2310016C16Rik	RIKEN cDNA 2310016C16 gene (2310016C16Rik)	-3.7146136
Id2	inhibitor of DNA binding 2 (Id2)	-3.931908685
Erdr1	erythroid differentiation regulator 1 (Erdr1)	-4.046012119
LOC668837	similar to ATP synthase, H+ transporting, mitochondrial F0 complex, subunit G (LOC668837), misc RNA.	-4.118625454
Aprt	adenine phosphoribosyl transferase (Aprt)	-4.337859151
Sgsm1	small G protein signaling modulator 1 (Sgsm1)	-4.5747027
Pebp1	phosphatidylethanolamine binding protein 1 (Pebp1)	-4.880811794
Tmsb10	thymosin, beta 10 (Tmsb10)	-5.010906229
Ccrn4l	CCR4 carbon catabolite repression 4-like (S. cerevisiae) (Ccrn4l)	-6.695594997

Table S3. Significantly-regulated transcripts differentially regulated in 8 month old GIT2KO mice compared to age-matched WT controls. For each significantly-regulated transcript the Official Gene Symbol, transcript description and expression Z ratio, GIT2KO versus WT are represented.

Gene Symbol	Description	Z ratio GIT2KO vs. WT
Gbx2	gastrulation brain homeobox 2 (Gbx2)	6.626384775
Hba-a1	hemoglobin alpha, adult chain 1 (Hba-a1)	6.369780315
Prkcd	protein kinase C, delta (Prkcd)	6.14489038
Tcf7l2	transcription factor 7-like 2, T-cell specific, HMG-box (Tcf7l2)	5.102267035
4933439C20Rik	RIKEN cDNA 4933439C20 gene (4933439C20Rik)	5.099271894
Rshl2a	radial spokehead-like 2A (Rshl2a)	4.01907551
Cck	cholecystokinin (Cck)	3.962542694
Cox6a2	cytochrome c oxidase, subunit VI a, polypeptide 2 (Cox6a2), nuclear gene encoding mitochondrial protein	3.581121436
Vps33a	vacuolar protein sorting 33A (yeast) (Vps33a)	3.577008747
Ccndbp1	cyclin D-type binding-protein 1 (Ccndbp1)	3.474492615
Ly6a	lymphocyte antigen 6 complex, locus A (Ly6a)	3.289839538
2610524H06Rik	RIKEN cDNA 2610524H06 gene (2610524H06Rik)	3.036193794
Tpst2	protein-tyrosine sulfotransferase 2 (Tpst2)	2.80933748
Taf15	TAF15 RNA polymerase II, TATA box binding protein (TBP)-associated factor (Taf15)	2.756092819
Ly6c1	lymphocyte antigen 6 complex, locus C1 (Ly6c1)	2.68406105
Tubb2b	tubulin, beta 2b (Tubb2b)	2.657038239
D630048P19Rik	optic atrophy 3 (Opa3)	2.622982452
Rab6	RAB6, member RAS oncogene family (Rab6)	2.613932931
Fscn1	fascin homolog 1, actin bundling protein (Strongylocentrotus purpuratus) (Fscn1)	2.524397056
Scrg1	scrapie responsive gene 1 (Scrg1)	2.516903121
Alk	anaplastic lymphoma kinase (Alk)	2.495578129
Tceal8	transcription elongation factor A (SII)-like 8 (Tceal8)	2.463039304
AI593442	expressed sequence AI593442 (AI593442), transcript variant 1	2.401523728
Tmem41a	transmembrane protein 41a (Tmem41a)	2.395164571
1700019D03Rik	RIKEN cDNA 1700019D03 gene (1700019D03Rik)	2.385688939
Cpne4	copine IV (Cpne4)	2.368939591
C1qc	complement component 1, q subcomponent, C chain (C1qc)	2.342547755
Apoc1	apolipoprotein C-I (Apoc1)	2.271177311
Rgs7	regulator of G protein signaling 7 (Rgs7)	2.25749504
Gsk3b	glycogen synthase kinase 3 beta (Gsk3b)	2.235869262
Eif2ak2	eukaryotic translation initiation factor 2-alpha kinase 2 (Eif2ak2)	2.202370989
Zmat4	zinc finger, matrin type 4 (Zmat4)	2.192763022
Kcnh1	potassium voltage-gated channel, subfamily H (eag-related), member 1 (Kcnh1), transcript variant 2	2.183897459
2300002D11Rik	RIKEN cDNA 2300002D11 gene (2300002D11Rik)	2.16830561
C1qb	complement component 1, q subcomponent, beta polypeptide (C1qb)	2.119342968
Mgst3	microsomal glutathione S-transferase 3 (Mgst3)	2.097263073
Pvalb	parvalbumin (Pvalb)	2.086253929
Atxn1	ataxin 1 (Atxn1)	2.068826933
Gatad1	GATA zinc finger domain containing 1 (Gatad1)	2.049536341
Atp6v1g2	ATPase, H+ transporting, lysosomal V1 subunit G2 (Atp6v1g2)	2.045658776
Hnrpl	heterogeneous nuclear ribonucleoprotein L (Hnrpl)	2.034521871

Ppapdc2	phosphatidic acid phosphatase type 2 domain containing 2 (Ppapdc2)	1.988169541
Ccdc86	coiled-coil domain containing 86 (Ccdc86)	1.986261938
Igsf3	immunoglobulin superfamily, member 3 (Igsf3)	1.953800699
1200003C05Rik	RIKEN cDNA 1200003C05 gene (1200003C05Rik)	1.937887986
Kcnip3	Kv channel interacting protein 3, calsenilin (Kcnip3)	1.917422447
BC048546	cDNA sequence BC048546 (BC048546)	1.912163083
C4b	complement component 4B (Childo blood group) (C4b)	1.897471113
Pigf	phosphatidylinositol glycan anchor biosynthesis, class F (Pigf)	1.880963104
Thoc3	THO complex 3 (Thoc3)	1.876339056
Nelf	nasal embryonic LHRH factor (Nelf), transcript variant 2	1.875489353
Mal	myelin and lymphocyte protein, T-cell differentiation protein (Mal)	1.862003919
Mnat1	menage a trois 1 (Mnat1)	1.857711515
Ddx6	DEAD (Asp-Glu-Ala-Asp) box polypeptide 6 (Ddx6)	1.855453632
Rora	RAR-related orphan receptor alpha (Rora)	1.843745214
Mbp	myelin basic protein (Mbp), transcript variant 8	1.842302382
Nrn1	neuritin 1 (Nrn1)	1.840311677
Nptx2	neuronal pentraxin 2 (Nptx2)	1.810157551
Chst2	carbohydrate sulfotransferase 2 (Chst2)	1.804514438
Rpl22	ribosomal protein L22 (Rpl22)	1.800943979
Znhit3	zinc finger, HIT type 3 (Znhit3)	1.791847506
Snx15	sorting nexin 15 (Snx15)	1.785252117
Fads2	fatty acid desaturase 2 (Fads2)	1.77287926
Maob	monoamine oxidase B (Maob), nuclear gene encoding mitochondrial protein	1.756539738
B230217C12Rik	RIKEN cDNA B230217C12 gene (B230217C12Rik)	1.755299768
Copg	coatomer protein complex, subunit gamma (Copg), transcript variant 2	1.737598235
Polr2h	polymerase (RNA) II (DNA directed) polypeptide H (Polr2h)	1.730601052
1810020D17Rik	RIKEN cDNA 1810020D17 gene (1810020D17Rik)	1.727999868
Snrpd1	small nuclear ribonucleoprotein D1 (Snrpd1)	1.703714332
Apoc1	apolipoprotein C-I (Apoc1)	1.702189193
3-Sep	septin 3 (Sept3)	1.675662049
Peg3	paternally expressed 3 (Peg3)	1.675551447
Ctxn1	cortexin 1 (Ctxn1)	1.67431095
Spock1	sparc/osteonectin, cwcv and kazal-like domains proteoglycan 1 (Spock1)	1.658079034
Ssu72	Ssu72 RNA polymerase II CTD phosphatase homolog (yeast) (Ssu72)	1.651955553
Olfm1	olfactomedin 1 (Olfm1), transcript variant 1	1.634782739
Snrpd1	small nuclear ribonucleoprotein D1 (Snrpd1)	1.631774611
Tmem60	transmembrane protein 60 (Tmem60)	1.60629939
Foxn3	forkhead box N3 (Foxn3)	1.594670803
Vdac1	voltage-dependent anion channel 1 (Vdac1)	1.592911248
Plch2	phospholipase C, eta 2 (Plch2), transcript variant 1	1.573211132
Asns	asparagine synthetase (Asns)	1.556261256
Mrpl48	mitochondrial ribosomal protein L48 (Mrpl48), transcript variant 1	1.555510766
Klhl32	kelch-like 32 (Drosophila) (Klhl32)	1.552064171
LOC100047012	similar to ubiquitin-conjugating enzyme UbcM2 (LOC100047012)	1.539841785
Hes6	hairy and enhancer of split 6 (Drosophila) (Hes6)	1.534303112
Pfn1	profilin 1 (Pfn1)	1.532441744
Slc1a1	solute carrier family 1 (neuronal/epithelial high affinity glutamate transporter, system Xag), member 1 (Slc1a1)	1.527778095
Rph3a	rabphilin 3A (Rph3a)	1.522281856

1810027O10Rik	RIKEN cDNA 1810027O10 gene (1810027O10Rik)	1.516759172
Acy1	aminoacylase 1 (Acy1)	1.516446814
Ehd4	EH-domain containing 4 (Ehd4)	1.504209567
Adora1	adenosine A1 receptor (Adora1), transcript variant 1	1.502606914
Chrna4	cholinergic receptor, nicotinic, alpha polypeptide 4 (Chrna4)	1.500092486
Nrd1	nardilysin, N-arginine dibasic convertase, NRD convertase 1 (Nrd1)	-1.503679589
Efha1	EF hand domain family A1 (Efha1)	-1.510090858
Golga3	golgi autoantigen, golgin subfamily a, 3 (Golga3)	-1.549749366
St5	suppression of tumorigenicity 5 (St5), transcript variant 2	-1.554453445
Krba1	KRAB-A domain containing 1 (Krba1)	-1.556158976
Nrf1	nuclear respiratory factor 1 (Nrf1)	-1.558155212
Gtf2h1	general transcription factor II H, polypeptide 1 (Gtf2h1)	-1.560673118
LOC100043391	similar to QM protein (LOC100043391)	-1.583695409
Maged1	melanoma antigen, family D, 1 (Maged1)	-1.585510311
Xbp1	X-box binding protein 1 (Xbp1)	-1.592215199
Slc7a10	solute carrier family 7 (cationic amino acid transporter, y+ system), member 10 (Slc7a10)	-1.612182098
Morc2a	microrchidia 2A (Morc2a)	-1.627777388
Mfap3	microfibrillar-associated protein 3 (Mfap3), transcript variant 2	-1.636109593
Igsf1	immunoglobulin superfamily, member 1 (Igsf1), transcript variant 4	-1.697365329
Ift81	intraflagellar transport 81 homolog (Chlamydomonas) (Ift81)	-1.707014079
Tmem136	transmembrane protein 136 (Tmem136)	-1.710695812
Cc2d2a	coiled-coil and C2 domain containing 2A (Cc2d2a)	-1.722385391
Sipa1l1	signal-induced proliferation-associated 1 like 1 (Sipa1l1)	-1.722699938
Adcy9	adenylate cyclase 9 (Adcy9)	-1.744266232
Zhx1	zinc fingers and homeoboxes 1 (Zhx1), transcript variant 1	-1.746059268
4933411K20Rik	RIKEN cDNA 4933411K20 gene (4933411K20Rik)	-1.775134857
Bop1	block of proliferation 1 (Bop1)	-1.785050781
2010321M09Rik	RIKEN cDNA 2010321M09 gene (2010321M09Rik), transcript variant 1	-1.802122302
Inppl1	inositol polyphosphate phosphatase-like 1 (Inppl1)	-1.804352106
Mfn1	mitofusin 1 (Mfn1)	-1.80626159
Doc2b	double C2, beta (Doc2b)	-1.812298953
Narg2	NMDA receptor-regulated gene 2 (Narg2)	-1.817017139
Rab1	RAB1, member RAS oncogene family (Rab1)	-1.826484927
Rasgrf1	RAS protein-specific guanine nucleotide-releasing factor 1 (Rasgrf1), transcript variant 1	-1.871209845
Stard4	StAR-related lipid transfer (START) domain containing 4 (Stard4)	-1.926517577
Zfp263	zinc finger protein 263 (Zfp263)	-1.931087097
Iap	magnesium transporter 1 (Magt)	-1.946857676
Heatr1	HEAT repeat containing 1 (Heatr1)	-1.950988944
Rb1	retinoblastoma 1 (Rb1)	-1.952516863
Zmym3	zinc finger, MYM-type 3 (Zmym3)	-1.977171054
Mfn1	mitofusin 1 (Mfn1)	-2.009575483
Nt5c	5',3'-nucleotidase, cytosolic (Nt5c)	-2.039008678
Nup210	nucleoporin 210 (Nup210)	-2.045951159
Spag5	sperm associated antigen 5 (Spag5)	-2.061899567
BC021381	cDNA sequence BC021381 (BC021381)	-2.072366749
Cml1	camello-like 1 (Cml1)	-2.081851727
Hsdl1	hydroxysteroid dehydrogenase like 1 (Hsdl1)	-2.103404399
Ttc27	tetratricopeptide repeat domain 27 (Ttc27)	-2.117077839

Bace1	beta-site APP cleaving enzyme 1 (Bace1)	-2.125194569
Fbxo18	F-box protein 18 (Fbxo18)	-2.141171447
Ganc	glucosidase, alpha; neutral C (Ganc)	-2.14989671
Txndc12	thioredoxin domain containing 12 (endoplasmic reticulum) (Txndc12)	-2.150086813
Ankle2	ankyrin repeat and LEM domain containing 2 (Ankle2)	-2.173763211
Lamp2	lysosomal-associated membrane protein 2 (Lamp2)	-2.177791212
Mapk10	mitogen-activated protein kinase 10 (Mapk10), transcript variant 1	-2.263673599
Igsf1	immunoglobulin superfamily, member 1 (Igsf1), transcript variant 4	-2.291734954
Dag1	dystroglycan 1 (Dag1)	-2.303725342
Tmem118	transmembrane protein 118 (Tmem118)	-2.323948545
Anapc5	anaphase-promoting complex subunit 5 (Anapc5), transcript variant 1	-2.347916771
Ifngr2	interferon gamma receptor 2 (Ifngr2)	-2.358257035
Tcof1	Treacher Collins Franceschetti syndrome 1, homolog (Tcof1)	-2.405206581
Evl	Ena-vasodilator stimulated phosphoprotein (Evl)	-2.413606156
Ahi1	Abelson helper integration site (Ahi1)	-2.444126093
Bbs9	Bardet-Biedl syndrome 9 (Bbs9)	-2.466775354
Pebp1	phosphatidylethanolamine binding protein 1 (Pebp1)	-2.480778318
BC026590	cDNA sequence BC026590 (BC026590)	-2.485006001
Mrps17	mitochondrial ribosomal protein S17 (Mrps17)	-2.509580186
Tex261	testis expressed gene 261 (Tex261)	-2.527993615
Mmp17	matrix metallopeptidase 17 (Mmp17)	-2.571305567
Agpat3	1-acylglycerol-3-phosphate O-acyltransferase 3 (Agpat3)	-2.572133429
Sytl4	synaptotagmin-like 4 (Sytl4)	-2.619455528
Dab2	disabled 2, mitogen-responsive phosphoprotein (Dab2)	-2.732379713
Nkx2-1	NK2 homeobox 1 (Nkx2-1)	-2.793335251
Ccdc117	coiled-coil domain containing 117 (Ccdc117)	-2.879651056
Stt3b	STT3, subunit of the oligosaccharyltransferase complex, homolog B (S. cerevisiae) (Stt3b)	-2.922904936
Ntrk3	neurotrophic tyrosine kinase, receptor, type 3 (Ntrk3), transcript variant 2	-3.045749617
D030028O16Rik	prolyl endopeptidase-like (Prepl)	-3.060079272
Diablo	diablo homolog (Drosophila) (Diablo), nuclear gene encoding mitochondrial protein	-3.077811292
Sgsm1	small G protein signaling modulator 1 (Sgsm1)	-3.385216179
Gjb2	gap junction protein, beta 2 (Gjb2)	-3.459770638
Actl6b	actin-like 6B (Actl6b)	-6.915433237
Ptgds	prostaglandin D2 synthase (brain) (Ptgds)	-7.60184328

Table S4. Textrous! Collective analysis of significantly-regulated hypothalamic transcripts common across GITKO mice of 2, 4 and 8 months of age compared top age-matched controls. Output words were categorized according to their major molecular function (Metabolism, Neurotransmission, Musculoskeletal/Cardiac, Behavioral, Cell Signaling). For each extracted word the Cosine Similarity score (ranging from 0.1 to 1.0, with 1.0 representing the strongest transcript-word association).

Category	Word	Cosine Similarity
Metabolism	adenosine	0.484127286
	sedative	0.454258234
	adipocytes	0.442756374
	disposal	0.394869729
	lipolysis	0.391250485
	insulin	0.387412579
	adipocyte	0.38221831
	uncoupling	0.37836132
	proinsulin	0.377527625
	euglycemic	0.371305862
	glucose	0.361471934
	fatty	0.359751184
	insulinoma	0.357679037
	appetitive	0.356731173
	intolerance	0.351213085
	brown	0.350300572
	hexokinase	0.340886461
	insulin-responsive	0.336716248
	composition	0.328982902
	triacylglycerol	0.327510184
	stearoyl	0.325790402
	incretin	0.32362864
	propionate	0.320725306
	c-peptide	0.316171515
	thermogenesis	0.314643299
	glucagon	0.314530778
	propionic	0.311694835
Neurotransmission	ampa	0.473965815
	transients	0.440161292
	synapses	0.426325175
	store	0.423832937

	potentiation	0.419391409
	synaptic	0.418008216
	epileptiform	0.417623566
	n-methyl-d-aspartic	0.417473881
	nmda	0.413161867
	postsynaptic	0.405869227
	ionotropic	0.401519803
	halothane	0.396510454
	n-methyl-d-aspartate	0.392839492
	kainate	0.383384475
	nmdar	0.378965488
	impulses	0.375068683
	spines	0.373297582
	neostriatum	0.371691844
	post-synaptic	0.367109291
	plasticity	0.365964557
	pools	0.364249228
	dissociated	0.363872627
	release	0.357801833
	epileptic	0.352826109
	synapsin	0.346343932
	spikes	0.344784091
	tonic	0.338413146
	picrotoxin	0.33828452
	calcium	0.336823089
	calcium	0.336823089
	clustering	0.335467833
	synaptobrevin	0.330696655
	tetanic	0.329383717
	bushy	0.327504652
	electrodes	0.326629168
	fiber	0.325209852
	l-type	0.323771328
	electrical	0.321973348
	ionophore	0.320712571
	tetanus	0.319371528
	evoked	0.312854852
	microdomain	0.312745543
	receptor-mediated	0.310835459
	seizure	0.310475412

Muscolokeletal/Cardiac	gastrocnemius	0.37594938
	beat	0.365479609
	myotubes	0.349338992
	skeletal	0.345972235
	strength	0.333075738
	sinoatrial	0.320158357
	hearts	0.319426435
	beta-ar	0.317142239
	sarcoplasmic	0.310402962
Behavioral	swimming	0.425193737
	righting	0.415563008
	thicknesses	0.341921267
	apyrase	0.334226377
	bathing	0.333214314
	photobleaching	0.332533996
	immunogold	0.327860455
	hyperthyroidism	0.318862456
	knock-out	0.313112886
	erythroblastosis	0.311481281
	vastus	0.311192893
	knock-down	0.310269945
Cell Signaling	inositol	0.389090632
	oligomycin	0.388464126
	camk	0.362554963
	ribonucleoside	0.352332043
	somatostatin	0.348689825
	scaffolding	0.337833775
	laminin	0.324340261
	phosphatidylinositide	0.317269325
	endoproteases	0.312700031

Table S5. User-defined input interrogator terms for hypothalamic transcriptome transcript-word association analysis across the three experimental timepoints. The input interrogator terms were applied to the 2, 4 or 8 month old transcript datasets pre-loaded into GeneIndexer. A Cosine Similarity cut-off score of >0.1 was employed for the extracted transcripts linked implicitly with the interrogator terms.

**Interrogator Term**
diabetes
obesity
metabolism
energy
aging
mitochondria
oxidative phosphorylation
electron transport chain
thermogenesis
heat
insulin
appetite
thirst
circadian
sleep
glucose
fat
adipose

Table S9. GeneIndexer LSI-based targeted analysis of hypothalamic transcriptomic data from GIT2KO mice. For each input interrogator term, used to analyze 2 m.o., 4 m.o. or 8 m.o. GIT2KO transcriptomic data, the mean cosine similarity (CS) score, the relative rank among the other interrogator terms (Rank) and the reciprocal of this Rank score is indicated.

Interrogator Term	2 m.o. mean CS	Rank	$Rank^{-1}$		4 m.o. mean CS	Rank	$Rank^{-1}$		8 m.o. mean CS	Rank	$Rank^{-1}$
mitochondria	0.215	4	0.250		0.221	3	0.333		0.221	1	1.000
diabetes	0.183	14	0.071		0.151	16	0.063		0.194	2	0.500
insulin	0.191	9	0.111		0.175	13	0.077		0.192	3	0.333
glucose	0.191	10	0.100		0.151	15	0.067		0.189	4	0.250
electron transport chain	0.183	15	0.067		0.178	12	0.083		0.189	5	0.200
fat	0.189	12	0.083		0.189	10	0.100		0.184	8	0.125
energy	0.179	16	0.063		0.166	14	0.071		0.161	12	0.083
aging	0.144	18	0.056		0.144	17	0.059		0.160	13	0.077
circadian	0.322	1	1.000		0.424	1	1.000		0.188	6	0.167
thirst	0.293	2	0.500		0.000	18	0.056		0.000	18	0.056
thermogenesis	0.287	3	0.333		0.195	6	0.167		0.146	16	0.063
heat	0.190	11	0.091		0.198	5	0.200		0.115	17	0.059
sleep	0.195	8	0.125		0.193	7	0.143		0.168	9	0.111
appetite	0.163	17	0.059		0.181	11	0.091		0.147	15	0.067
obesity	0.208	5	0.200		0.240	2	0.500		0.149	14	0.071
adipose	0.196	7	0.143		0.204	4	0.250		0.165	10	0.100
oxidative phosphorylation	0.189	13	0.077		0.192	8	0.125		0.164	11	0.091
metabolism	0.200	6	0.167		0.189	9	0.111		0.185	7	0.143

Table S10. KEGG signaling pathway analysis of hypothalamic transcriptomic data from 2 m.o. GIT2KO mice. Significantly-populated KEGG signaling pathways, generated using the transcripts differentially regulated in 2 m.o. GIT2KO hypothalamic compared to WT controls, are depicted. For each specific KEGG pathway annotation the following parameter indices are indicated: **C** - total background number of transcripts populating the KEGG pathway; **O** – number of observed transcripts within the input dataset that are contained within the specific KEGG pathway; **E** – number of transcripts from the input dataset expected to be present at background levels; **R** – transcript enrichment factor in specific KEGG pathway, **P** – enrichment probability; **H** – hybrid score = $-\log_{10}P * R$.

KEGG Pathway	C	O	E	R	P	H
Metabolic pathways	1229	30	6.91	4.34	1.55E-09	38.23396
Dorso-ventral axis formation	22	4	0.12	32.32	0.0002	119.5507
Spliceosome	146	7	0.82	8.52	0.0004	28.95045
Endocytosis	222	8	1.25	6.4	0.0006	20.61983
Glycosaminoglycan degradation	23	3	0.13	23.18	0.0028	59.17488
Notch signaling pathway	56	4	0.32	12.7	0.0028	32.42109
Huntington's disease	231	7	1.3	5.39	0.0032	13.44724
Pathways in cancer	344	8	1.94	4.13	0.0056	9.299983
Melanoma	77	4	0.43	9.23	0.0062	20.37622
MAPK signaling pathway	281	7	1.58	4.43	0.0067	9.630489
Synthesis and degradation of ketone bodies	11	2	0.06	32.32	0.007	69.64643
Pyruvate metabolism	43	3	0.24	12.4	0.007	26.72078
Amino sugar and nucleotide sugar metabolism	44	3	0.25	12.12	0.007	26.11741
Nucleotide excision repair	44	3	0.25	12.12	0.007	26.11741
Prostate cancer	94	4	0.53	7.56	0.007	16.29106
Insulin signaling pathway	146	5	0.82	6.09	0.007	13.12335
N-Glycan biosynthesis	46	3	0.26	11.59	0.0072	24.83352
Other glycan degradation	16	2	0.09	22.22	0.0112	43.34638
RNA degradation	69	3	0.39	7.73	0.0204	13.06656
Adipocytokine signaling pathway	70	3	0.39	7.62	0.0204	12.88062
Glycosylphosphatidylinositol(GPI)-anchor biosynthesis	26	2	0.15	13.67	0.0239	22.1673
Ubiquitin mediated proteolysis	147	4	0.83	4.84	0.0239	7.848554
Regulation of actin cytoskeleton	227	5	1.28	3.91	0.0239	6.340464
Heparan sulfate biosynthesis	28	2	0.16	12.7	0.0252	20.30221
Hypertrophic cardiomyopathy (HCM)	85	3	0.48	6.27	0.0257	9.969719
PPAR signaling pathway	85	3	0.48	6.27	0.0257	9.969719
Chronic myeloid leukemia	85	3	0.48	6.27	0.0257	9.969719
Alanine, aspartate and glutamate metabolism	31	2	0.17	11.47	0.0264	18.1042
Purine metabolism	163	4	0.92	4.36	0.0268	6.853332
Gap junction	94	3	0.53	5.67	0.0302	8.618361
Butanoate metabolism	38	2	0.21	9.35	0.034	13.73067
Systemic lupus erythematosus	179	4	1.01	3.97	0.034	5.830029
Porphyrin and chlorophyll metabolism	39	2	0.22	9.11	0.0346	13.30902
Lysine degradation	41	2	0.23	8.67	0.0369	12.42388

Valine, leucine and isoleucine degradation	48	2	0.27	7.41	0.048	9.772002

Table S11. KEGG signaling pathway analysis of hypothalamic transcriptomic data from 4 m.o. GIT2KO mice. Significantly-populated KEGG signaling pathways, generated using the transcripts differentially regulated in 4 m.o. GIT2KO hypothalamic compared to WT controls, are depicted. For each specific KEGG pathway annotation the following parameter indices are indicated: **C** - total background number of transcripts populating the KEGG pathway; **O** – number of observed transcripts within the input dataset that are contained within the specific KEGG pathway; **E** – number of transcripts from the input dataset expected to be present at background levels; **R** – transcript enrichment factor in specific KEGG pathway, **P** – enrichment probability; **H** – hybrid score = $-\log_{10}P * R$.

KEGG Pathway	C	O	E	R	P	H
Synthesis and degradation of ketone bodies	10	2	0.03	59.34	0.0077	125.4156
Glutathione metabolism	54	4	0.18	21.98	0.0011	65.03019
Butanoate metabolism	30	2	0.1	19.78	0.0143	36.48745
Metabolism of xenobiotics by cytochrome P450	77	3	0.26	11.56	0.0107	22.78032
TGF-beta signaling pathway	85	3	0.29	10.47	0.0107	20.63235
Ribosome biogenesis in eukaryotes	86	3	0.29	10.35	0.0107	20.39588
N-Glycan biosynthesis	50	2	0.17	11.87	0.0279	18.45068
Allograft rejection	52	2	0.18	11.41	0.0279	17.73566
Graft-versus-host disease	54	2	0.18	10.99	0.0279	17.08281
Type I diabetes mellitus	59	2	0.2	10.06	0.031	15.1769
Purine metabolism	168	4	0.57	7.06	0.0107	13.91255
RNA transport	168	4	0.57	7.06	0.0107	13.91255
Endocytosis	220	5	0.74	6.74	0.0093	13.69242
Autoimmune thyroid disease	67	2	0.23	8.86	0.0359	12.80186
Lysosome	123	3	0.41	7.24	0.024	11.72727
Antigen processing and presentation	78	2	0.26	7.61	0.0434	10.3687
Hypertrophic cardiomyopathy (HCM)	83	2	0.28	7.15	0.0453	9.608898
Cell adhesion molecules (CAMs)	149	3	0.5	5.97	0.0279	9.279743
Drug metabolism - cytochrome P450	87	2	0.29	6.82	0.0453	9.16541
Dilated cardiomyopathy	89	2	0.3	6.67	0.0453	8.963825
Viral myocarditis	89	2	0.3	6.67	0.0453	8.963825
Phagosome	176	3	0.59	5.06	0.0359	7.311222
MAPK signaling pathway	268	4	0.9	4.43	0.0279	6.885973
Huntington's disease	197	3	0.66	4.52	0.0434	6.158546
Metabolic pathways	1184	11	3.99	2.76	0.0107	5.438901

Table S12. KEGG signaling pathway analysis of hypothalamic transcriptomic data from 8 m.o. GIT2KO mice. Significantly-populated KEGG signaling pathways, generated using the transcripts differentially regulated in 8 m.o. GIT2KO hypothalamic compared to WT controls, are depicted. For each specific KEGG pathway annotation the following parameter indices are indicated: **C** - total background number of transcripts populating the KEGG pathway; **O** – number of observed transcripts within the input dataset that are contained within the specific KEGG pathway; **E** – number of transcripts from the input dataset expected to be present at background levels; **R** – transcript enrichment factor in specific KEGG pathway, **P** – enrichment probability; **H** – hybrid score = $-\log_{10}P * R$.

KEGG	C	O	E	R	P	H
Metabolic pathways	1229	14	3.32	4.22	0.0003	14.86655
Neurotrophin signaling pathway	144	5	0.39	12.87	0.001	38.61
Complement and coagulation cascades	75	3	0.2	14.82	0.0129	28.00106
Progesterone-mediated oocyte maturation	91	3	0.25	12.22	0.0129	23.08859
Prostate cancer	94	3	0.25	11.83	0.0129	22.35172
Colorectal cancer	94	3	0.25	11.83	0.0129	22.35172
Systemic lupus erythematosus	179	4	0.48	8.28	0.0129	15.64432
GnRH signaling pathway	103	3	0.28	10.79	0.0137	20.10479
Melanogenesis	106	3	0.29	10.49	0.0137	19.5458
Huntington's disease	231	4	0.62	6.42	0.0152	11.67256
Prion diseases	38	2	0.1	19.5	0.0179	34.06937
Nucleotide excision repair	44	2	0.12	16.84	0.0205	28.43006
Cell cycle	139	3	0.38	8	0.0205	13.50597
Type II diabetes mellitus	49	2	0.13	15.12	0.0207	25.46253
Glutathione metabolism	54	2	0.15	13.72	0.0207	23.10489
Arginine and proline metabolism	55	2	0.15	13.47	0.0207	22.68388
Basal cell carcinoma	56	2	0.15	13.23	0.0207	22.27971
Endometrial cancer	56	2	0.15	13.23	0.0207	22.27971
Wnt signaling pathway	160	3	0.43	6.95	0.0207	11.70401
Purine metabolism	163	3	0.44	6.82	0.0207	11.48508
Pathways in cancer	344	4	0.93	4.31	0.0283	6.6728
Arrhythmogenic right ventricular cardiomyopathy (ARVC)	76	2	0.21	9.75	0.0308	14.73663
Pancreatic cancer	77	2	0.21	9.62	0.0308	14.54014
Chemokine signaling pathway	203	3	0.55	5.48	0.0308	8.282742
Focal adhesion	207	3	0.56	5.37	0.0308	8.116483
Drug metabolism - cytochrome P450	85	2	0.23	8.72	0.0339	12.81666
Fc epsilon RI signaling pathway	85	2	0.23	8.72	0.0339	12.81666
ErbB signaling pathway	93	2	0.25	7.97	0.0368	11.43019
Dilated cardiomyopathy	94	2	0.25	7.88	0.0368	11.30112
Gap junction	94	2	0.25	7.88	0.0368	11.30112
Pyrimidine metabolism	102	2	0.28	7.27	0.0414	10.05441
Alzheimer's disease	265	3	0.72	4.19	0.0454	5.626936

Table S14. Significantly-regulated transcripts differentially regulated in 8 month old GIT2KO mouse extracted pancreatic islets compared to age-matched WT control islets. For each significantly-regulated transcript the Official Gene Symbol, transcript description and expression Z ratio, GIT2KO versus WT are represented.

Gene Symbol	Description	Z ratio
Cxcl1	chemokine (C-X-C motif) ligand 1 (Cxcl1)	12.01
Glycam1	glycosylation dependent cell adhesion molecule 1 (Glycam1)	11.69
LOC100041504	similar to beta chemokine Exodus-2 (LOC100041504)	11.26
Slpi	secretory leukocyte peptidase inhibitor (Slpi)	11.21
Ccl21b	chemokine (C-C motif) ligand 21b (Ccl21b)	11.15
Ppy	pancreatic polypeptide (Ppy)	10.92
Ccl21c	chemokine (C-C motif) ligand 21c (leucine) (Ccl21c)	10.6
Ctrc	chymotrypsin C (caldecrin) (Ctrc)	10.07
Ccl4	chemokine (C-C motif) ligand 4 (Ccl4)	9.85
Prg2	proteoglycan 2, bone marrow (Prg2)	9.62
Slc7a14	solute carrier family 7 (cationic amino acid transporter, y+ system), member 14 (Slc7a14)	9.27
2300002D11Rik	RIKEN cDNA 2300002D11 gene (2300002D11Rik)	8.95
Angptl7	angiopoietin-like 7 (Angptl7)	8.81
Vwf	Von Willebrand factor homolog (Vwf)	8.47
Rgs5	regulator of G-protein signaling 5 (Rgs5)	8.46
Ccl4	chemokine (C-C motif) ligand 4 (Ccl4)	8.43
Mustn1	musculoskeletal, embryonic nuclear protein 1 (Mustn1)	8.08
Acta2	actin, alpha 2, smooth muscle, aorta (Acta2)	8.04
Rbp7	retinol binding protein 7, cellular (Rbp7)	7.77
Cytl1	cytokine like 1 (Cytl1)	7.66
Bdh2	3-hydroxybutyrate dehydrogenase, type 2 (Bdh2)	7.58
Actg2	actin, gamma 2, smooth muscle, enteric (Actg2)	7.54
Gpr81	G protein-coupled receptor 81 (Gpr81)	7.38
Spp1	secreted phosphoprotein 1 (Spp1)	7.19
Rnase4	ribonuclease, RNase A family 4 (Rnase4), transcript variant 1	7.16
Il6	interleukin 6 (Il6)	6.81
2010001M09Rik	RIKEN cDNA 2010001M09 gene (2010001M09Rik)	6.58
Ccl19	chemokine (C-C motif) ligand 19 (Ccl19)	6.51
Tubb6	tubulin, beta 6 (Tubb6)	6.48
Sncg	synuclein, gamma (Sncg)	6.4
Dapl1	death associated protein-like 1 (Dapl1)	6.36
Qpct	glutaminyl-peptide cyclotransferase (glutaminyl cyclase) (Qpct)	6.36
Inmt	indolethylamine N-methyltransferase (Inmt)	6.05
Myh11	myosin, heavy polypeptide 11, smooth muscle (Myh11)	6.05
Cap1	CAP, adenylate cyclase-associated protein 1 (yeast) (Cap1)	5.99
Edn1	endothelin 1 (Edn1)	5.88
Tek	endothelial-specific receptor tyrosine kinase (Tek)	5.82
Acot1	acyl-CoA thioesterase 1 (Acot1)	5.75
Cldn11	claudin 11 (Cldn11)	5.71
Cav1	caveolin, caveolae protein 1 (Cav1)	5.69

Ddit3	DNA-damage inducible transcript 3 (Ddit3)	5.67
6430573F11Rik	RIKEN cDNA 6430573F11 gene (6430573F11Rik)	5.6
S100a6	S100 calcium binding protein A6 (calcyclin) (S100a6)	5.6
Hyou1	hypoxia up-regulated 1 (Hyou1)	5.57
Ogn	osteoglycin (Ogn)	5.53
Tpm2	tropomyosin 2, beta (Tpm2)	5.53
Vim	vimentin (Vim)	5.5
Fabp3	fatty acid binding protein 3, muscle and heart (Fabp3)	5.49
Serpinf1	serine (or cysteine) peptidase inhibitor, clade F, member 1 (Serpinf1)	5.45
Igf2	insulin-like growth factor 2 (Igf2)	5.34
S100a8	S100 calcium binding protein A8 (calgranulin A) (S100a8)	5.33
Emp1	epithelial membrane protein 1 (Emp1)	5.32
Arntl	aryl hydrocarbon receptor nuclear translocator-like (Arntl)	5.23
Mgp	matrix Gla protein (Mgp)	5.22
Hbegf	heparin-binding EGF-like growth factor (Hbegf)	5.15
Bgn	biglycan (Bgn)	5.14
Icam2	intercellular adhesion molecule 2 (Icam2)	5.13
Cuedc1	CUE domain containing 1 (Cuedc1)	5.12
Spon2	spondin 2, extracellular matrix protein (Spon2)	5.09
Anxa3	annexin A3 (Anxa3)	5.05
Nfkbia	nuclear factor of kappa light polypeptide gene enhancer in B-cells inhibitor, alpha (Nfkbia)	5.04
Prelp	proline arginine-rich end leucine-rich repeat (Prelp)	5.04
2610524H06Rik	RIKEN cDNA 2610524H06 gene (2610524H06Rik)	5.02
Prcp	prolylcarboxypeptidase (angiotensinase C) (Prcp)	5
Ctgf	connective tissue growth factor (Ctgf)	4.98
Tmsb10	thymosin, beta 10 (Tmsb10)	4.98
Kras	v-Ki-ras2 Kirsten rat sarcoma viral oncogene homolog (Kras)	4.96
Lum	lumican (Lum)	4.96
Mgst1	microsomal glutathione S-transferase 1 (Mgst1)	4.95
Fbln2	fibulin 2 (Fbln2), transcript variant 2	4.94
Dcn	decorin (Dcn)	4.88
Fgd2	FYVE, RhoGEF and PH domain containing 2 (Fgd2)	4.87
ENSMUSG00000068790	predicted gene, ENSMUSG00000068790 (ENSMUSG00000068790)	4.86
Igfbp5	insulin-like growth factor binding protein 5 (Igfbp5)	4.86
Sdpr	serum deprivation response (Sdpr)	4.79
Ndufb10	NADH dehydrogenase (ubiquinone) 1 beta subcomplex, 10 (Ndufb10)	4.78
Tubb2b	tubulin, beta 2b (Tubb2b)	4.78
Glo1	glyoxalase 1 (Glo1)	4.76
Darc	Duffy blood group, chemokine receptor (Darc)	4.73
Ccl3	chemokine (C-C motif) ligand 3 (Ccl3)	4.72
Ier3	immediate early response 3 (Ier3)	4.71
Aqp1	aquaporin 1 (Aqp1)	4.69
Rarres2	retinoic acid receptor responder (tazarotene induced) 2 (Rarres2)	4.69
Cd83	CD83 antigen (Cd83)	4.66
Mef2c	myocyte enhancer factor 2C (Mef2c)	4.61
Fcgrt	Fc receptor, IgG, alpha chain transporter (Fcgrt)	4.6
Pecam1	platelet/endothelial cell adhesion molecule 1 (Pecam1), transcript variant 1	4.57
Aoc3	amine oxidase, copper containing 3 (Aoc3)	4.53

Gucy1a3	guanylate cyclase 1, soluble, alpha 3 (Gucy1a3)	4.51
Ckb	creatine kinase, brain (Ckb)	4.48
Lmcd1	LIM and cysteine-rich domains 1 (Lmcd1)	4.47
Fbln1	fibulin 1 (Fbln1)	4.44
Gja5	gap junction membrane channel protein alpha 5 (Gja5)	4.43
Adi1	acireductone dioxygenase 1 (Adi1)	4.42
Hsd11b1	hydroxysteroid 11-beta dehydrogenase 1 (Hsd11b1), transcript variant 1	4.42
Rbp1	retinol binding protein 1, cellular (Rbp1)	4.41
Id2	inhibitor of DNA binding 2 (Id2)	4.4
Slc2a2	solute carrier family 2 (facilitated glucose transporter), member 2 (Slc2a2)	4.37
Scara3	scavenger receptor class A, member 3 (Scara3)	4.35
Vcam1	vascular cell adhesion molecule 1 (Vcam1)	4.35
Plvap	plasmalemma vesicle associated protein (Plvap)	4.3
Fxyd6	FXYD domain-containing ion transport regulator 6 (Fxyd6)	4.29
Lmo2	LIM domain only 2 (Lmo2)	4.29
Sparc	secreted acidic cysteine rich glycoprotein (Sparc)	4.26
Gdf15	growth differentiation factor 15 (Gdf15)	4.25
Angptl4	angiopoietin-like 4 (Angptl4)	4.24
Ift81	intraflagellar transport 81 homolog (Chlamydomonas) (Ift81)	4.21
Ptprb	protein tyrosine phosphatase, receptor type, B (Ptprb)	4.21
Igfbp4	insulin-like growth factor binding protein 4 (Igfbp4)	4.18
Tmem66	transmembrane protein 66 (Tmem66)	4.17
Gadd45b	growth arrest and DNA-damage-inducible 45 beta (Gadd45b)	4.16
Gp38	podoplanin (Pdpln)	4.15
Srgn	serglycin (Srgn)	4.15
Ahnak	AHNAK nucleoprotein (desmoyokin) (Ahnak), transcript variant 1	4.13
Sostdc1	sclerostin domain containing 1 (Sostdc1)	4.13
Wfdc2	WAP four-disulfide core domain 2 (Wfdc2)	4.13
C1qtnf9	C1q and tumor necrosis factor related protein 9 (C1qtnf9)	4.12
2310016C08Rik	RIKEN cDNA 2310016C08 gene (2310016C08Rik)	4.06
Apoe	apolipoprotein E (Apoe)	4.06
Ranbp3l	RAN binding protein 3-like (Ranbp3l)	4.06
Sertad1	SERTA domain containing 1 (Sertad1)	4.05
Akap12	A kinase (PRKA) anchor protein (gravin) 12 (Akap12)	4.04
Prkcdbp	protein kinase C, delta binding protein (Prkcdbp)	4.04
Aqp8	aquaporin 8 (Aqp8)	4.03
Pecam1	platelet/endothelial cell adhesion molecule 1 (Pecam1), transcript variant 2	4.01
Wfdc1	WAP four-disulfide core domain 1 (Wfdc1)	4.01
Palmd	palmdelphin (Palmd)	3.96
Ehd4	EH-domain containing 4 (Ehd4)	3.95
4930438O05Rik	RIKEN cDNA 4930438O05Rik gene (4930438O05Rik)	3.94
Rem2	rad and gem related GTP binding protein 2 (Rem2)	3.94
1110032E23Rik	RIKEN cDNA 1110032E23 gene (1110032E23Rik)	3.93
Th	tyrosine hydroxylase (Th)	3.93
Cdk5rap1	CDK5 regulatory subunit associated protein 1 (Cdk5rap1)	3.9
Adamts2	a disintegrin-like and metallopeptidase (reprolysin type) with thrombospondin type 1 motif, 2 (Adamts2)	3.89
8430408G22Rik	RIKEN cDNA 8430408G22 gene (8430408G22Rik)	3.88
Deadc1	deaminase domain containing 1 (Deadc1)	3.88

Cd9	CD9 antigen (Cd9)	3.87
Cyr61	cysteine rich protein 61 (Cyr61)	3.87
Pdlim3	PDZ and LIM domain 3 (Pdlim3)	3.87
LOC100046232	similar to NFIL3/E4BP4 transcription factor (LOC100046232)	3.85
Mmrn2	multimerin 2 (Mmrn2)	3.85
Nubp2	nucleotide binding protein 2 (Nubp2)	3.85
S100a13	S100 calcium binding protein A13 (S100a13)	3.81
Aebp1	AE binding protein 1 (Aebp1)	3.8
Mfge8	milk fat globule-EGF factor 8 protein (Mfge8), transcript variant 2	3.76
Mfap4	microfibrillar-associated protein 4 (Mfap4)	3.72
Pmp22	peripheral myelin protein (Pmp22)	3.72
Ptn	pleiotrophin (Ptn)	3.72
Mylk	myosin, light polypeptide kinase (Mylk)	3.7
Vegfc	vascular endothelial growth factor C (Vegfc)	3.7
Tnfaip3	tumor necrosis factor, alpha-induced protein 3 (Tnfaip3)	3.68
Hist1h2ad	histone cluster 1, H2ad (Hist1h2ad)	3.66
Kdelr2	KDEL (Lys-Asp-Glu-Leu) endoplasmic reticulum protein retention receptor 2 (Kdelr2)	3.63
Lmna	lamin A (Lmna), transcript variant 2	3.63
S100a10	S100 calcium binding protein A10 (calpactin) (S100a10)	3.62
2310016C16Rik	RIKEN cDNA 2310016C16 gene (2310016C16Rik)	3.59
Cp	ceruloplasmin (Cp), transcript variant 2	3.55
Itgbl1	integrin, beta-like 1 (Itgbl1)	3.55
Olfml3	olfactomedin-like 3 (Olfml3)	3.54
Kcnk5	potassium channel, subfamily K, member 5 (Kcnk5)	3.53
Hs3st1	heparan sulfate (glucosamine) 3-O-sulfotransferase 1 (Hs3st1)	3.52
LOC100043257	similar to RNA binding motif protein 3 (LOC100043257)	3.51
Loxl1	lysyl oxidase-like 1 (Loxl1)	3.51
Pak3	p21 (CDKN1A)-activated kinase 3 (Pak3)	3.51
Ppp1r3c	protein phosphatase 1, regulatory (inhibitor) subunit 3C (Ppp1r3c)	3.49
Tspan8	tetraspanin 8 (Tspan8)	3.48
Angpt2	angiopoietin 2 (Angpt2)	3.46
Cd81	CD 81 antigen (Cd81)	3.43
Dio1	deiodinase, iodothyronine, type I (Dio1)	3.43
Stmn2	stathmin-like 2 (Stmn2)	3.43
Crp	C-reactive protein, pentraxin-related (Crp)	3.42
Hoxa5	homeobox A5 (Hoxa5)	3.42
Lmna	lamin A (Lmna), transcript variant 1	3.42
Zeb2	zinc finger E-box binding homeobox 2 (Zeb2), transcript variant 2	3.42
Rnd3	Rho family GTPase 3 (Rnd3)	3.4
Tspo	translocator protein (Tspo)	3.4
Ctsa	cathepsin A (Ctsa), transcript variant 2	3.38
Rgs10	regulator of G-protein signalling 10 (Rgs10)	3.38
Hist1h2af	histone cluster 1, H2af (Hist1h2af)	3.37
Itih4	inter alpha-trypsin inhibitor, heavy chain 4 (Itih4)	3.37
Ptp4a2	protein tyrosine phosphatase 4a2 (Ptp4a2)	3.37
Fstl1	follistatin-like 1 (Fstl1)	3.36
Gng10	guanine nucleotide binding protein (G protein), gamma 10 (Gng10)	3.36
Hist1h2ak	histone cluster 1, H2ak (Hist1h2ak)	3.36

Mmp2	matrix metallopeptidase 2 (Mmp2)	3.35
Nuak1	NUAK family, SNF1-like kinase, 1 (Nuak1)	3.34
LOC100047583	similar to apolipoprotein D (LOC100047583)	3.33
Klf2	Kruppel-like factor 2 (lung) (Klf2)	3.32
Lgi1	leucine-rich repeat LGI family, member 1 (Lgi1)	3.32
Phlda1	pleckstrin homology-like domain, family A, member 1 (Phlda1)	3.32
2310016C16Rik	RIKEN cDNA 2310016C16 gene (2310016C16Rik)	3.31
Gcnt1	glucosaminyl (N-acetyl) transferase 1, core 2 (Gcnt1)	3.29
Irf1	interferon regulatory factor 1 (Irf1)	3.27
Gfer	growth factor, erv1 (S. cerevisiae)-like (augmenter of liver regeneration) (Gfer)	3.25
Nicn1	nicolin 1 (Nicn1)	3.25
Aldh3b1	aldehyde dehydrogenase 3 family, member B1 (Aldh3b1)	3.24
Klf4	Kruppel-like factor 4 (gut) (Klf4)	3.23
Flna	filamin, alpha (Flna)	3.22
Selp	selectin, platelet (Selp)	3.22
Thy1	thymus cell antigen 1, theta (Thy1)	3.22
Anxa2	annexin A2 (Anxa2)	3.21
G0s2	G0/G1 switch gene 2 (G0s2)	3.21
Klk8	kallikrein related-peptidase 8 (Klk8)	3.21
4930455C21Rik	RIKEN cDNA 4930455C21 gene (4930455C21Rik)	3.2
Cd14	CD14 antigen (Cd14)	3.2
H2-Eb1	histocompatibility 2, class II antigen E beta (H2-Eb1)	3.2
Mbp	myelin basic protein (Mbp), transcript variant 7	3.2
Papss2	3'-phosphoadenosine 5'-phosphosulfate synthase 2 (Papss2)	3.19
Serpinh1	serine (or cysteine) peptidase inhibitor, clade H, member 1 (Serpinh1)	3.19
Timp2	tissue inhibitor of metalloproteinase 2 (Timp2)	3.18
Hist1h2ah	histone cluster 1, H2ah (Hist1h2ah)	3.17
Gucy1b3	guanylate cyclase 1, soluble, beta 3 (Gucy1b3)	3.16
Hspg2	perlecan (heparan sulfate proteoglycan 2) (Hspg2)	3.15
Hist1h2an	histone cluster 1, H2an (Hist1h2an)	3.14
Bicc1	bicaudal C homolog 1 (Drosophila) (Bicc1)	3.13
Emp3	epithelial membrane protein 3 (Emp3)	3.13
Mxra8	matrix-remodelling associated 8 (Mxra8)	3.12
Nr5a2	nuclear receptor subfamily 5, group A, member 2 (Nr5a2)	3.12
Esam1	endothelial cell-specific adhesion molecule (Esam1)	3.11
Des	desmin (Des)	3.1
Zfp36	zinc finger protein 36 (Zfp36)	3.09
Gja4	gap junction protein, alpha 4 (Gja4)	3.08
Irs2	insulin receptor substrate 2 (Irs2)	3.08
Col18a1	procollagen, type XVIII, alpha 1 (Col18a1)	3.07
Ramp2	receptor (calcitonin) activity modifying protein 2 (Ramp2)	3.07
Vip	vasoactive intestinal polypeptide (Vip)	3.07
Bik	Bcl2-interacting killer (Bik)	3.05
Cdc42ep2	CDC42 effector protein (Rho GTPase binding) 2 (Cdc42ep2)	3.05
Ppap2a	phosphatidic acid phosphatase 2a (Ppap2a), transcript variant 1	3.05
Rin2	Ras and Rab interactor 2 (Rin2)	3.05
Lrrc1	leucine rich repeat containing 1 (Lrrc1)	3.04
Pyy	peptide YY (Pyy)	3.04

Cd3d	CD3 antigen, delta polypeptide (Cd3d)	3.02
Htra1	HtrA serine peptidase 1 (Htra1)	3.02
Dbi	diazepam binding inhibitor (Dbi), transcript variant 2	3.01
Gm2a	GM2 ganglioside activator protein (Gm2a)	3.01
Zbtb2	zinc finger and BTB domain containing 2 (Zbtb2)	3
Rhoj	ras homolog gene family, member J (Rhoj)	2.99
Skil	SKI-like (Skil), transcript variant 2	2.99
Lmna	lamin A (Lmna), transcript variant 2	2.96
Nkx2-3	NK2 homeobox 3 (Nkx2-3)	2.95
Hist1h2ao	histone cluster 1, H2ao (Hist1h2ao)	2.94
Slco2a1	solute carrier organic anion transporter family, member 2a1 (Slco2a1)	2.94
Ccl5	chemokine (C-C motif) ligand 5 (Ccl5)	2.93
Map3k8	mitogen-activated protein kinase kinase kinase 8 (Map3k8)	2.93
P2rx1	purinergic receptor P2X, ligand-gated ion channel, 1 (P2rx1)	2.93
Tgfbr2	transforming growth factor, beta receptor II (Tgfbr2), transcript variant 1	2.93
Lamb2	laminin, beta 2 (Lamb2)	2.91
MGC41689	MGC41689	2.91
Accn2	amiloride-sensitive cation channel 2, neuronal (Accn2)	2.89
Car4	carbonic anhydrase 4 (Car4)	2.89
Cenpa	centromere protein A (Cenpa)	2.89
2310016C08Rik	RIKEN cDNA 2310016C08 gene (2310016C08Rik)	2.88
Arrdc4	arrestin domain containing 4 (Arrdc4), transcript variant 1	2.88
Higd1b	HIG1 domain family, member 1B (Higd1b)	2.88
LOC100046254	similar to Adi1 protein (LOC100046254)	2.88
Emcn	endomucin (Emcn)	2.86
Tpm1	tropomyosin 1, alpha (Tpm1)	2.86
3110001A13Rik	RIKEN cDNA 3110001A13 gene (3110001A13Rik)	2.85
Cdc42ep3	CDC42 effector protein (Rho GTPase binding) 3 (Cdc42ep3)	2.85
Fxyd5	FXYD domain-containing ion transport regulator 5 (Fxyd5)	2.85
Gstt1	glutathione S-transferase, theta 1 (Gstt1)	2.85
3110009E18Rik	RIKEN cDNA 3110009E18 gene (3110009E18Rik)	2.84
Kctd10	potassium channel tetramerisation domain containing 10 (Kctd10)	2.84
BC029169	cDNA sequence BC029169 (BC029169)	2.82
Ccdc23	coiled-coil domain containing 23 (Ccdc23), transcript variant 1	2.82
Cxcl12	chemokine (C-X-C motif) ligand 12 (Cxcl12)	2.82
Pdzrn3	PDZ domain containing RING finger 3 (Pdzrn3)	2.81
Pkia	protein kinase inhibitor, alpha (Pkia)	2.81
Hdc	histidine decarboxylase (Hdc)	2.8
Lims2	LIM and senescent cell antigen like domains 2 (Lims2)	2.8
Myd116	myeloid differentiation primary response gene 116 (Myd116)	2.8
Ctsh	cathepsin H (Ctsh)	2.79
Dpt	dermatopontin (Dpt)	2.79
Rras	Harvey rat sarcoma oncogene, subgroup R (Rras)	2.79
Svop	SV2 related protein (Svop)	2.79
Efemp2	epidermal growth factor-containing fibulin-like extracellular matrix protein 2 (Efemp2)	2.78
B2m	beta-2 microglobulin (B2m)	2.77
Bex4	brain expressed gene 4 (Bex4)	2.77
Elf3	E74-like factor 3 (Elf3)	2.77

Nipsnap1	4-nitrophenylphosphatase domain and non-neuronal SNAP25-like protein homolog 1 (C. elegans) (Nipsnap1)	2.77
Upb1	ureidopropionase, beta (Upb1)	2.77
Gsto2	glutathione S-transferase omega 2 (Gsto2)	2.76
Il6st	interleukin 6 signal transducer (Il6st)	2.76
Rbpms	RNA binding protein gene with multiple splicing (Rbpms), transcript variant 3	2.76
Cd151	CD151 antigen (Cd151)	2.75
Dcps	decapping enzyme, scavenger (Dcps)	2.74
Defb1	defensin beta 1 (Defb1)	2.74
Krt23	keratin 23 (Krt23)	2.74
Otoa	otoancorin (Otoa)	2.74
Tppp3	tubulin polymerization-promoting protein family member 3 (Tppp3)	2.74
Egr2	early growth response 2 (Egr2)	2.73
Ppap2a	phosphatidic acid phosphatase 2a (Ppap2a), transcript variant 2	2.73
Prkd3	protein kinase D3 (Prkd3)	2.73
Gas6	growth arrest specific 6 (Gas6)	2.71
F2r	coagulation factor II (thrombin) receptor (F2r)	2.7
Olfml2b	olfactomedin-like 2B (Olfml2b)	2.7
2310002B06Rik	RIKEN cDNA 2310002B06 gene (2310002B06Rik)	2.67
Arhgdib	Rho, GDP dissociation inhibitor (GDI) beta (Arhgdib)	2.67
Serpina7	serine (or cysteine) peptidase inhibitor, clade A (alpha-1 antiproteinase, antitrypsin), member 7 (Serpina7)	2.67
St3gal6	ST3 beta-galactoside alpha-2,3-sialyltransferase 6 (St3gal6)	2.67
Tmod1	tropomodulin 1 (Tmod1)	2.67
Myl6	myosin, light polypeptide 6, alkali, smooth muscle and non-muscle (Myl6)	2.66
Hmgb2	high mobility group box 2 (Hmgb2)	2.65
Xpa	xeroderma pigmentosum, complementation group A (Xpa)	2.63
Dnaic1	dynein, axonemal, intermediate chain 1 (Dnaic1)	2.62
Hey1	hairy/enhancer-of-split related with YRPW motif 1 (Hey1)	2.62
Itgb3bp	integrin beta 3 binding protein (beta3-endonexin) (Itgb3bp)	2.61
Plscr1	phospholipid scramblase 1 (Plscr1)	2.61
Slc2a6	solute carrier family 2 (facilitated glucose transporter), member 6 (Slc2a6)	2.61
Vdr	vitamin D receptor (Vdr)	2.61
Cdkn1c	cyclin-dependent kinase inhibitor 1C (P57) (Cdkn1c)	2.6
Golm1	golgi membrane protein 1 (Golm1), transcript variant 1	2.6
Hexim1	hexamethylene bis-acetamide inducible 1 (Hexim1)	2.59
LOC100047173	similar to synaptotagmin-like 1 (LOC100047173), misc RNA.	2.59
Slc10a6	solute carrier family 10 (sodium/bile acid cotransporter family), member 6 (Slc10a6)	2.59
Chgb	chromogranin B (Chgb)	2.58
Ebpl	emopamil binding protein-like (Ebpl)	2.58
Rassf3	Ras association (RalGDS/AF-6) domain family member 3 (Rassf3)	2.58
Trex1	three prime repair exonuclease 1 (Trex1), transcript variant 2	2.58
Lpl	lipoprotein lipase (Lpl)	2.57
Mmp14	matrix metallopeptidase 14 (membrane-inserted) (Mmp14)	2.56
Tgfb3	transforming growth factor, beta 3 (Tgfb3)	2.56
Wfdc15b	WAP four-disulfide core domain 15B (Wfdc15b), transcript variant 1	2.56
Enpp6	ectonucleotide pyrophosphatase/phosphodiesterase 6 (Enpp6)	2.55
Gpc3	glypican 3 (Gpc3)	2.55
H2-Aa	histocompatibility 2, class II antigen A, alpha (H2-Aa)	2.55
Rasl12	RAS-like, family 12 (Rasl12)	2.55

Tcte3	t-complex-associated testis expressed 3 (Tcte3), transcript variant 2	2.55
Dctn3	dynactin 3 (Dctn3)	2.54
Elovl5	ELOVL family member 5, elongation of long chain fatty acids (yeast) (Elovl5)	2.54
Gcap14	granule cell antiserum positive 14 (Gcap14), transcript variant 2	2.54
Bex2	brain expressed X-linked 2 (Bex2)	2.53
Gngt2	guanine nucleotide binding protein (G protein), gamma transducing activity polypeptide 2 (Gngt2), transcript variant 2	2.53
2310016C16Rik	RIKEN cDNA 2310016C16 gene (2310016C16Rik)	2.52
Rbms3	RNA binding motif, single stranded interacting protein (Rbms3)	2.51
Cxx1c	CAAX box 1 homolog C (human) (Cxx1c)	2.5
E430002G05Rik	RIKEN cDNA E430002G05 gene (E430002G05Rik)	2.5
Hyal2	hyaluronoglucosaminidase 2 (Hyal2)	2.5
Spnb2	spectrin beta 2 (Spnb2), transcript variant 2	2.5
C2	complement component 2 (within H-2S) (C2)	2.49
Cdkn1a	cyclin-dependent kinase inhibitor 1A (P21) (Cdkn1a)	2.49
Fis1	fission 1 (mitochondrial outer membrane) homolog (yeast) (Fis1)	2.49
Plscr4	phospholipid scramblase 4 (Plscr4)	2.49
Cxcl16	chemokine (C-X-C motif) ligand 16 (Cxcl16)	2.48
Ffar2	free fatty acid receptor 2 (Ffar2)	2.48
Gadd45a	growth arrest and DNA-damage-inducible 45 alpha (Gadd45a)	2.48
Lgals9	lectin, galactose binding, soluble 9 (Lgals9)	2.48
Golm1	golgi membrane protein 1 (Golm1), transcript variant 2	2.47
Nos3	nitric oxide synthase 3, endothelial cell (Nos3)	2.47
Zfp608	zinc finger protein 608 (Zfp608)	2.47
Jmjd3	jumonji domain containing 3 (Jmjd3)	2.45
Lgi1	leucine-rich repeat LGI family, member 1 (Lgi1)	2.45
Ctf1	cardiotrophin 1 (Ctf1)	2.44
Fhl1	four and a half LIM domains 1 (Fhl1), transcript variant 1	2.44
Podxl2	podocalyxin-like 2 (Podxl2)	2.44
D330028D13Rik	RIKEN cDNA D330028D13 gene (D330028D13Rik)	2.43
Col6a1	procollagen, type VI, alpha 1 (Col6a1)	2.41
Aplp2	amyloid beta (A4) precursor-like protein 2 (Aplp2), transcript variant 3	2.4
2700094K13Rik	RIKEN cDNA 2700094K13 gene (2700094K13Rik), transcript variant 2	2.39
Rasgrp3	RAS, guanyl releasing protein 3 (Rasgrp3)	2.39
Lhfp	lipoma HMGIC fusion partner (Lhfp)	2.38
LOC100047427	similar to thyroid hormone receptor (LOC100047427)	2.38
Ltbp4	latent transforming growth factor beta binding protein 4 (Ltbp4)	2.38
Tgfbr3	transforming growth factor, beta receptor III (Tgfbr3)	2.38
Ugt2b34	UDP glucuronosyltransferase 2 family, polypeptide B34 (Ugt2b34)	2.38
Pros1	protein S (alpha) (Pros1)	2.37
Acaa2	acetyl-Coenzyme A acyltransferase 2 (mitochondrial 3-oxoacyl-Coenzyme A thiolase) (Acaa2)	2.36
Cd93	CD93 antigen (Cd93)	2.36
5730437N04Rik	RIKEN cDNA 5730437N04 gene (5730437N04Rik)	2.35
Cygb	cytoglobin (Cygb)	2.34
Ddc	dopa decarboxylase (Ddc)	2.34
0610040J01Rik	RIKEN cDNA 0610040J01 gene (0610040J01Rik)	2.33
Cat	catalase (Cat)	2.33
Edg2	endothelial differentiation, lysophosphatidic acid G-protein-coupled receptor, 2 (Edg2)	2.33
Gdf10	growth differentiation factor 10 (Gdf10)	2.33

C3	complement component 3 (C3)	2.32
Elk3	ELK3, member of ETS oncogene family (Elk3), transcript variant 2	2.32
Socs3	suppressor of cytokine signaling 3 (Socs3)	2.32
3110004L20Rik	RIKEN cDNA 3110004L20 gene (3110004L20Rik)	2.31
Ccnl1	cyclin L1 (Ccnl1)	2.31
Stard4	StAR-related lipid transfer (START) domain containing 4 (Stard4)	2.31
Acadm	acyl-Coenzyme A dehydrogenase, medium chain (Acadm)	2.3
Maged2	melanoma antigen, family D, 2 (Maged2)	2.3
Sult1c2	sulfotransferase family, cytosolic, 1C, member 2 (Sult1c2)	2.3
Abca8a	ATP-binding cassette, sub-family A (ABC1), member 8a (Abca8a)	2.29
Cd59a	CD59a antigen (Cd59a)	2.29
Ephx1	epoxide hydrolase 1, microsomal (Ephx1)	2.29
Gimap6	GTPase, IMAP family member 6 (Gimap6)	2.29
Snx30	sorting nexin family member 30 (Snx30)	2.29
Thra	thyroid hormone receptor alpha (Thra)	2.29
Col6a1	procollagen, type VI, alpha 1 (Col6a1)	2.28
Cdc42ep4	CDC42 effector protein (Rho GTPase binding) 4 (Cdc42ep4)	2.27
Igfbp7	insulin-like growth factor binding protein 7 (Igfbp7)	2.27
Pdia6	protein disulfide isomerase associated 6 (Pdia6)	2.27
Slc41a3	solute carrier family 41, member 3 (Slc41a3), transcript variant 2	2.27
Hspb8	heat shock protein 8 (Hspb8)	2.26
LOC100040592	similar to Hmgcs1 protein, transcript variant 1 (LOC100040592)	2.26
Dynll1	dynein light chain LC8-type 1 (Dynll1)	2.25
Gpx3	glutathione peroxidase 3 (Gpx3), transcript variant 2	2.25
Mylc2b	myosin light chain, regulatory B (Mylc2b)	2.25
Nfib	nuclear factor I/B (Nfib)	2.25
Smarce1	SWI/SNF related, matrix associated, actin dependent regulator of chromatin, subfamily e, member 1 (Smarce1)	2.25
Clk4	CDC like kinase 4 (Clk4)	2.24
Grk5	G protein-coupled receptor kinase 5 (Grk5)	2.24
Casp4	caspase 4, apoptosis-related cysteine peptidase (Casp4)	2.23
Gm889	gene model 889, (NCBI) (Gm889)	2.23
Ifitm2	interferon induced transmembrane protein 2 (Ifitm2)	2.23
2010011I20Rik	RIKEN cDNA 2010011I20 gene (2010011I20Rik)	2.22
Adcy4	adenylate cyclase 4 (Adcy4)	2.22
Col5a1	procollagen, type V, alpha 1 (Col5a1)	2.22
Blvrb	biliverdin reductase B (flavin reductase (NADPH)) (Blvrb)	2.21
Nr1d1	nuclear receptor subfamily 1, group D, member 1 (Nr1d1)	2.21
Snx24	sorting nexing 24 (Snx24)	2.21
Atp6v1g1	ATPase, H+ transporting, lysosomal V1 subunit G1 (Atp6v1g1)	2.2
Tgm2	transglutaminase 2, C polypeptide (Tgm2)	2.2
Jam2	junction adhesion molecule 2 (Jam2)	2.19
Siva1	SIVA1, apoptosis-inducing factor (Siva1)	2.19
Eng	endoglin (Eng)	2.18
Hagh	hydroxyacyl glutathione hydrolase (Hagh)	2.18
Hexb	hexosaminidase B (Hexb)	2.18
Ldb2	LIM domain binding 2 (Ldb2), transcript variant 2	2.18
Gpr120	G protein-coupled receptor 120 (Gpr120)	2.17
Itgb1bp1	integrin beta 1 binding protein 1 (Itgb1bp1)	2.17

Lefty1	left right determination factor 1 (Lefty1)	2.17
Robo4	roundabout homolog 4 (Drosophila) (Robo4)	2.17
Ambp	alpha 1 microglobulin/bikunin (Ambp)	2.16
D12Ertd553e	DNA segment, Chr 12, ERATO Doi 553, expressed (D12Ertd553e)	2.16
Rpo2tc1	SUB1 homolog (S. cerevisiae) (Rpo2tc1)	2.16
B9d1	B9 protein domain 1 (B9d1)	2.15
Fhl1	four and a half LIM domains 1 (Fhl1), transcript variant 2	2.15
Gja1	gap junction membrane channel protein alpha 1 (Gja1)	2.15
Lhfpl2	lipoma HMGIC fusion partner-like 2 (Lhfpl2)	2.15
Plp1	proteolipid protein (myelin) 1 (Plp1)	2.15
Sep15	selenoprotein (Sep15)	2.14
Dkk3	dickkopf homolog 3 (Xenopus laevis) (Dkk3)	2.14
Pcolce	procollagen C-endopeptidase enhancer protein (Pcolce)	2.14
Pea15	phosphoprotein enriched in astrocytes 15 (Pea15)	2.14
Phca	phytoceramidase, alkaline (Phca)	2.14
Ube2e2	ubiquitin-conjugating enzyme E2E 2 (UBC4/5 homolog, yeast) (Ube2e2)	2.14
Ces3	carboxylesterase 3 (Ces3)	2.13
Scd2	stearoyl-Coenzyme A desaturase 2 (Scd2)	2.13
Slc2a1	solute carrier family 2 (facilitated glucose transporter), member 1 (Slc2a1)	2.13
Stmn3	stathmin-like 3 (Stmn3)	2.13
Asah3l	N-acylsphingosine amidohydrolase 3-like (Asah3l)	2.12
Ifi27	interferon, alpha-inducible protein 27 (Ifi27)	2.12
Lgmn	legumain (Lgmn)	2.12
Mns1	meiosis-specific nuclear structural protein 1 (Mns1)	2.12
Tmsb4x	thymosin, beta 4, X chromosome (Tmsb4x)	2.12
Pdgfrl	platelet-derived growth factor receptor-like (Pdgfrl)	2.11
Pls3	plastin 3 (T-isoform) (Pls3)	2.11
Cdh5	cadherin 5 (Cdh5)	2.1
Rbpms	RNA binding protein gene with multiple splicing (Rbpms), transcript variant 1	2.1
Rnu6	U6 small nuclear RNA (Rnu6).	2.1
Oat	ornithine aminotransferase (Oat)	2.09
Pdrg1	p53 and DNA damage regulated 1 (Pdrg1)	2.09
Rbm35a	RNA binding motif protein 35A (Rbm35a)	2.09
Aqp11	aquaporin 11 (Aqp11)	2.08
Hspa2	heat shock protein 2 (Hspa2), transcript variant 1	2.08
Slc11a1	solute carrier family 11 (proton-coupled divalent metal ion transporters), member 1 (Slc11a1)	2.08
Slc12a7	solute carrier family 12, member 7 (Slc12a7)	2.08
Hmgn2	high mobility group nucleosomal binding domain 2 (Hmgn2)	2.07
Osgin1	oxidative stress induced growth inhibitor 1 (Osgin1)	2.07
Pdia4	protein disulfide isomerase associated 4 (Pdia4)	2.07
Pitpnm3	PITPNM family member 3 (Pitpnm3), transcript variant 1	2.07
Vps33a	vacuolar protein sorting 33A (yeast) (Vps33a)	2.07
Lrrc26	leucine rich repeat containing 26 (Lrrc26)	2.06
Ppap2a	phosphatidic acid phosphatase 2a (Ppap2a), transcript variant 1	2.06
Sdcbp2	syndecan binding protein (syntenin) 2 (Sdcbp2)	2.06
Cd52	CD52 antigen (Cd52)	2.05
D8Ertd738e	DNA segment, Chr 8, ERATO Doi 738, expressed (D8Ertd738e)	2.05
Dmrtc1a	DMRT-like family C1a (Dmrtc1a), transcript variant 3	2.05

Napepld	N-acyl phosphatidylethanolamine phospholipase D (Napepld)	2.05
Pfn1	profilin 1 (Pfn1)	2.05
S100a1	S100 calcium binding protein A1 (S100a1)	2.05
Scarf2	scavenger receptor class F, member 2 (Scarf2)	2.05
1190017O12Rik	RIKEN cDNA 1190017O12 gene (1190017O12Rik)	2.04
Col4a2	collagen, type IV, alpha 2 (Col4a2)	2.04
Ctnnbip1	catenin beta interacting protein 1 (Ctnnbip1)	2.04
Serping1	serine (or cysteine) peptidase inhibitor, clade G, member 1 (Serping1)	2.04
Slc29a1	solute carrier family 29 (nucleoside transporters), member 1 (Slc29a1)	2.04
Tmpo	thymopoietin (Tmpo), transcript variant 6	2.04
Tsc22d2	TSC22 domain family 2 (Tsc22d2)	2.04
Calu	calumenin (Calu), transcript variant 1	2.03
Chic1	cysteine-rich hydrophobic domain 1 (Chic1)	2.03
Eltd1	EGF, latrophilin seven transmembrane domain containing 1 (Eltd1)	2.03
Padi2	peptidyl arginine deiminase, type II (Padi2)	2.03
Pdlim4	PDZ and LIM domain 4 (Pdlim4)	2.03
Pik3r3	phosphatidylinositol 3 kinase, regulatory subunit, polypeptide 3 (p55) (Pik3r3)	2.03
Tmem204	transmembrane protein 204 (Tmem204)	2.03
Atf3	activating transcription factor 3 (Atf3)	2.02
Sox7	SRY-box containing gene 7 (Sox7)	2.02
Vgf	VGF nerve growth factor inducible (Vgf)	2.02
Atox1	ATX1 (antioxidant protein 1) homolog 1 (yeast) (Atox1)	2.01
Bhlhb2	basic helix-loop-helix domain containing, class B2 (Bhlhb2)	2.01
Dph4	DPH4 homolog (JJJ3, S. cerevisiae) (Dph4)	2.01
Eno1	enolase 1, alpha non-neuron (Eno1)	2.01
Hhex	hematopoietically expressed homeobox (Hhex)	2.01
Hyi	hydroxypyruvate isomerase homolog (E. coli) (Hyi)	2.01
Pip4k2a	phosphatidylinositol-5-phosphate 4-kinase, type II, alpha (Pip4k2a)	2.01
Rdbp	RD RNA-binding protein (Rdbp), transcript variant 3	2.01
Zfp46	zinc finger protein 46 (Zfp46)	2.01
Chrnb1	cholinergic receptor, nicotinic, beta polypeptide 1 (muscle) (Chrnb1)	2
Klhl6	kelch-like 6 (Drosophila) (Klhl6)	2
Pam	peptidylglycine alpha-amidating monooxygenase (Pam)	2
Fxyd3	FXYD domain-containing ion transport regulator 3 (Fxyd3)	1.98
Lrrc33	leucine rich repeat containing 33 (Lrrc33)	1.98
Necab2	N-terminal EF-hand calcium binding protein 2 (Necab2)	1.98
Npas4	neuronal PAS domain protein 4 (Npas4)	1.98
3110050N22Rik	RIKEN cDNA 3110050N22 gene (3110050N22Rik)	1.97
Col4a1	procollagen, type IV, alpha 1 (Col4a1)	1.97
Sox5	SRY-box containing gene 5 (Sox5)	1.97
Swap70	SWA-70 protein (Swap70)	1.97
1810021J13Rik	RIKEN cDNA 1810021J13 gene (1810021J13Rik)	1.96
Suhw4	suppressor of hairy wing homolog 4 (Drosophila) (Suhw4)	1.96
Vkorc1	vitamin K epoxide reductase complex, subunit 1 (Vkorc1)	1.96
Cryl1	crystallin, lambda 1 (Cryl1)	1.95
Etfdh	electron transferring flavoprotein, dehydrogenase (Etfdh)	1.95
Ly6a	lymphocyte antigen 6 complex, locus A (Ly6a)	1.95
Mcam	melanoma cell adhesion molecule (Mcam)	1.95

March2	membrane-associated ring finger (C3HC4) 2 (March2)	1.94
Capg	capping protein (actin filament), gelsolin-like (Capg), transcript variant 1	1.94
Cish	cytokine inducible SH2-containing protein (Cish)	1.93
Coro1a	coronin, actin binding protein 1A (Coro1a)	1.93
Eif2ak2	eukaryotic translation initiation factor 2-alpha kinase 2 (Eif2ak2)	1.93
Hes1	hairy and enhancer of split 1 (Drosophila) (Hes1)	1.93
S100a11	S100 calcium binding protein A11 (calgizzarin) (S100a11)	1.93
Art3	ADP-ribosyltransferase 3 (Art3)	1.92
Ccdc126	coiled-coil domain containing 126 (Ccdc126)	1.92
Cd53	CD53 antigen (Cd53)	1.92
Ctnnbl1	catenin, beta like 1 (Ctnnbl1)	1.92
Hemk1	HemK methyltransferase family member 1 (Hemk1)	1.92
Wrn	Werner syndrome homolog (human) (Wrn)	1.92
Cirbp	cold inducible RNA binding protein (Cirbp)	1.91
Pdzk1ip1	PDZK1 interacting protein 1 (Pdzk1ip1)	1.91
Tspan12	tetraspanin 12 (Tspan12)	1.91
Sar1b	SAR1 gene homolog B (S. cerevisiae) (Sar1b)	1.9
Arl4a	ADP-ribosylation factor-like 4A (Arl4a), transcript variant 1	1.89
Il18	interleukin 18 (Il18)	1.89
LOC100046883	similar to CKLF-like MARVEL transmembrane domain containing 3 (LOC100046883)	1.89
Mras	muscle and microspikes RAS (Mras)	1.89
Nrarp	Notch-regulated ankyrin repeat protein (Nrarp)	1.89
Rbms1	RNA binding motif, single stranded interacting protein 1 (Rbms1)	1.89
Add3	adducin 3 (gamma) (Add3)	1.88
Apof	apolipoprotein F (Apof)	1.88
Nme3	non-metastatic cells 3, protein expressed in (Nme3)	1.88
Ostf1	osteoclast stimulating factor 1 (Ostf1)	1.88
Rnps1	ribonucleic acid binding protein S1 (Rnps1)	1.88
Spsb1	splA/ryanodine receptor domain and SOCS box containing 1 (Spsb1)	1.88
Zmpste24	zinc metallopeptidase, STE24 homolog (S. cerevisiae) (Zmpste24)	1.88
Crip2	cysteine rich protein 2 (Crip2)	1.87
Dscr1	regulator of calcineurin 1 (Dscr1)	1.87
Gpd2	glycerol phosphate dehydrogenase 2, mitochondrial (Gpd2), nuclear gene encoding mitochondrial protein	1.87
Ier3ip1	immediate early response 3 interacting protein 1 (Ier3ip1)	1.87
Ifngr1	interferon gamma receptor 1 (Ifngr1)	1.87
Prox1	prospero-related homeobox 1 (Prox1)	1.87
6720467C03Rik	RIKEN cDNA 6720467C03 gene (6720467C03Rik)	1.86
Cd97	CD97 antigen (Cd97)	1.86
Ednra	endothelin receptor type A (Ednra)	1.86
Gcnt2	glucosaminyl (N-acetyl) transferase 2, I-branching enzyme (Gcnt2), transcript variant 3	1.86
Krt19	keratin 19 (Krt19)	1.86
LOC100048301	similar to RNA Polymerase II subunit 14.5 kD (LOC100048301)	1.86
1700023M03Rik	RIKEN 1700023M03Rik cDNA (1700023M03Rik)	1.85
Atp6v0e2	ATPase, H+ transporting, lysosomal V0 subunit E2 (Atp6v0e2)	1.85
Dpep1	dipeptidase 1 (renal) (Dpep1)	1.85
LOC100045567	similar to purine nucleoside phosphorylase (LOC100045567)	1.85
Nbl1	neuroblastoma, suppression of tumorigenicity 1 (Nbl1)	1.85
Zc3h3	zinc finger CCCH type containing 3 (Zc3h3)	1.85

2210023G05Rik	RIKEN cDNA 2210023G05 gene (2210023G05Rik)	1.84
Adam17	a disintegrin and metallopeptidase domain 17 (Adam17)	1.84
Efcab1	EF hand calcium binding domain 1 (Efcab1)	1.84
LOC100044177	hypothetical protein LOC100044177 (LOC100044177)	1.84
Tes	testis derived transcript (Tes), transcript variant 1	1.84
Uxt	ubiquitously expressed transcript (Uxt)	1.84
1110067D22Rik	RIKEN cDNA 1110067D22 gene (1110067D22Rik)	1.83
1700011H14Rik	RIKEN cDNA 1700011H14 gene (1700011H14Rik)	1.83
Ankrd56	ankyrin repeat domain 56 (Ankrd56)	1.83
Anxa4	annexin A4 (Anxa4)	1.83
Arpc3	actin related protein 2/3 complex, subunit 3 (Arpc3)	1.83
Bex2	brain expressed X-linked 2 (Bex2)	1.83
Col15a1	collagen, type XV, alpha 1 (Col15a1)	1.83
Nedd9	neural precursor cell expressed, developmentally down-regulated gene 9 (Nedd9)	1.83
Gimap1	GTPase, IMAP family member 1 (Gimap1), transcript variant 1	1.82
Osbpl9	oxysterol binding protein-like 9 (Osbpl9), transcript variant 2	1.82
Rnf11	ring finger protein 11 (Rnf11)	1.82
Tbc1d22a	TBC1 domain family, member 22a (Tbc1d22a)	1.82
Wars2	tryptophanyl tRNA synthetase 2 (mitochondrial) (Wars2), nuclear gene encoding mitochondrial protein	1.82
Axl	AXL receptor tyrosine kinase (Axl)	1.81
Lass5	LAG1 homolog, ceramide synthase 5 (Lass5)	1.81
Tnfrsf11b	tumor necrosis factor receptor superfamily, member 11b (osteoprotegerin) (Tnfrsf11b)	1.81
Zfp292	zinc finger protein 292, transcript variant 4 (Zfp292)	1.81
Dad1	defender against cell death 1 (Dad1)	1.8
Dazap2	DAZ associated protein 2 (Dazap2)	1.8
Enc1	ectodermal-neural cortex 1 (Enc1)	1.8
Lhfp	lipoma HMGIC fusion partner (Lhfp)	1.8
Use1	unconventional SNARE in the ER 1 homolog (S. cerevisiae) (Use1), transcript variant 1	1.8
1700012H17Rik	RIKEN cDNA 1700012H17 gene (1700012H17Rik)	1.79
Mgst3	microsomal glutathione S-transferase 3 (Mgst3)	1.79
Obrgrp	leptin receptor (Lepr)	1.79
Prkra	protein kinase, interferon inducible double stranded RNA dependent activator (Prkra)	1.79
Rwdd1	RWD domain containing 1 (Rwdd1)	1.79
Supt4h2	suppressor of Ty 4 homolog 2 (S. cerevisiae) (Supt4h2)	1.79
Cryba2	crystallin, beta A2 (Cryba2)	1.78
Dcx	doublecortin (Dcx), transcript variant 4	1.78
Lrrc28	leucine rich repeat containing 28 (Lrrc28)	1.78
Med30	mediator complex subunit 30 (Med30)	1.78
Trib3	tribbles homolog 3 (Drosophila) (Trib3)	1.78
Crmp1	collapsin response mediator protein 1 (Crmp1)	1.77
Ctsz	cathepsin Z (Ctsz)	1.77
Abcb4	ATP-binding cassette, sub-family B (MDR/TAP), member 4 (Abcb4)	1.76
BC031353	cDNA sequence BC031353 (BC031353)	1.76
Hmgcl	3-hydroxy-3-methylglutaryl-Coenzyme A lyase (Hmgcl)	1.76
Polr2d	polymerase (RNA) II (DNA directed) polypeptide D (Polr2d), transcript variant 1	1.76
Scg2	secretogranin II (Scg2)	1.76
Trip6	thyroid hormone receptor interactor 6 (Trip6)	1.76
Ech1	enoyl coenzyme A hydratase 1, peroxisomal (Ech1)	1.75

Gpx1	glutathione peroxidase 1 (Gpx1)	1.75
Ly6c1	lymphocyte antigen 6 complex, locus C1 (Ly6c1)	1.75
Mapk6	mitogen-activated protein kinase 6 (Mapk6), transcript variant 2	1.75
Trex1	three prime repair exonuclease 1 (Trex1), transcript variant 1	1.75
Trim47	tripartite motif-containing 47 (Trim47)	1.75
2010111I01Rik	RIKEN cDNA 2010111I01 gene (2010111I01Rik)	1.74
Ace2	angiotensin I converting enzyme (peptidyl-dipeptidase A) 2 (Ace2)	1.74
C4b	complement component 4B (Childo blood group) (C4b)	1.74
Mospd3	motile sperm domain containing 3 (Mospd3)	1.74
Nfkbib	nuclear factor of kappa light polypeptide gene enhancer in B cells inhibitor, beta (Nfkbib)	1.74
Scotin	scotin gene (Scotin), transcript variant 1	1.74
Syf2	SYF2 homolog, RNA splicing factor (S. cerevisiae) (Syf2)	1.74
Tuft1	tuftelin 1 (Tuft1)	1.74
Aldoa	aldolase 1, A isoform (Aldoa)	1.73
Smtn	smoothelin (Smtn)	1.73
Hsd17b11	hydroxysteroid (17-beta) dehydrogenase 11 (Hsd17b11)	1.72
Vgll4	vestigial like 4 (Drosophila) (Vgll4)	1.72
2410018G20Rik	RIKEN cDNA 2410018G20 gene (2410018G20Rik)	1.71
5133400G04Rik	RIKEN cDNA 5133400G04 gene (5133400G04Rik), transcript variant 2	1.71
Cyp51	cytochrome P450, family 51 (Cyp51)	1.71
Hn1	hematological and neurological expressed sequence 1 (Hn1)	1.71
Mapk8ip1	mitogen activated protein kinase 8 interacting protein 1 (Mapk8ip1)	1.71
Mrpl11	mitochondrial ribosomal protein L11 (Mrpl11), nuclear gene encoding mitochondrial protein	1.71
Prf1	perforin 1 (pore forming protein) (Prf1)	1.71
Vasn	vasorin (Vasn)	1.71
Hes6	hairy and enhancer of split 6 (Drosophila) (Hes6)	1.7
Cryzl1	crystallin, zeta (quinone reductase)-like 1 (Cryzl1)	1.69
EG622339	predicted gene, EG622339 (EG622339)	1.69
Snrpg	small nuclear ribonucleoprotein polypeptide G (Snrpg)	1.69
1700019H03Rik	RIKEN cDNA 1700019H03 gene (1700019H03Rik)	1.68
Elovl4	elongation of very long chain fatty acids (FEN1/Elo2, SUR4/Elo3, yeast)-like 4 (Elovl4)	1.68
Npy	neuropeptide Y (Npy)	1.68
Sspn	sarcospan (Sspn)	1.68
Tspan14	tetraspanin 14 (Tspan14)	1.68
Add1	adducin 1 (alpha) (Add1), transcript variant 1	1.67
Dapk2	death-associated kinase 2 (Dapk2)	1.67
Dtd1	D-tyrosyl-tRNA deacylase 1 homolog (S. cerevisiae) (Dtd1)	1.67
Ednrb	endothelin receptor type B (Ednrb)	1.67
Lrrc49	leucine rich repeat containing 49 (Lrrc49)	1.67
Crtap	cartilage associated protein (Crtap)	1.66
Dlg3	discs, large homolog 3 (Drosophila) (Dlg3)	1.66
Fbxo2	F-box protein 2 (Fbxo2)	1.66
Hmgcl	3-hydroxy-3-methylglutaryl-Coenzyme A lyase (Hmgcl)	1.66
Il1b	interleukin 1 beta (Il1b)	1.66
Akr1c19	aldo-keto reductase family 1, member C19 (Akr1c19)	1.65
Capn5	calpain 5 (Capn5)	1.65
Casp1	caspase 1 (Casp1)	1.65
Snx12	sorting nexin 12 (Snx12)	1.65

Snx6	sorting nexin 6 (Snx6)	1.65
Adh1	alcohol dehydrogenase 1 (class I) (Adh1)	1.64
Casp6	caspase 6 (Casp6)	1.64
ENSMUSG00000043795	predicted gene, ENSMUSG00000043795 (ENSMUSG00000043795)	1.64
LOC100048589	similar to CDNA sequence BC052040, transcript variant 1 (LOC100048589)	1.64
Pcdh17	protocadherin 17 (Pcdh17)	1.64
Scp2	sterol carrier protein 2, liver (Scp2)	1.64
Sema5a	sema domain, seven thrombospondin repeats (type 1 and type 1-like), transmembrane domain (TM) and short cytoplasmic domain, (semaphorin) 5A (Sema5a)	1.64
AB112350	cDNA sequence AB112350 (AB112350)	1.63
Cyb5r3	cytochrome b5 reductase 3 (Cyb5r3)	1.63
Efna1	ephrin A1 (Efna1)	1.63
Gm1821	gene model 1821, (NCBI) (Gm1821) on chromosome 14.	1.63
H3f3b	H3 histone, family 3B (H3f3b)	1.63
Myt1	myelin transcription factor 1 (Myt1)	1.63
Uaca	uveal autoantigen with coiled-coil domains and ankyrin repeats (Uaca)	1.63
Zfp667	zinc finger protein 667 (Zfp667)	1.63
2310036O22Rik	RIKEN cDNA 2310036O22 gene (2310036O22Rik)	1.62
Asah1	N-acylsphingosine amidohydrolase 1 (Asah1)	1.62
Asb3	ankyrin repeat and SOCS box-containing protein 3 (Asb3)	1.62
Bbc3	Bcl-2 binding component 3 (Bbc3)	1.62
Sdcbp	syndecan binding protein (Sdcbp), transcript variant 1	1.62
Spg20	spastic paraplegia 20, spartin (Troyer syndrome) homolog (human) (Spg20)	1.62
Thbd	thrombomodulin (Thbd)	1.62
Btg1	B-cell translocation gene 1, anti-proliferative (Btg1)	1.6
Kcnip1	Kv channel-interacting protein 1 (Kcnip1)	1.6
Morf4l1	mortality factor 4 like 1 (Morf4l1), transcript variant 2	1.6
Nt5c3	5'-nucleotidase, cytosolic III (Nt5c3)	1.6
OTTMUSG00000004461	predicted gene, OTTMUSG00000004461 (OTTMUSG00000004461)	1.6
Pkig	protein kinase inhibitor, gamma (Pkig), transcript variant 3	1.6
Wdr45l	Wdr45 like (Wdr45l)	1.6
Adam15	a disintegrin and metallopeptidase domain 15 (metargidin) (Adam15), transcript variant 2	1.59
Coil	coilin (Coil)	1.59
Cstb	cystatin B (Cstb)	1.59
4931406P16Rik	RIKEN cDNA 4931406P16 gene (4931406P16Rik)	1.58
Adam32	a disintegrin and metallopeptidase domain 32 (Adam32)	1.58
Arhgef6	Rac/Cdc42 guanine nucleotide exchange factor (GEF) 6 (Arhgef6)	1.58
BC004728	cDNA sequence BC004728 (BC004728), transcript variant 1	1.58
D230037D09Rik	RIKEN cDNA D230037D09 gene (D230037D09Rik)	1.58
Enpp4	ectonucleotide pyrophosphatase/phosphodiesterase 4 (Enpp4)	1.58
Fgf21	fibroblast growth factor 21 (Fgf21)	1.58
Grip1	glutamate receptor interacting protein 1 (Grip1), transcript variant 2	1.58
Med31	mediator of RNA polymerase II transcription, subunit 31 homolog (yeast) (Med31)	1.58
Prmt2	protein arginine N-methyltransferase 2 (Prmt2), transcript variant 2	1.58
Tgfbi	transforming growth factor, beta induced (Tgfbi)	1.58
1110020P15Rik	RIKEN cDNA 1110020P15 gene (1110020P15Rik)	1.57
Dpp4	dipeptidylpeptidase 4 (Dpp4)	1.57
Fem1c	fem-1 homolog c (C.elegans) (Fem1c)	1.57
Gpx4	glutathione peroxidase 4 (Gpx4), transcript variant 1	1.57

Jam4	junction adhesion molecule 4 (Jam4)	1.57
Mtus1	mitochondrial tumor suppressor 1 (Mtus1), nuclear gene encoding mitochondrial protein, transcript variant 4	1.57
Nhp2l1	NHP2 non-histone chromosome protein 2-like 1 (S. cerevisiae) (Nhp2l1)	1.57
Palld	palladin, cytoskeletal associated protein (Palld)	1.57
Atp5l	ATP synthase, H+ transporting, mitochondrial F0 complex, subunit g (Atp5l), nuclear gene encoding mitochondrial protein	1.56
Cyba	cytochrome b-245, alpha polypeptide (Cyba)	1.56
Endod1	endonuclease domain containing 1 (Endod1)	1.56
Fmo1	flavin containing monooxygenase 1 (Fmo1)	1.56
Notch4	Notch gene homolog 4 (Drosophila) (Notch4)	1.56
Ovol2	ovo-like 2 (Drosophila) (Ovol2), transcript variant B	1.56
Pmm1	phosphomannomutase 1 (Pmm1)	1.56
1110020P15Rik	RIKEN cDNA 1110020P15 gene (1110020P15Rik)	1.55
1700019D03Rik	RIKEN cDNA 1700019D03 gene (1700019D03Rik)	1.55
Apoc1	apolipoprotein C-I (Apoc1)	1.55
H2afz	H2A histone family, member Z (H2afz)	1.55
Litaf	LPS-induced TN factor (Litaf)	1.55
Nudt7	nudix (nucleoside diphosphate linked moiety X)-type motif 7 (Nudt7), transcript variant 1	1.55
Agpat4	1-acylglycerol-3-phosphate O-acyltransferase 4 (lysophosphatidic acid acyltransferase, delta) (Agpat4)	1.54
Cgrrf1	cell growth regulator with ring finger domain 1 (Cgrrf1)	1.54
Copg	coatomer protein complex, subunit gamma (Copg), transcript variant 2	1.54
Dab2	disabled homolog 2 (Drosophila) (Dab2), transcript variant 2	1.54
Gm1673	gene model 1673, (NCBI) (Gm1673)	1.54
LOC386486	LOC386486	1.54
Mnat1	menage a trois 1 (Mnat1)	1.54
Nr1h3	nuclear receptor subfamily 1, group H, member 3 (Nr1h3)	1.54
Phactr4	phosphatase and actin regulator 4 (Phactr4)	1.54
Phf13	PHD finger protein 13 (Phf13)	1.54
Rdm1	RAD52 motif 1 (Rdm1)	1.54
Sgpp2	sphingosine-1-phosphate phosphotase 2 (Sgpp2)	1.54
Tceb1	transcription elongation factor B (SIII), polypeptide 1 (Tceb1)	1.54
1110058L19Rik	RIKEN cDNA 1110058L19 gene (1110058L19Rik)	1.53
Dctn6	dynactin 6 (Dctn6)	1.53
Emp2	epithelial membrane protein 2 (Emp2)	1.53
Pftk1	PFTAIRE protein kinase 1 (Pftk1)	1.53
Snn	stannin (Snn)	1.53
Snx4	sorting nexin 4 (Snx4)	1.53
2310016M24Rik	RIKEN cDNA 2310016M24 gene (2310016M24Rik)	1.52
Arl6ip5	ADP-ribosylation factor-like 6 interacting protein 5 (Arl6ip5)	1.52
Atp1b3	ATPase, Na+/K+ transporting, beta 3 polypeptide (Atp1b3)	1.52
Mdk	midkine (Mdk), transcript variant 3	1.52
Nipsnap3a	nipsnap homolog 3A (C. elegans) (Nipsnap3a)	1.52
Thoc4	THO complex 4 (Thoc4)	1.52
Vps29	vacuolar protein sorting 29 (S. pombe) (Vps29)	1.52
2610208M17Rik	RIKEN cDNA 2610208M17 gene (2610208M17Rik)	1.51
Bola3	bolA-like 3 (E. coli) (Bola3)	1.51
Gtf2f2	general transcription factor IIF, polypeptide 2 (Gtf2f2)	1.51
Ucn3	urocortin 3 (Ucn3)	1.51

Fbxo16	F-box protein 16 (Fbxo16)	1.5
Fmnl3	formin-like 3 (Fmnl3)	1.5
Mrpl33	mitochondrial ribosomal protein L33 (Mrpl33), nuclear gene encoding mitochondrial protein	1.5
Slc7a8	solute carrier family 7 (cationic amino acid transporter, y+ system), member 8 (Slc7a8)	1.5
Lpin2	lipin 2 (Lpin2)	-1.5
Sgsm3	small G protein signaling modulator 3 (Sgsm3)	-1.5
Snapc4	small nuclear RNA activating complex, polypeptide 4 (Snapc4)	-1.5
Ctsc	cathepsin C (Ctsc)	-1.51
Gtf2h4	general transcription factor II H, polypeptide 4 (Gtf2h4)	-1.51
Irak3	interleukin-1 receptor-associated kinase 3 (Irak3)	-1.51
Mrps17	mitochondrial ribosomal protein S17 (Mrps17)	-1.51
Nek9	NIMA (never in mitosis gene a)-related expressed kinase 9 (Nek9)	-1.51
Npepl1	aminopeptidase-like 1 (Npepl1)	-1.51
Ring1	ring finger protein 1 (Ring1)	-1.51
Sh3rf1	SH3 domain containing ring finger 1 (Sh3rf1)	-1.51
4933426M11Rik	RIKEN cDNA 4933426M11 gene (4933426M11Rik)	-1.52
Atp9b	ATPase, class II, type 9B (Atp9b)	-1.52
Aven	apoptosis, caspase activation inhibitor (Aven)	-1.52
Cog3	component of oligomeric golgi complex 3 (Cog3)	-1.52
Ddx17	DEAD (Asp-Glu-Ala-Asp) box polypeptide 17 (Ddx17), transcript variant 1	-1.52
Emg1	EMG1 nucleolar protein homolog (S. cerevisiae) (Emg1)	-1.52
Herc2	hect (homologous to the E6-AP (UBE3A) carboxyl terminus) domain and RCC1 (CHC1)-like domain (RLD) 2 (Herc2)	-1.52
Hs6st1	heparan sulfate 6-O-sulfotransferase 1 (Hs6st1)	-1.52
Josd2	Josephin domain containing 2 (Josd2)	-1.52
Lztr1	leucine-zipper-like transcriptional regulator, 1 (Lztr1)	-1.52
Pcyt2	phosphate cytidylyltransferase 2, ethanolamine (Pcyt2)	-1.52
Pias4	protein inhibitor of activated STAT 4 (Pias4)	-1.52
Sf4	splicing factor 4 (Sf4)	-1.52
Tha1	threonine aldolase 1 (Tha1)	-1.52
Tyk2	tyrosine kinase 2 (Tyk2)	-1.52
Usp4	ubiquitin specific peptidase 4 (proto-oncogene) (Usp4)	-1.52
Zhx2	zinc fingers and homeoboxes 2 (Zhx2)	-1.52
2410002F23Rik	RIKEN cDNA 2410002F23 gene (2410002F23Rik)	-1.53
Cmtm8	CKLF-like MARVEL transmembrane domain containing 8 (Cmtm8)	-1.53
Gabarap	gamma-aminobutyric acid receptor associated protein (Gabarap)	-1.53
Irf9	interferon regulatory factor 9 (Irf9)	-1.53
Ogfrl1	opioid growth factor receptor-like 1 (Ogfrl1)	-1.53
Pdk2	pyruvate dehydrogenase kinase, isoenzyme 2 (Pdk2)	-1.53
Pomgnt1	protein O-linked mannose beta1,2-N-acetylglucosaminyltransferase (Pomgnt1), transcript variant 2	-1.53
Unc84b	unc-84 homolog B (C. elegans) (Unc84b)	-1.53
Wipi2	WD repeat domain, phosphoinositide interacting 2 (Wipi2)	-1.53
Yrdc	yrdC domain containing (E.coli) (Yrdc)	-1.53
Abcc5	ATP-binding cassette, sub-family C (CFTR/MRP), member 5 (Abcc5), transcript variant 2	-1.54
Aldh1l2	aldehyde dehydrogenase 1 family, member L2 (Aldh1l2)	-1.54
Alg3	asparagine-linked glycosylation 3 homolog (yeast, alpha-1,3-mannosyltransferase) (Alg3)	-1.54
C630004H02Rik	RIKEN cDNA C630004H02 gene (C630004H02Rik)	-1.54
Kif12	kinesin family member 12 (Kif12)	-1.54
Lrrn1	leucine rich repeat protein 1, neuronal (Lrrn1)	-1.54

Pgam5	phosphoglycerate mutase family member 5 (Pgam5)	-1.54
Rfx1	regulatory factor X, 1 (influences HLA class II expression) (Rfx1)	-1.54
Slc35e3	solute carrier family 35, member E3 (Slc35e3)	-1.54
Slc7a3	solute carrier family 7 (cationic amino acid transporter, y+ system), member 3 (Slc7a3)	-1.54
Tbrg1	transforming growth factor beta regulated gene 1 (Tbrg1)	-1.54
2210010C04Rik	RIKEN cDNA 2210010C04 gene (2210010C04Rik)	-1.55
2310044G17Rik	RIKEN cDNA 2310044G17 gene (2310044G17Rik)	-1.55
Ang	angiogenin, ribonuclease, RNase A family, 5 (Ang)	-1.55
Cad	carbamoyl-phosphate synthetase 2, aspartate transcarbamylase, and dihydroorotase (Cad)	-1.55
Capzb	capping protein (actin filament) muscle Z-line, beta (Capzb), transcript variant 1	-1.55
Daam1	dishevelled associated activator of morphogenesis 1 (Daam1), transcript variant 1	-1.55
Gpc4	glypican 4 (Gpc4)	-1.55
Rtn1	reticulon 1 (Rtn1), transcript variant 2	-1.55
Vps16	vacuolar protein sorting 16 (yeast) (Vps16)	-1.55
Zfp524	zinc finger protein 524 (Zfp524)	-1.55
0910001L09Rik	RIKEN cDNA 0910001L09 gene (0910001L09Rik)	-1.56
Bcl2l13	BCL2-like 13 (apoptosis facilitator) (Bcl2l13), nuclear gene encoding mitochondrial protein	-1.56
Bmpr1b	bone morphogenetic protein receptor, type 1B (Bmpr1b)	-1.56
Dhrsx	dehydrogenase/reductase (SDR family) X chromosome (Dhrsx)	-1.56
Ptrh1	peptidyl-tRNA hydrolase 1 homolog (S. cerevisiae) (Ptrh1)	-1.56
Tmem63a	transmembrane protein 63a (Tmem63a)	-1.56
Itgb4	integrin beta 4 (Itgb4), transcript variant 2	-1.57
Lcmt1	leucine carboxyl methyltransferase 1 (Lcmt1)	-1.57
LOC100045617	similar to Eukaryotic translation initiation factor 4A2 (LOC100045617)	-1.57
LOC100047009	hypothetical protein LOC100047009 (LOC100047009), misc RNA.	-1.57
Parp16	poly (ADP-ribose) polymerase family, member 16 (Parp16)	-1.57
Prmt7	protein arginine N-methyltransferase 7 (Prmt7)	-1.57
Ror2	receptor tyrosine kinase-like orphan receptor 2 (Ror2)	-1.57
Slc19a2	solute carrier family 19 (thiamine transporter), member 2 (Slc19a2)	-1.57
Ttyh1	tweety homolog 1 (Drosophila) (Ttyh1), transcript variant 2	-1.57
Ctps	cytidine 5'-triphosphate synthase (Ctps)	-1.58
LOC100047323	similar to ataxin 2 (LOC100047323), misc RNA.	-1.58
Nr2f6	nuclear receptor subfamily 2, group F, member 6 (Nr2f6)	-1.58
Psme3	proteaseome (prosome, macropain) 28 subunit, 3 (Psme3)	-1.58
Rae1	RAE1 RNA export 1 homolog (S. pombe) (Rae1)	-1.58
Slc27a1	solute carrier family 27 (fatty acid transporter), member 1 (Slc27a1)	-1.58
Sqrdl	sulfide quinone reductase-like (yeast) (Sqrdl)	-1.58
Zfp259	zinc finger protein 259 (Zfp259)	-1.58
4833439L19Rik	RIKEN cDNA 4833439L19 gene (4833439L19Rik)	-1.59
Card10	caspase recruitment domain family, member 10 (Card10)	-1.59
Foxj2	forkhead box J2 (Foxj2)	-1.59
Ndor1	NADPH dependent diflavin oxidoreductase 1 (Ndor1)	-1.59
Rhbdl2	rhomboid, veinlet-like 2 (Drosophila) (Rhbdl2)	-1.59
Setd6	SET domain containing 6 (Setd6)	-1.59
Sult4a1	sulfotransferase family 4A, member 1 (Sult4a1)	-1.59
Ube3b	ubiquitin protein ligase E3B (Ube3b)	-1.59
Zxdc	ZXD family zinc finger C (Zxdc), transcript variant 2	-1.59
5930434B04Rik	RIKEN cDNA 5930434B04 gene (5930434B04Rik)	-1.6

Arglu1	arginine and glutamate rich 1 (Arglu1)	-1.6
Dgkq	diacylglycerol kinase, theta (Dgkq)	-1.6
Furin	furin (paired basic amino acid cleaving enzyme) (Furin)	-1.6
Men1	multiple endocrine neoplasia 1 (Men1)	-1.6
Nme2	non-metastatic cells 2, protein (NM23B) expressed in (Nme2), transcript variant 1	-1.6
1110039B18Rik	RIKEN cDNA 1110039B18 gene (1110039B18Rik)	-1.61
Akt1s1	AKT1 substrate 1 (proline-rich) (Akt1s1)	-1.61
Gpt1	glutamic pyruvic transaminase 1, soluble (Gpt1)	-1.61
Pnliprp2	pancreatic lipase-related protein 2 (Pnliprp2)	-1.61
Pols	polymerase (DNA directed) sigma (Pols)	-1.61
Rpap2	RNA polymerase II associated protein 2 (Rpap2)	-1.61
Slc26a6	solute carrier family 26, member 6 (Slc26a6)	-1.61
Slc2a13	solute carrier family 2 (facilitated glucose transporter), member 13 (Slc2a13)	-1.61
Brp16	brain protein 16 (Brp16)	-1.62
Dars	aspartyl-tRNA synthetase (Dars)	-1.62
E4f1	E4F transcription factor 1 (E4f1)	-1.62
Es22	esterase 22 (Es22)	-1.62
Dus1l	dihydrouridine synthase 1-like (S. cerevisiae) (Dus1l)	-1.63
Edc4	enhancer of mRNA decapping 4 (Edc4)	-1.63
Ehmt2	euchromatic histone lysine N-methyltransferase 2 (Ehmt2), transcript variant short	-1.63
Eif4h	eukaryotic translation initiation factor 4H (Eif4h)	-1.63
Ptpla	protein tyrosine phosphatase-like (proline instead of catalytic arginine), member a (Ptpla), transcript variant 2	-1.63
Setd5	SET domain containing 5 (Setd5)	-1.63
Slc1a3	solute carrier family 1 (glial high affinity glutamate transporter), member 3 (Slc1a3)	-1.63
Spast	spastin (Spast)	-1.63
Unc84a	unc-84 homolog A (C. elegans) (Unc84a)	-1.63
D12Ertd647e	DNA segment, Chr 12, ERATO Doi 647, expressed (D12Ertd647e), transcript variant 4	-1.64
Dgcr2	DiGeorge syndrome critical region gene 2 (Dgcr2)	-1.64
Exosc6	exosome component 6 (Exosc6)	-1.64
Pebp1	phosphatidylethanolamine binding protein 1 (Pebp1)	-1.64
Pex11c	peroxisomal biogenesis factor 11c (Pex11c)	-1.64
Plrg1	pleiotropic regulator 1, PRL1 homolog (Arabidopsis) (Plrg1)	-1.64
Tmem68	transmembrane protein 68 (Tmem68)	-1.64
Zadh2	zinc binding alcohol dehydrogenase, domain containing 2 (Zadh2)	-1.64
Dido1	death inducer-obliterator 1 (Dido1), transcript variant 1	-1.65
Rab3gap1	RAB3 GTPase activating protein subunit 1 (Rab3gap1)	-1.65
Slc25a39	solute carrier family 25, member 39 (Slc25a39)	-1.65
5830457O10Rik	RIKEN cDNA 5830457O10 gene (5830457O10Rik)	-1.66
Entpd4	ectonucleoside triphosphate diphosphohydrolase 4 (Entpd4)	-1.66
Wbp7	WW domain binding protein 7 (Wbp7)	-1.66
Zdhhc12	zinc finger, DHHC domain containing 12 (Zdhhc12)	-1.66
Sept9	septin 9 (Sept9)	-1.67
Abhd4	abhydrolase domain containing 4 (Abhd4)	-1.67
Acad9	acyl-Coenzyme A dehydrogenase family, member 9 (Acad9)	-1.67
Cdc42se1	CDC42 small effector 1 (Cdc42se1), transcript variant 1	-1.67
Itgb4	integrin beta 4 (Itgb4), transcript variant 1	-1.67
Prkcd	protein kinase C, delta (Prkcd)	-1.67
Rnf25	ring finger protein 25 (Rnf25)	-1.67

Slc35c2	solute carrier family 35, member C2 (Slc35c2)	-1.67
Tars	threonyl-tRNA synthetase (Tars)	-1.67
Tnfrsf21	tumor necrosis factor receptor superfamily, member 21 (Tnfrsf21)	-1.67
AI450540	expressed sequence AI450540 (AI450540)	-1.68
Arfip2	ADP-ribosylation factor interacting protein 2 (Arfip2)	-1.68
Clk2	CDC-like kinase 2 (Clk2)	-1.68
Foxred1	FAD-dependent oxidoreductase domain containing 1 (Foxred1)	-1.68
St14	suppression of tumorigenicity 14 (colon carcinoma) (St14)	-1.68
Abcf3	ATP-binding cassette, sub-family F (GCN20), member 3 (Abcf3)	-1.69
Acsl6	acyl-CoA synthetase long-chain family member 6 (Acsl6), transcript variant 4	-1.69
Fbxl6	F-box and leucine-rich repeat protein 6 (Fbxl6)	-1.69
Mtap1s	microtubule-associated protein 1S (Mtap1s)	-1.69
Sel1l	sel-1 suppressor of lin-12-like (C. elegans) (Sel1l), transcript variant 1	-1.69
Aof1	amine oxidase, flavin containing 1 (Aof1)	-1.7
Brf1	BRF1 homolog, subunit of RNA polymerase III transcription initiation factor IIIB (S. cerevisiae) (Brf1)	-1.7
Cuzd1	CUB and zona pellucida-like domains 1 (Cuzd1)	-1.7
Gcs1	glucosidase 1 (Gcs1)	-1.7
Mapk13	mitogen-activated protein kinase 13 (Mapk13)	-1.7
Mrpl12	mitochondrial ribosomal protein L12 (Mrpl12), nuclear gene encoding mitochondrial protein	-1.7
Rnpep	arginyl aminopeptidase (aminopeptidase B) (Rnpep)	-1.7
Slc25a37	solute carrier family 25, member 37 (Slc25a37), nuclear gene encoding mitochondrial protein	-1.7
Tbl1x	transducin (beta)-like 1 X-linked (Tbl1x)	-1.7
Ufsp2	UFM1-specific peptidase 2 (Ufsp2)	-1.7
Abhd11	abhydrolase domain containing 11 (Abhd11)	-1.71
Cc2d1b	coiled-coil and C2 domain containing 1B (Cc2d1b)	-1.71
Cic	capicua homolog (Drosophila) (Cic)	-1.71
Gpr107	G protein-coupled receptor 107 (Gpr107)	-1.71
Mecr	mitochondrial trans-2-enoyl-CoA reductase (Mecr), nuclear gene encoding mitochondrial protein	-1.71
Psme2	proteasome (prosome, macropain) 28 subunit, beta (Psme2), transcript variant 1	-1.71
Rbm28	RNA binding motif protein 28 (Rbm28), transcript variant 2	-1.71
Sharpin	SHANK-associated RH domain interacting protein (Sharpin)	-1.71
Stxbp2	syntaxin binding protein 2 (Stxbp2)	-1.71
Cecr5	cat eye syndrome chromosome region, candidate 5 homolog (human) (Cecr5)	-1.72
Cpa2	carboxypeptidase A2, pancreatic (Cpa2)	-1.72
Herpud1	homocysteine-inducible, endoplasmic reticulum stress-inducible, ubiquitin-like domain member 1 (Herpud1)	-1.72
LOC100044122	similar to Potassium voltage-gated channel, subfamily Q, member 1 (LOC100044122)	-1.72
Upf1	UPF1 regulator of nonsense transcripts homolog (yeast) (Upf1)	-1.72
Cdc42ep1	CDC42 effector protein (Rho GTPase binding) 1 (Cdc42ep1)	-1.73
Git2	G protein-coupled receptor kinase-interactor 2 (Git2), transcript variant 1	-1.73
Ly6e	lymphocyte antigen 6 complex, locus E (Ly6e)	-1.73
Sema4a	sema domain, immunoglobulin domain (Ig), transmembrane domain (TM) and short cytoplasmic domain, (semaphorin) 4A (Sema4a)	-1.73
Snf8	SNF8, ESCRT-II complex subunit, homolog (S. cerevisiae) (Snf8)	-1.73
Ttc14	tetratricopeptide repeat domain 14 (Ttc14)	-1.73
AA409316	expressed sequence AA409316 (AA409316)	-1.74
Hsbp1	heat shock factor binding protein 1 (Hsbp1)	-1.74
Pkp3	plakophilin 3 (Pkp3)	-1.74
Sfrs7	splicing factor, arginine/serine-rich 7 (Sfrs7)	-1.74

BC085271	cDNA sequence BC085271 (BC085271)	-1.75
Cbs	cystathionine beta-synthase (Cbs), transcript variant 1	-1.75
Asb13	ankyrin repeat and SOCS box-containing protein 13 (Asb13)	-1.76
Gbp3	guanylate nucleotide binding protein 3 (Gbp3)	-1.76
H2-Ab1	histocompatibility 2, class II antigen A, beta 1 (H2-Ab1)	-1.76
Itpr2	inositol 1,4,5-triphosphate receptor 2 (Itpr2), transcript variant 1	-1.76
Ptpn1	protein tyrosine phosphatase, non-receptor type 1 (Ptpn1)	-1.76
Rrp12	ribosomal RNA processing 12 homolog (S. cerevisiae) (Rrp12)	-1.76
Ubqln4	ubiquilin 4 (Ubqln4)	-1.76
Zfp592	zinc finger protein 592 (Zfp592)	-1.76
Cd82	CD82 antigen (Cd82)	-1.77
Eef1g	eukaryotic translation elongation factor 1 gamma (Eef1g)	-1.77
Insrr	insulin receptor-related receptor (Insrr)	-1.77
Matn4	matrilin 4 (Matn4)	-1.77
Mlycd	malonyl-CoA decarboxylase (Mlycd), nuclear gene encoding mitochondrial protein	-1.77
5930434B04Rik	RIKEN cDNA 5930434B04 gene (5930434B04Rik)	-1.78
Adck4	aarF domain containing kinase 4 (Adck4)	-1.78
Atp9a	ATPase, class II, type 9A (Atp9a)	-1.78
Bmp3	bone morphogenetic protein 3 (Bmp3)	-1.78
Impdh2	inosine 5'-phosphate dehydrogenase 2 (Impdh2)	-1.78
2310061F22Rik	RIKEN cDNA 2310061F22 gene (2310061F22Rik)	-1.79
4833420G17Rik	RIKEN cDNA 4833420G17 gene (4833420G17Rik)	-1.79
4930504E06Rik	RIKEN cDNA 4930504E06 gene (4930504E06Rik)	-1.79
Cpne8	copine VIII (Cpne8), transcript variant 2	-1.79
Csnk2a2	casein kinase 2, alpha prime polypeptide (Csnk2a2)	-1.79
Eif2b1	eukaryotic translation initiation factor 2B, subunit 1 (alpha) (Eif2b1)	-1.79
AA881470	EST AA881470 (AA881470), transcript variant 1	-1.8
Thap4	THAP domain containing 4 (Thap4)	-1.8
Ttc13	tetratricopeptide repeat domain 13 (Ttc13)	-1.8
Zc3h13	zinc finger CCCH type containing 13 (Zc3h13)	-1.8
Anpep	alanyl (membrane) aminopeptidase (Anpep)	-1.81
Nubp1	nucleotide binding protein 1 (Nubp1)	-1.81
Whrn	whirlin (Whrn), transcript variant 3	-1.81
Ankle2	ankyrin repeat and LEM domain containing 2 (Ankle2)	-1.82
Paox	polyamine oxidase (exo-N4-amino) (Paox)	-1.82
Scrib	scribbled homolog (Drosophila) (Scrib)	-1.82
2810403A07Rik	RIKEN cDNA 2810403A07 gene (2810403A07Rik)	-1.83
Hectd3	HECT domain containing 3 (Hectd3)	-1.83
Nomo1	nodal modulator 1 (Nomo1)	-1.83
Thop1	thimet oligopeptidase 1 (Thop1)	-1.83
Tmem150	transmembrane protein 150 (Tmem150)	-1.83
Trim39	tripartite motif-containing 39 (Trim39)	-1.83
Trmt1	TRM1 tRNA methyltransferase 1 homolog (S. cerevisiae) (Trmt1)	-1.83
Akap1	A kinase (PRKA) anchor protein 1 (Akap1), nuclear gene encoding mitochondrial protein, transcript variant 2	-1.84
Arrb2	arrestin, beta 2 (Arrb2)	-1.84
Bahd1	bromo adjacent homology domain containing 1 (Bahd1)	-1.84
Chfr	checkpoint with forkhead and ring finger domains (Chfr)	-1.84
Gripap1	GRIP1 associated protein 1 (Gripap1)	-1.84

Htra2	HtrA serine peptidase 2 (Htra2), nuclear gene encoding mitochondrial protein	-1.84
Klf6	Kruppel-like factor 6 (Klf6)	-1.84
Lman2l	lectin, mannose-binding 2-like (Lman2l)	-1.84
Map3k7ip1	mitogen-activated protein kinase kinase kinase 7 interacting protein 1 (Map3k7ip1)	-1.84
Plac8	placenta-specific 8 (Plac8)	-1.84
Serinc3	serine incorporator 3 (Serinc3)	-1.84
0710008K08Rik	RIKEN cDNA 0710008K08 gene (0710008K08Rik)	-1.85
Bop1	block of proliferation 1 (Bop1)	-1.85
Dgat1	diacylglycerol O-acyltransferase 1 (Dgat1)	-1.85
Dvl1	dishevelled, dsh homolog 1 (Drosophila) (Dvl1)	-1.85
Map2k2	mitogen activated protein kinase kinase 2 (Map2k2)	-1.85
Pfdn2	prefoldin 2 (Pfdn2)	-1.85
Slc12a8	solute carrier family 12 (potassium/chloride transporters), member 8 (Slc12a8), transcript variant 2	-1.85
Tfdp2	transcription factor Dp 2 (Tfdp2)	-1.85
Tmem120b	transmembrane protein 120B (Tmem120b)	-1.85
Dus4l	dihydrouridine synthase 4-like (S. cerevisiae) (Dus4l)	-1.86
Erh	enhancer of rudimentary homolog (Drosophila) (Erh)	-1.86
Gvin1	GTPase, very large interferon inducible 1 (Gvin1), transcript variant B	-1.86
Nol6	nucleolar protein family 6 (RNA-associated) (Nol6)	-1.86
Ssh3	slingshot homolog 3 (Drosophila) (Ssh3)	-1.86
Tmem11	transmembrane protein 11 (Tmem11)	-1.86
BC005537	cDNA sequence BC005537 (BC005537)	-1.87
Gltp	glycolipid transfer protein (Gltp)	-1.87
Hspa1a	heat shock protein 1A (Hspa1a)	-1.87
Nab1	Ngfi-A binding protein 1 (Nab1)	-1.87
Pick1	protein interacting with C kinase 1 (Pick1), transcript variant 1	-1.87
Ppm1l	protein phosphatase 1 (formerly 2C)-like (Ppm1l)	-1.87
Tinag	tubulointerstitial nephritis antigen (Tinag)	-1.87
Ulk1	Unc-51 like kinase 1 (C. elegans) (Ulk1)	-1.87
Adcy9	adenylate cyclase 9 (Adcy9)	-1.88
Nup210	nucleoporin 210 (Nup210)	-1.88
Ppp1r13b	protein phosphatase 1, regulatory (inhibitor) subunit 13B (Ppp1r13b)	-1.88
Sez6l	seizure related 6 homolog like (Sez6l)	-1.88
Sytl4	synaptotagmin-like 4 (Sytl4)	-1.88
Tmem160	transmembrane protein 160 (Tmem160)	-1.88
Bcl9l	B cell CLL/lymphoma 9-like (Bcl9l)	-1.89
Bpnt1	bisphosphate 3'-nucleotidase 1 (Bpnt1)	-1.89
Cabp2	calcium binding protein 2 (Cabp2)	-1.89
Jmjd5	jumonji domain containing 5 (Jmjd5)	-1.89
Laptm5	lysosomal-associated protein transmembrane 5 (Laptm5)	-1.89
Arfgap1	ADP-ribosylation factor GTPase activating protein 1 (Arfgap1)	-1.9
Gtf3c2	general transcription factor IIIC, polypeptide 2, beta (Gtf3c2)	-1.9
Rcan2	regulator of calcineurin 2 (Rcan2), transcript variant 1	-1.9
Tmem90a	transmembrane protein 90a (Tmem90a)	-1.9
Aff1	AF4/FMR2 family, member 1 (Aff1), transcript variant 2	-1.91
Arl3	ADP-ribosylation factor-like 3 (Arl3)	-1.91
Chkb	choline kinase beta (Chkb)	-1.91
Cog8	component of oligomeric golgi complex 8 (Cog8)	-1.91

Ergic1	endoplasmic reticulum-golgi intermediate compartment (ERGIC) 1 (Ergic1)	-1.91
Frag1	FGF receptor activating protein 1 (Frag1)	-1.91
Gnmt	glycine N-methyltransferase (Gnmt)	-1.91
Rab24	RAB24, member RAS oncogene family (Rab24)	-1.91
Abcc10	ATP-binding cassette, sub-family C (CFTR/MRP), member 10 (Abcc10), transcript variant mrp7B	-1.92
Skiv2l	superkiller viralicidic activity 2-like (S. cerevisiae) (Skiv2l)	-1.92
Timm8a1	translocase of inner mitochondrial membrane 8 homolog a1 (yeast) (Timm8a1)	-1.92
Ikbkap	inhibitor of kappa light polypeptide enhancer in B-cells, kinase complex-associated protein (Ikbkap)	-1.93
Mpnd	MPN domain containing (Mpnd)	-1.93
Nod1	nucleotide-binding oligomerization domain containing 1 (Nod1)	-1.93
Smad1	MAD homolog 1 (Drosophila) (Smad1)	-1.93
Ttc7	tetratricopeptide repeat domain 7 (Ttc7)	-1.93
Vsig2	V-set and immunoglobulin domain containing 2 (Vsig2)	-1.93
1810022C23Rik	RIKEN cDNA 1810022C23 gene (1810022C23Rik)	-1.94
Ahctf1	AT hook containing transcription factor 1 (Ahctf1)	-1.94
Atp13a1	ATPase type 13A1 (Atp13a1)	-1.94
BC037034	cDNA sequence BC037034 (BC037034)	-1.94
Brsk2	BR serine/threonine kinase 2 (Brsk2), transcript variant 1	-1.94
Atg2a	ATG2 autophagy related 2 homolog A (S. cerevisiae) (Atg2a)	-1.95
Cpeb1	cytoplasmic polyadenylation element binding protein 1 (Cpeb1)	-1.95
Dap	death-associated protein (Dap)	-1.95
Ptprk	protein tyrosine phosphatase, receptor type, K (Ptprk)	-1.95
4632419K20Rik	RIKEN cDNA 4632419K20 gene (4632419K20Rik)	-1.96
Elovl6	ELOVL family member 6, elongation of long chain fatty acids (yeast) (Elovl6)	-1.96
Mib1	mindbomb homolog 1 (Drosophila) (Mib1)	-1.96
Timm44	translocase of inner mitochondrial membrane 44 (Timm44)	-1.96
Idua	iduronidase, alpha-L- (Idua)	-1.97
Lrrc16b	leucine rich repeat containing 16B (Lrrc16b)	-1.97
Mapkapk3	mitogen-activated protein kinase-activated protein kinase 3 (Mapkapk3)	-1.97
Mfn1	mitofusin 1 (Mfn1)	-1.97
Rgs17	regulator of G-protein signaling 17 (Rgs17)	-1.97
Rnf10	ring finger protein 10 (Rnf10)	-1.97
Smg7	Smg-7 homolog, nonsense mediated mRNA decay factor (C. elegans) (Smg7)	-1.97
Trafd1	TRAF type zinc finger domain containing 1 (Trafd1)	-1.97
Coq10b	coenzyme Q10 homolog B (S. cerevisiae) (Coq10b), transcript variant 1	-1.98
Csnk1g2	casein kinase 1, gamma 2 (Csnk1g2)	-1.98
Esd	esterase D/formylglutathione hydrolase (Esd)	-1.98
Lyar	Ly1 antibody reactive clone (Lyar)	-1.98
Plcd1	phospholipase C, delta 1 (Plcd1)	-1.98
Ppox	protoporphyrinogen oxidase (Ppox), nuclear gene encoding mitochondrial protein	-1.98
Rce1	RCE1 homolog, prenyl protein peptidase (S. cerevisiae) (Rce1)	-1.98
AA536749	expressed sequence AA536749 (AA536749)	-1.99
AI314180	expressed sequence AI314180 (AI314180)	-1.99
AU022252	expressed sequence AU022252 (AU022252)	-1.99
Ipo13	importin 13 (Ipo13)	-1.99
Mat2a	methionine adenosyltransferase II, alpha (Mat2a)	-1.99
Mccc1	methylcrotonoyl-Coenzyme A carboxylase 1 (alpha) (Mccc1)	-1.99
Psmb8	proteasome (prosome, macropain) subunit, beta type 8 (large multifunctional peptidase 7)	-1.99

	(Psmb8)	
Sypl	synaptophysin-like protein (Sypl), transcript variant 2	-1.99
Tsta3	tissue specific transplantation antigen P35B (Tsta3)	-1.99
A630042L21Rik	RIKEN cDNA A630042L21 gene (A630042L21Rik), transcript variant 2	-2
Adck5	aarF domain containing kinase 5 (Adck5)	-2
Exosc2	exosome component 2 (Exosc2)	-2
Naprt1	nicotinate phosphoribosyltransferase domain containing 1 (Naprt1)	-2
Rnpepl1	arginyl aminopeptidase (aminopeptidase B)-like 1 (Rnpepl1)	-2
Snapc3	small nuclear RNA activating complex, polypeptide 3 (Snapc3)	-2
2210412D01Rik	RIKEN cDNA 2210412D01 gene (2210412D01Rik)	-2.01
Zfp282	zinc finger protein 282 (Zfp282)	-2.01
1200015F23Rik	RIKEN cDNA 1200015F23 gene (1200015F23Rik)	-2.02
2410015N17Rik	RIKEN cDNA 2410015N17 gene (2410015N17Rik)	-2.02
D4Wsu114e	DNA segment, Chr 4, Wayne State University 114, expressed (D4Wsu114e)	-2.02
Slc35c2	solute carrier family 35, member C2 (Slc35c2)	-2.02
Ubp1	upstream binding protein 1 (Ubp1)	-2.02
Arl2bp	ADP-ribosylation factor-like 2 binding protein (Arl2bp), transcript variant 1	-2.03
Dgcr6	DiGeorge syndrome critical region gene 6 (Dgcr6)	-2.03
Eif2b4	eukaryotic translation initiation factor 2B, subunit 4 delta (Eif2b4)	-2.03
H2-DMb1	histocompatibility 2, class II, locus Mb1 (H2-DMb1)	-2.03
Pgs1	phosphatidylglycerophosphate synthase 1 (Pgs1)	-2.03
Setd1b	SET domain containing 1B (Setd1b)	-2.03
Stk25	serine/threonine kinase 25 (yeast) (Stk25)	-2.03
Glb1	galactosidase, beta 1 (Glb1)	-2.04
Tmem120a	transmembrane protein 120A (Tmem120a)	-2.04
Gna13	guanine nucleotide binding protein, alpha 13 (Gna13)	-2.05
Mid1ip1	Mid1 interacting protein 1 (gastrulation specific G12-like (zebrafish)) (Mid1ip1)	-2.05
Slc25a10	solute carrier family 25 (mitochondrial carrier, dicarboxylate transporter), member 10 (Slc25a10), nuclear gene encoding mitochondrial protein	-2.05
Zfp692	zinc finger protein 692 (Zfp692), transcript variant 1	-2.05
1700027J05Rik	RIKEN cDNA 1700027J05 gene (1700027J05Rik)	-2.06
Camkk2	calcium/calmodulin-dependent protein kinase kinase 2, beta (Camkk2)	-2.06
Pkp2	plakophilin 2 (Pkp2)	-2.06
Ptger3	prostaglandin E receptor 3 (subtype EP3) (Ptger3)	-2.06
Tle1	transducin-like enhancer of split 1, homolog of Drosophila E(spl) (Tle1)	-2.06
Ywhag	tyrosine 3-monooxygenase/tryptophan 5-monooxygenase activation protein, gamma polypeptide (Ywhag)	-2.06
1810044A24Rik	RIKEN cDNA 1810044A24 gene (1810044A24Rik), transcript variant 1	-2.07
Acin1	apoptotic chromatin condensation inducer 1 (Acin1), transcript variant 1	-2.07
Loh11cr2a	loss of heterozygosity, 11, chromosomal region 2, gene A homolog (human) (Loh11cr2a)	-2.07
Rnf31	ring finger protein 31 (Rnf31)	-2.07
Keap1	kelch-like ECH-associated protein 1 (Keap1)	-2.08
Pitpnm2	phosphatidylinositol transfer protein, membrane-associated 2 (Pitpnm2)	-2.08
Polrmt	polymerase (RNA) mitochondrial (DNA directed) (Polrmt), nuclear gene encoding mitochondrial protein	-2.08
6030443O07Rik	RIKEN cDNA 6030443O07 gene (6030443O07Rik)	-2.09
Gas2l1	growth arrest-specific 2 like 1 (Gas2l1), transcript variant beta	-2.09
Kcnk6	potassium inwardly-rectifying channel, subfamily K, member 6 (Kcnk6)	-2.09
Nola1	nucleolar protein family A, member 1 (H/ACA small nucleolar RNPs) (Nola1)	-2.09
Fbxw7	F-box and WD-40 domain protein 7, archipelago homolog (Drosophila) (Fbxw7)	-2.1

Prep	prolyl endopeptidase (Prep)	-2.1
Slc40a1	solute carrier family 40 (iron-regulated transporter), member 1 (Slc40a1)	-2.11
4833439L19Rik	RIKEN cDNA 4833439L19 gene (4833439L19Rik)	-2.12
Dalrd3	DALR anticodon binding domain containing 3 (Dalrd3)	-2.12
Mars	methionine-tRNA synthetase (Mars)	-2.13
Tmem125	transmembrane protein 125 (Tmem125)	-2.13
Abcb6	ATP-binding cassette, sub-family B (MDR/TAP), member 6 (Abcb6), nuclear gene encoding mitochondrial protein	-2.14
LOC100047810	similar to transmembrane emp24 protein transport domain containing 6 (LOC100047810)	-2.14
Nelf	nasal embryonic LHRH factor (Nelf), transcript variant 2	-2.14
Pex6	peroxisomal biogenesis factor 6 (Pex6)	-2.14
BC003266	cDNA sequence BC003266 (BC003266)	-2.15
Eif2s3x	eukaryotic translation initiation factor 2, subunit 3, structural gene X-linked (Eif2s3x)	-2.15
Pafah1b1	platelet-activating factor acetylhydrolase, isoform 1b, beta1 subunit (Pafah1b1)	-2.15
Bag3	Bcl2-associated athanogene 3 (Bag3)	-2.16
Bhlhb8	basic helix-loop-helix domain containing, class B, 8 (Bhlhb8)	-2.16
Grhpr	glyoxylate reductase/hydroxypyruvate reductase (Grhpr)	-2.16
Lcmt1	leucine carboxyl methyltransferase 1 (Lcmt1)	-2.16
Nuak2	NUAK family, SNF1-like kinase, 2 (Nuak2)	-2.16
Ube1l	ubiquitin-activating enzyme E1-like (Ube1l)	-2.16
2510010F15Rik	RIKEN 2510010F15Rik cDNA (2510010F15Rik)	-2.17
Clic6	chloride intracellular channel 6 (Clic6)	-2.17
Efcab4a	EF-hand calcium binding domain 4A (Efcab4a)	-2.17
Mon1a	MON1 homolog A (yeast) (Mon1a)	-2.17
Wdr5	WD repeat domain 5 (Wdr5)	-2.17
D19Wsu162e	DNA segment, Chr 19, Wayne State University 162, expressed (D19Wsu162e)	-2.18
Extl3	exostoses (multiple)-like 3 (Extl3)	-2.18
Gabarapl1	gamma-aminobutyric acid (GABA(A)) receptor-associated protein-like 1 (Gabarapl1)	-2.18
Rab6	RAB6, member RAS oncogene family (Rab6)	-2.18
1810033M07Rik	RIKEN cDNA 1810033M07 gene (1810033M07Rik)	-2.19
Ldb1	LIM domain binding 1 (Ldb1), transcript variant 3	-2.19
Slc25a28	solute carrier family 25, member 28 (Slc25a28)	-2.19
Cdv3	carnitine deficiency-associated gene expressed in ventricle 3 (Cdv3), transcript variant CDV3A	-2.2
LOC100044862	similar to Fbxl3 protein (LOC100044862)	-2.2
LOC546015	similar to ribosomal protein S9 (LOC546015), misc RNA.	-2.2
Pctk1	PCTAIRE-motif protein kinase 1 (Pctk1)	-2.2
Pdyn	prodynorphin (Pdyn)	-2.2
Cml4	camello-like 4 (Cml4)	-2.21
Esrra	estrogen related receptor, alpha (Esrra)	-2.21
Gorasp2	golgi reassembly stacking protein 2 (Gorasp2)	-2.21
Plekhg3	pleckstrin homology domain containing, family G (with RhoGef domain) member 3 (Plekhg3)	-2.21
Sfrs1	splicing factor, arginine/serine-rich 1 (ASF/SF2) (Sfrs1), transcript variant 1	-2.21
Snd1	staphylococcal nuclease and tudor domain containing 1 (Snd1)	-2.21
Tmub1	transmembrane and ubiquitin-like domain containing 1 (Tmub1)	-2.21
Hnrpdl	heterogeneous nuclear ribonucleoprotein D-like (Hnrpdl)	-2.22
Hps1	Hermansky-Pudlak syndrome 1 homolog (human) (Hps1)	-2.22
Macrod1	MACRO domain containing 1 (Macrod1)	-2.22
Nrbp1	nuclear receptor binding protein 1 (Nrbp1)	-2.22
Heatr1	HEAT repeat containing 1 (Heatr1)	-2.23

Mcfd2	multiple coagulation factor deficiency 2 (Mcfd2)	-2.23
Metap2	methionine aminopeptidase 2 (Metap2)	-2.23
Zfp579	zinc finger protein 579 (Zfp579)	-2.23
Gnb2l1	guanine nucleotide binding protein (G protein), beta polypeptide 2 like 1 (Gnb2l1)	-2.24
Ifit3	interferon-induced protein with tetratricopeptide repeats 3 (Ifit3)	-2.24
Mettl3	methyltransferase-like 3 (Mettl3)	-2.24
Mist1	basic helix-loop-helix family, member a15 (Mist1)	-2.24
2810046L04Rik	RIKEN cDNA 2810046L04 gene (2810046L04Rik)	-2.25
Arf5	ADP-ribosylation factor 5 (Arf5)	-2.25
Dnmt3b	DNA methyltransferase 3B (Dnmt3b), transcript variant 4	-2.25
Hgfac	hepatocyte growth factor activator (Hgfac)	-2.25
Rcor1	REST corepressor 1 (Rcor1)	-2.25
1110049F12Rik	RIKEN cDNA 1110049F12 gene (1110049F12Rik)	-2.26
2310061F22Rik	RIKEN cDNA 2310061F22 gene (2310061F22Rik)	-2.26
Cmtm4	CKLF-like MARVEL transmembrane domain containing 4 (Cmtm4)	-2.26
Mapkapk2	MAP kinase-activated protein kinase 2 (Mapkapk2)	-2.26
Als2	amyotrophic lateral sclerosis 2 (juvenile) homolog (human) (Als2)	-2.27
Atp13a2	ATPase type 13A2 (Atp13a2)	-2.27
Srm	spermidine synthase (Srm)	-2.27
Aup1	ancient ubiquitous protein (Aup1)	-2.28
Cd3e	CD3 antigen, epsilon polypeptide (Cd3e)	-2.28
Sec16b	SEC16 homolog B (S. cerevisiae) (Sec16b)	-2.28
Fkbp2	FK506 binding protein 2 (Fkbp2)	-2.29
Ipo4	importin 4 (Ipo4)	-2.29
LOC100042777	similar to human protein homologous to DROER protein (LOC100042777)	-2.29
LOC100048622	similar to EF-hand Ca2+ binding protein p22 (LOC100048622)	-2.29
Serpinb1a	serine (or cysteine) peptidase inhibitor, clade B, member 1a (Serpinb1a)	-2.29
2610002J02Rik	RIKEN cDNA 2610002J02 gene (2610002J02Rik)	-2.31
Aldh18a1	aldehyde dehydrogenase 18 family, member A1 (Aldh18a1), nuclear gene encoding mitochondrial protein, transcript variant 1	-2.32
Ddx20	DEAD (Asp-Glu-Ala-Asp) box polypeptide 20 (Ddx20)	-2.32
Med23	mediator complex subunit 23 (Med23)	-2.32
Snai3	snail homolog 3 (Drosophila) (Snai3)	-2.32
Cdv3	carnitine deficiency-associated gene expressed in ventricle 3 (Cdv3), transcript variant CDV3B	-2.33
Clec2d	C-type lectin domain family 2, member d (Clec2d)	-2.33
Sdccag3	serologically defined colon cancer antigen 3 (Sdccag3), transcript variant 4	-2.33
Exosc5	exosome component 5 (Exosc5)	-2.34
Stt3b	STT3, subunit of the oligosaccharyltransferase complex, homolog B (S. cerevisiae) (Stt3b)	-2.34
Rcl1	RNA terminal phosphate cyclase-like 1 (Rcl1)	-2.35
Snx8	sorting nexin 8 (Snx8)	-2.35
Eef1b2	eukaryotic translation elongation factor 1 beta 2 (Eef1b2)	-2.36
Ldhd	lactate dehydrogenase D (Ldhd), nuclear gene encoding mitochondrial protein	-2.36
Galk1	galactokinase 1 (Galk1)	-2.37
LOC100047937	Aldehyde dehydrogenase 1 family, member L1 (LOC100047937)	-2.37
Neurl	neuralized homolog (Drosophila) (Neurl)	-2.37
Prkd1	protein kinase D1 (Prkd1)	-2.37
Rrbp1	ribosome binding protein 1 (Rrbp1), transcript variant 1	-2.37
Srrm2	serine/arginine repetitive matrix 2 (Srrm2)	-2.37
9130404D08Rik	RIKEN cDNA 9130404D08 gene (9130404D08Rik)	-2.38

Osbpl6	oxysterol binding protein-like 6 (Osbpl6)	-2.38
Rnf219	ring finger protein 219 (Rnf219)	-2.38
Rpl7l1	ribosomal protein L7-like 1 (Rpl7l1)	-2.38
D10Wsu52e	DNA segment, Chr 10, Wayne State University 52, expressed (D10Wsu52e)	-2.4
Cdkn2b	cyclin-dependent kinase inhibitor 2B (p15, inhibits CDK4) (Cdkn2b)	-2.41
Lars2	leucyl-tRNA synthetase, mitochondrial (Lars2), nuclear gene encoding mitochondrial protein	-2.41
Parp14	poly (ADP-ribose) polymerase family, member 14 (Parp14)	-2.41
Trabd	TraB domain containing (Trabd)	-2.41
1700021K19Rik	RIKEN cDNA 1700021K19 gene (1700021K19Rik)	-2.42
Gspt1	G1 to S phase transition 1 (Gspt1)	-2.42
Rxrb	retinoid X receptor beta (Rxrb)	-2.42
BC017643	cDNA sequence BC017643 (BC017643)	-2.43
Qtrt1	queuine tRNA-ribosyltransferase 1 (Qtrt1)	-2.44
Smarcd2	SWI/SNF related, matrix associated, actin dependent regulator of chromatin, subfamily d, member 2 (Smarcd2)	-2.44
Acsl1	acyl-CoA synthetase long-chain family member 1 (Acsl1)	-2.45
Arhgef18	rho/rac guanine nucleotide exchange factor (GEF) 18 (Arhgef18)	-2.45
Lbh	limb-bud and heart (Lbh)	-2.45
Slc39a7	solute carrier family 39 (zinc transporter), member 7 (Slc39a7)	-2.45
4933439C20Rik	RIKEN cDNA 4933439C20 gene (4933439C20Rik)	-2.46
Fcer1g	Fc receptor, IgE, high affinity I, gamma polypeptide (Fcerg1)	-2.46
Cugbp1	CUG triplet repeat, RNA binding protein 1 (Cugbp1), transcript variant 2	-2.47
Ivd	isovaleryl coenzyme A dehydrogenase (Ivd), nuclear gene encoding mitochondrial protein	-2.47
Slc16a6	solute carrier family 16 (monocarboxylic acid transporters), member 6 (Slc16a6), transcript variant 2	-2.47
Snrpd3	small nuclear ribonucleoprotein D3 (Snrpd3)	-2.47
Rhbdf1	rhomboid family 1 (Drosophila) (Rhbdf1)	-2.48
Blmh	bleomycin hydrolase (Blmh)	-2.49
Cugbp1	CUG triplet repeat, RNA binding protein 1 (Cugbp1), transcript variant 2	-2.49
Klk1b4	kallikrein 1-related pepidase b4 (Klk1b4)	-2.49
Pkn1	protein kinase N1 (Pkn1)	-2.49
Aldh1l1	aldehyde dehydrogenase 1 family, member L1 (Aldh1l1)	-2.5
Psmb10	proteasome (prosome, macropain) subunit, beta type 10 (Psmb10)	-2.5
Zfp39	zinc finger protein 39 (Zfp39)	-2.5
Atp5e	ATP synthase, H+ transporting, mitochondrial F1 complex, epsilon subunit (Atp5e), nuclear gene encoding mitochondrial protein	-2.51
Prpf19	PRP19/PSO4 pre-mRNA processing factor 19 homolog (S. cerevisiae) (Prpf19)	-2.52
Ubqln1	ubiquilin 1 (Ubqln1), transcript variant 1	-2.52
Usp52	ubiquitin specific peptidase 52 (Usp52)	-2.52
Prss8	protease, serine, 8 (prostasin) (Prss8)	-2.53
Kctd5	potassium channel tetramerisation domain containing 5 (Kctd5)	-2.54
Mettl1	methyltransferase-like 1 (Mettl1)	-2.54
Por	P450 (cytochrome) oxidoreductase (Por)	-2.54
Grwd1	glutamate-rich WD repeat containing 1 (Grwd1)	-2.55
Adpgk	ADP-dependent glucokinase (Adpgk)	-2.57
LOC100046898	similar to Cell division cycle 34 homolog (S. cerevisiae) (LOC100046898)	-2.59
Extl3	exostoses (multiple)-like 3 (Extl3)	-2.6
Gorasp2	golgi reassembly stacking protein 2 (Gorasp2)	-2.6
Clmn	calmin (Clmn), transcript variant 2	-2.61
Gls2	glutaminase 2 (liver, mitochondrial) (Gls2)	-2.61

Hbs1l	Hbs1-like (S. cerevisiae) (Hbs1l), transcript variant 1	-2.61
2610507B11Rik	RIKEN cDNA 2610507B11 gene (2610507B11Rik)	-2.62
Ivns1abp	influenza virus NS1A binding protein (Ivns1abp), transcript variant 2	-2.62
Klc4	kinesin light chain 4 (Klc4)	-2.62
2310045A20Rik	RIKEN cDNA 2310045A20 gene (2310045A20Rik)	-2.63
Lrg1	leucine-rich alpha-2-glycoprotein 1 (Lrg1)	-2.63
Stard10	START domain containing 10 (Stard10)	-2.63
Fez1	fasciculation and elongation protein zeta 1 (zygin I) (Fez1)	-2.64
Gstp1	glutathione S-transferase, pi 1 (Gstp1)	-2.64
Rsad1	radical S-adenosyl methionine domain containing 1 (Rsad1)	-2.64
Spnb3	spectrin beta 3 (Spnb3)	-2.64
Clcn7	chloride channel 7 (Clcn7)	-2.68
Irf3	interferon regulatory factor 3 (Irf3)	-2.68
Pa2g4	proliferation-associated 2G4 (Pa2g4)	-2.68
Ccdc131	coiled-coil domain containing 131 (Ccdc131)	-2.69
Fcgr4	Fc receptor, IgG, low affinity IV (Fcgr4)	-2.69
Gls2	glutaminase 2 (liver, mitochondrial) (Gls2), nuclear gene encoding mitochondrial protein	-2.69
Prss3	protease, serine, 3 (Prss3)	-2.69
Bcl2l12	BCL2-like 12 (proline rich) (Bcl2l12)	-2.7
P2ry1	purinergic receptor P2Y, G-protein coupled 1 (P2ry1)	-2.71
Zfp771	zinc finger protein 771 (Zfp771)	-2.72
Asns	asparagine synthetase (Asns)	-2.73
LOC216443	LOC216443	-2.74
Bckdha	branched chain ketoacid dehydrogenase E1, alpha polypeptide (Bckdha)	-2.75
Gnb2	guanine nucleotide binding protein (G protein), beta 2 (Gnb2)	-2.75
4121402D02Rik	RIKEN cDNA 4121402D02 gene (4121402D02Rik)	-2.76
Atad3a	ATPase family, AAA domain containing 3A (Atad3a)	-2.76
Lyrm5	LYR motif containing 5 (Lyrm5)	-2.76
Capn2	calpain 2 (Capn2)	-2.77
Eif4el3	eukaryotic translation initiation factor 4E member 2 (Eif4el3)	-2.77
Insig2	insulin induced gene 2 (Insig2)	-2.77
Cd320	CD320 antigen (Cd320)	-2.78
Gad1	glutamic acid decarboxylase 1 (Gad1)	-2.78
Ubtf	upstream binding transcription factor, RNA polymerase I (Ubtf), transcript variant 2	-2.79
Gpt2	glutamic pyruvate transaminase (alanine aminotransferase) 2 (Gpt2)	-2.8
Rrbp1	ribosome binding protein 1 (Rrbp1), transcript variant 2	-2.8
Eif4a1	eukaryotic translation initiation factor 4A1 (Eif4a1)	-2.82
Slc6a9	solute carrier family 6 (neurotransmitter transporter, glycine), member 9 (Slc6a9)	-2.82
Bach2	BTB and CNC homology 2 (Bach2)	-2.83
Gpsm1	G-protein signalling modulator 1 (AGS3-like, C. elegans) (Gpsm1)	-2.83
Gtpbp2	GTP binding protein 2 (Gtpbp2)	-2.83
Rsad2	radical S-adenosyl methionine domain containing 2 (Rsad2)	-2.83
Echdc2	enoyl Coenzyme A hydratase domain containing 2 (Echdc2)	-2.84
Stat1	signal transducer and activator of transcription 1 (Stat1)	-2.84
Tle6	transducin-like enhancer of split 6, homolog of Drosophila E(spl) (Tle6)	-2.84
Gad1	glutamate decarboxylase 1 (Gad1)	-2.85
Stat3	signal transducer and activator of transcription 3 (Stat3), transcript variant 3	-2.86
Bckdhb	branched chain ketoacid dehydrogenase E1, beta polypeptide (Bckdhb), nuclear gene encoding mitochondrial protein	-2.87

LOC100043986	hypothetical protein LOC100043986 (LOC100043986), misc RNA.	-2.88
Nrbp2	nuclear receptor binding protein 2 (Nrbp2)	-2.89
Aqp12	aquaporin 12 (Aqp12)	-2.9
6330403K07Rik	RIKEN cDNA 6330403K07 gene (6330403K07Rik)	-2.91
BC027231	cDNA sequence BC027231 (BC027231)	-2.91
Cxxc1	CXXC finger 1 (PHD domain) (Cxxc1)	-2.91
Arhgap26	Rho GTPase activating protein 26 (Arhgap26)	-2.92
Hnf4a	hepatic nuclear factor 4, alpha (Hnf4a)	-2.92
Ddx54	DEAD (Asp-Glu-Ala-Asp) box polypeptide 54 (Ddx54)	-2.95
Adcy6	adenylate cyclase 6 (Adcy6)	-2.97
Arf6	ADP-ribosylation factor 6 (Arf6)	-2.97
Snx10	sorting nexin 10 (Snx10)	-2.97
Parp2	poly (ADP-ribose) polymerase family, member 2 (Parp2)	-2.98
Alg9	asparagine-linked glycosylation 9 homolog (yeast, alpha 1,2 mannosyltransferase) (Alg9)	-2.99
Mrps7	mitchondrial ribosomal protein S7 (Mrps7)	-2.99
Rps3a	ribosomal protein S3a (Rps3a)	-3
Vars	valyl-tRNA synthetase (Vars)	-3
Per1	period homolog 1 (Drosophila) (Per1)	-3.01
Eif4ebp1	eukaryotic translation initiation factor 4E binding protein 1 (Eif4ebp1)	-3.02
Mknk1	MAP kinase-interacting serine/threonine kinase 1 (Mknk1)	-3.02
Tmem184a	transmembrane protein 184a (Tmem184a)	-3.02
Ogt	O-linked N-acetylglucosamine (GlcNAc) transferase (UDP-N-acetylglucosamine:polypeptide-N-acetylglucosaminyl transferase) (Ogt)	-3.03
Sidt2	SID1 transmembrane family, member 2 (Sidt2)	-3.03
6720458F09Rik	RIKEN cDNA 6720458F09 gene (6720458F09Rik)	-3.04
2610207I05Rik	RIKEN cDNA 2610207I05 gene (2610207I05Rik)	-3.05
Klk1b5	kallikrein 1-related peptidase b5 (Klk1b5)	-3.06
Mthfd2	methylenetetrahydrofolate dehydrogenase (NAD+ dependent), methenyltetrahydrofolate cyclohydrolase (Mthfd2)	-3.07
Trim11	tripartite motif-containing 11 (Trim11)	-3.08
AI646023	expressed sequence AI646023 (AI646023)	-3.11
Iars	isoleucine-tRNA synthetase (Iars)	-3.11
Psat1	phosphoserine aminotransferase 1 (Psat1)	-3.11
Tapbpl	TAP binding protein-like (Tapbpl)	-3.11
Bmp7	bone morphogenetic protein 7 (Bmp7)	-3.12
Nudt6	nudix (nucleoside diphosphate linked moiety X)-type motif 6 (Nudt6)	-3.12
Sbf1	SET binding factor 1 (Sbf1)	-3.12
Slc38a3	solute carrier family 38, member 3 (Slc38a3)	-3.12
Inppl1	inositol polyphosphate phosphatase-like 1 (Inppl1)	-3.14
Atpbd1b	ATP binding domain 1 family, member B (Atpbd1b)	-3.15
Csf2ra	colony stimulating factor 2 receptor, alpha, low-affinity (granulocyte-macrophage) (Csf2ra)	-3.15
Suv420h2	suppressor of variegation 4-20 homolog 2 (Drosophila) (Suv420h2)	-3.15
Ece2	endothelin converting enzyme 2 (Ece2), transcript variant 3	-3.16
Rims3	regulating synaptic membrane exocytosis 3 (Rims3)	-3.16
Shmt2	serine hydroxymethyltransferase 2 (mitochondrial) (Shmt2), nuclear gene encoding mitochondrial protein	-3.16
B3galnt1	UDP-GalNAc:betaGlcNAc beta 1,3-galactosaminyltransferase, polypeptide 1 (B3galnt1)	-3.18
Ei24	etoposide induced 2.4 mRNA (Ei24)	-3.18
Wars	tryptophanyl-tRNA synthetase (Wars)	-3.19
Tst	thiosulfate sulfurtransferase, mitochondrial (Tst), nuclear gene encoding mitochondrial protein	-3.2

Frag1	FGF receptor activating protein 1 (Frag1)	-3.22
Tcea3	transcription elongation factor A (SII), 3 (Tcea3)	-3.22
Cdc25a	cell division cycle 25 homolog A (S. pombe) (Cdc25a)	-3.23
H2-M3	histocompatibility 2, M region locus 3 (H2-M3)	-3.23
Slc38a5	solute carrier family 38, member 5 (Slc38a5)	-3.24
2610204K14Rik	RIKEN cDNA 2610204K14 gene (2610204K14Rik)	-3.26
Chst2	carbohydrate sulfotransferase 2 (Chst2)	-3.26
Adk	adenosine kinase (Adk)	-3.27
Eif2b5	eukaryotic translation initiation factor 2B, subunit 5 epsilon (Eif2b5)	-3.27
Prss8	protease, serine, 8 (prostasin) (Prss8)	-3.28
Amy1	amylase 1, salivary (Amy1)	-3.29
Dbt	dihydrolipoamide branched chain transacylase E2 (Dbt)	-3.3
Tnfrsf12a	tumor necrosis factor receptor superfamily, member 12a (Tnfrsf12a)	-3.3
Atf5	activating transcription factor 5 (Atf5)	-3.32
B4galt1	UDP-Gal:betaGlcNAc beta 1,4- galactosyltransferase, polypeptide 1 (B4galt1)	-3.33
Afg3l1	AFG3(ATPase family gene 3)-like 1 (yeast) (Afg3l1)	-3.34
Wipi1	WD repeat domain, phosphoinositide interacting 1 (Wipi1)	-3.34
Klk1b8	kallikrein 1-related peptidase b8 (Klk1b8)	-3.36
Tcf25	transcription factor 25 (basic helix-loop-helix) (Tcf25), transcript variant 3	-3.37
Reg3a	regenerating islet-derived 3 alpha (Reg3a)	-3.38
Muc1	mucin 1, transmembrane (Muc1)	-3.39
AI428936	expressed sequence AI428936 (AI428936)	-3.4
Apobec1	apolipoprotein B editing complex 1 (Apobec1)	-3.4
Dusp7	dual specificity phosphatase 7 (Dusp7)	-3.45
Wnk2	WNK lysine deficient protein kinase 2 (Wnk2)	-3.45
Bzrap1	benzodiazapine receptor associated protein 1 (Bzrap1)	-3.46
Serinc2	serine incorporator 2 (Serinc2)	-3.46
Trim56	tripartite motif-containing 56 (Trim56)	-3.46
Iqgap2	IQ motif containing GTPase activating protein 2 (Iqgap2)	-3.47
Rbpjl	recombination signal binding protein for immunoglobulin kappa J region-like (Rbpjl)	-3.47
Cul7	cullin 7 (Cul7)	-3.49
Afg3l1	AFG3(ATPase family gene 3)-like 1 (yeast) (Afg3l1)	-3.5
Oasl2	2'-5' oligoadenylate synthetase-like 2 (Oasl2)	-3.5
Tap2	transporter 2, ATP-binding cassette, sub-family B (MDR/TAP) (Tap2)	-3.5
Rbm13	RNA binding motif protein 13 (Rbm13)	-3.54
Ahcy	S-adenosylhomocysteine hydrolase (Ahcy)	-3.56
P2ry14	purinergic receptor P2Y, G-protein coupled, 14 (P2ry14), transcript variant 1	-3.56
B3galt6	UDP-Gal:betaGal beta 1,3-galactosyltransferase, polypeptide 6 (B3galt6)	-3.57
H2-T23	histocompatibility 2, T region locus 23 (H2-T23)	-3.58
Iigp2	interferon inducible GTPase 2 (Iigp2)	-3.6
Pus1	pseudouridine synthase 1 (Pus1), transcript variant 1	-3.6
Slc39a5	solute carrier family 39 (metal ion transporter), member 5 (Slc39a5)	-3.6
Ak2	adenylate kinase 2 (Ak2), transcript variant 2	-3.62
Sema7a	sema domain, immunoglobulin domain (Ig), and GPI membrane anchor, (semaphorin) 7A (Sema7a)	-3.62
Slc39a5	solute carrier family 39 (metal ion transporter), member 5 (Slc39a5)	-3.62
Tmem63b	transmembrane protein 63b (Tmem63b)	-3.62
Camk2b	calcium/calmodulin-dependent protein kinase II, beta (Camk2b)	-3.64
Tsc2	tuberous sclerosis 2 (Tsc2), transcript variant 2	-3.66

Eif2b2	eukaryotic translation initiation factor 2B, subunit 2 beta (Eif2b2)	-3.67
LOC545056	ubiquitin-conjugating enzyme E2, J2 homolog pseudogene (LOC545056) on chromosome 14.	-3.67
Tead2	TEA domain family member 2 (Tead2)	-3.67
Sord	sorbitol dehydrogenase (Sord)	-3.72
Arhgdig	Rho GDP dissociation inhibitor (GDI) gamma (Arhgdig)	-3.73
Ube2f	ubiquitin-conjugating enzyme E2F (putative) (Ube2f)	-3.74
Serpina10	serine (or cysteine) peptidase inhibitor, clade A (alpha-1 antiproteinase, antitrypsin), member 10 (Serpina10)	-3.76
Fbxo31	F-box protein 31 (Fbxo31)	-3.79
Taf6	TAF6 RNA polymerase II, TATA box binding protein (TBP)-associated factor (Taf6)	-3.82
Clpx	caseinolytic peptidase X (E.coli) (Clpx), transcript variant 1	-3.83
Ppp1r1b	protein phosphatase 1, regulatory (inhibitor) subunit 1B (Ppp1r1b)	-3.87
Smox	spermine oxidase (Smox)	-3.88
Tef	thyrotroph embryonic factor (Tef), transcript variant 1	-3.88
9530027K23Rik	RIKEN 9530027K23Rik cDNA (9530027K23Rik)	-3.91
Mbd1	methyl-CpG binding domain protein 1 (Mbd1)	-3.95
Hdgfrp2	hepatoma-derived growth factor, related protein 2 (Hdgfrp2)	-4.02
LOC100047619	similar to solute carrier family 7 (cationic amino acid transporter, y+ system), member 5 (LOC100047619), misc RNA.	-4.02
Galntl4	UDP-N-acetyl-alpha-D-galactosamine:polypeptide N-acetylgalactosaminyltransferase-like 4 (Galntl4)	-4.05
Ifrd2	interferon-related developmental regulator 2 (Ifrd2)	-4.06
Adora2b	adenosine A2b receptor (Adora2b)	-4.08
Atm	ataxia telangiectasia mutated homolog (human) (Atm)	-4.1
Prodh	proline dehydrogenase (Prodh), nuclear gene encoding mitochondrial protein	-4.1
Sgsm1	small G protein signaling modulator 1 (Sgsm1)	-4.11
Azgp1	alpha-2-glycoprotein 1, zinc (Azgp1)	-4.13
Rab6b	RAB6B, member RAS oncogene family (Rab6b)	-4.13
Xdh	xanthine dehydrogenase (Xdh)	-4.16
Ddr1	discoidin domain receptor family, member 1 (Ddr1), transcript variant 1	-4.18
Slc16a6	solute carrier family 16 (monocarboxylic acid transporters), member 6 (Slc16a6), transcript variant 2	-4.18
Ramp1	receptor (calcitonin) activity modifying protein 1 (Ramp1)	-4.24
Gtf3c1	general transcription factor III C 1 (Gtf3c1)	-4.27
Psmb5	proteasome (prosome, macropain) subunit, beta type 5 (Psmb5)	-4.31
Aldh1a7	aldehyde dehydrogenase family 1, subfamily A7 (Aldh1a7)	-4.36
Reg3d	regenerating islet-derived 3 delta (Reg3d)	-4.39
Erdr1	erythroid differentiation regulator 1 (Erdr1)	-4.4
Gpc1	glypican 1 (Gpc1)	-4.4
Mdm1	transformed mouse 3T3 cell double minute 1 (Mdm1), transcript variant 2	-4.42
5730502D15Rik	RIKEN 5730502D15Rik cDNA (5730502D15Rik)	-4.44
Bcat2	branched chain aminotransferase 2, mitochondrial (Bcat2)	-4.45
Mrpl48	mitochondrial ribosomal protein L48 (Mrpl48), nuclear gene encoding mitochondrial protein	-4.46
Atp2c1	ATPase, Ca++-sequestering (Atp2c1)	-4.47
Hbb-b1	hemoglobin, beta adult major chain (Hbb-b1)	-4.49
Stat3	signal transducer and activator of transcription 3 (Stat3), transcript variant 1	-4.49
LOC100047934	hypothetical protein LOC100047934 (LOC100047934)	-4.52
Actl6b	actin-like 6B (Actl6b)	-4.63
Klk1b24	kallikrein 1-related peptidase b24 (Klk1b24)	-4.63
Kcnf1	potassium voltage-gated channel, subfamily F, member 1 (Kcnf1)	-4.67
Abcf1	ATP-binding cassette, sub-family F (GCN20), member 1 (Abcf1)	-4.68

Spdef	SAM pointed domain containing ets transcription factor (Spdef)	-4.68
3110032G18Rik	RIKEN cDNA 3110032G18 gene (3110032G18Rik)	-4.69
Pex11a	peroxisomal biogenesis factor 11a (Pex11a)	-4.72
Arl5b	ADP-ribosylation factor-like 5B (Arl5b)	-4.74
Gde1	glycerophosphodiester phosphodiesterase 1 (Gde1)	-5.03
1110038D17Rik	RIKEN cDNA 1110038D17 gene (1110038D17Rik)	-5.12
Lcat	lecithin cholesterol acyltransferase (Lcat)	-5.14
6430706D22Rik	RIKEN cDNA 6430706D22 gene (6430706D22Rik)	-5.16
Ykt6	YKT6 homolog (S. Cerevisiae) (Ykt6)	-5.27
Sesn1	sestrin 1 (Sesn1)	-5.34
Dpagt1	dolichyl-phosphate (UDP-N-acetylglucosamine) acetylglucosaminephosphotransferase 1 (GlcNAc-1-P transferase) (Dpagt1)	-5.37
Atad4	ATPase family, AAA domain containing 4 (Atad4)	-5.46
St6galnac2	ST6 (alpha-N-acetyl-neuraminyl-2,3-beta-galactosyl-1, 3)-N-acetylgalactosaminide alpha-2,6-sialyltransferase 2 (St6galnac2)	-5.48
Col7a1	collagen, type VII, alpha 1 (Col7a1)	-5.53
Igfals	insulin-like growth factor binding protein, acid labile subunit (Igfals)	-5.54
Dbp	D site albumin promoter binding protein (Dbp)	-5.62
4933427D14Rik	RIKEN cDNA 4933427D14 gene (4933427D14Rik)	-5.65
Zfp68	zinc finger protein 68 (Zfp68), transcript variant 1	-5.65
1810015C04Rik	RIKEN cDNA 1810015C04 gene (1810015C04Rik), transcript variant 2	-5.92
S3-12	plasma membrane associated protein, S3-12 (S3-12)	-5.92
Srpr	signal recognition particle receptor ('docking protein') (Srpr)	-6.11
Usp2	ubiquitin specific peptidase 2 (Usp2), transcript variant 2	-6.16
Foxa3	forkhead box A3 (Foxa3)	-6.18
Ccrn4l	CCR4 carbon catabolite repression 4-like (S. cerevisiae) (Ccrn4l)	-6.26
Cops8	COP9 (constitutive photomorphogenic) homolog, subunit 8 (Arabidopsis thaliana) (Cops8)	-6.34
Fkbp5	FK506 binding protein 5 (Fkbp5)	-6.35
Igtp	interferon gamma induced GTPase (Igtp)	-6.45
Prom2	prominin 2 (Prom2), transcript variant 2	-6.58
Epm2aip1	EPM2A (laforin) interacting protein 1 (Epm2aip1)	-6.72
Bsdc1	BSD domain containing 1 (Bsdc1)	-7.06
Rpl29	ribosomal protein L29 (Rpl29)	-7.47
Klk1b11	kallikrein 1-related peptidase b11 (Klk1b11)	-7.8
Gbp2	guanylate nucleotide binding protein 2 (Gbp2)	-9.08
Cxcl9	chemokine (C-X-C motif) ligand 9 (Cxcl9)	-9.27
Tom1l2	target of myb1-like 2 (chicken) (Tom1l2), transcript variant 1	-10.39
Tmem25	transmembrane protein 25 (Tmem25)	-10.4
Reg3b	regenerating islet-derived 3 beta (Reg3b)	-10.62
Thumpd1	THUMP domain containing 1 (Thumpd1)	-11.95
Serpini2	serine (or cysteine) peptidase inhibitor, clade I, member 2 (Serpini2)	-12.8

Table S15. Ingenuity Pathway analysis (IPA)-Disease/Bio-Function analysis of the 8 month old GIT2KO pancreatic islet transcriptome. Using the IPA pancreas-specific tissue database the significantly-populated Disease/Bio-Function pathways were extracted with the GIT2KO islet transcriptome. For each specific disease function the enrichment p-value and the specific transcripts populating the function are represented. Transcripts denoted in red were upregulated in GIT2KO islets compared to WT controls: transcripts denoted in green were downregulated in GIT2KO islets compared to WT controls.

Diseases or Functions Annotation	p-Value	Molecules (upregulated – red: downregulated green)
abnormal morphology of beta islet cells	5.98E-04	B2M,IGFBP4,IGFBP5,IRS2,MAPK8IP1,PRKCD,SLC2A2,SYTL4
abnormal morphology of cells	4.59E-03	ATF3,B2M,EHD4,IGFBP4,IGFBP5,IL6,IRS2,MAPK8IP1,PRKCD,RHBDF1,SLC2A2,SYTL4
morphology of pancreas	8.69E-03	ARNTL,ATF3,B2M,EHD4,HES1,IGF2,IGFBP4,IGFBP5,IL6,IRS2,MAPK8IP1,MEN1,PRKCD,RHBDF1,SLC2A2,SYTL4
angiogenesis of islets of Langerhans	8.99E-03	MEN1,OGT
development of pancreas	1.50E-02	ATF3,HES1,HHEX,IL6,IRS2,MEN1,OGT,TGFBR2,VEGFC
abnormal morphology of islets of Langerhans	1.77E-02	ARNTL,ATF3,B2M,EHD4,IGFBP4,IGFBP5,IL6,IRS2,MAPK8IP1,PRKCD,SLC2A2,SYTL4
differentiation of exocrine cells	2.22E-02	HES1,KRAS,TGFBR2
synthesis of nucleotide	2.22E-02	KRAS,NPAS4,VIP
differentiation of duct cells	2.53E-02	HES1,KRAS
survival of organism	3.31E-02	KRAS,SLC2A1,SLC2A2
hyperglycemia	4.70E-02	B2M,IGF2,LPL,MEN1
synthesis of cyclic AMP	4.74E-02	NPAS4,VIP
cytostasis	4.74E-02	KRAS,TGFBR2
survival of islets of Langerhans	4.74E-02	IRF1,IRS2

Table S16. Proteins selectively co-immunoprecipitating with GIT2 in WT pancreatic whole-cell lysates. For each protein identified using LC-MS/MS the Official Gene Symbol, Sf, Score, Coverage, MW (Molecular Weight) and GenPept protein Accession is given.

Protein Description	Gene Symbol	Sf	Score	Coverage	MW	Accession
hypothetical protein LOC229722 [Mus musculus]	5330417C22Rik	0.22	10.13	2.40	99961.5	85701810
RIKEN cDNA A230062G08 [Mus musculus]	Aasdh	0.81	10.13	1.00	121492.0	30348962
acyl-CoA synthetase medium-chain family member 1 [Mus musculus]	Acsm1	0.20	10.14	3.50	64719.1	16905127
actin, alpha 2, smooth muscle, aorta [Mus musculus]	Acta2	0.82	10.13	4.20	41981.8	6671507
actinin alpha 2 [Mus musculus]	Actn2	0.85	10.15	1.20	103853.3	59709449
a disintegrin-like and metalloprotease (reprolysin type) with thrombospondin type 1 motif, 12 [Mus musculus]	Adamts12	0.50	10.20	1.90	177676.5	29789429
amylo-1,6-glucosidase, 4-alpha-glucanotransferase [Mus musculus]	Agl	0.51	10.14	1.40	174174.8	124486747
ankyrin repeat domain 52 [Mus musculus]	Ankrd52	0.30	10.14	1.60	114959.3	27370168
ankyrin repeat and sterile alpha motif domain containing 1 [Mus musculus]	Anks1	0.15	10.11	0.70	125164.9	31088892
adaptor-related protein complex AP-4, epsilon 1 [Mus musculus]	Ap4e1	0.34	10.17	1.90	124766.3	124487335
apolipoprotein A-I [Mus musculus]	Apoa1	0.29	10.14	7.60	30568.7	6753096
ataxin 10 [Mus musculus]	Atxn10	0.15	10.10	1.70	53672.6	83649709
hypothetical protein LOC224171 [Mus musculus]	C330027C09Rik	0.16	10.11	0.80	101978.2	125858491
nuclear domain 10 protein 52 [Mus musculus]	Calcoco2	0.10	10.13	9.40	28092.7	110626078
cask-interacting protein 2 [Mus musculus]	Caskin2	0.40	10.10	0.60	126701.6	31981530
cerebellin 3 precursor protein [Mus musculus]	Cbln3	0.35	10.11	5.60	21063.8	9789903
coiled-coil domain containing 125 [Mus musculus]	Ccdc125	0.32	10.11	1.40	56575.4	55742872
coiled-coil domain containing 93 isoform a [Mus musculus]	Ccdc93	0.25	10.09	1.10	72557.7	68448542
CDK5 regulatory subunit associated protein 2 [Mus musculus]	Cdk5rap2	0.57	10.13	0.70	205816.5	50657347
cyclin-dependent kinase inhibitor 1B [Mus musculus]	Cdkn1b	0.37	10.13	11.70	22179.7	31542372
elastase 2 [Mus musculus]	Cela2a	0.90	10.18	5.90	28895.3	6681297
coiled-coil-helix-coiled-coil-helix domain containing 6 [Mus musculus]	Chchd6	0.21	10.14	8.40	29780.3	27754146
chloride channel calcium activated 4 [Mus musculus]	Clca4	0.20	10.14	2.20	100081.0	20982843
clathrin, heavy polypeptide (Hc) [Mus musculus]	Cltc	0.89	10.14	1.00	191433.7	51491845
CCR4-NOT transcription complex, subunit 2 isoform a [Mus musculus]	Cnot2	0.56	10.15	3.10	59703.0	83745108
trinucleotide repeat containing 5 [Mus musculus]	Cnpy3	0.18	10.13	7.60	30518.6	21312510
hypothetical protein LOC76178 [Mus musculus]	Coa5	0.67	10.15	24.30	8353.0	113461996
procollagen, type XI, alpha 1 [Mus musculus]	Col11a1	0.34	10.11	0.50	180920.2	124487346
procollagen, type XI, alpha 1 [Mus musculus]	Col11a1	0.37	10.09	0.50	180920.2	124487346
cytochrome c oxidase, subunit Va [Mus musculus]	Cox5a	0.93	10.23	20.50	16091.3	112181182
complexin 1 [Mus musculus]	Cplx1	0.29	10.09	5.20	15140.5	31542416
complexin III [Mus musculus]	Cplx3	0.82	10.17	12.70	17574.7	22122785
CASP2 and RIPK1 domain containing adaptor with death domain [Mus musculus]	Cradd	0.28	10.15	9.50	22641.9	6753516
collagen triple helix repeat containing 1 [Mus musculus]	Cthrc1	0.57	10.11	3.30	26443.4	110625696
DEAD (Asp-Glu-Ala-Asp) box polypeptide 23 [Mus musculus]	Ddx23	0.39	10.10	1.30	95436.1	124430514
DEAD/H (Asp-Glu-Ala-Asp/His) box polypeptide 3, X-linked [Mus musculus]	Ddx3x	0.95	10.21	3.30	73056.2	6753620
DEAD (Asp-Glu-Ala-Asp) box polypeptide 5 [Mus musculus]	Ddx5	0.33	10.09	1.50	69222.8	83816893
differentially expressed in FDCP 6 [Mus musculus]	Def6	0.29	10.11	1.60	73408.5	27734752
discs large homolog-associated protein 1 isoform 1 [Mus musculus]	Dlgap1	0.14	10.14	2.10	110304.9	51339023
dedicator of cytokinesis 7 [Mus musculus]	Dock7	0.43	10.13	1.00	237943.7	78191789

dihydropyrimidinase-related protein 4 [Mus musculus]	Dpysl4	0.55	10.15	3.30	61922.6	34328211
dynein, cytoplasmic, heavy chain 1 [Mus musculus]	Dync1h1	0.45	10.13	0.30	531709.6	134288917
eukaryotic translation initiation factor 2B, subunit 2 beta [Mus musculus]	Eif2b2	0.25	10.10	1.70	38872.9	21703888
eukaryotic translation initiation factor 4H [Mus musculus]	Eif4h	0.92	10.15	4.40	27324.4	15808988
E1A binding protein p300 [Mus musculus]	Ep300	0.33	10.13	0.80	263134.4	94421034
ELKS/RAB6-interacting/CAST family member 2 [Mus musculus]	Erc2	0.62	10.18	2.00	115430.8	37360977
E26 avian leukemia oncogene 1, 5' domain isoform 1 [Mus musculus]	Ets1	0.61	10.14	4.10	50169.7	84579952
exonuclease 3''-5'' domain-like 2 [Mus musculus]	Exd2	0.23	10.13	3.80	56749.2	19527014
exocyst complex component 3 [Mus musculus]	Exoc3	0.55	10.14	3.00	86399.7	84579825
fatty acid binding protein 2, intestinal [Mus musculus]	Fabp2	0.90	10.15	9.10	15116.7	6679737
fidgetin [Mus musculus]	Fign	0.15	10.10	0.90	82046.7	11181772
FK506 binding protein 11 [Mus musculus]	Fkbp11	0.91	10.16	5.00	22123.2	15277331
fukutin related protein [Mus musculus]	Fkrp	0.72	10.13	3.00	54817.5	27734120
follistatin-like 5 [Mus musculus]	Fstl5	0.24	10.09	0.90	95755.1	50054054
GA repeat binding protein, alpha [Mus musculus]	Gabpa	0.35	10.09	2.20	51312.2	34328119
hypothetical protein LOC330361 [Mus musculus]	Gcfc2	0.59	10.15	2.50	87387.9	70608163
growth differentiation factor 11 [Mus musculus]	Gdf11	0.46	10.12	2.20	44917.7	112807180
complement factor H-related protein C [Mus musculus]	Gm4788	0.48	10.12	0.90	99360.9	114145728
G protein-coupled receptor 101 [Mus musculus]	Gpr101	0.21	10.13	4.50	56208.5	85701848
glutamate receptor, ionotropic, AMPA 2 isoform 2 [Mus musculus]	Gria2	0.70	10.14	1.70	98726.3	85861224
glutamate receptor, ionotropic, delta 2 [Mus musculus]	Grid2	0.31	10.13	2.00	113010.1	6680091
gtf2ird2 [Mus musculus]	Gtf2ird2	0.39	10.13	2.00	104510.9	16716603
H1 histone family, member 0 [Mus musculus]	H1f0	0.57	10.10	4.10	20848.2	31560697
H3 histone, family 3B [Mus musculus]	H3f3b	1.53	20.24	28.70	15318.5	6680161
histone 1, H2af [Mus musculus]	Hist1h2af	1.54	20.24	20.00	14153.0	30061379
histone 1, H2bh [Mus musculus]	Hist1h2bh	0.94	10.19	11.90	13911.6	30061387
histone 1, H4b [Mus musculus]	Hist1h4b	5.99	70.21	49.50	11360.4	30061405
histone 2, H2ab [Mus musculus]	Hist2h2ab	1.35	20.29	24.60	14004.8	119433657
heterogeneous nuclear ribonucleoprotein D isoform a [Mus musculus]	Hnrnpd	0.41	10.14	4.50	38330.3	116256512
Hermansky-Pudlak syndrome 4 homolog [Mus musculus]	Hps4	0.50	10.13	3.30	72588.3	42476087
interferon gamma induced GTPase [Mus musculus]	Igtp	0.49	10.14	3.50	48448.9	31980875
inhibitor of kappa light polypeptide enhancer in B-cells, kinase complex-associated protein [Mus musculus]	Ikbkap	0.21	10.09	0.70	149349.6	27734086
insulin receptor [Mus musculus]	Insr	0.25	10.12	0.60	155539.6	6754360
insulin receptor substrate 2 [Mus musculus]	Irs2	0.31	8.94	1.20	136763.0	223461918
integrin alpha V [Mus musculus]	Itgav	0.17	10.14	2.00	115205.1	6680486
influenza virus NS1A binding protein isoform 2 [Mus musculus]	Ivns1abp	0.19	10.10	1.60	71534.7	87239990
keratin complex 2, basic, gene 1 [Mus musculus]	Krt1	0.73	10.15	1.90	65565.2	126116585
keratin 5 [Mus musculus]	Krt5	0.51	10.11	2.10	61728.6	20911031
keratin 80 [Mus musculus]	Krt80	0.21	10.14	4.40	50628.9	124249090
keratin 84 [Mus musculus]	Krt84	0.41	10.10	1.20	64946.7	33563238
Leo1, Paf1/RNA polymerase II complex component, homolog [Mus musculus]	Leo1	0.20	10.14	2.80	75552.3	87299619
lamin B2 [Mus musculus]	Lmnb2	0.76	10.12	1.30	67277.4	113195686
hypothetical protein LOC76415 [Mus musculus]	LOC76415	0.31	10.13	5.60	40930.9	30172568
hypothetical protein LOC76787 [Mus musculus]	LOC76787	0.49	10.13	2.00	116208.2	58037461
low density lipoprotein receptor-related protein 1 [Mus musculus]	Lrp1	0.16	10.13	0.20	504439.1	124494256
leucine rich repeat containing 40 [Mus musculus]	Lrrc40	0.20	10.11	1.20	68006.3	31541911
leucine rich repeat containing 59 [Mus musculus]	Lrrc59	0.84	10.15	6.80	34855.8	19527026

LSM12 homolog [Mus musculus]	Lsm12	0.25	10.13	10.30	21687.1	31711990
melanoma antigen family A, 9 [Mus musculus]	Maged2	0.12	10.13	8.60	27180.7	32469505
microtubule-associated protein 1 A [Mus musculus]	Map1a	0.40	10.10	0.30	325686.1	124244033
methionine-tRNA synthetase [Mus musculus]	Mars	0.20	10.10	1.10	101366.3	51491852
macrophage stimulating 1 receptor [Mus musculus]	Mst1r	0.32	10.13	1.30	150442.4	6677765
misato [Mus musculus]	Msto1	0.45	10.17	3.20	61183.0	21450273
melanoma associated antigen (mutated) 1-like 1 [Mus musculus]	Mum1l1	0.52	10.11	1.20	66747.4	29789431
myosin, light polypeptide 6B [Mus musculus]	Myl6b	0.04	10.10	5.30	22734.7	26986555
myosin VIIa [Mus musculus]	Myo7a	0.21	10.16	0.30	250936.1	115511010
NCK-associated protein 1 [Mus musculus]	Nckap1	0.36	10.13	2.00	128700.5	28395023
neurofibromin [Mus musculus]	Nf1	0.33	10.10	0.40	319388.9	34878892
neurotrophin 5 [Mus musculus]	Ntf5	0.41	10.13	9.10	22331.5	38016142
Nedd8 ultimate buster-1 [Mus musculus]	Nub1	0.69	10.14	2.30	70262.8	119360354
nuclear distribution gene C homolog [Mus musculus]	Nudc	0.68	10.14	3.90	38334.3	6754910
hypothetical protein LOC52014 [Mus musculus]	Nus1	0.58	10.13	6.40	33464.2	13384840
OCIA domain containing 1 [Mus musculus]	Ociad1	0.09	10.11	0.00	27592.9	12963675
olfactory receptor 601 [Mus musculus]	Olfr601	0.21	10.10	0.00	34933.2	22129661
olfactory receptor 644 [Mus musculus]	Olfr644	0.33	10.10	0.00	35488.5	22128755
alcohol dehydrogenase PAN1B-like [Mus musculus]	Pan1b	1.43	20.23	9.50	33496.9	37574136
3'-phosphoadenosine 5'-phosphosulfate synthase 2 [Mus musculus]	Papss2	0.08	10.13	3.40	70306.0	61098088
protocadherin beta 13 [Mus musculus]	Pcdhb13	0.67	10.15	2.50	87534.6	18087789
p53 and DNA damage regulated 1 [Mus musculus]	Pdrg1	0.65	10.14	15.00	15372.2	32490570
pyridoxal (pyridoxine, vitamin B6) kinase [Mus musculus]	Pdxk	0.45	10.14	0.00	34992.7	26006861
PDZ domain containing RING finger 3 [Mus musculus]	Pdzrn3	0.43	10.15	1.30	119436.9	9256642
6-phosphofructo-2-kinase/fructose-2,6-biphosphatase 2 [Mus musculus]	Pfkfb2	0.19	10.13	3.70	59778.5	34147045
1-phosphatidylinositol-4-phosphate 5-kinase [Mus musculus]	Pikfyve	0.36	10.12	0.30	231931.0	115529473
polyductin [Mus musculus]	Pkhd1	0.08	10.11	0.20	444599.6	126157466
plexin A4 [Mus musculus]	Plxna4	0.51	10.14	0.80	212092.8	28461143
polynucleotide kinase 3'- phosphatase [Mus musculus]	Pnkp	0.23	10.09	1.30	57178.7	118601009
proline-rich nuclear receptor coactivator 1 [Mus musculus]	Pnrc1	0.12	10.14	7.40	32326.3	113461982
protein arginine N-methyltransferase 7 [Mus musculus]	Prmt7	0.36	10.11	1.20	78250.8	21703808
protease, serine, 1 [Mus musculus]	Prss1	0.93	10.17	8.10	26117.8	16716569
pleckstrin and Sec7 domain containing homolog [Mus musculus]	Psd	0.16	10.11	0.70	109619.3	51317392
pre T-cell antigen receptor alpha [Mus musculus]	Ptcra	0.20	10.11	3.40	22357.8	6755216
pseudouridylate synthase 7 homolog (S. cerevisiae)-like [Mus musculus]	Pus7l	0.55	10.16	2.40	79147.5	27369583
periodic tryptophan protein 1 homolog [Mus musculus]	Pwp2	0.58	10.09	1.20	55580.1	19923062
retinoic acid induced 1 [Mus musculus]	Rai1	0.37	10.14	1.20	201443.9	83649747
RB-associated KRAB repressor [Mus musculus]	Rbak	0.34	10.11	1.30	81622.1	113865971
retinol dehydrogenase 13 (all-trans and 9-cis) [Mus musculus]	Rdh13	0.81	10.21	0.00	36441.1	30425078
rhomboid-like protein 6 [Mus musculus]	Rhbdf2	0.88	10.18	2.10	93358.8	27369806
ribosomal protein L18 [Mus musculus]	Rpl18	1.86	20.16	13.80	21631.2	83699424
ribosomal protein L19 [Mus musculus]	Rpl19	0.97	10.21	8.70	23467.3	6677773
ribosomal protein L22 like 1 [Mus musculus]	Rpl22l1	0.83	10.13	9.80	14458.6	13386010
ribosomal protein L23 [Mus musculus]	Rpl23	1.59	20.15	27.10	14856.1	12584986
ribosomal protein L4 [Mus musculus]	Rpl4	3.21	40.17	9.80	47124.1	30794450
ribosomal protein L7 [Mus musculus]	Rpl7	0.91	10.17	6.70	31399.8	31981515
ribosomal protein L8 [Mus musculus]	Rpl8	0.74	10.14	4.30	28007.3	6755358
ribosomal protein L9 [Mus musculus]	Rpl9	1.88	20.23	17.20	21867.8	14149647

ribosomal protein, large P2 [Mus musculus]	Rplp2	0.97	10.23	13.90	11643.8	83745120
ribophorin II [Mus musculus]	Rpn2	1.81	20.15	4.60	69020.2	34996495
ribosomal protein S13 [Mus musculus]	Rps13	1.76	20.14	8.60	17211.7	13386034
ribosomal protein S3 [Mus musculus]	Rps3	0.81	10.14	3.70	26657.4	6755372
ribosomal protein S8 [Mus musculus]	Rps8	3.32	40.19	22.60	24190.2	6677813
ribosome binding protein 1 isoform b [Mus musculus]	Rrp1b	0.97	10.19	2.60	72425.0	19482168
sodium channel, voltage-gated, type I, alpha [Mus musculus]	Scn1a	0.48	10.09	0.50	227467.9	125630315
solute carrier family 9 (sodium/hydrogen exchanger), member 2 [Mus musculus]	Slc9a2	0.26	10.13	0.00	91587.7	75832049
SWI/SNF related, matrix associated, actin dependent regulator of chromatin, subfamily a, member 2 isoform 1 [Mus musculus]	Smarca2	0.07	10.10	0.60	180608.6	51593084
structural maintenance of chromosomes 5 [Mus musculus]	Smc5	0.35	10.14	2.10	127301.7	24497433
sushi, nidogen and EGF-like domains 1 [Mus musculus]	Sned1	0.46	10.15	1.10	151564.1	40254325
signal peptidase complex subunit 3 [Mus musculus]	Spcs3	0.93	10.17	6.70	20300.5	125988403
sperm antigen with calponin homology and coiled-coil domains 1 [Mus musculus]	Specc1	0.66	10.12	0.70	108777.1	71979930
sphingosine kinase type 1-interacting protein [Mus musculus]	Sphkap	0.17	10.15	1.10	181987.3	66773173
signal sequence receptor, delta [Mus musculus]	Ssr4	0.72	10.14	11.00	18924.5	6678145
serine/threonine kinase 17b (apoptosis-inducing) [Mus musculus]	Stk17b	0.49	10.13	3.50	41956.1	31559988
syntaxin 7 [Mus musculus]	Stx7	0.35	10.10	2.70	29718.2	31560462
synaptonemal complex protein 1 [Mus musculus]	Sycp1	0.54	10.13	1.80	115890.5	45597449
TAF1 RNA polymerase II, TATA box binding protein (TBP)-associated factor [Mus musculus]	Taf1	0.59	10.15	0.90	215715.4	124486596
tektin 4 [Mus musculus]	Tekt4	0.49	10.14	3.60	52013.2	110625871
thrombospondin 1 [Mus musculus]	Thbs1	0.26	10.14	1.90	129606.4	47059073
thrombospondin, type I, domain containing 7B [Mus musculus]	Thsd7b	0.08	10.15	1.10	179189.2	40254219
hypothetical protein LOC319776 [Mus musculus]	Tmem72	0.76	10.15	7.30	30352.7	31982359
translocase of outer mitochondrial membrane 34 [Mus musculus]	Tomm34	0.28	10.11	2.90	34258.8	13385500
testis-specific serine kinase 1 [Mus musculus]	Tssk1	0.27	10.13	5.50	41562.4	112734851
hypothetical protein LOC218343 [Mus musculus]	Ttc37	0.07	10.10	0.60	173831.0	124486883
tubulin tyrosine ligase-like family, member 4 [Mus musculus]	Ttll4	0.19	10.10	0.50	132447.2	62510079
synaptobrevin like 1 [Mus musculus]	Vamp7	0.64	10.11	3.20	24951.0	33468929
vaccinia related kinase 2 [Mus musculus]	Vrk2	0.19	10.13	4.20	58108.0	21312468
WD repeat domain 18 [Mus musculus]	Wdr18	0.55	10.14	0.00	47181.1	83649741
WD repeat domain 34 [Mus musculus]	Wdr34	0.66	10.20	5.80	58137.3	139948827
WW domain-containing protein 1 [Mus musculus]	Wwp1	0.48	10.09	0.90	104628.0	112734836
cardiomyopathy associated 3 isoform 1 [Mus musculus]	Xirp2	0.46	10.11	0.20	427991.7	66841385
hypothetical protein LOC214779 [Mus musculus]	Zfp879	0.44	10.13	3.00	64024.8	27734192
zinc finger protein 94 [Mus musculus]	Zfp94	0.45	10.09	1.70	54799.2	6677631
zinc finger, MYM-type 6 [Mus musculus]	Zmym6	0.31	10.13	1.70	139249.8	29126191

Table S17. Proteins selectively co-immunoprecipitating with GIT2 in *db/db* pancreatic whole-cell lysates. For each protein identified using LC-MS/MS the Official Gene Symbol, Sf, Score, Coverage, MW (Molecular Weight) and GenPept protein Accession is given.

Protein Description	Gene Symbol	Sf	Score	Coverage	MW	Accession
RIKEN cDNA A230062G08 [Mus musculus]	Aasdh	0.81	10.13	1.00	121492.0	30348962
acyl-CoA synthetase medium-chain family member 1 [Mus musculus]	Acsm1	0.21	10.14	0.00	64719.1	16905127
actin, alpha 2, smooth muscle, aorta [Mus musculus]	Acta2	0.79	10.13	4.20	41981.8	6671507
RIKEN cDNA 2610208M17 gene [Mus musculus]	Aida	0.03	9.12	0.84	34888.05	32189430
adaptor-related protein complex AP-4, epsilon 1 [Mus musculus]	Ap4e1	0.34	10.17	1.90	124766.3	124487335
apolipoprotein A-I [Mus musculus]	Apoa1	0.29	10.14	7.60	30568.7	6753096
ataxin 10 [Mus musculus]	Atxn10	0.15	10.10	1.70	53672.6	83649709
hypothetical protein LOC224171 [Mus musculus]	C330027C09Rik	0.16	10.11	0.80	101978.2	125858491
cask-interacting protein 2 [Mus musculus]	Caskin2	0.40	10.10	0.60	126701.6	31981530
coiled-coil domain containing 93 isoform a [Mus musculus]	Ccdc93	0.25	10.09	1.10	72557.7	68448542
CDK5 regulatory subunit associated protein 2 [Mus musculus]	Cdk5rap2	0.57	10.13	0.70	205816.5	50657347
cyclin-dependent kinase inhibitor 1B [Mus musculus]	Cdkn1b	0.37	10.13	11.70	22179.7	31542372
elastase 2 [Mus musculus]	Cela2a	0.90	10.18	5.90	28895.3	6681297
coiled-coil-helix-coiled-coil-helix domain containing 6 [Mus musculus]	Chchd6	0.21	10.14	8.40	29780.3	27754146
chromodomain helicase DNA binding protein 2 isoform 5 [Mus musculus]	Chd5	0.33	9.12	0	218953.5	189458814
chloride channel calcium activated 4 [Mus musculus]	Clca3b	0.20	10.14	2.20	100081.0	20982843
trinucleotide repeat containing 5 [Mus musculus]	Cnpy3	0.18	10.13	7.60	30518.6	21312510
hypothetical protein LOC76178 [Mus musculus]	Coa5	0.67	10.15	24.30	8353.0	113461996
procollagen, type XI, alpha 1 [Mus musculus]	Col11a1	0.21	9.09	4.08	178694.39	148680461
complexin 1 [Mus musculus]	Cplx1	0.29	10.09	5.20	15140.5	31542416
CASP2 and RIPK1 domain containing adaptor with death domain [Mus musculus]	Cradd	0.28	10.15	9.50	22641.9	6753516
collagen triple helix repeat containing 1 [Mus musculus]	Cthrc1	0.57	10.11	3.30	26443.4	110625696
DEAD (Asp-Glu-Ala-Asp) box polypeptide 23 [Mus musculus]	Ddx23	0.39	10.10	1.30	95436.1	124430514
DEAD (Asp-Glu-Ala-Asp) box polypeptide 5 [Mus musculus]	Ddx5	0.33	10.09	1.50	69222.8	83816893
discs large homolog-associated protein 1 isoform 1 [Mus musculus]	Dlgap1	0.14	10.14	2.10	110304.9	51339023
dedicator of cytokinesis 7 [Mus musculus]	Dock7	0.43	10.13	1.00	237943.7	78191789
dynein, cytoplasmic, heavy chain 1 [Mus musculus]	Dync1h1	0.45	10.13	0.30	531709.6	134288917
eukaryotic translation initiation factor 4H [Mus musculus]	Eif4h	0.92	10.15	4.40	27324.4	15808988
ELKS/RAB6-interacting/CAST family member 2 [Mus musculus]	Erc2	0.62	10.18	2.00	115430.8	37360977
exonuclease 3''-5'' domain-like 2 [Mus musculus]	Exd2	0.23	10.13	3.80	56749.2	19527014
exocyst complex component 3 [Mus musculus]	Exoc3	0.55	10.14	3.00	86399.7	84579825
fau [Mus musculus]	Fau	0.45	9.10	1.44	14415.68	497611
F-box only protein 24 [Mus musculus]	Fbxo24	0.03	9.12	0.84	65,429	254553394
fidgetin [Mus musculus]	Fign	0.15	10.10	0.90	82046.7	11181772
FK506 binding protein 11 [Mus musculus]	Fkbp11	0.91	10.16	5.00	22123.2	15277331
follistatin-like 5 [Mus musculus]	Fstl5	0.24	10.09	0.90	95755.1	50054054
GA repeat binding protein, alpha [Mus musculus]	Gabpa	0.35	10.09	2.20	51312.2	34328119
glutamate decarboxylase-like 1 [Mus musculus]	Gadl1	0.64	9.12	0.96	57144.12	197382957
Glyceraldehyde-3-phosphate dehydrogenase (GAPDH) [Mus musculus]	Gapdh	0.56	9.13	0	35810.01	6679937
growth differentiation factor 11 [Mus musculus]	Gdf11	0.46	10.12	2.20	44917.7	112807180

ribosomal protein L27a-like [Mus musculus]	Gm14407	0.24	9.12	6.6	16440.18	309264679
complement factor H-related protein C [Mus musculus]	Gm4788	0.48	10.12	0.90	99360.9	114145728
Gprin1 protein [Mus musculus]	Gprin1	1.01	9.19	0	95495.9	34784265
glutamate receptor, ionotropic, delta 2 [Mus musculus]	Grid2	0.31	10.13	2.00	113010.1	6680091
glutathione S-transferase pi class A [Mus musculus]	Gstp2	0.84	9.14	3	23537.03	577419
gtf2ird2 [Mus musculus]	Gtf2ird2	0.39	10.13	2.00	104510.9	16716603
H3 histone, family 3B [Mus musculus]	H3f3b	1.53	20.24	28.70	15318.5	6680161
histone 1, H2af [Mus musculus]	Hist1h2af	1.54	20.24	20.00	14153.0	30061379
histone 1, H4b [Mus musculus]	Hist1h4b	5.39	60.24	49.50	11360.4	30061405
histone 2, H2ab [Mus musculus]	Hist2h2ab	1.35	20.29	24.60	14004.8	119433657
heterogeneous nuclear ribonucleoprotein D isoform a [Mus musculus]	Hnrnpd	0.41	10.14	4.50	38330.3	116256512
inhibitor of kappa light polypeptide enhancer in B-cells, kinase complex-associated protein [Mus musculus]	Ikbkap	0.21	10.09	0.70	149349.6	27734086
insulin receptor [Mus musculus]	Ikbkap	0.17	10.10	0.60	155539.6	6754360
influenza virus NS1A binding protein isoform 2 [Mus musculus]	Ivns1abp	0.19	10.10	1.60	71534.7	87239990
KIAA1683 protein [Mus musculus]	KIAA1683	0.83	9.11	0.84	140835	149258880
Kifc1 protein [Mus musculus]	Kifc1	0.21	9.14	1.32	74068.92	71679893
Kelch-like protein 3 [Mus musculus]	Klhl3	1.19	9.15	8.28	70758.04	303519514
killer cell lectin-like receptor subfamily A member 33 [Mus musculus]	Klra33	1.04	9.12	11.76	30738.72	84993761
keratin complex 2, basic, gene 1 [Mus musculus]	Krt1	0.73	10.15	1.90	65565.2	126116585
keratin 5 [Mus musculus]	Krt5	0.51	10.11	2.10	61728.6	20911031
keratin 80 [Mus musculus]	Krt80	0.21	10.14	4.40	50628.9	124249090
keratin 84 [Mus musculus]	Krt84	0.41	10.10	1.20	64946.7	33563238
Leo1, Paf1/RNA polymerase II complex component, homolog [Mus musculus]	Leo1	0.20	10.14	2.80	75552.3	87299619
alcohol dehydrogenase PAN1B-like [Mus musculus]	LOC638245	1.43	20.23	9.50	33496.9	37574136
hypothetical protein LOC76415 [Mus musculus]	LOC76415	0.31	10.13	5.60	40930.9	30172568
low density lipoprotein receptor-related protein 1 [Mus musculus]	Lrp1	0.16	10.13	0.20	504439.1	124494256
leucine rich repeat containing 40 [Mus musculus]	Lrrc40	0.20	10.11	1.20	68006.3	31541911
leucine rich repeat containing 59 [Mus musculus]	Lrrc59	0.84	10.15	6.80	34855.8	19527026
LSM12 homolog [Mus musculus]	Lsm12	0.25	10.13	10.30	21687.1	31711990
melanoma antigen family A, 9 [Mus musculus]	Maged2	0.12	10.13	8.60	27180.7	32469505
microtubule-associated protein 1 A [Mus musculus]	Map1a	0.40	10.10	0.30	325686.1	124244033
methionine-tRNA synthetase [Mus musculus]	Mars	0.20	10.10	1.10	101366.3	51491852
multiple EGF-like-domains 8 [Mus musculus]	Megf8	0.29	9.08	1.56	297487.34	189485002
KIAA0026 [Mus musculus]	Morf4l2	1.21	9.19	10.44	32183.71	59798476
misato [Mus musculus]	Msto1	0.45	10.17	3.20	61183.0	21450273
melanoma associated antigen (mutated) 1-like 1 [Mus musculus]	Mum1l1	0.52	10.11	1.20	66747.4	29789431
myosin, light polypeptide 6B [Mus musculus]	Myl6b	0.04	10.10	5.30	22734.7	26986555
myosin VIIa [Mus musculus]	Myo7a	0.21	10.16	0.30	250936.1	115511010
NCK-associated protein 1 [Mus musculus]	Nckap1	0.36	10.13	2.00	128700.5	28395023
neurofibromin [Mus musculus]	Nf1	0.33	10.10	0.40	319388.9	34878892
neurotrophin 5 [Mus musculus]	Ntf5	0.41	10.13	9.10	22331.5	38016142
nuclear distribution gene C homolog [Mus musculus]	Nudc	0.68	10.14	3.90	38334.3	6754910
olfactory receptor 601 [Mus musculus]	Olfr601	0.21	10.10	0.00	34933.2	22129661
olfactory receptor 644 [Mus musculus]	Olfr644	0.33	10.10	0.00	35488.5	22128755
3'-phosphoadenosine 5'-phosphosulfate synthase 2 [Mus musculus]	Papss2	0.08	10.13	3.40	70306.0	61098088
protocadherin beta 13 [Mus musculus]	Pcdhb13	0.67	10.15	2.50	87534.6	18087789

p53 and DNA damage regulated 1 [Mus musculus]	Pdrg1	0.65	10.14	15.00	15372.2	32490570
pyridoxal (pyridoxine, vitamin B6) kinase [Mus musculus]	Pdxk	0.45	10.14	0.00	34992.7	26006861
Membrane-associated progesterone receptor component 2 isoform 3 [Mus musculus]	Pgrmc2	3.89	36.16	11.76	23334.13	226442772
1-phosphatidylinositol-4-phosphate 5-kinase [Mus musculus]	Pikfyve	0.36	10.12	0.30	231931.0	115529473
polyductin [Mus musculus]	Pkhd1	0.08	10.11	0.20	444599.6	126157466
hypothetical protein isoform 4 [Mus musculus]	Plbd2	0.64	9.12	0.96	66289.46	146324958
plexin A4 [Mus musculus]	Plxna4	0.51	10.14	0.80	212092.8	28461143
polynucleotide kinase 3'- phosphatase [Mus musculus]	Pnkp	0.23	10.09	1.30	57178.7	118601009
proline-rich nuclear receptor coactivator 1 [Mus musculus]	Pnrc1	0.12	10.14	7.40	32326.3	113461982
PR domain containing 15 [Mus musculus]	Prdm15	0.10	9.10	0.24	132870.27	226531251
protein arginine N-methyltransferase 7 [Mus musculus]	Prmt7	0.36	10.11	1.20	78250.8	21703808
protease, serine, 1 [Mus musculus]	Prss1	0.93	10.17	8.10	26117.8	16716569
pleckstrin and Sec7 domain containing homolog [Mus musculus]	Psd	0.02	10.13	0.70	109619.3	51317392
pre T-cell antigen receptor alpha [Mus musculus]	Ptcra	0.17	10.10	3.40	22357.8	6755216
periodic tryptophan protein 1 homolog [Mus musculus]	Pwp2	0.58	10.09	1.20	55580.1	19923062
retinol dehydrogenase 13 (all-trans and 9-cis) [Mus musculus]	Rdh13	0.81	10.21	0.00	36441.1	30425078
Ac2-210 [Mus musculus]	RGD1359290	1.16	9.15	8.04	25077.31	114145792
ribosomal protein L11 [Mus musculus]	Rpl11	1.06	9.13	4.92	20252.39	47682920
ribosomal protein L15 [Mus musculus]	Rpl15	0.90	9.11	5.16	24146.07	72679859
Rpl17 protein [Mus musculus]	Rpl17	1.01	9.12	1.2	21397.02	31324951
ribosomal protein L18 [Mus musculus]	Rpl18	0.95	10.17	6.90	21631.2	83699424
ribosomal protein L19 [Mus musculus]	Rpl19	0.97	10.21	8.70	23467.3	6677773
ribosomal protein L21 [Mus musculus]	Rpl21	0.09	9.09	0.72	18590.92	66792597
ribosomal protein L22 like 1 [Mus musculus]	Rpl22l1	0.83	10.13	9.80	14458.6	13386010
ribosomal protein L23 [Mus musculus]	Rpl23	0.96	10.24	14.30	14856.1	12584986
ribosomal protein L23a [Mus musculus]	Rpl23a	0.80	9.10	3.84	17695.06	33317318
ribosomal protein L26 [Mus musculus]	Rpl26	0.40	9.10	3.24	17258.21	71051411
ribosomal protein L3 [Mus musculus]	Rpl3	2.35	18.20	20.64	46109.92	187956900
ribosomal protein L37a [Mus musculus]	Rpl37a	1.01	9.12	4.44	10275.25	312414
ribosomal protein L38 [Mus musculus]	Rpl38	2.40	18.19	14.4	8203.86	63101642
ribosomal protein L4 [Mus musculus]	Rpl4	3.11	40.18	9.80	47124.1	30794450
ribosomal protein L6 [Mus musculus]	Rpl6	0.88	9.13	16.68	33509.63	77415464
ribosomal protein L7 [Mus musculus]	Rpl7	0.85	10.14	4.10	31399.8	31981515
ribosomal protein L8 [Mus musculus]	Rpl8	0.72	10.12	4.30	28007.3	6755358
60S ribosomal protein L9 isoform 2 [Mus musculus]	Rpl9	0.19	9.09	0.6	21881.43	7862171
ribosomal protein L6 isoform 1 [Mus musculus]	Rpl9	0.46	9.14	3.6	21881.43	148705791
ribosomal protein L9 [Mus musculus]	Rpl9	1.88	20.23	17.20	21867.8	14149647
Acidic ribosomal phosphoprotein P0 [Mus musculus]	Rplp0	0.15	9.12	8.88	34216.41	6671569
acidic ribosomal phosphoprotein P1 isoform 1 [Mus musculus]	Rplp1	1.16	9.16	9.72	11474.9	902558
ribosomal protein, large P2 [Mus musculus]	Rplp2	0.70	10.15	13.90	11643.8	83745120
LOC665931 protein isoform 1 [Mus musculus]	Rplp2-ps1	1.20	9.22	17.16	379518.14	130529
ribosomal protein S3 [Mus musculus]	Rps3	0.81	10.14	3.70	26657.4	6755372
ribosomal protein S7 isoform 1 [Mus musculus]	Rps7	1.21	9.17	3.12	22126.85	72679428
ribosomal protein S8 [Mus musculus]	Rps8	1.92	20.21	12.00	24190.2	6677813
ribosome binding protein 1 isoform b [Mus musculus]	Rrbp1	0.97	10.19	2.60	72425.0	19482168

ribosomal RNA processing 1 homolog B [Mus musculus]	Rrp1b	0.37	10.16	3.00	80505.1	28076997
Solute carrier family 45, member 4 [Mus musculus]	Slc45a4	0.61	9.12	4.2	85765.11	112180534
solute carrier family 9 (sodium/hydrogen exchanger), member 2 [Mus musculus]	Slc9a2	0.26	10.13	0.00	91587.7	75832049
SWI/SNF related, matrix associated, actin dependent regulator of chromatin, subfamily a, member 2 isoform 1 [Mus musculus]	Smarca2	0.07	10.10	0.60	180608.6	51593084
hypothetical protein isoform 1 [Mus musculus]	Sned1	0.10	9.10	0.24	151580.57	158563954
signal peptidase complex subunit 3 [Mus musculus]	Spcs3	0.93	10.17	6.70	20300.5	125988403
sperm antigen with calponin homology and coiled-coil domains 1 [Mus musculus]	Specc1	0.66	10.12	0.70	108777.1	71979930
sphingosine kinase type 1-interacting protein [Mus musculus]	Sphkap	0.17	10.15	1.10	181987.3	66773173
serine/threonine kinase 17b (apoptosis-inducing) [Mus musculus]	Stk17b	0.49	10.13	3.50	41956.1	31559988
syntaxin 7 [Mus musculus]	Stx7	0.32	10.11	2.70	29718.2	31560462
thrombospondin 1 [Mus musculus]	Thbs1	0.26	10.14	1.90	129606.4	47059073
hypothetical protein LOC319776 [Mus musculus]	Tmem72	0.76	10.15	7.30	30352.7	31982359
translocase of outer mitochondrial membrane 34 [Mus musculus]	Tomm34	0.28	10.11	2.90	34258.8	13385500
testis-specific serine kinase 1 [Mus musculus]	Tssk1	0.19	10.13	5.50	41562.4	112734851
4930401A09Rik protein [Mus musculus]	Ttc23l	0.21	9.09	4.08	51532.07	254281218
tubulin tyrosine ligase-like family, member 4 [Mus musculus]	Ttll4	0.19	10.10	0.50	132447.2	62510079
synaptobrevin like 1 [Mus musculus]	Vamp7	0.64	10.11	3.20	24951.0	33468929
vomeronasal 1 receptor, H1 [Mus musculus]	Vmn1r-ps103	0.29	9.08	1.56	36943.78	256773238
vaccinia related kinase 2 [Mus musculus]	Vrk2	0.19	10.13	4.20	58108.0	21312468
WD repeat domain 18 [Mus musculus]	Wdr18	0.55	10.14	0.00	47181.1	83649741
WD repeat domain containing 82 isoform 7 [Mus musculus]	Wdr19	0.19	9.09	0.6	151456.71	94730677
WD repeat domain 34 [Mus musculus]	Wdr34	0.66	10.20	5.80	58137.3	139948827
WW domain-containing protein 1 [Mus musculus]	Wwp1	0.48	10.09	0.90	104628.0	112734836
hypothetical protein LOC214779 [Mus musculus]	Zfp879	0.44	10.13	3.00	64024.8	27734192
zinc finger, MYM-type 6 [Mus musculus]	Zmym6	0.31	10.13	1.70	139249.8	29126191

Table S18. Venn diagram analysis of co-IP proteins from WT or *db/db* pancreatic lysates. The Official Gene Symbol for each specific protein from the denoted Venn sectors is indicated.

Common WT-*db/db*	WT only	*db/db* only
Aasdh	5330417C22Rik	Aida
Acsm1	Actn2	Chd5
Acta2	Adamts12	Clca3b
Ap4e1	Agl	Fau
Apoa1	Ankrd52	Fbxo24
Atxn10	Anks1	Gadl1
C330027C09Rik	Calcoco2	Gapdh
Caskin2	Cbln3	Gm14407
Ccdc93	Ccdc125	Gprin1
Cdk5rap2	Clca4	Gstp2
Cdkn1b	Cltc	KIAA1683
Cela2a	Cnot2	Kifc1
Chchd6	Cox5a	Klhl3
Cnpy3	Cplx3	Klra33
Coa5	Ddx3x	LOC638245
Col11a1	Def6	Megf8
Cplx1	Dpysl4	Morf4l2
Cradd	Eif2b2	Pgrmc2
Cthrc1	Ep300	Plbd2
Ddx23	Ets1	Prdm15
Ddx5	Fabp2	RGD1359290
Dlgap1	Fkrp	Rpl11
Dock7	Gcfc2	Rpl15
Dync1h1	Gpr101	Rpl17
Eif4h	Gria2	Rpl21
Erc2	H1f0	Rpl23a
Exd2	Hist1h2bh	Rpl26
Exoc3	Hps4	Rpl3
Fign	Igtp	Rpl37a
Fkbp11	Insr	Rpl38
Fstl5	Irs2	Rpl6
Gabpa	Itgav	Rplp0
Gdf11	Lmnb2	Rplp1
Gm4788	LOC76787	Rplp2-ps1
Grid2	Mst1r	Rps7
Gtf2ird2	Nub1	Rrbp1
H3f3b	Nus1	Slc45a4
Hist1h2af	Ociad1	Ttc23l
Hist1h4b	PAn1b	Vmn1r-ps103
Hist2h2ab	Pan1b	Wdr19
Hnrnpd	Pdzrn3	
Ikbkap	Pfkfb2	

Ivns1abp	Pus7l	
Krt1	Rai1	
Krt5	Rbak	
Krt80	Rhbdf2	
Krt84	Rpn2	
Leo1	Rps13	
LOC76415	Scn1a	
Lrp1	Smc5	
Lrrc40	Ssr4	
Lrrc59	Sycp1	
Lsm12	Taf1	
Maged2	Tekt4	
Map1a	Thsd7b	
Mars	Ttc37	
Msto1	Xirp2	
Mum1l1	Zfp94	
Myl6b		
Myo7a		
Nckap1		
Nf1		
Ntf5		
Nudc		
Olfr601		
Olfr644		
Papss2		
Pcdhb13		
Pdrg1		
Pdxk		
Pikfyve		
Pkhd1		
Plxna4		
Pnkp		
Pnrc1		
Prmt7		
Prss1		
Psd		
Ptcra		
Pwp2		
Rdh13		
Rpl18		
Rpl19		
Rpl22l1		
Rpl23		
Rpl4		
Rpl7		
Rpl8		
Rpl9		
Rplp2		

Rps3		
Rps8		
Rrp1b		
Slc9a2		
Smarca2		
Sned1		
Spcs3		
Specc1		
Sphkap		
Stk17b		
Stx7		
Thbs1		
Tmem72		
Tomm34		
Tssk1		
Ttll4		
Vamp7		
Vrk2		
Wdr18		
Wdr34		
Wwp1		
Zfp879		
Zmym6		

Table S19. KEGG pathway analysis of proteins selectively co-immunoprecipitating with GIT2 in WT pancreatic whole-cell lysates. Significantly-populated KEGG signaling pathways, generated using the proteins co-immunoprecipitating with GIT2 in WT pancreatic whole-cell lysates are depicted. For each specific KEGG pathway annotation the following parameter indices are indicated: **C** - total background number of transcripts populating the KEGG pathway; **O** – number of observed transcripts within the input dataset that are contained within the specific KEGG pathway; **E** – number of transcripts from the input dataset expected to be present at background levels; **R** – transcript enrichment factor in specific KEGG pathway, **P** – enrichment probability; **H** – hybrid score = $-\log_{10}P * R$.

KEGG Pathway	C	O	E	R	P	H
Ribosome	119	12	0.33	36.02	4.48E-14	4.81E+02
Selenocompound metabolism	17	2	0.05	42.02	0.0128	7.95E+01
Systemic lupus erythematosus	149	6	0.42	14.38	8.66E-05	5.84E+01
SNARE interactions in vesicular transport	35	2	0.1	20.41	0.0293	3.13E+01
Adherens junction	75	3	0.21	14.29	0.0128	2.70E+01
Aldosterone-regulated sodium reabsorption	44	2	0.12	16.23	0.0316	2.44E+01
Fat digestion and absorption	45	2	0.13	15.87	0.0316	2.38E+01
Malaria	46	2	0.13	15.53	0.0316	2.33E+01
Type II diabetes mellitus	49	2	0.14	14.58	0.0316	2.19E+01
Notch signaling pathway	50	2	0.14	14.29	0.0316	2.14E+01
Phagosome	176	4	0.49	8.12	0.0128	1.54E+01
Long-term potentiation	69	2	0.19	10.35	0.0424	1.42E+01
Renal cell carcinoma	71	2	0.2	10.06	0.0424	1.38E+01
Long-term depression	72	2	0.2	9.92	0.0424	1.36E+01
RNA degradation	76	2	0.21	9.4	0.0424	1.29E+01
Protein digestion and absorption	78	2	0.22	9.16	0.0424	1.26E+01
PPAR signaling pathway	80	2	0.22	8.93	0.0424	1.23E+01
TGF-beta signaling pathway	85	2	0.24	8.4	0.0424	1.15E+01
ECM-receptor interaction	86	2	0.24	8.31	0.0424	1.14E+01
Prostate cancer	89	2	0.25	8.03	0.0433	1.09E+01
Huntington's disease	197	3	0.55	5.44	0.0424	7.47E+00
Focal adhesion	200	3	0.56	5.36	0.0424	7.36E+00
Regulation of actin cytoskeleton	216	3	0.6	4.96	0.0424	6.81E+00
Endocytosis	220	3	0.62	4.87	0.0424	6.68E+00

Table S20. KEGG pathway analysis of proteins selectively co-immunoprecipitating with GIT2 in *db/db* pancreatic whole-cell lysates. Significantly-populated KEGG signaling pathways, generated using the proteins co-immunoprecipitating with GIT2 in *db/db* pancreatic whole-cell lysates are depicted. For each specific KEGG pathway annotation the following parameter indices are indicated: **C** - total background number of transcripts populating the KEGG pathway; **O** – number of observed transcripts within the input dataset that are contained within the specific KEGG pathway; **E** – number of transcripts from the input dataset expected to be present at background levels; **R** – transcript enrichment factor in specific KEGG pathway, **P** – enrichment probability; **H** – hybrid score = $-\log_{10}P * R$.

KEGG Pathway	C	O	E	R	P	H
Ribosome	119	25	0.3	82.69	7.11E-40	3237.159
Selenocompound metabolism	17	2	0.04	46.31	0.0047	107.8051
Systemic lupus erythematosus	149	4	0.38	10.57	0.0047	24.60593
Phagosome	176	4	0.45	8.95	0.0047	20.83472
SNARE interactions in vesicular transport	35	2	0.09	22.49	0.0122	43.03777
Malaria	46	2	0.12	17.11	0.0176	30.01928
Protein digestion and absorption	78	2	0.2	10.09	0.0413	13.96506
ECM-receptor interaction	86	2	0.22	9.15	0.0433	12.47614

Table S21. IPA Canonical Signaling Pathway analysis of proteins selectively co-immunoprecipitating with GIT2 in WT pancreatic whole-cell lysates. Significantly-populated Canonical Pathways, generated using the proteins co-immunoprecipitating with GIT2 in WT pancreatic whole-cell lysates are depicted. For each specific signaling pathway annotation the following parameter indices are indicated: -$\log_{10}$ of the enrichment probability (-log(p-value)) and the enrichment ratio (Ratio).

**Ingenuity Canonical Pathways**	**-log(p-value)**	**Ratio**
EIF2 Signaling	9.71E+00	7.57E-02
Estrogen Receptor Signaling	2.50E+00	3.94E-02
Granzyme A Signaling	1.99E+00	1.00E-01
Maturity Onset Diabetes of Young (MODY) Signaling	1.90E+00	9.09E-02
AMPK Signaling	1.89E+00	2.79E-02
ILK Signaling	1.82E+00	2.69E-02
Caveolar-mediated Endocytosis Signaling	1.75E+00	4.23E-02
Regulation of eIF4 and p70S6K Signaling	1.57E+00	2.74E-02
Epithelial Adherens Junction Signaling	1.57E+00	2.74E-02
Actin Cytoskeleton Signaling	1.57E+00	2.30E-02
Regulation of Actin-based Motility by Rho	1.47E+00	3.30E-02
VEGF Signaling	1.46E+00	3.26E-02
Mechanisms of Viral Exit from Host Cells	1.40E+00	4.88E-02
Amyotrophic Lateral Sclerosis Signaling	1.39E+00	3.06E-02
Paxillin Signaling	1.36E+00	2.97E-02
RhoGDI Signaling	1.34E+00	2.31E-02
MSP-RON Signaling Pathway	1.31E+00	4.35E-02
Calcium Signaling	1.30E+00	2.25E-02

Table S22. IPA Canonical Signaling Pathway analysis of proteins selectively co-immunoprecipitating with GIT2 in *db/db* pancreatic whole-cell lysates. Significantly-populated Canonical Pathways, generated using the proteins co-immunoprecipitating with GIT2 in *db/db* pancreatic whole-cell lysates are depicted. For each specific signaling pathway annotation the following parameter indices are indicated: -$\log_{10}$ of the enrichment probability (-log(p-value)) and the enrichment ratio (Ratio).

**Ingenuity Canonical Pathways**	**-log(p-value)**	**Ratio**
EIF2 Signaling	2.18E+01	1.24E-01
Regulation of eIF4 and p70S6K Signaling	1.74E+00	2.74E-02
Regulation of Actin-based Motility by Rho	1.60E+00	3.30E-02
mTOR Signaling	1.39E+00	2.13E-02
